# Modeling Transcriptional Rewiring in Neutrophils Through the Course of Treated Juvenile Idiopathic Arthritis

**DOI:** 10.1038/s41598-018-26163-4

**Published:** 2018-05-17

**Authors:** Zihua Hu, Kaiyu Jiang, Mark Barton Frank, Yanmin Chen, James N. Jarvis

**Affiliations:** 1 Center for Computational Research, New York State Center of Excellence in Bioinformatics & Life Sciences, State University of New York at Buffalo, Buffalo, NY 14260 USA; 2 Department of Ophthalmology, Department of Biostatistics, Department of Medicine, State University of New York at Buffalo, Buffalo, NY 14260 USA; 3 SUNY Eye Institute, Buffalo, NY 14260 USA; 4 Department of Pediatrics, Division of Allergy/Immunology/Rheumatology, University at Buffalo, Buffalo, NY USA; 5 Arthritis & Immunology Program, Oklahoma Medical Research Foundation, Oklahoma City, OK USA; 6 Genetics, Genomics, & Bioinformatics Program, University at Buffalo, Buffalo, NY USA

## Abstract

Neutrophils in children with the polyarticular form of juvenile idiopathic arthritis (JIA) display abnormal transcriptional patterns linked to fundamental metabolic derangements. In this study, we sought to determine the effects of therapy on mRNA and miRNA expression networks in polyarticular JIA. Using exon and miRNA microarrays, we studied children with untreated active JIA (ADU, n = 35), children with active disease on therapy with methotrexate ± etanercept (ADT, n = 26), and children with inactive disease also on therapy (ID, n = 14). We compared the results to findings from healthy control children (HC, n = 35). We found substantial re-ordering of mRNA and miRNA expression networks after the initiation of therapy. Each disease state was associated with a distinct transcriptional profile, with the ADT state differing the most from HC, and ID more strongly resembling HC. Changes at the mRNA level were mirrored in changes in miRNA expression patterns. The analysis of the expression dynamics from differentially expressed genes across three disease states indicated that therapeutic response is a complex process. This process does not simply involve genes slowly correcting in a linear fashion over time. Computational modeling of miRNA and transcription factor (TF) co-regulatory networks demonstrated that combinational regulation of miRNA and TF might play an important role in dynamic transcriptome changes.

## Introduction

The polyarticular form of juvenile idiopathic arthritis (JIA) is associated with complex transcriptional abnormalities that can be observed in whole blood, peripheral blood mononuclear cells (PBMC), and neutrophils^1,2^. The neutrophil abnormalities are associated with specific aberrations in the metabolic pathways involved in myeloperoxidase and superoxide ion formation^3^. Furthermore, transcriptional abnormalities that differ from the untreated disease state persist even when children achieve remission on medication or have been disease-free and off medication for a full year. Remission, as defined by Wallace and colleagues^4,5^, is therefore not a return to normal, but rather the establishment of a new homeostatic state with its own specific transcriptional signature. This “remission signature” shows subtle differences depending on whether remission was achieved with methotrexate (MTX) or with MTX plus a TNF inhibitor^6^. While published studies provide some insights into the disease and mechanisms of therapeutic response, there are important questions that persist. For example, although it is now clear that therapy is associated with significant rearrangement of the transcriptomes of peripheral blood leukocytes^7^, little is known about the dynamics of therapeutic response. It is therefore critical to know when, how, and over what time course transcriptional changes occur. In addition, it would be useful to determine whether, when, and how the usual therapeutic dynamic is altered in those children fated to have a poor response to first-line therapy.

To answer the above questions and to develop a mechanistic understanding of treatment response in JIA at the genome level, it is necessary to have a more detailed look at the transcriptomes and functional elements in pathologically-relevant cells. One of the exciting discoveries in genome biology over the past 15 years has been how complex mammalian transcriptomes are. We now know, for example, that the human transcriptome is far more complicated than was initially anticipated from the structure of the human genome. While some controversy still exists regarding just how much of the human genome is transcribed, it is now clear that a large number of RNA transcripts do not originate from protein-coding regions. These transcripts include miRNA^8,9^ and other small RNAs, which perform important roles in the regulating transcriptional process^10^. In JIA neutrophils, we have demonstrated the complexity of mRNA-miRNA interactions and interaction networks^2,11^. Therefore, gene network rewiring is a critical component of the application of systems biology to clinical disease^12^.

In this study, we used Affymetrix exon and miRNA hybridization arrays to model the “arc” of therapeutic response in polyarticular, rheumatoid factor negative JIA. We demonstrate that therapeutic response is a complex process that does not simply involve genes normalizing transcription levels in a linear fashion over time. Instead, extensive transcriptional rewiring occurs within weeks or months of the initiation of therapy, even when the disease remains active. We further demonstrate that transcriptional response is associated with complex gene dynamics regulated by both miRNA and TFs.

## Results

### Extensive transcriptional rewiring occurs with the use of effective therapeutic agents

**Figure 1:**
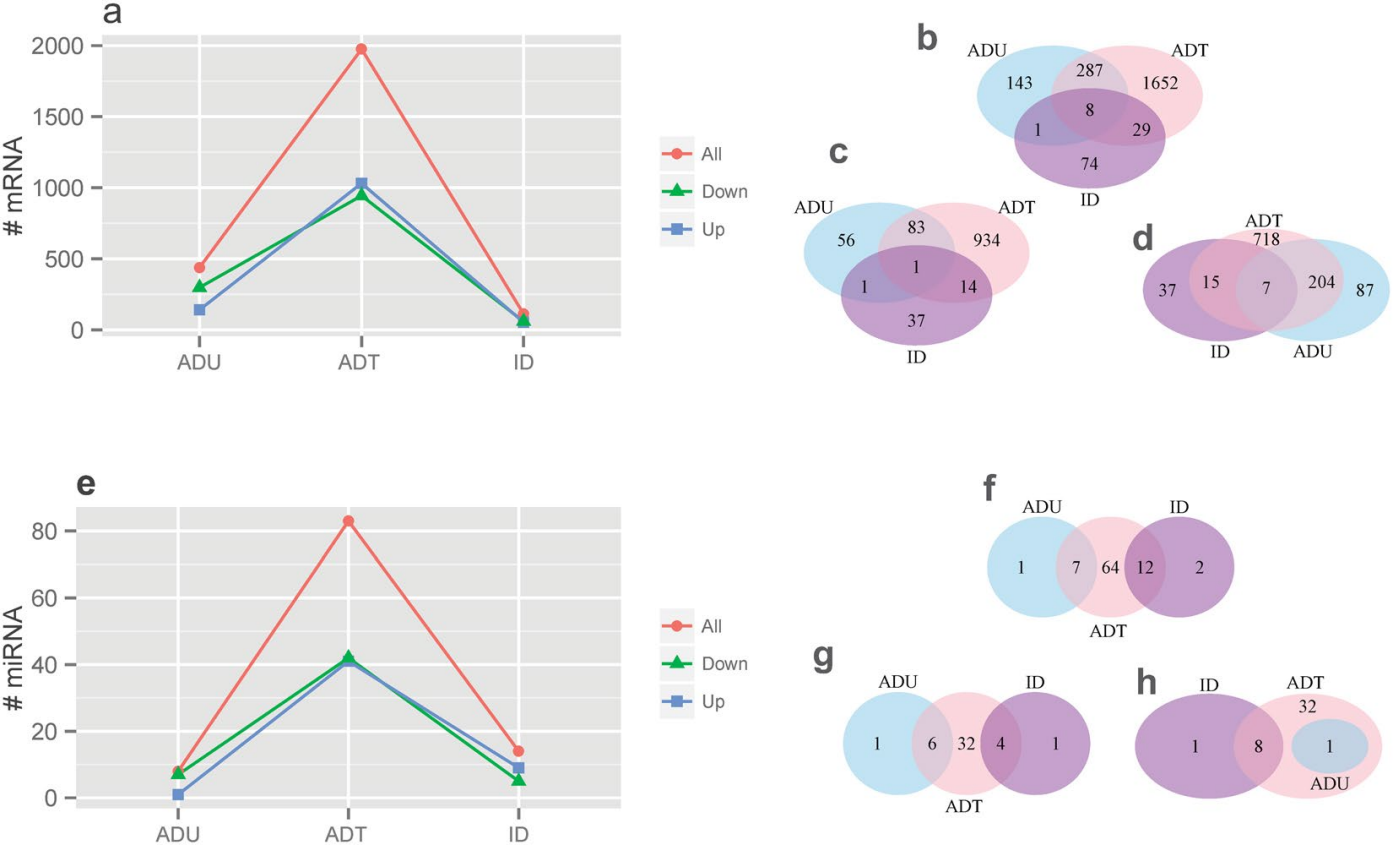
Differentially expressed mRNAs and miRNAs across 3 disease phenotypes. The number of differentially expressed mRNAs (**a**) and miRNAs (**e**) in 3 disease phenotypes when compared to HC. All: all DEGs/miRNAs; Down: down-regulated genes/miRNAs; Up: up-regulated genes/miRNAs. Venn diagrams showing the number of shared and unique mRNAs across 3 disease phenotypes for (**b**) all DEGs; (**c**) up-regulated genes; and (**d**) down-regulated genes. Significant overlap was detected between ADU and ADT (*p* = 3.7E-190), ADT and ID (*p* = 3.5E-11), and ADU and ID (*p* = 2E-03). Venn diagrams showing the number of shared and unique miRNAs across 3 disease phenotypes for (**f**) all differentially expressed miRNAs; (**g**) up-regulated miRNAs; and (**h**) down-regulated miRNAs. Significant overlap was detected between ADU and ADT (*p* = 5E-07), and ADT and ID (*p* = 2.8E-11).

We used exon and miRNA hybridization-based microarrays to generate mRNA and miRNA expression profiles for 4 phenotypes as described in the Methods section. We studied children with active, untreated disease (ADU), children with active disease on therapy with methotrexate (MTX) or MTX +a TNF inhibitor (ADT), and children who had achieved inactive disease (ID) on those same medications. Setting the cutoff *q*-value to be 0.05, we identified 439, 1976, and 112 differentially expressed genes (DEGs) from ADU, ADT, and ID, respectively, when we compared these phenotypes to HC (Fig. 1a and Supplementary Table 1). We also observed similar trends at the miRNA transcript level, for which 8, 83, and 14 differentially expressed miRNA transcripts were detected from ADU, ADT, and ID, respectively (Fig. 1e and Supplementary Table 2), again in comparison of each phenotype with HC. It is worth noting that both up-regulated and down-regulated genes and miRNA transcripts displayed similar changing patterns when compared to HC, with the largest differences seen in the ADT patients (Fig. 1a and e). We next compared the DEGs and miRNA transcripts between ADU, ADT, and ID. We found that mRNA signatures had the most significant overlap between ADU and ADT, followed by ADT and ID. That is, untreated active disease and active disease on treatment more closely resembled each other than they did with ID. Whereas 295 (67.2%, *p* = 3.7E-190) and 37 (33.04%, *p* = 3.5E-11) of DEGsdif from ADU and ID were common to those from ADT, 9 (*p* = 2E-03) common genes were found between ADU and ID, of which 8 were common across all three phenotypes (Fig. 1b). On the other hand, there was little overlap between the 3 phenotypes (i.e., ADU, ADT, ID), supporting the idea that the disease stages derived from clinical data by Wallace *et al*.^4,5^ are also distinct biological states.

Similar to mRNA, miRNA transcripts displayed the highest degree of overlap between ADU and ADT with 7 (87.5%, *p* = 5E-07) common differentially expressed miRNA out of the 8 DE miRNAs from ADU, followed by ADT and ID with 12 common differentially expressed miRNAs (85.7%, p = 2.8E-11) out of 14 from ID patients (Fig. 1f). The adjacent stages shared a higher degree of similarity, as demonstrated by the absence of overlap between ADU and ID. That is, ADT shares many transcriptional commonalities with ADU. In contrast, achievement of ID results in a distinct transcriptional state. The findings were similar when up- and down-regulated DEGs (Fig. 1c,d) and differentially expressed miRNAs (Fig. 1g,h) were considered separately.

While ADU and ADT were more alike one another than either resembled the ID state, ADT still differed considerably from ADU. That is, the initiation of treatment was associated with significant transcriptional re-organization at both the gene and miRNA level. For example, 1681 (85.1%) of the DEGs (Fig. 1b) and 76 (91.57%) of the differentially expressed miRNAs (Fig. 1f) were unique to ADT when compared to ADU. Similarly, there were 1939 DEGs (98.13%, Fig. 1b) and 71 differentially expressed miRNAs (85.5%, Fig. 1f) that were unique to ADT when compared to ID. The results also suggest that there may be a broad spectrum of potential targets of therapy, as evidenced by the large number of unique DEGs and differentially expressed miRNAs for ADT when compared to ID. This finding was further exemplified by the overall relatively low number of DEGs from ID patients.

### Functional annotation of differentially expressed genes

We used the gene ontology features of the Ingenuity software to identify specific biological pathways that are perturbed or re-ordered in JIA neutrophils at each state of treatment (i.e., ADU, ADT, and ID). The results indicated that, although DEGs were associated with a broader range of physiologic functions, no overlapping pathway was obtained among the 3 phenotypes (Supplementary Table 3), a finding that corroborates the validity of the Wallace criteria^4,5^ for staging therapeutic response in JIA.

When we compared neutrophil expression signatures of untreated children to those of healthy controls, the most significant pathway (*p* = 1.36E-03) identified was AMP-activated protein kinase signaling. This was an interesting finding, as AMPK is a critical mediator of intracellular glucose metabolism^13^. We have previously shown that JIA neutrophils display an aberrant activation pattern related to the metabolism of glucose by the hexose monophosphate shunt^3^. Furthermore, AMPK is known to regulate neutrophil chemotaxis, phagocytosis, and killing, all of which are energy-dependent^14^. The next two most common pathways identified by Ingenuity in the ADU-HC comparison were related to IL17 signaling (*p* = 1.94E-03) and Erk signaling (*p* = 2.31E-03). IL17 signaling mediates numerous neutrophil-CD4+ T cells interactions, while Erk signaling is one of the primary regulators of neutrophil activation and chemotaxis^15,16^.

**Figure 2:**
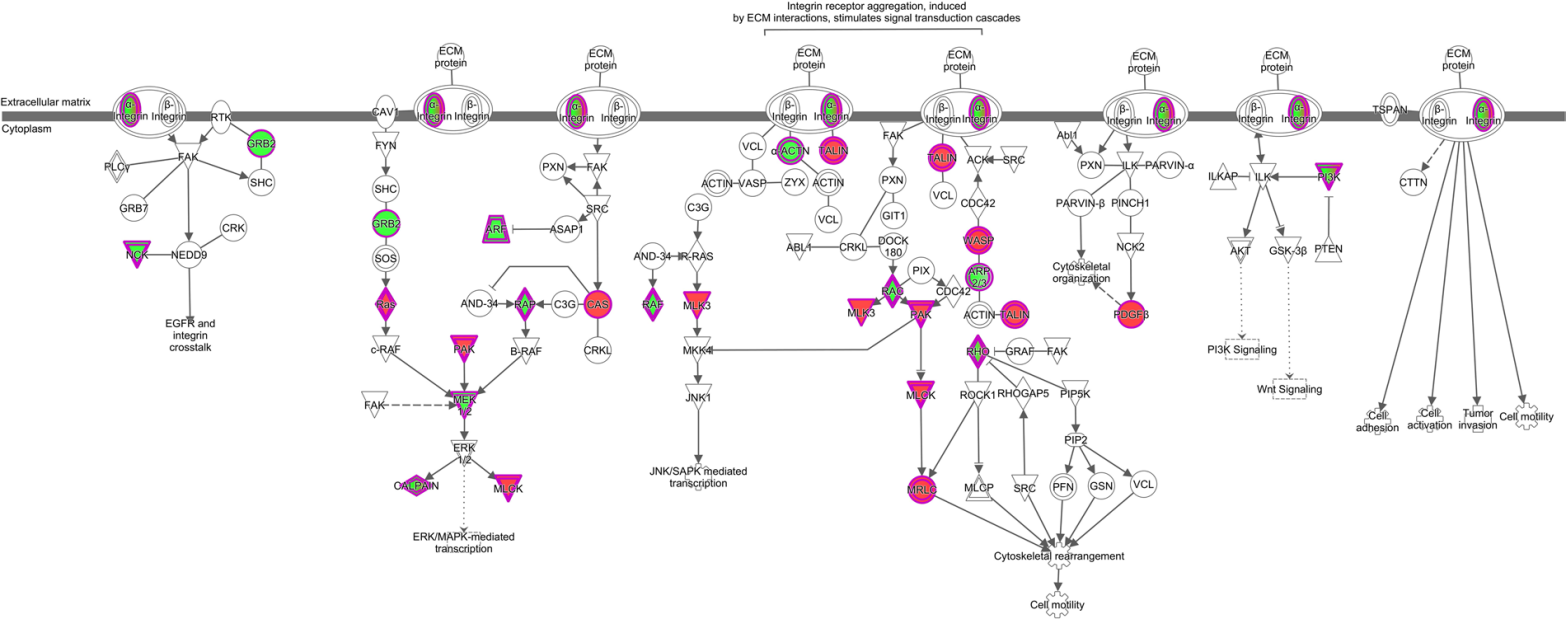
ADT DEGs enriched in integrin signaling pathway-associated transcripts. DEGs identified when comparing ADT and HC display enrichment in integrin signaling pathway transcripts as determined by IPA analysis. Genes up-regulated in ADT are highlighted in pink, and those down-regulated in green.

When we compared the ADT group with HC, the two most significant pathways identified were integrin signaling (*p* = 9.67E-04, Fig. 2 and Supplementary Table 3) and Wnt signaling pathways (*p* = 1.26E-03, Supplementary Table 3). Integrin signaling is a critical step that leads to the firm adherence of leukocytes to the endothelium and is required for extravasation^17^. Wnt signaling is required for the differential of neutrophils from CD34+ progenitors^18^. Wnt signaling also regulates neutrophil chemotaxis and chemokine production^19^. In the comparison between ID and HC, the most significant pathway that we identified (*p* = 3.02E-03) related to leukocyte extravasation (Supplementary Table 3). Taken together, all these results support the idea that pathways regulating leukocyte adherence and transmigration in JIA neutrophils remain activated after successful therapy. Our findings are consistent with earlier work describing enhanced migration in JIA neutrophils^20^. In contrast, the Erk and IL17 signals detected in active disease are extinguished by the time ID is achieved.

We next performed enrichment analyses to investigate the specific roles that might be played by transcription factors in the regulation of the DEGs during the course of therapeutic response. The results indicated that transcription factors were likely to play a more important role in the late expression networks, as evidenced by the large number of TFs associated with DEGs in ADT and ID (Supplementary Table 4). One interesting finding from this analysis was the identification of the large number of genes regulated by hepatic nuclear factor 4 A (HNF4A). We have previously shown that HNF4A, which regulates genes associated with attaining clinical remission on medication^6^, is expressed in neutrophils as well as CD4+ cells and CD8+ T cells. These findings are illustrated in Supplementary Figs 1 and 2, respectively.

### Dynamic transcriptome changes in patients induced by therapeutic agents

**Figure 3:**
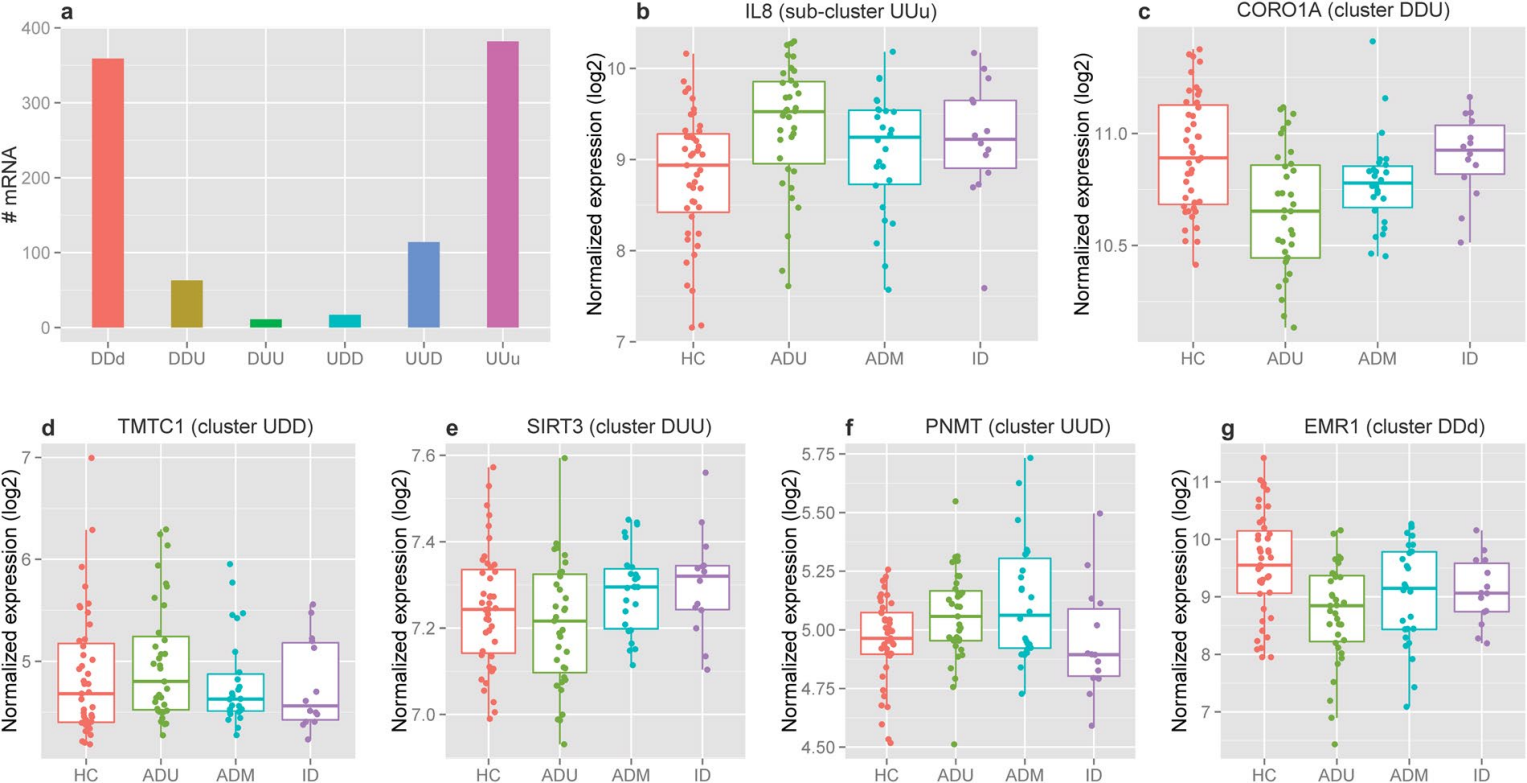
Examples of gene expression patterns for each of the 6 selected clusters. (**a**) The number of genes in the 6 selected clusters/sub-clusters; (**b**–**g**) box plots showing the expression patterns from representative genes across all 4 phenotypes for each cluster. The gene and cluster names are shown on the top of each figure, and the phenotype is depicted on the x axis.

We next examined the expression dynamics of individual genes over the course of therapy, as we have done in other work^21,22^. To classify DEGs with common dynamic patterns, we separated the DEGs into different groups based on their expression patterns, in each case comparing ADU, ADT, and ID to HC. A total of 2194 unique genes, which displayed differential expression in at least one of the three patient phenotypes, were classified into 3 groups of 8 possible clusters (Supplementary Figure 3 and Supplementary Table 5). Group one has 2 clusters with gene expression changes in the same direction in three patient phenotypes. The down-regulated cluster (cluster DDD) constituted 44.96% of all genes, of which those with smaller fold changes in ID than in ADU may be of interest (sub-cluster DDd) as targets of therapy. Further studies with larger groups of patients will be needed to confirm these findings and identify candidates that emerge. The second cluster in this group are up-regulated genes (cluster UUU), which constituted 45.2% of all genes. Similar to sub-cluster DDd those with smaller fold change in ID than in ADU (sub-cluster UUu) may also be a focus in larger studies aimed at identifying therapeutic targets. Group two has two clusters, up-regulated in ADU and ADT but down regulated in ID (cluster UUD) and down-regulated in ADU and ADT but up-regulated in ID (cluster DDU), with 114 (5.2%) and 63 genes (2.87%), respectively. Genes in this group display changes in opposite direction in ID patients, indicating a late response to therapy. The last group consists of 4 clusters with relatively small number of genes, including clusters UDD (0.77%, up-regulated in ADU but down-regulated in ADT and ID), DUU (0.5%, down-regulated in ADU but up-regulated in ADT and ID), UDU (0.45%, up-regulated in ADU and ID but down-regulated in ADT), and DUD (0.15%, down-regulated in ADU and ID but up-regulated in ADT). Genes in this group display changes at opposite direction in ADT patients, indicating an early response to therapy. Genes in individual clusters and the fold changes across three phenotypes are listed in Supplementary Table 5, and the number of genes as well as the expression patterns from a representative gene are depicted in Fig. 3. These results demonstrate broad patterns of dynamic gene expression that changes during the response to therapy. The results thus corroborate the general picture emerging from this study that transcriptional re-organization during therapeutic response is a non-linear process.

Among the genes in these dynamic clusters are several that are of specific interest to neutrophil function. For example, TRAM2 identified in the DUU cluster is a gene that operates to regulate metabolic responses to inflammation^23^, a process that we have previously shown is perturbed in JIA neutrophils^3^. GABARAP, another gene identified in the same cluster, is important in neutrophil autophagy^24^, a process which in turn is important in neutrophil NET formation^25^. TMTC1, which was identified in the UDD cluster, is a gene we have previously identified as highly expressed in active JIA^26^. TMTC1 expression levels distinguished JIA neutrophil activation patterns from those we observed in cystic fibrosis. NOD2, another gene identified in the UDD cluster, is a well described neutrophil regulator response to both bacterial^27^ and viral^11^ pathogens. Thus, the dynamic clusters reflect cogent and relevant aspects of neutrophil function. As noted above, larger and confirmatory studies will be needed to determine whether these transcripts or the proteins they encode may serve as biomarkers and/or therapeutic targets.

Functional annotation analysis indicated that all clusters except cluster UDD, UDU, and DUD were associated with enriched pathways, of which no overlap was observed between clusters (Supplementary Table 6). These findings demonstrate that each of the individual clusters represent distinct physiologic processes that are impacted by therapy, a finding consistent with our current understanding that even “targeted” therapies like anti-TNF agents may have a broad range of biological effects, not all of which are essential to therapeutic response.

### Identification of miRNAs and transcription factors involved in dynamic transcriptome changes

To elucidate the mechanism by which miRNAs and transcription factors act to regulate genes in individual clusters, we also performed analyses to identify miRNAs and transcription factors with enriched target genes out of all genes in individual clusters. Transcription factor target gene sets from GSEA (http://software.broadinstitute.org/gsea/msigdb/index.jsp) and miRNA target gene sets from TargetScan^28^ were used. We applied low stringency criteria to select enriched miRNAs without correcting for multiple tests (Fisher’s exact test p < 0.01), since we are interested in TF and miRNA co-regulatory networks for each cluster. Potential miRNAs and transcription factors regulating the dynamics of posttranscriptional and transcriptional events in each cluster are listed in Supplementary Table 7. Three clusters UUu, UDD, and DUU exhibited 9, 6, and 5 miRNAs with enriched targets genes, respectively. No overlapping miRNAs were found between clusters, suggesting that each gene cluster might be under different miRNA control. Similar to miRNAs, TF enrichment analysis indicated that, although each cluster associated with from 1 to 24 TFs (except cluster DDU), no overlapping TF was observed between each of the clusters. These results suggest that each gene cluster is under distinct mechanisms of transcriptional control.

### Expression validation of miRNAs involved in dynamic transcriptome changes

We next compared the expression patterns of miRNA with mRNA across different phenotypes to determine whether miRNAs with enriched targets in individual clusters were indeed involved in the regulation of gene expression in the cluster. If miRNAs are involved in the regulation of genes in a specific cluster, it is expected that miRNA transcripts would have expression changes in the opposite direction when compared to the targets in the cluster, as miRNAs typically mediate gene regulation by decreasing the stability of their target transcripts, leading to a reduced abundance of mRNA.

**Figure 4:**
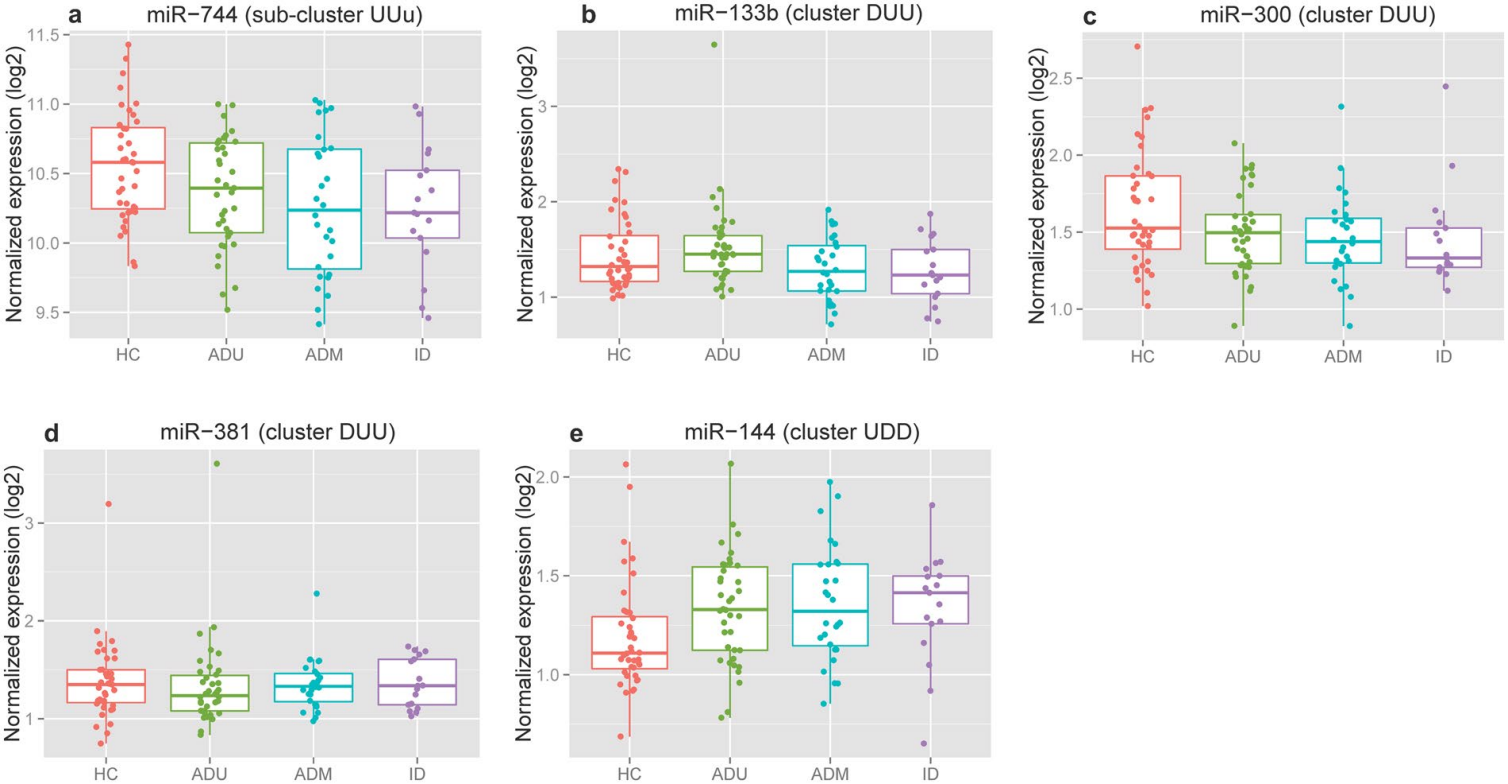
Expression patterns of miRNAs with enriched target genes in mRNA clusters. Box plot showing the expression patterns for each of 5 individual miRNAs across all 4 phenotypes. The miRNA names (e.g. miR-744) and mRNA cluster names (e.g. UUu) with enriched targets of the specific miRNA are depicted on the top of each individual figure. miR-744 (**a**) and miR-133b (**b**) display expression changes in the opposite direction to their targets from all three patient phenotypes, when compared to HC. miR-300 (**c**), miR-318 (**d**), and miR-144 (**e**) display expression changes at opposite direction to their targets in ADT and ID patients, when compared to HC.

Out of miRNAs with enriched targets genes from gene clusters UUu, UDD, and DUU (Supplementary Table 7), miR-744, miR-144, miR-133b, miR-381, and miR-300 had corresponding expression profiles from miRNA hybridization-based microarrays. We therefore performed analyses to determine whether the expression of these 5 miRNAs in the 3 patient phenotypes had opposite patterns of expression compared to their target genes in the cluster, when compared to healthy controls. These results are shown in Fig. 4, where miR-744 from sub-cluster UUu and miR-133b from cluster DUU display expression changes in the opposite direction to their targets from all three patient phenotypes. These results indicate that these 2 miRNAs are most likely involved in mediating gene expression in the corresponding clusters and therefore the regulatory networks. The remaining 3 miRNAs, miR-300 and miR-318 from cluster DUU, and miR-144 from cluster UDD, displayed expression changes in the opposite direction to their targets in ADT and ID patients. These results suggested that these 3 miRNAs are most likely involved in mediating gene expression induced by therapeutic agents.

### Constructing miRNA and TF co-regulatory networks involved in dynamic transcriptome changes

**Figure 5:**
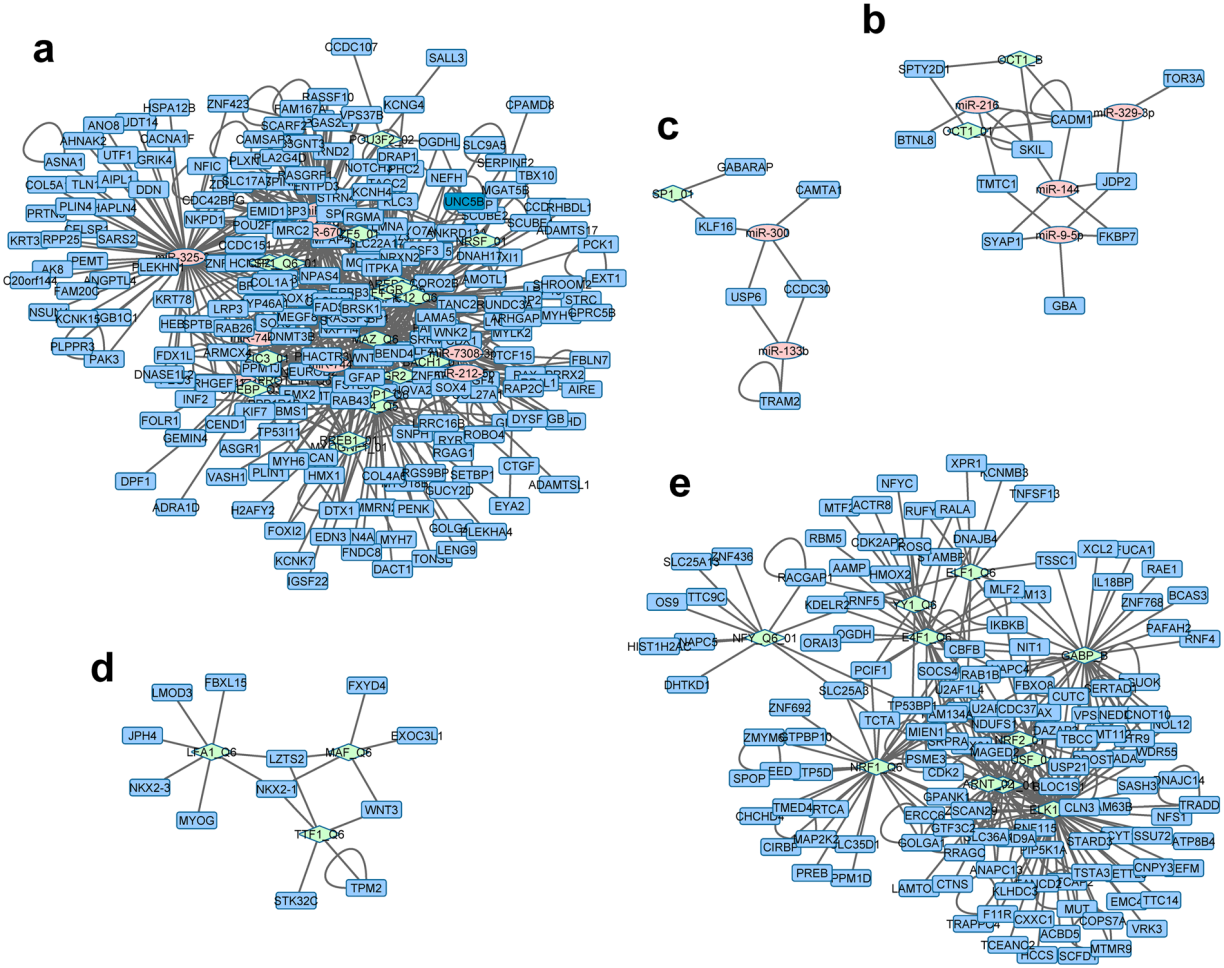
Integrated TF, miRNA, and gene regulatory networks involved in dynamic transcriptome changes. The networks were created by joining in each cluster TFs and miRNAs with their enriched target genes and interacting proteins. (**a**) Network from cluster UUu; (**b**) network from cluster DUU; (**c**) network from cluster UDD; (**d**) network from cluster UUD; (**e**) network from cluster DDd. Rectangles in blue: target genes or interacting proteins; circles in pink: miRNAs; diamonds in green: transcription factors.

Next, we constructed miRNA and TF co-regulatory networks, which highlighted the combinational regulation of miRNA and TF in each cluster. The networks were created by joining in each cluster miRNAs and TFs with the enriched target genes as well as interacting proteins^29^ using Cytoscape^30^. If there was more than one miRNA from the same functional family (e.g. miR-300 and miR-381), only one was used in network construction. Figure 5 shows networks from 5 clusters, each containing two or three types of nodes: miRNA, TF, and target genes. The number of gene, miRNA, TF nodes, and edges for each network are summarized in Supplementary Table 8, and the node relationships are listed in Supplementary Table 9. Whereas regulatory networks from three clusters UUu, UDD, and DUU possess three types of nodes, regulatory networks from cluster UUD and DDd include only TF and gene nodes. It is interesting to note that miRNAs, TFs, and genes form single regulatory networks in each of the 5 clusters, indicating a tight regulatory connection between miRNAs and the binding of TFs to their target genes.

### Validation of gene expression results

**Figure 6:**
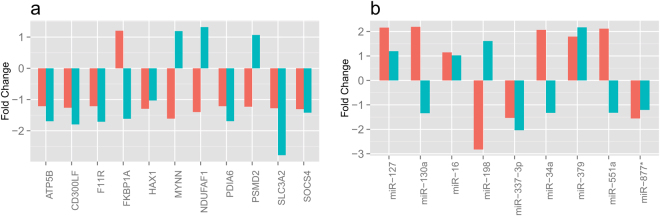
Validation of microarray data for selected genes and miRNAs by real-time PCR. DEGs (**a**) and miRNAs (**b**) from ADT patients who were compared to HC.

To validate the differences in gene expression and miRNA expression observed in the microarray experiments between JIA patients and HC individuals, we performed qRT-PCR analyses using an independent cohort of 8 ADT patients and 8 HC individuals. We randomly selected 11 genes and 9 miRNA transcripts that were significantly and differentially expressed between ADT and HC for qRT-PCR analysis. The results indicated that 7 of 11 mRNA genes (Fig. 6a) and 5 of 9 miRNA transcripts (Fig. 6b) had similar expression changes in qRT-PCR, when compared to expression changes in microarray analysis from ADT.

## Discussion

One of the many interesting insights to emerge from the Human Genome Project and its successors, ENCODE and Roadmap Epigenomics, has been the discovery of the complexity^10^, layers of organization, and control in the transcriptomes of mammalian cells^31–33^. We and others have hypothesized that human diseases may emerge because of genetic and/or epigenetic alterations that disrupt the mechanisms through which transcription is coordinated across the genome^34–37^, resulting in loss of transcription network organization. We believe that understanding the organization of transcriptional networks, and how those networks are perturbed in disease states, is critical to achieving the promise of genomic medicine and developing personalized therapies^12^.

miRNAs, which are known to be of particular importance in cells of the immune system^38–40^, are among the many regulators of transcriptional control and network organization. While miRNA have been extensively studied in the context of rheumatic diseases^9,41^, most studies have focused on describing miRNA repertoires of pathologically relevant cells and comparing expression patterns with healthy controls^42^. These studies generally did not investigate the complex transcriptional rewiring that might emerge as the result of differential expression of miRNA and/or their target genes^43,44^. We have recently reported that neutrophils of children with untreated JIA display numerous differences in their transcriptomes compared to either healthy children or children with cystic fibrosis, another illness characterized by chronic, indolent inflammation in soft tissues^11^. These differences were reflected in remodeled mRNA-miRNA networks that were largely phenotype-specific. The current study was undertaken to determine whether/how treatment with methotrexate and/or etanercept, agents commonly used to treat polyarticular JIA, might alter the mRNA-miRNA networks we observed in untreated patients.

In this paper, we demonstrated that effective therapies for JIA were associated with extensive rewiring of mRNA-miRNA networks in JIA. The initiation of therapy was associated with expression differences in ~2,000 genes, half of which showed higher expression levels and the other half lower expression levels compared to the untreated disease state. Changes at the mRNA level were mirrored in changes in miRNA expression patterns (Fig. 1). This initial phase of extensive transcriptional re-organization was then followed by a period of further re-equilibration, at the time children achieve ID status as defined by Wallace and colleagues^4,5^. We specifically noted that transcriptional re-organization resulted in the ablation of the AMPK, IL17, and Erk expression signatures identified in the untreated disease. The AMPK pathway can be manipulated pharmacologically by enhancing AMPK activity, such as the use of metformin in type 2 diabetes^13^. There is also considerable interest in targeting mitogen-activated protein kinase pathways in both cancer^45^ and rheumatoid arthritis^46,47^, and IL17 pathway inhibitors are likely to emerge as either alternatives or adjuncts to TNF inhibition^48–50^. It is important to note that the transcriptomes and transcriptional networks of children in the ID state did not return to the pattern seen in healthy children, consistent with our previous observations^6^. Specifically, gene networks that regulate neutrophil adhesion and extravasation remained active even after children had achieved ID status.

We also noted that these patterns emerged regardless of whether ID was achieved with methotrexate alone or methotrexate plus the TNF inhibitor etanercept. The observed patterns and clusters appeared to be more specific to disease activity than to the specific pharmacologic agents, suggesting that there are specific immunologic “set points” that must be attained in order to achieve disease control. Individual patient responses likely reflect the underlying genetic and epigenetic factors that determine whether those set points can be achieved with methotrexate alone or only with the addition of a TNF inhibitor^51^. That being said, it seems possible that more subtle effects would be observed if we directly compared MTX-treated patients with patients treated with MTX and etanercept. We observed such differences in neutrophils, but not PBMC, of children who had achieved the clinical remission on medication state in our previous study.

We note that the transcriptional rewiring that occurs during therapeutic response is not driven completely by the re-organization of mRNA-miRNA networks. Computational modeling strongly supports the idea that the orchestration of specific TFs also plays a role in this process^4,5^. This finding is consistent with our recent observation in CD4+ T cells of patients with JIA, where the transition from ADT to clinical remission on medication is associated with extensive chromatin re-organization detected by ATACseq (unpublished observation). Taken together, our findings indicate that the transcriptional re-organization that accompanies therapeutic response in JIA is a highly complex process. Given the multiple layers of reorganization that we have identified (chromatin accessibility, TF binding, reorganization of miRNA-RNA networks), we believe that these findings explain the diversity of therapeutic responses (e.g., time course, relative refractoriness to specific agents) observed in clinical practice.

In conclusion, we have found that therapeutic response in JIA is characterized by specific transcriptional phases that correspond to the clinically-derived phases developed by Wallace *et al*.^4,5^. The transcriptional re-organization, which is discerned even in a heterogeneous population who are treated with different therapeutic regimens, involves extensive rewiring of mRNA-miRNA regulatory networks. Computational analysis suggests that this transcriptional reorganization may also be mediated by alterations in chromatin accessibility, but this possibility must be confirmed experimentally.

## Methods

### Patients and patient characteristics

Patients and controls were recruited from the OU Children’s Physicians’ clinics at the University of Oklahoma Health Sciences Center in Oklahoma City, OK. IRB approval was obtained for this study. Parents of all patients and controls executed consent documents prior to providing specimens; where appropriate, child assent was also obtained. This study was carried out in conformance with the IRB-approved protocol.

We performed a cross-sectional study of children at different stages of treatment and treatment response in children who had the polyarticular, RF negative phenotype of JIA as defined by International League of Associations for Rheumatology (ILAR). Classifications for disease activity and disease state were made according to criteria developed by Wallace *et al*. and accepted by an international consensus panel. This was a cross sectional study, for which we studied 38 patients (35 for mRNA profiling) who had active, untreated disease (ADU) and were seen within 6 weeks of the onset of symptoms. In addition, we studied 28 (26 for mRNA profiling) children with active disease (ADT) who had been on therapy with methotrexate (MTX) or MTX plus a TNF inhibitor (typically etanercept) for durations ranging from 6 weeks to 22 months. Active disease was designated as the presence of warmth and synovial thickening in at least one joint at the time the clinical specimen was obtained^4,5^. Finally, we studied 17 (14 for mRNA profiling) children who fit standard criteria for inactive disease; that is, these children had no evidence of synovitis and did not have fever, rash, lymphadenopathy, splenomegaly, active uveitis. Criteria for inactive disease also included normal laboratory findings for erythrocyte sedimentation rates and/or C reactive protein, as well as a physician global assessment score indicating no active disease^4^. Findings from children with JIA were compared with findings from 43 (40 for miRNA transcript profiling) healthy control children recruited from the OU Children’s Physician General Pediatrics clinic.

### Cell Isolation

Whole blood was drawn into 10 mL citrated CPT tubes (Becton Dickinson, Franklin Lakes, NJ). Specimens were taken immediately to the Pediatric Rheumatology Research laboratories at the University of Oklahoma Health Sciences Center, and cell separation procedures were started within one hour from the time the specimen was drawn. Peripheral blood mononuclear cells (PBMC) were separated from granulocytes and red blood cells by density-gradient centrifugation. PBMC and granulocytes were then immediately placed in TRIzol® reagent (Invitrogen, Carlsbad, CA) and stored at −80 °C. Flow cytometry analysis of cells isolated in this fashion are ≥98% CD66b+ and contain no CD14+ cells, as we have previously reported^11^.

### RNA Isolation, Labeling, and Gene Expression Profiling

Total RNA was extracted from Trizol® reagent according to manufacturer’s directions. RNA was further purified using RNeasy MinElute Cleanup kit including a DNase digest according to the manufacturer’s instructions (QIAGEN, Valencia, CA). RNA was quantified spectrophotometrically (Nanodrop) and assessed for quality by capillary gel electrophoresis (Agilent 2100 Bioanalyzer; Agilent Technologies, Inc., Palo Alto, CA).

RNA samples were processed using GeneChip WT Terminal Labeling and Controls Kit and hybridized to Human Exon 1.0 ST array according to the manufacturer’s protocol (Affymetrix, Santa Clara, CA, USA). GeneChips™ were washed and stained using an Affymetrix automated GeneChip™ 450 fluidics station and scanned with an Affymetrix 3000 7 G scanner.

### GeneChip data processing and analysis

To generate expression summary values from Affymetrix Exon and miRNA arrays, RMA software in the “Affy” package of Bioconductor in the R statistical computing environment (http://www.r-project.org) was used with its default settings. For individual genes with multiple probe sets on the array and/or isoforms the average expression was computed within the same sample. DEGs and miRNA transcripts between patient phenotypes and heathy control were obtained using t-tests. For multiple test correction, the false discovery rate for both genes and miRNAs (q-value <= 0.05) was controlled by adjusting the p-values using the following formula:

$${\bf{q}}-{\bf{value}}=\frac{{\boldsymbol{N}}\,{\boldsymbol{\ast }}\,{{\boldsymbol{P}}}_{{\boldsymbol{c}}}}{{\boldsymbol{R}}}$$

where *N* is the number of genes/miRNAs in the test, and *R* is the ascending rank order of the respective *p*-value at certain p-value cutoff (*P*_*c*_).

### Functional Annotation

Functional enrichment analyses of DEGs was undertaken using the tool of Ingenuity Pathway Analysis (https://www.qiagenbioinformatics.com/products/ingenuity-pathway-analysis/).

### Gene expression validatio by quantitative real-time RT-PCR

To corroborate the microarray results, we analyzed the expression of single mRNAs and miRNA transcripts using quantitative real time-polymerase chain reaction (qRT-PCR) in an independent cohort samples as described in^11^. In brief, total RNA was reverse transcribed with iScript^TM^ cDNA synthesis kit according to the directions of the manufacturer (Bio-Rad, Hercules, CA, USA). qRT-PCR was then performed using SYBR Green reagents on a StepOne Plus (for the testing group; Applied Biosystems, Foster City, CA, USA). After gene amplification using specific primers (Supplementary Table 10), gene-specific amplification was confirmed by a single peak in the ABI Dissociation Curve software. Average threshold cycle (Ct) values for GAPDH (run in parallel reactions to the genes of interest) were then used to normalize average Ct values of the gene of interest. These values were used to calculate averages for each group (healthy control or patient subsets), and the relative ΔCt was used to calculate fold-change values between groups. All primers were tested and displayed an efficiency of amplification at 98.18% (±SD 5.19%).

To validate miRNA expression, qRT-PCR was performed using the TaqMan MicroRNA Reverse transcription kit (Applied Biosystems, USA), miRNA-specific stem-loop primers (TaqMan® microRNA assay kit, Applied Biosystems, USA) and the TaqMan Universal Master Mix II, no UNG (Applied Biosystems, USA), as described previously^11^. In brief, qPCRs for each individual miRNA were carried out in duplicate using the StepOne Plus PCR system (Applied Biosystems) with specific target sequence (Supplementary Table 11). The temperature profile consisted of an initial step at 95 °C for 10 minutes, followed by 40 cycles of 95 °C for 15 seconds, 60 °C for 1 minute. miRNAs of hsa-miR-191 was used as miRNA normalizers, since it displayed no statistically significant difference among groups, had the smallest variation across ADT (CV = 0.0047) and HC samples (CV = 0.008), and had relatively high expression values. The threshold cycle (Ct) values from the hsa-miR-191 miRNA (running in parallel reactions to the miRNA of interest) were used to normalize average Ct values of the miRNAs of interest. These values were used to calculate averages for each group (healthy control or patient subsets), and the relative ΔCt was used to calculate fold-change values between the groups.

## Electronic supplementary material

Supplementary Figures and Tables
