# Supplementary material for: Modeling Transcriptional Rewiring in Neutrophils Through the Course of Treated Juvenile Idiopathic Arthritis
Source: Sci Rep. 2018 May 17;8:7805. doi: 10.1038/s41598-018-26163-4 (PMC5958082; doi:10.1038/s41598-018-26163-4)
Supplement: Supplementary file 1 — Supplementary Figures and Tables [file 41598_2018_26163_MOESM1_ESM.pdf]

Supplementary Information

Modeling Transcriptional Rewiring in Neutrophils Through the Course of Treated Juvenile Idiopathic Arthritis

Zihua Hu[1,*,+], Kaiyu Jiang[2,+], Mark Barton Frank[3], Yanmin Chen[2], James N. Jarvis[2,4,*]

[1]Center for Computational Research, New York State Center of Excellence in Bioinformatics & Life Sciences, State University of New York at Buffalo, Buffalo NY 14260, USA; Department of Ophthalmology, Department of Biostatistics, Department of Medicine, State University of New York at Buffalo, Buffalo NY 14260 USA and SUNY Eye Institute, Buffalo NY 14260 USA

[2]Department of Pediatrics, Division of Allergy/Immunology/Rheumatology, University at Buffalo, Buffalo, NY

[3]Arthritis & Immunology Program, Oklahoma Medical Research Foundation, Oklahoma City, OK

[+]These authors contributed equally to this work

[4]Genetics, Genomics, & Bioinformatics Program, University at Buffalo, Buffalo, NY

[*]Correspondence and requests for materials should be addressed to J.N.J. (e-mail: jamesjar@buffalo.edu) or to Z.H. (e-mail: zihuahu@ccr.buffalo.edu)

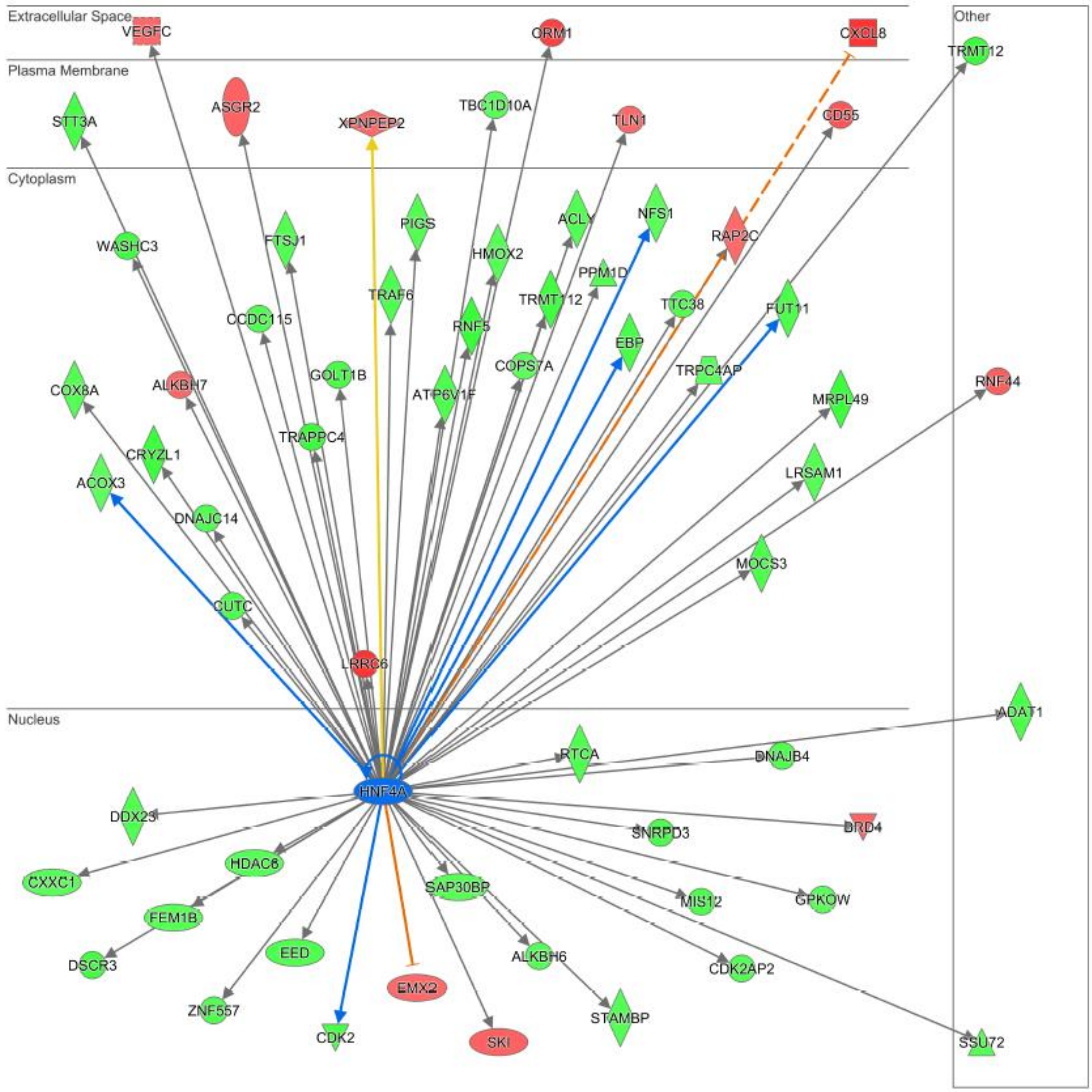

Supplementary Figure 1. HNF4A regulated DEGs from ADU. 64 genes that significantly changed between ADU and HC were directly regulated by HNF4A, as analyzed using IPA. Genes up-regulated in ADU are highlighted in red, and down-regulated in green. Also shown is the cellular location of the regulated genes.

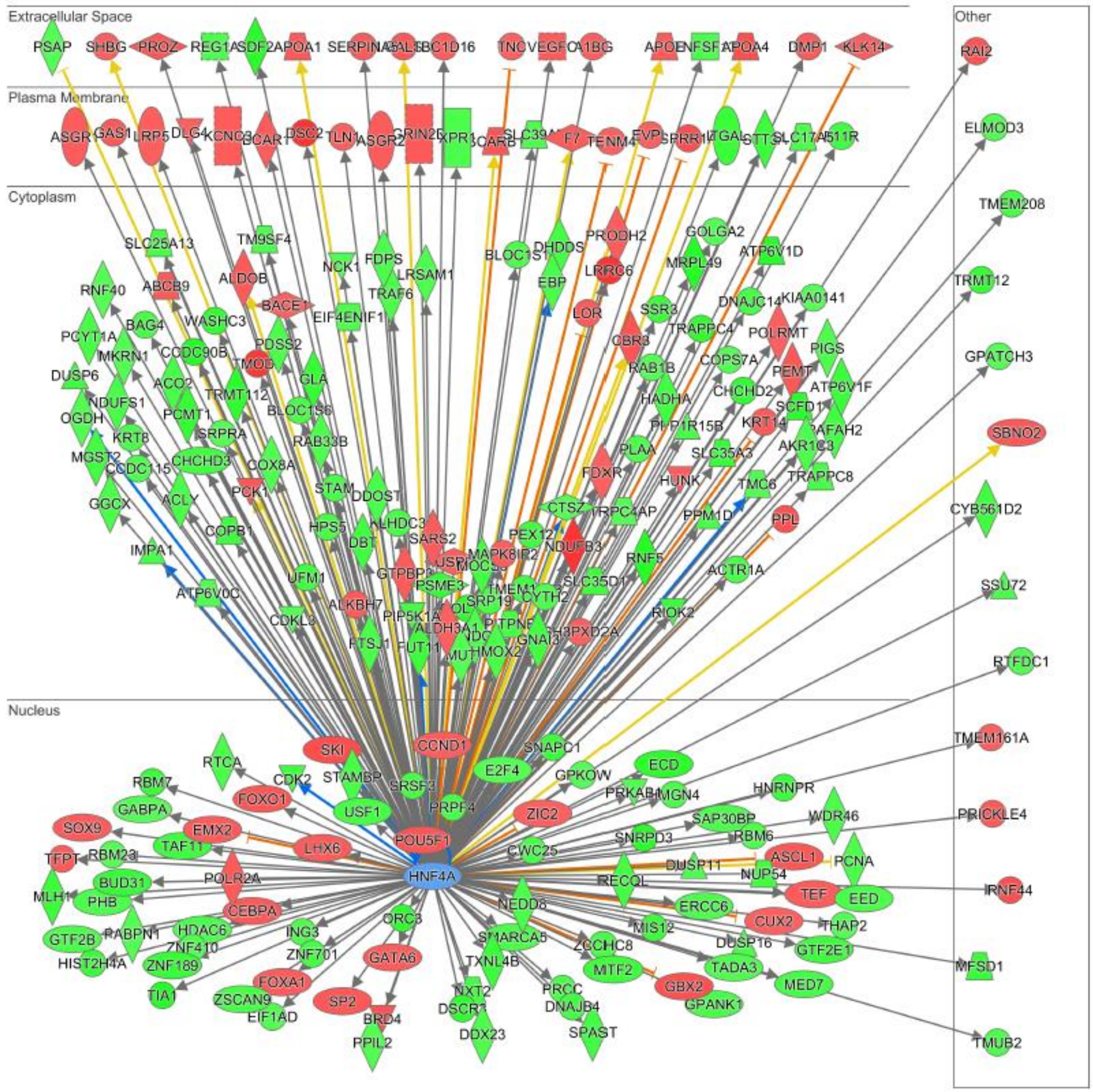

Supplementary Figure 2. HNF4A regulated DEGs from ADT. 244 genes that significantly changed between ADT and HC were directly regulated by HNF4A, as analyzed using IPA. Genes up-regulated in ADT are highlighted in red, and down-regulated in green. Also shown is the cellular location of the regulated genes.

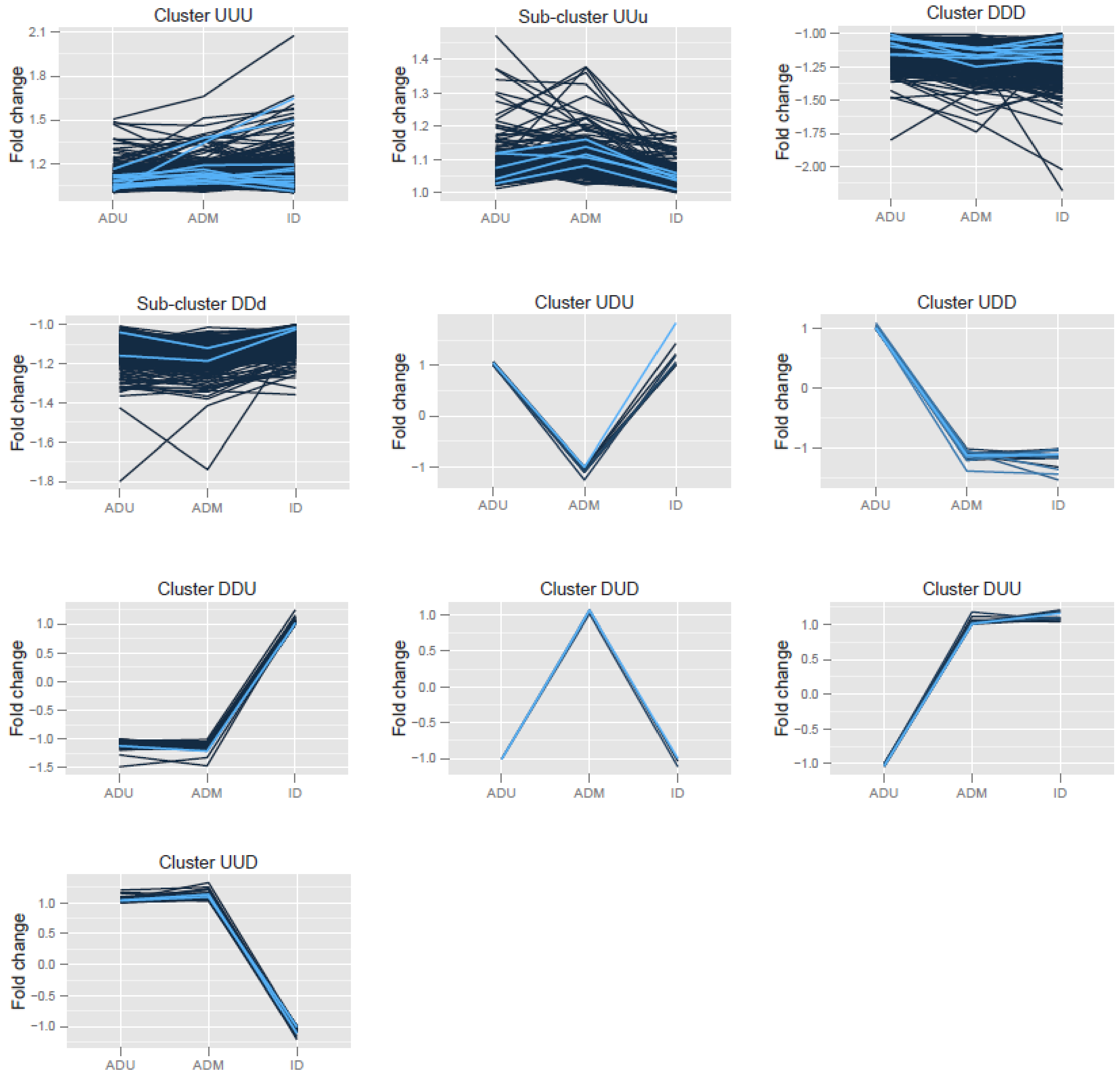

Supplementary Figure 3. **Expression pattern of clusters across 3 disease phenotypes.** 2194 genes**,** which displayed differential expression in at least one of the three patient phenotypes, were classified into 8 possible clusters based on their expression patterns, when compared to heathy controls (HC). Cluster UUU: genes were up-regulated in all three phenotypes; Sub-cluster UUu: genes in cluster UUU with smaller fold changes in ID than in ADT; Cluster UUD: genes were up-regulated in ADU and ADT but down-regulated in ID; Cluster UDU: genes were up-regulated in ADU and ID but down-regulated in ADT; Cluster UDD: genes were up-regulated in ADU but down-regulated in ADT and ID; Cluster DDU: genes were down-regulated in ADU and ADT but up-regulated in ID; Cluster DUD: genes were down-regulated in ADU and ID but up-regulated in ADT; Cluster DUU: genes were down-regulated in ADU but up-regulated in ADT and ID; Cluster DDD: genes were down-regulated in all three phenotypes; Sub-cluster DDd: genes in cluster DDD with smaller fold changes in ID than in ADT.

Supplementary table 1. Differentially expressed genes from ADU, ADT, and ID

Gene symbol	Mean (test sample)	SD (test sample)	Mean (HC)	SD (HC)	*p* value	Fold change	FDR	Phenotype
RNF5	5.91	0.232	6.306	0.281	1.96E-09	-1.32	3.46E-05	ADU
ZMAT2	7.902	0.2	7.583	0.23	6.08E-09	1.25	5.37E-05	ADU
UCK1	7.546	0.152	7.767	0.153	1.42E-08	-1.17	8.35E-05	ADU
CREBBP	10.063	0.136	9.899	0.097	1.09E-07	1.12	2.75E-04	ADU
ROMO1	6.961	0.305	6.506	0.377	9.74E-08	1.37	2.87E-04	ADU
ADRM1	7.543	0.144	7.729	0.124	6.69E-08	-1.14	2.96E-04	ADU
CD300LF	9.102	0.317	9.509	0.276	9.31E-08	-1.33	3.29E-04	ADU
PCIF1	8.041	0.173	8.253	0.149	2.52E-07	-1.16	4.06E-04	ADU
DYNLRB1	8.104	0.195	8.38	0.235	2.48E-07	-1.21	4.38E-04	ADU
ZBTB3	5.09	0.179	5.355	0.231	2.24E-07	-1.20	4.39E-04	ADU
DSCR3	8.118	0.297	8.46	0.192	2.24E-07	-1.27	4.94E-04	ADU
PCBD2	4.571	0.252	4.858	0.189	5.74E-07	-1.22	8.45E-04	ADU
DCAF5	6.852	0.12	7.002	0.123	6.42E-07	-1.11	8.73E-04	ADU
ARID1A	8.872	0.102	8.753	0.093	1.10E-06	1.09	1.39E-03	ADU
TRAPPC4	6.469	0.371	6.865	0.241	1.18E-06	-1.32	1.39E-03	ADU
SSNA1	6.711	0.225	6.975	0.215	1.48E-06	-1.20	1.45E-03	ADU
AAMP	5.647	0.131	5.848	0.203	1.36E-06	-1.15	1.50E-03	ADU
TAX1BP3	6.523	0.21	6.797	0.253	1.46E-06	-1.21	1.52E-03	ADU
AP2M1	8.544	0.235	8.249	0.275	2.45E-06	1.23	1.80E-03	ADU
MLX	8.447	0.195	8.669	0.18	2.16E-06	-1.17	1.82E-03	ADU
SOCS4	4.684	0.271	4.988	0.241	2.08E-06	-1.23	1.84E-03	ADU
RAB43	7.991	0.18	7.782	0.177	2.29E-06	1.16	1.84E-03	ADU
TRMT112	8.877	0.302	9.213	0.259	2.01E-06	-1.26	1.87E-03	ADU
MRPL49	6.758	0.297	7.121	0.329	2.43E-06	-1.29	1.87E-03	ADU
ITPR2	10.077	0.181	10.329	0.257	2.83E-06	-1.19	2.00E-03	ADU
PIN1	6.815	0.254	7.098	0.238	3.43E-06	-1.22	2.33E-03	ADU
ATXN3	7.077	0.579	7.647	0.332	3.82E-06	-1.48	2.50E-03	ADU
BRD4	8.079	0.15	7.929	0.1	4.56E-06	1.11	2.88E-03	ADU
BAZ2A	9.657	0.181	9.475	0.134	6.35E-06	1.13	3.74E-03	ADU
CDC37	7.36	0.24	7.599	0.169	6.21E-06	-1.18	3.79E-03	ADU
CREB5	8.542	0.142	8.369	0.174	6.91E-06	1.13	3.94E-03	ADU
CDK2AP2	6.949	0.155	7.134	0.183	7.58E-06	-1.14	4.19E-03	ADU
VPS37B	7.278	0.159	7.112	0.14	8.21E-06	1.12	4.40E-03	ADU
HCCS	5.717	0.253	5.979	0.225	9.84E-06	-1.20	5.12E-03	ADU
FNDC8	4.466	0.163	4.303	0.133	1.04E-05	1.12	5.13E-03	ADU
TMEM9B	7.266	0.235	7.521	0.243	1.21E-05	-1.19	5.23E-03	ADU
ARL8A	9.704	0.167	9.491	0.233	1.15E-05	1.16	5.23E-03	ADU
CCDC115	5.645	0.21	5.849	0.155	1.19E-05	-1.15	5.25E-03	ADU
S100PBP	6.745	0.267	7.009	0.21	1.13E-05	-1.20	5.25E-03	ADU
CTNS	5.736	0.24	6.098	0.423	1.04E-05	-1.29	5.25E-03	ADU
COG7	5.341	0.188	5.553	0.209	1.11E-05	-1.16	5.31E-03	ADU
CBFB	6.673	0.259	6.92	0.192	1.46E-05	-1.19	5.62E-03	ADU
NTAN1	6.269	0.428	6.718	0.422	1.46E-05	-1.37	5.72E-03	ADU

GOLGA1	6.331	0.205	6.525	0.144	1.44E-05	-1.14	5.78E-03	ADU
MLL3	9.691	0.193	9.512	0.125	1.42E-05	1.13	5.85E-03	ADU
CUTC	6.97	0.242	7.2	0.172	1.40E-05	-1.17	5.90E-03	ADU
FUT11	7.006	0.258	7.262	0.223	1.71E-05	-1.19	6.42E-03	ADU
RPS11	7.3	0.371	6.919	0.356	1.80E-05	1.30	6.63E-03	ADU
VPS33A	5.897	0.277	6.155	0.199	2.00E-05	-1.20	7.22E-03	ADU
HN1	9.048	0.205	9.278	0.243	2.13E-05	-1.17	7.24E-03	ADU
ZNF423	4.491	0.115	4.377	0.101	2.06E-05	1.08	7.28E-03	ADU
EMC4	7.994	0.247	8.248	0.244	2.11E-05	-1.19	7.30E-03	ADU
STARD3	8.082	0.154	8.231	0.136	2.82E-05	-1.11	9.23E-03	ADU
RHBDD2	8.997	0.179	9.198	0.217	2.81E-05	-1.15	9.36E-03	ADU
VPS72	8.014	0.227	8.227	0.182	2.93E-05	-1.16	9.43E-03	ADU
LILRA2	9.833	0.238	9.588	0.248	3.32E-05	1.19	9.94E-03	ADU
RBX1	6.113	0.303	6.433	0.337	3.26E-05	-1.25	9.94E-03	ADU
RNF44	8.229	0.211	8.017	0.208	3.17E-05	1.16	1.00E-02	ADU
ZDHHC13	6.312	0.357	6.662	0.331	3.24E-05	-1.27	1.00E-02	ADU
UBE2Q1	8.051	0.158	8.202	0.137	3.42E-05	-1.11	1.01E-02	ADU
SLC9A6	5.739	0.268	5.975	0.176	3.66E-05	-1.18	1.06E-02	ADU
ACLY	7.618	0.218	7.809	0.142	3.87E-05	-1.14	1.10E-02	ADU
BCL3	9.747	0.248	9.518	0.201	4.04E-05	1.17	1.13E-02	ADU
FUCA1	5.79	0.301	6.086	0.295	4.25E-05	-1.23	1.17E-02	ADU
NFS1	6.27	0.142	6.401	0.118	4.73E-05	-1.10	1.18E-02	ADU
HINFP	6.489	0.181	6.678	0.206	4.81E-05	-1.14	1.18E-02	ADU
MGAT2	4.713	0.177	4.89	0.181	4.58E-05	-1.13	1.19E-02	ADU
CRYZL1	4.962	0.355	5.317	0.369	4.73E-05	-1.28	1.20E-02	ADU
ORAI1	7.464	0.132	7.601	0.15	4.96E-05	-1.10	1.20E-02	ADU
C16orf93	6.886	0.389	7.244	0.322	4.56E-05	-1.28	1.20E-02	ADU
MAPKAPK2	8.006	0.176	7.829	0.183	4.49E-05	1.13	1.20E-02	ADU
ERP29	6.543	0.41	6.924	0.348	4.45E-05	-1.30	1.21E-02	ADU
SH3GL1	7.766	0.114	7.666	0.077	4.73E-05	1.07	1.21E-02	ADU
SMUG1	5.595	0.219	5.789	0.161	5.32E-05	-1.14	1.22E-02	ADU
FIBP	6.505	0.19	6.693	0.195	5.41E-05	-1.14	1.23E-02	ADU
TCHP	6.019	0.244	6.241	0.2	5.29E-05	-1.17	1.23E-02	ADU
MAP4K4	8.826	0.241	8.569	0.291	5.51E-05	1.19	1.23E-02	ADU
CHRFAM7A	5.977	0.232	6.217	0.262	5.27E-05	-1.18	1.24E-02	ADU
BLVRA	5.399	0.197	5.625	0.269	5.24E-05	-1.17	1.25E-02	ADU
TNFAIP8L2-SCNM	7.518	0.22	7.731	0.218	5.77E-05	-1.16	1.28E-02	ADU
RRBP1	6.448	0.123	6.588	0.167	6.04E-05	-1.10	1.32E-02	ADU
OTUB1	7.373	0.151	7.506	0.115	6.17E-05	-1.10	1.33E-02	ADU
ADCK4	6.626	0.177	6.783	0.14	6.27E-05	-1.11	1.34E-02	ADU
CORO1A	10.653	0.273	10.91	0.26	7.32E-05	-1.19	1.39E-02	ADU
TBC1D20	7.606	0.188	7.761	0.112	7.25E-05	-1.11	1.39E-02	ADU
LRPAP1	7.569	0.166	7.726	0.16	7.41E-05	-1.11	1.39E-02	ADU
MANBAL	7.932	0.303	8.197	0.232	7.20E-05	-1.20	1.40E-02	ADU
EMR1	8.721	0.874	9.569	0.91	7.85E-05	-1.80	1.40E-02	ADU
CD55	9.727	0.285	9.458	0.278	7.53E-05	1.20	1.40E-02	ADU
ATP6V1F	7.668	0.235	7.895	0.242	7.62E-05	-1.17	1.40E-02	ADU

WAS	10.026	0.194	9.85	0.166	6.67E-05	1.13	1.40E-02	ADU
ACBD5	6.399	0.22	6.584	0.144	7.20E-05	-1.14	1.41E-02	ADU
COPE	8.526	0.197	8.702	0.165	7.84E-05	-1.13	1.42E-02	ADU
DERA	4.805	0.178	4.997	0.228	7.77E-05	-1.14	1.42E-02	ADU
INHBB	6.382	0.215	6.171	0.226	7.16E-05	1.16	1.42E-02	ADU
RASSF3	9.292	0.212	9.066	0.267	8.20E-05	1.17	1.42E-02	ADU
COX8A	8.221	0.202	8.394	0.149	8.30E-05	-1.13	1.42E-02	ADU
CDK5RAP2	5.569	0.278	5.83	0.272	8.62E-05	-1.20	1.42E-02	ADU
IRF3	7.196	0.129	7.316	0.124	8.39E-05	-1.09	1.43E-02	ADU
KRTCAP2	7.447	0.239	7.674	0.237	8.15E-05	-1.17	1.43E-02	ADU
PLXNB2	6.134	0.491	5.678	0.473	9.05E-05	1.37	1.43E-02	ADU
OS9	9.601	0.235	9.812	0.203	8.74E-05	-1.16	1.43E-02	ADU
RAB4B	7.618	0.193	7.832	0.262	8.91E-05	-1.16	1.43E-02	ADU
PSPH	4.653	0.398	5.028	0.398	9.16E-05	-1.30	1.43E-02	ADU
ARHGAP25	11.442	0.238	11.645	0.179	9.40E-05	-1.15	1.43E-02	ADU
TMEM107	5.814	0.194	6.005	0.212	8.11E-05	-1.14	1.43E-02	ADU
ATE1	6.457	0.343	6.75	0.251	8.60E-05	-1.23	1.43E-02	ADU
ASGR2	5.715	0.186	5.56	0.125	9.58E-05	1.11	1.44E-02	ADU
IDH3G	8.416	0.192	8.603	0.205	8.86E-05	-1.14	1.44E-02	ADU
PIGS	7.781	0.22	7.974	0.172	7.15E-05	-1.14	1.44E-02	ADU
FAM63B	4.218	0.241	4.462	0.28	9.03E-05	-1.18	1.44E-02	ADU
ATPAF1	5.212	0.245	5.429	0.209	9.53E-05	-1.16	1.44E-02	ADU
SPRYD3	7.306	0.182	7.476	0.18	9.30E-05	-1.13	1.44E-02	ADU
GOLGA5	7.591	0.361	7.888	0.21	7.03E-05	-1.23	1.45E-02	ADU
MORF4L1	7.815	0.273	8.045	0.191	8.58E-05	-1.17	1.45E-02	ADU
MCU	7.009	0.229	7.221	0.221	9.40E-05	-1.16	1.45E-02	ADU
VPS53	7.287	0.264	7.539	0.26	7.12E-05	-1.19	1.45E-02	ADU
BEND4	4.912	0.122	4.8	0.108	6.97E-05	1.08	1.45E-02	ADU
MIER2	5.495	0.119	5.38	0.125	9.78E-05	1.08	1.45E-02	ADU
EIF2C2	8.912	0.275	9.173	0.281	1.00E-04	-1.20	1.48E-02	ADU
NUP85	6.195	0.289	6.468	0.293	1.03E-04	-1.21	1.49E-02	ADU
DYNC1LI2	6.456	0.175	6.611	0.151	1.03E-04	-1.11	1.50E-02	ADU
BET3L	3.691	0.184	3.529	0.158	1.06E-04	1.12	1.51E-02	ADU
PSMD2	6.919	0.274	7.146	0.189	1.05E-04	-1.17	1.51E-02	ADU
POLR2G	6.347	0.228	6.542	0.176	1.09E-04	-1.14	1.53E-02	ADU
MYO18A	6.646	0.21	6.873	0.279	1.09E-04	-1.17	1.54E-02	ADU
RBMS1	8.798	0.161	8.647	0.164	1.12E-04	1.11	1.56E-02	ADU
CYP26B1	5.555	0.306	5.303	0.213	1.13E-04	1.19	1.56E-02	ADU
GLIS2	6.5	0.183	6.338	0.162	1.21E-04	1.12	1.57E-02	ADU
KRTAP10-8	6.897	0.142	6.767	0.136	1.16E-04	1.09	1.57E-02	ADU
LRRC59	7.356	0.179	7.515	0.16	1.15E-04	-1.12	1.58E-02	ADU
PRKAR1A	10.011	0.256	9.781	0.24	1.21E-04	1.17	1.58E-02	ADU
TRIP4	6.218	0.187	6.391	0.186	1.17E-04	-1.13	1.58E-02	ADU
SERTAD1	6.93	0.277	7.179	0.257	1.19E-04	-1.19	1.58E-02	ADU
ZNF490	5.905	0.294	6.152	0.222	1.20E-04	-1.19	1.59E-02	ADU
RALGPS1	5.819	0.15	5.954	0.139	1.19E-04	-1.10	1.59E-02	ADU
INPP5B	6.783	0.306	7.078	0.34	1.27E-04	-1.23	1.63E-02	ADU

ARL2BP	6.294	0.351	6.589	0.271	1.28E-04	-1.23	1.64E-02	ADU
SCPEP1	7.988	0.399	8.391	0.483	1.29E-04	-1.32	1.64E-02	ADU
TTC9C	6.108	0.356	6.405	0.27	1.31E-04	-1.23	1.65E-02	ADU
CLN3	7.661	0.202	7.846	0.202	1.33E-04	-1.14	1.66E-02	ADU
ARIH2	6.597	0.43	6.945	0.29	1.35E-04	-1.27	1.67E-02	ADU
ARMC10	5.499	0.311	5.77	0.27	1.34E-04	-1.21	1.67E-02	ADU
SPCS1	6.639	0.183	6.809	0.19	1.36E-04	-1.13	1.67E-02	ADU
RACGAP1	4.605	0.129	4.752	0.191	1.39E-04	-1.11	1.68E-02	ADU
PARL	6.638	0.27	6.887	0.275	1.38E-04	-1.19	1.68E-02	ADU
GLB1	7.194	0.218	7.385	0.193	1.41E-04	-1.14	1.69E-02	ADU
HCST	7.613	0.311	7.262	0.457	1.41E-04	1.28	1.69E-02	ADU
ALKBH6	6.643	0.236	6.845	0.197	1.44E-04	-1.15	1.71E-02	ADU
ZSCAN29	5.942	0.356	6.323	0.484	1.46E-04	-1.30	1.71E-02	ADU
PREB	6.362	0.166	6.507	0.149	1.46E-04	-1.11	1.72E-02	ADU
MYLK2	4.977	0.141	4.855	0.125	1.48E-04	1.09	1.73E-02	ADU
PSMA4	7.361	0.378	7.688	0.33	1.50E-04	-1.25	1.73E-02	ADU
DDX23	7.937	0.239	8.124	0.14	1.52E-04	-1.14	1.73E-02	ADU
ADAT1	6.631	0.299	6.89	0.262	1.51E-04	-1.20	1.74E-02	ADU
ZNF703	6.649	0.131	6.535	0.117	1.57E-04	1.08	1.76E-02	ADU
CD300LB	6.887	0.398	7.249	0.399	1.57E-04	-1.29	1.76E-02	ADU
RUFY1	7.38	0.209	7.557	0.174	1.56E-04	-1.13	1.77E-02	ADU
DERL3	6.143	0.102	6.051	0.103	1.69E-04	1.07	1.80E-02	ADU
DHFRL1	4.628	0.126	4.739	0.12	1.71E-04	-1.08	1.81E-02	ADU
LAMTOR1	8.516	0.169	8.667	0.163	1.69E-04	-1.11	1.81E-02	ADU
ZHX1	5.037	0.241	5.251	0.228	1.64E-04	-1.16	1.82E-02	ADU
HSH2D	9.139	0.315	9.408	0.274	1.69E-04	-1.20	1.82E-02	ADU
DNAJC14	6.907	0.266	7.114	0.157	1.68E-04	-1.15	1.82E-02	ADU
PLEKHJ1	7.16	0.173	7.299	0.121	1.75E-04	-1.10	1.83E-02	ADU
NRG1	5.438	0.158	5.307	0.124	1.68E-04	1.10	1.83E-02	ADU
IL18BP	6.865	0.237	7.098	0.284	1.74E-04	-1.18	1.83E-02	ADU
EBP	5.994	0.295	6.254	0.282	1.77E-04	-1.20	1.84E-02	ADU
CCDC53	6.555	0.433	6.897	0.282	1.68E-04	-1.27	1.84E-02	ADU
RAB26	6.163	0.123	6.05	0.128	1.68E-04	1.08	1.85E-02	ADU
CCT5	6.897	0.4	7.208	0.252	1.91E-04	-1.24	1.86E-02	ADU
ECHDC3	6.802	0.359	6.42	0.5	1.90E-04	1.30	1.86E-02	ADU
TRAF6	8.179	0.286	8.393	0.134	1.81E-04	-1.16	1.86E-02	ADU
RBM4	7.592	0.212	7.771	0.183	1.95E-04	-1.13	1.86E-02	ADU
ZNF18	6.647	0.193	6.816	0.183	1.94E-04	-1.12	1.86E-02	ADU
PHF5A	6.118	0.216	6.307	0.202	1.90E-04	-1.14	1.86E-02	ADU
HEBP2	9.874	0.146	9.695	0.248	1.87E-04	1.13	1.86E-02	ADU
ARL6IP4	7.62	0.104	7.735	0.153	1.80E-04	-1.08	1.86E-02	ADU
CHCHD4	4.777	0.195	4.947	0.183	1.89E-04	-1.13	1.87E-02	ADU
C16orf82	5.184	0.144	5.062	0.125	1.93E-04	1.09	1.87E-02	ADU
SIPA1L1	9.901	0.207	9.696	0.254	1.86E-04	1.15	1.87E-02	ADU
ACADVL	7.689	0.221	7.908	0.272	1.88E-04	-1.16	1.87E-02	ADU
POP4	5.694	0.187	5.865	0.195	1.83E-04	-1.13	1.87E-02	ADU
KAT8	7.441	0.167	7.601	0.191	1.86E-04	-1.12	1.87E-02	ADU

EMC6	5.273	0.282	5.51	0.239	1.85E-04	-1.18	1.88E-02	ADU
CORO1B	6.876	0.147	7.021	0.181	1.99E-04	-1.11	1.89E-02	ADU
RGS2	12.092	0.144	11.969	0.131	2.02E-04	1.09	1.91E-02	ADU
VEGFC	5.205	0.108	5.101	0.126	2.07E-04	1.07	1.95E-02	ADU
MTMR1	7.727	0.32	7.984	0.237	2.12E-04	-1.19	1.97E-02	ADU
BECN1	7.953	0.272	8.169	0.197	2.14E-04	-1.16	1.97E-02	ADU
STAMBP	6.527	0.19	6.692	0.181	2.13E-04	-1.12	1.97E-02	ADU
ZNF180	5.717	0.213	5.894	0.178	2.11E-04	-1.13	1.98E-02	ADU
CHMP2A	11.08	0.162	11.215	0.139	2.19E-04	-1.10	2.01E-02	ADU
SAP30BP	7.273	0.199	7.433	0.154	2.25E-04	-1.12	2.05E-02	ADU
PFKM	4.833	0.2	5.083	0.358	2.26E-04	-1.19	2.05E-02	ADU
SSU72	7.657	0.205	7.824	0.168	2.31E-04	-1.12	2.08E-02	ADU
ANO8	5.866	0.125	5.763	0.102	2.32E-04	1.07	2.08E-02	ADU
SPPL2A	8.427	0.345	8.717	0.306	2.34E-04	-1.22	2.08E-02	ADU
B4GALT1	7.915	0.176	7.763	0.168	2.34E-04	1.11	2.09E-02	ADU
SKI	8.015	0.19	7.841	0.207	2.37E-04	1.13	2.10E-02	ADU
PIK3R5	9.441	0.16	9.29	0.186	2.46E-04	1.11	2.15E-02	ADU
NDUFA11	5.959	0.118	5.835	0.164	2.45E-04	1.09	2.15E-02	ADU
PPIB	9.608	0.177	9.753	0.148	2.44E-04	-1.11	2.15E-02	ADU
LNX2	5.835	0.304	6.103	0.308	2.48E-04	-1.20	2.15E-02	ADU
SNRPD3	6.546	0.294	6.797	0.278	2.52E-04	-1.19	2.15E-02	ADU
DNAJB4	3.136	0.253	3.367	0.277	2.52E-04	-1.17	2.16E-02	ADU
TSPAN31	6.392	0.301	6.645	0.271	2.51E-04	-1.19	2.16E-02	ADU
FREM1	3.075	0.149	2.948	0.138	2.60E-04	1.09	2.21E-02	ADU
CDC40	7.083	0.291	7.298	0.159	2.62E-04	-1.16	2.21E-02	ADU
ECH1	7.806	0.4	8.169	0.435	2.65E-04	-1.29	2.23E-02	ADU
VPS33B	5.4	0.232	5.592	0.204	2.66E-04	-1.14	2.23E-02	ADU
PPP2R1B	5.766	0.355	6.048	0.272	2.69E-04	-1.22	2.24E-02	ADU
MAP3K11	8.748	0.169	8.611	0.137	2.70E-04	1.10	2.24E-02	ADU
DRAP1	8.788	0.484	8.367	0.482	2.75E-04	1.34	2.27E-02	ADU
TMEM38B	4.13	0.2	4.322	0.245	2.78E-04	-1.14	2.28E-02	ADU
CCNJL	9.206	0.24	8.972	0.301	2.82E-04	1.18	2.30E-02	ADU
ZNF557	5.72	0.239	5.916	0.204	2.82E-04	-1.15	2.30E-02	ADU
NOL12	7.563	0.181	7.709	0.15	2.88E-04	-1.11	2.34E-02	ADU
MON2	7.271	0.279	7.479	0.167	2.91E-04	-1.16	2.35E-02	ADU
GIPC3	5.686	0.191	5.538	0.138	2.94E-04	1.11	2.36E-02	ADU
FAM100A	8.594	0.18	8.446	0.158	3.00E-04	1.11	2.40E-02	ADU
ZNF143	6.402	0.268	6.613	0.205	3.02E-04	-1.16	2.41E-02	ADU
PRKAR2B	5.413	0.716	4.839	0.59	3.11E-04	1.49	2.46E-02	ADU
LCAT	7.407	0.187	7.565	0.177	3.12E-04	-1.12	2.47E-02	ADU
POLR2E	7.45	0.203	7.633	0.225	3.23E-04	-1.14	2.53E-02	ADU
PPP2R4	5.584	0.205	5.752	0.183	3.23E-04	-1.12	2.53E-02	ADU
S100Z	5.857	0.415	6.213	0.412	3.27E-04	-1.28	2.54E-02	ADU
ACAA1	7.383	0.221	7.565	0.199	3.28E-04	-1.13	2.55E-02	ADU
PRMT2	6.41	0.243	6.616	0.236	3.41E-04	-1.15	2.62E-02	ADU
GAB3	8.201	0.277	8.427	0.245	3.40E-04	-1.17	2.63E-02	ADU
TBCC	5.982	0.201	6.135	0.143	3.63E-04	-1.11	2.66E-02	ADU

TMEM14B	6.672	0.418	7.003	0.344	3.62E-04	-1.26	2.67E-02	ADU
ZBTB43	5.023	0.156	5.162	0.173	3.62E-04	-1.10	2.68E-02	ADU
FADS3	6.291	0.131	6.173	0.149	3.67E-04	1.09	2.68E-02	ADU
PSMA6	7.56	0.367	7.838	0.254	3.51E-04	-1.21	2.68E-02	ADU
DYSF	11.34	0.258	11.057	0.406	3.62E-04	1.22	2.69E-02	ADU
VPS39	8.192	0.233	8.361	0.131	3.58E-04	-1.12	2.69E-02	ADU
TMEM14C	6.371	0.441	6.78	0.525	3.61E-04	-1.33	2.70E-02	ADU
WDR37	7.34	0.184	7.492	0.173	3.57E-04	-1.11	2.70E-02	ADU
MAGED4B	5.733	0.114	5.641	0.101	3.60E-04	1.07	2.70E-02	ADU
NDUFAF1	4.578	0.46	4.918	0.289	3.55E-04	-1.27	2.70E-02	ADU
MTMR9	5.182	0.224	5.367	0.21	3.72E-04	-1.14	2.70E-02	ADU
ATP8B4	5.853	0.393	6.176	0.358	3.56E-04	-1.25	2.70E-02	ADU
WIZ	6.465	0.112	6.371	0.108	3.81E-04	1.07	2.71E-02	ADU
SLC30A5	7.837	0.323	8.098	0.286	3.74E-04	-1.20	2.71E-02	ADU
MIS12	5.1	0.33	5.364	0.282	3.83E-04	-1.20	2.71E-02	ADU
COPS7A	7.047	0.158	7.173	0.133	3.81E-04	-1.09	2.71E-02	ADU
TMED6	3.998	0.187	4.153	0.178	3.87E-04	-1.11	2.72E-02	ADU
UNC5B	5.972	0.142	5.859	0.117	3.80E-04	1.08	2.72E-02	ADU
CCDC159	6.793	0.245	7.018	0.29	3.89E-04	-1.17	2.73E-02	ADU
SSX5	3.636	0.216	3.454	0.214	3.78E-04	1.13	2.73E-02	ADU
TMEM50B	5.078	0.342	5.368	0.343	3.80E-04	-1.22	2.73E-02	ADU
TERF2	7.102	0.165	7.233	0.138	3.94E-04	-1.10	2.73E-02	ADU
CIRBP	8.847	0.287	9.067	0.216	3.93E-04	-1.16	2.74E-02	ADU
GPRC5B	4.598	0.212	4.437	0.158	3.97E-04	1.12	2.74E-02	ADU
TMEM81	6.108	0.236	6.324	0.28	3.92E-04	-1.16	2.74E-02	ADU
XCL2	2.593	0.349	2.911	0.409	4.10E-04	-1.25	2.77E-02	ADU
MLKL	8.357	0.466	8.7	0.302	4.06E-04	-1.27	2.77E-02	ADU
MVD	6.379	0.145	6.267	0.113	4.10E-04	1.08	2.78E-02	ADU
ENTPD3	5.159	0.394	4.785	0.497	4.05E-04	1.30	2.78E-02	ADU
JMJD7	6.808	0.239	7.02	0.267	4.04E-04	-1.16	2.78E-02	ADU
TM2D2	4.892	0.355	5.163	0.27	4.14E-04	-1.21	2.78E-02	ADU
DPF1	5.673	0.16	5.553	0.113	4.10E-04	1.09	2.79E-02	ADU
UQCRC2	8.091	0.365	8.351	0.2	4.20E-04	-1.20	2.79E-02	ADU
ZNF140	4.894	0.314	5.138	0.252	4.17E-04	-1.18	2.79E-02	ADU
PPM1D	8.198	0.242	8.384	0.19	4.18E-04	-1.14	2.79E-02	ADU
MIEN1	8.73	0.194	8.946	0.317	4.24E-04	-1.16	2.81E-02	ADU
STT3A	6.979	0.351	7.265	0.326	4.29E-04	-1.22	2.83E-02	ADU
HAX1	5.896	0.371	6.17	0.251	4.35E-04	-1.21	2.86E-02	ADU
RUNDC3A	5.656	0.137	5.543	0.132	4.39E-04	1.08	2.87E-02	ADU
MDP1	5.392	0.219	5.57	0.202	4.38E-04	-1.13	2.87E-02	ADU
ARHGAP24	5.629	0.23	5.409	0.3	4.44E-04	1.16	2.89E-02	ADU
HMGCL	5.807	0.186	5.958	0.173	4.49E-04	-1.11	2.91E-02	ADU
UBE2Z	7.269	0.228	7.457	0.221	4.63E-04	-1.14	2.97E-02	ADU
BFAR	5.813	0.23	6.003	0.223	4.60E-04	-1.14	2.97E-02	ADU
SRF	8.212	0.115	8.104	0.148	4.62E-04	1.08	2.97E-02	ADU
RNF34	7.255	0.217	7.417	0.159	4.69E-04	-1.12	2.99E-02	ADU
HDAC6	6.437	0.194	6.603	0.205	4.72E-04	-1.12	3.00E-02	ADU

PSTPIP1	8.352	0.181	8.496	0.161	4.75E-04	-1.10	3.01E-02	ADU
NFIC	7.48	0.143	7.338	0.201	4.86E-04	1.10	3.06E-02	ADU
CD300C	7.298	0.232	7.515	0.294	4.85E-04	-1.16	3.06E-02	ADU
RIPK4	5.052	0.225	4.878	0.185	4.95E-04	1.13	3.07E-02	ADU
ME3	5.302	0.124	5.205	0.104	4.91E-04	1.07	3.08E-02	ADU
DHRS1	6.123	0.267	6.338	0.246	4.93E-04	-1.16	3.08E-02	ADU
TTC30B	3.903	0.285	4.139	0.284	4.95E-04	-1.18	3.08E-02	ADU
MYL9	7.034	0.302	6.799	0.255	5.01E-04	1.18	3.10E-02	ADU
PTPN9	5.325	0.251	5.56	0.32	5.08E-04	-1.18	3.13E-02	ADU
SLC9A5	5.343	0.138	5.236	0.116	5.16E-04	1.08	3.17E-02	ADU
BTK	7.409	0.446	7.77	0.424	5.21E-04	-1.28	3.18E-02	ADU
NME6	5.786	0.331	6.031	0.24	5.32E-04	-1.19	3.18E-02	ADU
SHROOM2	4.967	0.147	4.854	0.121	5.38E-04	1.08	3.18E-02	ADU
APEX2	5.843	0.149	5.975	0.173	5.27E-04	-1.10	3.18E-02	ADU
LACTB2	3.432	0.262	3.652	0.271	5.20E-04	-1.16	3.18E-02	ADU
AMMECR1L	5.741	0.209	5.904	0.179	5.34E-04	-1.12	3.18E-02	ADU
SNPH	6.496	0.141	6.373	0.159	5.29E-04	1.09	3.18E-02	ADU
FBXO8	6.669	0.224	6.849	0.209	5.31E-04	-1.13	3.18E-02	ADU
FBXO34	6.011	0.19	6.166	0.182	5.26E-04	-1.11	3.18E-02	ADU
GPKOW	6.63	0.154	6.759	0.157	5.24E-04	-1.09	3.19E-02	ADU
COX15	6.478	0.231	6.648	0.167	5.41E-04	-1.13	3.19E-02	ADU
VRK3	8.347	0.225	8.515	0.169	5.37E-04	-1.12	3.19E-02	ADU
FAM175B	6.459	0.327	6.696	0.222	5.49E-04	-1.18	3.22E-02	ADU
RBM14	7.48	0.283	7.72	0.302	5.58E-04	-1.18	3.27E-02	ADU
GPR144	6.393	0.151	6.282	0.11	5.63E-04	1.08	3.29E-02	ADU
STRN3	5.805	0.196	5.953	0.155	5.66E-04	-1.11	3.29E-02	ADU
TMEM150B	5.849	0.194	5.702	0.159	5.81E-04	1.11	3.36E-02	ADU
AIG1	6.009	0.322	6.296	0.384	5.80E-04	-1.22	3.36E-02	ADU
GSDMB	6.001	0.475	6.428	0.575	5.88E-04	-1.34	3.39E-02	ADU
HMOX2	7.225	0.165	7.367	0.186	5.93E-04	-1.10	3.39E-02	ADU
LRRC6	7.679	0.531	7.118	0.837	5.95E-04	1.48	3.39E-02	ADU
MED27	4.996	0.329	5.258	0.307	5.92E-04	-1.20	3.40E-02	ADU
PLXNA1	6.013	0.112	5.919	0.119	6.04E-04	1.07	3.41E-02	ADU
CARM1	6.282	0.195	6.112	0.221	6.00E-04	1.13	3.41E-02	ADU
GDF3	3.308	0.276	3.104	0.206	6.03E-04	1.15	3.42E-02	ADU
GAB4	5.898	0.158	5.779	0.127	6.13E-04	1.09	3.43E-02	ADU
ACAD10	6.036	0.206	6.185	0.143	6.13E-04	-1.11	3.44E-02	ADU
DPEP2	9.678	0.297	9.897	0.225	6.13E-04	-1.16	3.45E-02	ADU
CMIP	8.619	0.211	8.473	0.118	6.20E-04	1.11	3.45E-02	ADU
FABP5	5.9	0.151	5.782	0.136	6.23E-04	1.09	3.45E-02	ADU
PTER	4.958	0.277	5.195	0.307	6.20E-04	-1.18	3.46E-02	ADU
ACOX3	6.151	0.109	6.247	0.128	6.27E-04	-1.07	3.46E-02	ADU
MAGED2	5.248	0.231	5.441	0.245	6.29E-04	-1.14	3.47E-02	ADU
TONSL	6.311	0.168	6.171	0.178	6.34E-04	1.10	3.48E-02	ADU
CXXC1	6.328	0.147	6.451	0.158	6.48E-04	-1.09	3.50E-02	ADU
KIF26A	6.395	0.138	6.291	0.112	6.44E-04	1.07	3.51E-02	ADU
PLEKHN1	6.394	0.149	6.291	0.089	6.52E-04	1.07	3.51E-02	ADU

TSPAN10	5.823	0.218	5.644	0.224	6.47E-04	1.13	3.51E-02	ADU
ZNF616	4.221	0.361	4.486	0.274	6.52E-04	-1.20	3.51E-02	ADU
GPR173	5.179	0.162	5.04	0.181	6.44E-04	1.10	3.51E-02	ADU
LRP10	9.945	0.212	9.761	0.245	6.58E-04	1.14	3.52E-02	ADU
RAP2C	6.61	0.101	6.519	0.123	6.43E-04	1.07	3.52E-02	ADU
CHMP6	6.456	0.173	6.606	0.198	6.57E-04	-1.11	3.52E-02	ADU
MAP6	4.847	0.146	4.724	0.159	6.64E-04	1.09	3.54E-02	ADU
PRRT3	6.334	0.14	6.222	0.137	6.75E-04	1.08	3.58E-02	ADU
NLRP6	8.155	0.33	7.9	0.296	6.74E-04	1.19	3.58E-02	ADU
ZNF234	4.839	0.297	5.081	0.303	6.88E-04	-1.18	3.63E-02	ADU
PABPC1L	7.495	0.175	7.34	0.211	6.93E-04	1.11	3.64E-02	ADU
CDK2	5.665	0.325	5.924	0.316	6.92E-04	-1.20	3.64E-02	ADU
QPCT	8.28	0.298	8.013	0.37	6.99E-04	1.20	3.66E-02	ADU
ASNA1	7.432	0.204	7.281	0.163	7.08E-04	1.11	3.68E-02	ADU
FAM169B	4.393	0.189	4.25	0.161	7.08E-04	1.10	3.69E-02	ADU
PPM1J	5.537	0.146	5.43	0.116	7.14E-04	1.08	3.70E-02	ADU
ATP5D	7.294	0.141	7.424	0.186	7.29E-04	-1.09	3.73E-02	ADU
FAM82B	5.007	0.337	5.272	0.319	7.21E-04	-1.20	3.73E-02	ADU
C3orf58	5.256	0.577	5.681	0.454	7.28E-04	-1.34	3.73E-02	ADU
SUMO1	5.978	0.269	5.769	0.246	7.26E-04	1.16	3.74E-02	ADU
STRN4	8.348	0.168	8.179	0.253	7.28E-04	1.12	3.74E-02	ADU
GATAD1	5.831	0.174	5.968	0.165	7.39E-04	-1.10	3.76E-02	ADU
ZNF615	4.902	0.249	5.096	0.231	7.37E-04	-1.14	3.76E-02	ADU
ANKRD6	4.92	0.243	5.131	0.285	7.43E-04	-1.16	3.77E-02	ADU
IGIP	2.806	0.207	2.984	0.241	7.54E-04	-1.13	3.81E-02	ADU
TBL2	5.976	0.152	6.115	0.2	7.58E-04	-1.10	3.82E-02	ADU
LRSAM1	5.848	0.201	6.014	0.216	7.60E-04	-1.12	3.82E-02	ADU
SH3BGRL	7.863	0.279	8.088	0.287	7.78E-04	-1.17	3.90E-02	ADU
AGGF1	6.154	0.29	6.355	0.188	7.84E-04	-1.15	3.90E-02	ADU
CYB5B	5.663	0.293	5.905	0.316	7.83E-04	-1.18	3.90E-02	ADU
IL8	9.371	0.684	8.814	0.715	7.82E-04	1.47	3.91E-02	ADU
TRADD	7.525	0.165	7.655	0.161	7.93E-04	-1.09	3.93E-02	ADU
TMEM65	6.625	0.224	6.788	0.174	7.98E-04	-1.12	3.94E-02	ADU
ZFPL1	7.89	0.134	7.999	0.14	8.00E-04	-1.08	3.94E-02	ADU
FEM1B	7.528	0.178	7.657	0.14	8.10E-04	-1.09	3.97E-02	ADU
TRIM52	6.594	0.284	6.804	0.231	8.09E-04	-1.16	3.97E-02	ADU
PLK1S1	5.911	0.283	6.121	0.238	8.18E-04	-1.16	3.99E-02	ADU
DOK2	6.868	0.442	7.286	0.616	8.31E-04	-1.34	4.05E-02	ADU
RHBDL1	6.164	0.128	6.068	0.108	8.36E-04	1.07	4.06E-02	ADU
SETD1B	8.545	0.15	8.43	0.139	8.40E-04	1.08	4.07E-02	ADU
MOCS3	4.882	0.297	5.11	0.273	8.49E-04	-1.17	4.08E-02	ADU
RPP25	4.829	0.187	4.698	0.127	8.45E-04	1.10	4.08E-02	ADU
SAMD4B	7.461	0.206	7.317	0.138	8.49E-04	1.10	4.09E-02	ADU
COG4	6.525	0.358	6.772	0.229	8.57E-04	-1.19	4.11E-02	ADU
ANXA1	9.859	0.518	10.287	0.569	8.64E-04	-1.35	4.12E-02	ADU
SHC2	5.685	0.148	5.576	0.119	8.62E-04	1.08	4.12E-02	ADU
RTEL1	6.093	0.113	6.014	0.077	8.74E-04	1.06	4.13E-02	ADU

XPNPEP2	4.966	0.135	4.858	0.14	8.77E-04	1.08	4.14E-02	ADU
PARVB	5.643	0.237	5.461	0.22	8.73E-04	1.13	4.14E-02	ADU
TEFM	4.188	0.299	4.44	0.342	8.71E-04	-1.19	4.14E-02	ADU
FTSJ1	5.882	0.205	6.065	0.26	8.87E-04	-1.14	4.16E-02	ADU
SLC22A17	6.501	0.112	6.413	0.11	8.84E-04	1.06	4.16E-02	ADU
EMX2	4.951	0.125	4.848	0.138	9.00E-04	1.07	4.21E-02	ADU
TLN1	10.143	0.148	10.036	0.119	9.14E-04	1.08	4.24E-02	ADU
IQSEC1	9.585	0.127	9.472	0.162	9.10E-04	1.08	4.25E-02	ADU
DHX33	4.885	0.163	5.014	0.163	9.13E-04	-1.09	4.25E-02	ADU
CD9	6.556	0.596	7.125	0.854	9.22E-04	-1.48	4.27E-02	ADU
FOSL1	6.216	0.191	6.076	0.16	9.24E-04	1.10	4.27E-02	ADU
EED	6.249	0.288	6.459	0.233	9.27E-04	-1.16	4.27E-02	ADU
ZNF673	6.182	0.205	6.33	0.165	9.68E-04	-1.11	4.44E-02	ADU
PHF20L1	8.757	0.341	8.511	0.273	9.66E-04	1.19	4.44E-02	ADU
CAPNS1	8.462	0.267	8.67	0.265	9.79E-04	-1.16	4.47E-02	ADU
KIAA1967	7.41	0.364	7.676	0.306	9.85E-04	-1.20	4.48E-02	ADU
PDIA6	6.165	0.254	6.358	0.239	9.83E-04	-1.14	4.48E-02	ADU
ORM1	7.048	0.518	6.598	0.642	9.94E-04	1.37	4.51E-02	ADU
ZNF467	8.112	0.196	7.949	0.224	1.01E-03	1.12	4.51E-02	ADU
DIP2A	6.93	0.204	7.131	0.311	1.00E-03	-1.15	4.52E-02	ADU
ASNSD1	5.591	0.32	5.303	0.423	1.01E-03	1.22	4.52E-02	ADU
FAM125A	6.246	0.164	6.375	0.165	1.00E-03	-1.09	4.52E-02	ADU
TRMT12	4.915	0.521	5.308	0.479	1.02E-03	-1.31	4.55E-02	ADU
FAM149B1	5.017	0.227	5.182	0.192	1.03E-03	-1.12	4.59E-02	ADU
ALKBH7	5.013	0.157	4.899	0.131	1.03E-03	1.08	4.59E-02	ADU
ZNF512B	5.617	0.133	5.517	0.123	1.04E-03	1.07	4.62E-02	ADU
FOXI1	5.615	0.177	5.476	0.181	1.06E-03	1.10	4.65E-02	ADU
TP53I11	7.36	0.199	7.167	0.3	1.06E-03	1.14	4.66E-02	ADU
PPP1R18	9.082	0.134	8.964	0.173	1.06E-03	1.09	4.66E-02	ADU
RTCA	6.863	0.303	7.07	0.204	1.06E-03	-1.15	4.66E-02	ADU
SFI1	7.248	0.198	7.407	0.214	1.07E-03	-1.12	4.67E-02	ADU
JPH3	5.023	0.203	4.887	0.127	1.06E-03	1.10	4.68E-02	ADU
PNMA6A	6.877	0.214	6.72	0.186	1.09E-03	1.11	4.68E-02	ADU
SLCO2B1	4.408	0.142	4.308	0.112	1.10E-03	1.07	4.69E-02	ADU
PTTG1IP	8.238	0.188	8.389	0.203	1.08E-03	-1.11	4.69E-02	ADU
PRPF6	6.755	0.204	6.899	0.159	1.07E-03	-1.10	4.69E-02	ADU
SNRNP25	6.538	0.294	6.81	0.41	1.09E-03	-1.21	4.69E-02	ADU
TCEB2	7.222	0.211	7.025	0.3	1.09E-03	1.15	4.69E-02	ADU
FICD	5.604	0.252	5.791	0.227	1.10E-03	-1.14	4.69E-02	ADU
TRPC4AP	9.399	0.154	9.502	0.096	1.08E-03	-1.07	4.70E-02	ADU
CENPQ	2.945	0.21	3.11	0.219	1.09E-03	-1.12	4.70E-02	ADU
JMJD7-PLA2G4B	5.977	0.278	6.21	0.329	1.08E-03	-1.18	4.70E-02	ADU
GOLT1B	4.182	0.42	4.468	0.287	1.11E-03	-1.22	4.71E-02	ADU
TMEM57	6.632	0.34	6.887	0.317	1.12E-03	-1.19	4.74E-02	ADU
DESI2	6.017	0.26	6.206	0.222	1.12E-03	-1.14	4.74E-02	ADU
STX4	7.75	0.236	7.909	0.155	1.12E-03	-1.12	4.75E-02	ADU
NLRP1	9.784	0.194	9.599	0.285	1.13E-03	1.14	4.75E-02	ADU

GOLGA7	8.694	0.19	8.547	0.191	1.13E-03	1.11	4.76E-02	ADU
ZNF434	5.018	0.167	5.139	0.141	1.13E-03	-1.09	4.76E-02	ADU
TBC1D10A	7.413	0.165	7.57	0.241	1.14E-03	-1.11	4.78E-02	ADU
NEB	4.888	0.316	4.667	0.239	1.15E-03	1.17	4.79E-02	ADU
CDKN2AIP	6.766	0.374	7.017	0.242	1.15E-03	-1.19	4.80E-02	ADU
BCAS3	8.471	0.214	8.615	0.141	1.16E-03	-1.10	4.82E-02	ADU
TTC38	6.128	0.242	6.338	0.309	1.16E-03	-1.16	4.83E-02	ADU
EXOC7	7.395	0.222	7.555	0.186	1.17E-03	-1.12	4.83E-02	ADU
BTF3L4	6.98	0.296	7.181	0.204	1.18E-03	-1.15	4.86E-02	ADU
NAPG	6.088	0.277	6.29	0.242	1.18E-03	-1.15	4.87E-02	ADU
ASTE1	5.047	0.281	5.248	0.232	1.20E-03	-1.15	4.91E-02	ADU
PHC2	8.089	0.146	7.972	0.16	1.20E-03	1.08	4.92E-02	ADU
CREB3	6.437	0.27	6.619	0.184	1.21E-03	-1.13	4.93E-02	ADU
ZNF436	4.828	0.149	4.935	0.124	1.23E-03	-1.08	4.96E-02	ADU
NUP37	4.026	0.246	4.218	0.254	1.22E-03	-1.14	4.96E-02	ADU
TANC2	7.303	0.36	7.043	0.311	1.23E-03	1.20	4.97E-02	ADU
OBSL1	5.552	0.137	5.451	0.127	1.22E-03	1.07	4.97E-02	ADU
SOGA3	4.01	0.134	3.909	0.13	1.22E-03	1.07	4.97E-02	ADU
KDM4E	4.359	0.166	4.232	0.165	1.23E-03	1.09	4.98E-02	ADU
SOX4	5.717	0.127	5.621	0.122	1.22E-03	1.07	4.98E-02	ADU
SNRPD3	6.351	0.174	6.797	0.278	1.66E-11	-1.36	2.94E-07	ADT
OS9	9.471	0.157	9.812	0.203	1.34E-10	-1.27	7.92E-07	ADT
MRPL49	6.661	0.163	7.121	0.329	1.10E-10	-1.38	9.76E-07	ADT
PRCC	7.834	0.105	8.048	0.138	1.08E-09	-1.16	4.77E-06	ADT
CAPNS1	8.294	0.18	8.67	0.265	2.27E-09	-1.30	8.01E-06	ADT
ADRM1	7.503	0.126	7.729	0.124	3.29E-09	-1.17	9.68E-06	ADT
FUT11	6.953	0.165	7.262	0.223	1.42E-08	-1.24	1.79E-05	ADT
TRMT112	8.814	0.221	9.213	0.259	9.15E-09	-1.32	1.80E-05	ADT
TCF15	7.393	0.093	7.205	0.14	8.59E-09	1.14	1.90E-05	ADT
BRD4	8.122	0.117	7.929	0.1	1.63E-08	1.14	1.92E-05	ADT
CHMP2A	10.992	0.129	11.215	0.139	1.42E-08	-1.17	1.93E-05	ADT
FABP5	6.005	0.125	5.782	0.136	7.73E-09	1.17	1.95E-05	ADT
ADCK4	6.57	0.119	6.783	0.14	1.17E-08	-1.16	2.07E-05	ADT
CREBBP	10.094	0.119	9.899	0.097	1.41E-08	1.14	2.08E-05	ADT
FKBP1A	9.145	0.144	8.88	0.184	1.31E-08	1.20	2.11E-05	ADT
VPS72	7.922	0.184	8.227	0.182	2.22E-08	-1.24	2.45E-05	ADT
DSCR3	8.065	0.25	8.46	0.192	3.12E-08	-1.31	3.07E-05	ADT
SAMD4B	7.519	0.117	7.317	0.138	3.03E-08	1.15	3.15E-05	ADT
RNF5	5.841	0.286	6.306	0.281	3.60E-08	-1.38	3.35E-05	ADT
CERS5	7.778	0.212	8.111	0.171	3.86E-08	-1.26	3.41E-05	ADT
KIF7	6.317	0.097	6.147	0.124	4.11E-08	1.13	3.46E-05	ADT
FSCN2	6.276	0.159	6.025	0.142	4.74E-08	1.19	3.65E-05	ADT
MAGEA6	5.598	0.242	5.151	0.352	4.65E-08	1.36	3.74E-05	ADT
CLN3	7.604	0.124	7.846	0.202	5.38E-08	-1.18	3.97E-05	ADT
MMP17	6.119	0.122	5.918	0.137	6.13E-08	1.15	4.01E-05	ADT
TRAPPC4	6.454	0.261	6.865	0.241	6.08E-08	-1.33	4.14E-05	ADT
TCTA	6.605	0.123	6.833	0.182	5.91E-08	-1.17	4.18E-05	ADT

MIOX	6.157	0.127	5.953	0.136	7.96E-08	1.15	4.85E-05	ADT
FAM90A1	5.56	0.104	5.39	0.118	7.75E-08	1.13	4.89E-05	ADT
ECD	8.636	0.262	9.054	0.278	8.48E-08	-1.34	5.00E-05	ADT
BECN1	7.825	0.226	8.169	0.197	9.97E-08	-1.27	5.04E-05	ADT
FBXO8	6.582	0.154	6.849	0.209	9.80E-08	-1.20	5.10E-05	ADT
INTS5	6.274	0.11	6.061	0.183	9.65E-08	1.16	5.17E-05	ADT
KIF26A	6.444	0.092	6.291	0.112	9.14E-08	1.11	5.21E-05	ADT
BAZ2A	9.687	0.135	9.475	0.134	9.53E-08	1.16	5.26E-05	ADT
MLX	8.356	0.209	8.669	0.18	1.32E-07	-1.24	5.71E-05	ADT
TAX1BP3	6.513	0.143	6.797	0.253	1.21E-07	-1.22	5.80E-05	ADT
TBCC	5.87	0.179	6.135	0.143	1.28E-07	-1.20	5.81E-05	ADT
MPLKIP	6.203	0.164	6.488	0.229	1.32E-07	-1.22	5.83E-05	ADT
PHLDB3	6.159	0.097	6.004	0.108	1.19E-07	1.11	5.86E-05	ADT
FBXO28	5.321	0.307	5.788	0.286	1.27E-07	-1.38	5.91E-05	ADT
HCN4	5.771	0.106	5.595	0.133	1.50E-07	1.13	6.03E-05	ADT
HADHA	7.445	0.162	7.699	0.174	1.48E-07	-1.19	6.07E-05	ADT
PRRX2	6.769	0.127	6.561	0.155	1.45E-07	1.16	6.12E-05	ADT
UBAP1L	6.475	0.135	6.272	0.125	1.64E-07	1.15	6.17E-05	ADT
STX4	7.681	0.148	7.909	0.155	1.64E-07	-1.17	6.30E-05	ADT
ATPAF1	5.17	0.151	5.429	0.209	1.62E-07	-1.20	6.36E-05	ADT
SOCS4	4.6	0.259	4.988	0.241	1.76E-07	-1.31	6.49E-05	ADT
INSM1	7.375	0.106	7.212	0.114	2.18E-07	1.12	7.86E-05	ADT
C20orf144	7.054	0.112	6.882	0.123	2.48E-07	1.13	8.44E-05	ADT
SCNM1	7.093	0.167	7.384	0.247	2.39E-07	-1.22	8.45E-05	ADT
MAP3K10	6.71	0.09	6.576	0.087	2.48E-07	1.10	8.60E-05	ADT
TSPAN31	6.288	0.225	6.645	0.271	2.58E-07	-1.28	8.60E-05	ADT
TMED4	9.077	0.146	9.31	0.182	2.78E-07	-1.18	9.11E-05	ADT
CREB5	8.596	0.143	8.369	0.174	2.89E-07	1.17	9.28E-05	ADT
SETD1B	8.624	0.128	8.43	0.139	3.00E-07	1.14	9.46E-05	ADT
SPTBN2	5.383	0.095	5.233	0.116	3.41E-07	1.11	1.06E-04	ADT
GNB1	10.592	0.094	10.748	0.132	3.58E-07	-1.11	1.07E-04	ADT
APC2	6.176	0.096	6.022	0.124	3.53E-07	1.11	1.08E-04	ADT
UBE2Q1	7.956	0.175	8.202	0.137	3.83E-07	-1.19	1.11E-04	ADT
CD300LF	9.174	0.206	9.509	0.276	3.78E-07	-1.26	1.11E-04	ADT
RBFA	5.45	0.096	5.31	0.091	4.08E-07	1.10	1.16E-04	ADT
LAMTOR1	8.463	0.13	8.667	0.163	4.37E-07	-1.15	1.23E-04	ADT
PSMD2	6.848	0.209	7.146	0.189	4.73E-07	-1.23	1.31E-04	ADT
PNRC2	9.054	0.22	9.38	0.243	5.25E-07	-1.25	1.41E-04	ADT
ARPC1B	10.257	0.129	10.453	0.149	5.21E-07	-1.15	1.42E-04	ADT
CHMP3	8.721	0.239	9.051	0.199	6.32E-07	-1.26	1.67E-04	ADT
ZNF205	6.045	0.116	5.873	0.133	7.45E-07	1.13	1.91E-04	ADT
DESI2	5.925	0.188	6.206	0.222	7.43E-07	-1.22	1.93E-04	ADT
SYNDIG1	5	0.153	4.764	0.199	8.38E-07	1.18	2.09E-04	ADT
CDKN2AIP	6.719	0.198	7.017	0.242	8.35E-07	-1.23	2.11E-04	ADT
CASP9	7.091	0.154	7.335	0.212	8.85E-07	-1.18	2.14E-04	ADT
COPE	8.45	0.183	8.702	0.165	8.81E-07	-1.19	2.16E-04	ADT
F11R	8.358	0.191	8.636	0.216	9.94E-07	-1.21	2.28E-04	ADT

BARX1	6.379	0.145	6.17	0.158	9.64E-07	1.16	2.30E-04	ADT
NIT1	6.756	0.15	6.987	0.198	9.92E-07	-1.17	2.31E-04	ADT
SAMD1	6.726	0.116	6.547	0.152	9.86E-07	1.13	2.33E-04	ADT
KIF19	5.483	0.074	5.364	0.107	1.04E-06	1.09	2.37E-04	ADT
ACAA1	7.297	0.189	7.565	0.199	1.07E-06	-1.20	2.39E-04	ADT
CDC40	6.976	0.247	7.298	0.159	1.10E-06	-1.25	2.44E-04	ADT
CHCHD2	6.979	0.289	7.361	0.224	1.17E-06	-1.30	2.51E-04	ADT
CMIP	8.666	0.144	8.473	0.118	1.16E-06	1.14	2.54E-04	ADT
UBL3	6.846	0.102	7.009	0.148	1.21E-06	-1.12	2.57E-04	ADT
KAT5	7.402	0.169	7.628	0.142	1.30E-06	-1.17	2.73E-04	ADT
CCT5	6.706	0.388	7.208	0.252	1.33E-06	-1.42	2.76E-04	ADT
CCDC85B	6.672	0.147	6.475	0.127	1.38E-06	1.15	2.83E-04	ADT
S100PBP	6.678	0.25	7.009	0.21	1.46E-06	-1.26	2.94E-04	ADT
MYNN	6.424	0.497	7.112	0.512	1.48E-06	-1.61	2.94E-04	ADT
RUNDC1	5.984	0.185	6.231	0.16	1.50E-06	-1.19	2.95E-04	ADT
RAB11FIP3	6.091	0.111	5.938	0.115	1.54E-06	1.11	2.95E-04	ADT
DAGLB	7.578	0.145	7.8	0.194	1.46E-06	-1.17	2.96E-04	ADT
ATP5B	8.585	0.203	8.863	0.203	1.53E-06	-1.21	2.98E-04	ADT
HAX1	5.797	0.279	6.17	0.251	1.61E-06	-1.30	3.05E-04	ADT
GFAP	5.452	0.093	5.306	0.134	1.67E-06	1.11	3.05E-04	ADT
LRRC4B	6.123	0.104	5.979	0.11	1.63E-06	1.10	3.06E-04	ADT
ARPC1A	7.932	0.143	8.131	0.153	1.67E-06	-1.15	3.08E-04	ADT
ZNF574	6.163	0.102	6.329	0.157	1.66E-06	-1.12	3.09E-04	ADT
ACTR1A	7.449	0.143	7.647	0.153	1.84E-06	-1.15	3.31E-04	ADT
RTEL1	6.129	0.087	6.014	0.077	1.86E-06	1.08	3.33E-04	ADT
NEFH	5.15	0.121	4.981	0.136	1.91E-06	1.12	3.38E-04	ADT
LZIC	5.617	0.298	6.01	0.269	1.96E-06	-1.31	3.43E-04	ADT
HMX3	6.896	0.156	6.677	0.178	2.04E-06	1.16	3.49E-04	ADT
PREB	6.339	0.114	6.507	0.149	2.06E-06	-1.12	3.50E-04	ADT
CDH16	5.311	0.103	5.167	0.117	2.03E-06	1.10	3.52E-04	ADT
VRK3	8.281	0.176	8.515	0.169	2.15E-06	-1.18	3.61E-04	ADT
NEU1	7.823	0.204	8.165	0.339	2.17E-06	-1.27	3.62E-04	ADT
GOLT1B	4.053	0.316	4.468	0.287	2.21E-06	-1.33	3.65E-04	ADT
NDUFAF1	4.432	0.382	4.918	0.289	2.32E-06	-1.40	3.73E-04	ADT
WAS	10.058	0.151	9.85	0.166	2.35E-06	1.16	3.74E-04	ADT
COG7	5.319	0.158	5.553	0.209	2.37E-06	-1.18	3.74E-04	ADT
RAX	6.03	0.13	5.853	0.14	2.43E-06	1.13	3.74E-04	ADT
WDR55	5.836	0.208	6.127	0.239	2.41E-06	-1.22	3.74E-04	ADT
CORO2B	4.762	0.118	4.59	0.151	2.29E-06	1.13	3.75E-04	ADT
RAB4B	7.522	0.217	7.832	0.262	2.32E-06	-1.24	3.76E-04	ADT
FBXO18	7.398	0.209	7.66	0.142	2.41E-06	-1.20	3.77E-04	ADT
TEX22	5.137	0.163	4.875	0.256	2.50E-06	1.20	3.81E-04	ADT
NECAP2	8.009	0.138	8.194	0.14	2.55E-06	-1.14	3.85E-04	ADT
C3orf58	5.097	0.433	5.681	0.454	2.67E-06	-1.50	4.00E-04	ADT
ATP6V1F	7.579	0.237	7.895	0.242	2.72E-06	-1.24	4.04E-04	ADT
PSMA6	7.404	0.347	7.838	0.254	2.87E-06	-1.35	4.23E-04	ADT
FAM123A	5.581	0.115	5.422	0.132	3.22E-06	1.12	4.70E-04	ADT

SLC30A5	7.733	0.275	8.098	0.286	3.29E-06	-1.29	4.77E-04	ADT
TGM2	5.403	0.083	5.283	0.111	3.41E-06	1.09	4.89E-04	ADT
ARHGAP25	11.401	0.189	11.645	0.179	3.63E-06	-1.18	5.17E-04	ADT
C16orf93	6.873	0.267	7.244	0.322	3.74E-06	-1.29	5.29E-04	ADT
SHANK3	5.977	0.119	5.822	0.121	3.86E-06	1.11	5.42E-04	ADT
VPS33A	5.858	0.237	6.155	0.199	3.90E-06	-1.23	5.42E-04	ADT
GLA	6.79	0.276	7.145	0.262	4.00E-06	-1.28	5.52E-04	ADT
ZNF865	7.345	0.118	7.199	0.094	4.10E-06	1.11	5.62E-04	ADT
UBE2Z	7.198	0.192	7.457	0.221	4.64E-06	-1.20	6.27E-04	ADT
TCEANC2	5.917	0.223	6.195	0.191	4.62E-06	-1.21	6.28E-04	ADT
SGPP1	6.553	0.142	6.302	0.273	4.91E-06	1.19	6.58E-04	ADT
MANBAL	7.876	0.256	8.197	0.232	4.98E-06	-1.25	6.61E-04	ADT
TONSL	6.353	0.123	6.171	0.178	5.09E-06	1.13	6.72E-04	ADT
DCTN3	7.979	0.18	8.225	0.216	5.15E-06	-1.19	6.74E-04	ADT
DACT1	6.611	0.104	6.472	0.118	5.32E-06	1.10	6.91E-04	ADT
LENG9	5.923	0.144	5.711	0.206	5.41E-06	1.16	6.98E-04	ADT
CNOT8	7.687	0.225	7.972	0.216	5.48E-06	-1.22	7.02E-04	ADT
BPIFB1	4.805	0.091	4.689	0.088	5.53E-06	1.08	7.03E-04	ADT
STX5	7.641	0.166	7.898	0.262	5.77E-06	-1.19	7.29E-04	ADT
SKI	8.08	0.18	7.841	0.207	5.86E-06	1.18	7.35E-04	ADT
LILRA2	9.863	0.201	9.588	0.248	6.18E-06	1.21	7.58E-04	ADT
KLHL17	6.346	0.106	6.21	0.11	6.14E-06	1.10	7.60E-04	ADT
FOXO1	7.558	0.124	7.385	0.162	6.13E-06	1.13	7.63E-04	ADT
TBX10	5.643	0.159	5.451	0.13	6.26E-06	1.14	7.63E-04	ADT
SLC3A2	6.677	0.22	7.032	0.377	6.46E-06	-1.28	7.82E-04	ADT
SRCIN1	5.655	0.1	5.53	0.096	6.74E-06	1.09	8.10E-04	ADT
ARX	6.435	0.122	6.276	0.137	6.98E-06	1.12	8.18E-04	ADT
VPS11	6.764	0.274	7.115	0.289	6.94E-06	-1.28	8.18E-04	ADT
ZNF219	5.773	0.131	5.601	0.147	6.93E-06	1.13	8.22E-04	ADT
PDGFB	5.341	0.127	5.181	0.127	6.91E-06	1.12	8.25E-04	ADT
PDIA6	6.079	0.214	6.358	0.239	7.11E-06	-1.21	8.27E-04	ADT
TRADD	7.495	0.107	7.655	0.161	7.22E-06	-1.12	8.29E-04	ADT
PKN3	5.836	0.126	5.684	0.106	7.20E-06	1.11	8.31E-04	ADT
PYCRL	6.052	0.139	5.874	0.149	7.29E-06	1.13	8.32E-04	ADT
NHEJ1	4.814	0.168	5.037	0.198	7.70E-06	-1.17	8.73E-04	ADT
SNPH	6.559	0.145	6.373	0.159	7.82E-06	1.14	8.75E-04	ADT
ATP6V1B2	11.374	0.232	11.646	0.162	7.78E-06	-1.21	8.76E-04	ADT
IQSEC3	6.379	0.093	6.257	0.109	8.13E-06	1.09	8.99E-04	ADT
SSNA1	6.727	0.191	6.975	0.215	8.11E-06	-1.19	9.02E-04	ADT
CORO1B	6.778	0.2	7.021	0.181	8.54E-06	-1.18	9.27E-04	ADT
ADAT3	6.618	0.172	6.409	0.156	8.66E-06	1.16	9.28E-04	ADT
MAGED4B	5.76	0.094	5.641	0.101	8.62E-06	1.09	9.30E-04	ADT
ZNF579	7.221	0.14	7.037	0.165	8.54E-06	1.14	9.32E-04	ADT
ZSWIM4	6.575	0.081	6.466	0.102	8.50E-06	1.08	9.34E-04	ADT
GPR108	8.225	0.206	8.485	0.217	8.96E-06	-1.20	9.54E-04	ADT
SPTBN5	6	0.105	5.871	0.101	9.40E-06	1.09	9.84E-04	ADT
FOXD4L2	6.04	0.143	5.853	0.167	9.31E-06	1.14	9.85E-04	ADT

EMID1	6.354	0.115	6.213	0.108	9.40E-06	1.10	9.89E-04	ADT
SMU1	7.289	0.229	7.559	0.184	9.53E-06	-1.21	9.91E-04	ADT
CABP1	5.333	0.138	5.158	0.15	9.98E-06	1.13	1.03E-03	ADT
CLK2	8.243	0.141	8.445	0.205	9.96E-06	-1.15	1.03E-03	ADT
CDC42BPG	6.248	0.116	6.109	0.106	1.04E-05	1.10	1.06E-03	ADT
EEF1A2	5.995	0.135	5.825	0.147	1.10E-05	1.13	1.11E-03	ADT
NUMBL	7.172	0.111	7.035	0.113	1.11E-05	1.10	1.11E-03	ADT
PRM1	4.824	0.188	4.58	0.222	1.10E-05	1.18	1.11E-03	ADT
PABPC1L	7.554	0.157	7.34	0.211	1.14E-05	1.16	1.13E-03	ADT
BCL3	9.831	0.272	9.518	0.201	1.16E-05	1.24	1.15E-03	ADT
MLH1	5.134	0.252	5.453	0.28	1.16E-05	-1.25	1.15E-03	ADT
PROZ	4.745	0.142	4.577	0.127	1.25E-05	1.12	1.23E-03	ADT
WDR73	6.258	0.208	6.501	0.173	1.29E-05	-1.18	1.24E-03	ADT
STAMBP	6.478	0.174	6.692	0.181	1.28E-05	-1.16	1.24E-03	ADT
DBR1	4.094	0.222	4.373	0.244	1.28E-05	-1.21	1.25E-03	ADT
NDUFC1	4.213	0.186	4.436	0.179	1.33E-05	-1.17	1.28E-03	ADT
PPM1D	8.161	0.182	8.384	0.19	1.39E-05	-1.17	1.33E-03	ADT
NPAS4	4.489	0.088	4.375	0.11	1.41E-05	1.08	1.33E-03	ADT
DMRT3	5.204	0.14	5.034	0.143	1.41E-05	1.13	1.33E-03	ADT
SHROOM2	5.009	0.13	4.854	0.121	1.42E-05	1.11	1.33E-03	ADT
ATP5H	6.938	0.396	7.409	0.369	1.40E-05	-1.39	1.34E-03	ADT
RBM20	4.855	0.126	4.711	0.1	1.44E-05	1.10	1.34E-03	ADT
MATR3	7.215	0.276	7.542	0.259	1.49E-05	-1.25	1.38E-03	ADT
TAF11	5.909	0.247	6.207	0.247	1.50E-05	-1.23	1.38E-03	ADT
CYB561D2	6.353	0.229	6.622	0.207	1.52E-05	-1.20	1.40E-03	ADT
RPUSD1	6.149	0.099	6.026	0.11	1.56E-05	1.09	1.42E-03	ADT
PCBD2	4.626	0.195	4.858	0.189	1.61E-05	-1.17	1.46E-03	ADT
CCDC102A	5.995	0.148	5.812	0.163	1.62E-05	1.14	1.46E-03	ADT
NTAN1	6.298	0.315	6.718	0.422	1.69E-05	-1.34	1.51E-03	ADT
NAGK	9.438	0.334	9.819	0.271	1.74E-05	-1.30	1.56E-03	ADT
SPPL3	7.286	0.178	7.479	0.097	1.77E-05	-1.14	1.57E-03	ADT
SAFB	7.665	0.111	7.794	0.099	1.76E-05	-1.09	1.57E-03	ADT
TCEB3C	5.022	0.109	4.879	0.143	1.78E-05	1.10	1.57E-03	ADT
WDR47	6.676	0.246	6.952	0.184	1.82E-05	-1.21	1.60E-03	ADT
SARS	7.742	0.251	8.045	0.264	1.92E-05	-1.23	1.60E-03	ADT
ADD1	9.349	0.21	9.585	0.167	1.95E-05	-1.18	1.60E-03	ADT
CERS2	8.249	0.171	8.447	0.155	1.95E-05	-1.15	1.60E-03	ADT
FSD1	5.605	0.106	5.474	0.119	1.92E-05	1.10	1.60E-03	ADT
ECHDC3	6.86	0.288	6.42	0.5	1.94E-05	1.36	1.60E-03	ADT
VPS39	8.173	0.167	8.361	0.131	1.92E-05	-1.14	1.61E-03	ADT
NKX6-2	7.135	0.126	6.97	0.164	1.86E-05	1.12	1.61E-03	ADT
TEX261	6.86	0.161	7.068	0.203	1.91E-05	-1.16	1.61E-03	ADT
EYA2	4.949	0.094	4.834	0.104	1.90E-05	1.08	1.61E-03	ADT
IGF2BP1	4.556	0.118	4.417	0.115	1.90E-05	1.10	1.62E-03	ADT
RAPSN	6.459	0.1	6.297	0.189	1.86E-05	1.12	1.62E-03	ADT
GFPT1	4.869	0.257	5.187	0.294	2.00E-05	-1.25	1.62E-03	ADT
KLC3	6.015	0.139	5.853	0.127	1.90E-05	1.12	1.62E-03	ADT

GATAD1	5.77	0.167	5.968	0.165	1.99E-05	-1.15	1.62E-03	ADT
KCNA1	4.551	0.12	4.401	0.141	1.89E-05	1.11	1.63E-03	ADT
TNFAIP8L2-SCNM	7.484	0.205	7.731	0.218	2.02E-05	-1.19	1.63E-03	ADT
YIPF3	9.389	0.232	9.675	0.262	1.89E-05	-1.22	1.63E-03	ADT
SAP30BP	7.225	0.184	7.433	0.154	2.05E-05	-1.16	1.63E-03	ADT
SYS1	7.175	0.13	7.341	0.163	2.04E-05	-1.12	1.63E-03	ADT
ATXN3	7.123	0.474	7.647	0.332	2.04E-05	-1.44	1.64E-03	ADT
FAM175B	6.358	0.306	6.696	0.222	2.08E-05	-1.26	1.64E-03	ADT
IFT20	12.861	0.081	12.952	0.067	2.07E-05	-1.07	1.64E-03	ADT
FAM169B	4.465	0.19	4.25	0.161	2.17E-05	1.16	1.68E-03	ADT
ABCB9	5.316	0.119	5.175	0.119	2.16E-05	1.10	1.68E-03	ADT
ZFPM1	6.857	0.137	6.698	0.131	2.17E-05	1.12	1.68E-03	ADT
ITGAL	9.314	0.288	9.63	0.196	2.15E-05	-1.24	1.68E-03	ADT
MEX3D	6.023	0.125	5.879	0.116	2.15E-05	1.10	1.69E-03	ADT
PSPH	4.577	0.378	5.028	0.398	2.26E-05	-1.37	1.72E-03	ADT
KIAA0284	6.268	0.108	6.144	0.101	2.27E-05	1.09	1.73E-03	ADT
ELOVL1	7.734	0.225	7.991	0.2	2.25E-05	-1.19	1.73E-03	ADT
RETSAT	5.719	0.146	5.91	0.195	2.26E-05	-1.14	1.73E-03	ADT
GDI2	8.763	0.278	9.066	0.191	2.31E-05	-1.23	1.75E-03	ADT
ATP6V1D	6.631	0.418	7.083	0.27	2.35E-05	-1.37	1.75E-03	ADT
PEX19	5.74	0.134	5.94	0.227	2.34E-05	-1.15	1.75E-03	ADT
CHCHD10	6.155	0.143	5.964	0.201	2.33E-05	1.14	1.76E-03	ADT
MTF2	6.339	0.25	6.635	0.26	2.40E-05	-1.23	1.78E-03	ADT
IKBKB	7.357	0.108	7.48	0.096	2.41E-05	-1.09	1.78E-03	ADT
MARCH9	5.331	0.161	5.14	0.171	2.45E-05	1.14	1.80E-03	ADT
CLASRP	7.314	0.171	7.125	0.136	2.46E-05	1.14	1.81E-03	ADT
POLR2E	7.411	0.173	7.633	0.225	2.48E-05	-1.17	1.81E-03	ADT
CCDC53	6.522	0.335	6.897	0.282	2.55E-05	-1.30	1.86E-03	ADT
LRRC59	7.329	0.159	7.515	0.16	2.58E-05	-1.14	1.87E-03	ADT
GRIN2D	6.334	0.119	6.199	0.108	2.66E-05	1.10	1.91E-03	ADT
LILRA4	5.946	0.144	5.777	0.148	2.66E-05	1.12	1.92E-03	ADT
LCN9	4.325	0.139	4.158	0.154	2.71E-05	1.12	1.94E-03	ADT
ZNF565	4.11	0.283	4.473	0.373	2.76E-05	-1.29	1.97E-03	ADT
GATA5	6.794	0.147	6.628	0.131	2.83E-05	1.12	2.00E-03	ADT
CCND1	5.764	0.081	5.66	0.107	2.85E-05	1.07	2.01E-03	ADT
GRIK5	5.767	0.107	5.646	0.1	2.83E-05	1.09	2.01E-03	ADT
CD300LB	6.854	0.312	7.249	0.399	2.87E-05	-1.31	2.02E-03	ADT
SARDH	5.909	0.094	5.794	0.111	2.91E-05	1.08	2.02E-03	ADT
SLC27A1	5.721	0.12	5.575	0.14	2.92E-05	1.11	2.03E-03	ADT
GUCY2D	5.577	0.117	5.439	0.125	2.90E-05	1.10	2.03E-03	ADT
C20orf151	6.72	0.125	6.575	0.125	2.99E-05	1.11	2.03E-03	ADT
SPTBN4	5.775	0.101	5.654	0.114	2.98E-05	1.09	2.03E-03	ADT
SSX5	3.697	0.209	3.454	0.214	2.97E-05	1.18	2.04E-03	ADT
GTF2E1	5.387	0.264	5.679	0.221	2.96E-05	-1.22	2.04E-03	ADT
PIP5K1A	7.261	0.217	7.5	0.177	2.97E-05	-1.18	2.04E-03	ADT
CNPPD1	7.176	0.114	7.326	0.16	3.03E-05	-1.11	2.05E-03	ADT
RUNDC3A	5.678	0.11	5.543	0.132	3.05E-05	1.10	2.06E-03	ADT

ANGPTL4	5.207	0.131	5.057	0.128	3.07E-05	1.11	2.07E-03	ADT
JAKMIP3	5.056	0.114	4.92	0.127	3.12E-05	1.10	2.09E-03	ADT
EMC3	7.296	0.29	7.61	0.218	3.15E-05	-1.24	2.10E-03	ADT
DNAJC14	6.895	0.201	7.114	0.157	3.15E-05	-1.16	2.10E-03	ADT
CD300C	7.194	0.274	7.515	0.294	3.20E-05	-1.25	2.12E-03	ADT
CCNL2	7.219	0.151	7.392	0.148	3.22E-05	-1.13	2.12E-03	ADT
TMEM65	6.591	0.17	6.788	0.174	3.24E-05	-1.15	2.13E-03	ADT
VSTM2B	6.318	0.113	6.181	0.133	3.31E-05	1.10	2.17E-03	ADT
RASSF3	9.337	0.222	9.066	0.267	3.38E-05	1.21	2.21E-03	ADT
OR2T29	2.536	0.138	2.7	0.155	3.44E-05	-1.12	2.21E-03	ADT
MED7	5.238	0.223	5.484	0.193	3.44E-05	-1.19	2.22E-03	ADT
CRBN	8.386	0.319	8.759	0.342	3.42E-05	-1.30	2.22E-03	ADT
FLJ44635	7.593	0.172	7.346	0.285	3.44E-05	1.19	2.23E-03	ADT
CGNL1	4.339	0.124	4.189	0.148	3.61E-05	1.11	2.29E-03	ADT
ZNF836	4.837	0.184	5.054	0.206	3.60E-05	-1.16	2.29E-03	ADT
COPS7A	7.044	0.102	7.173	0.133	3.58E-05	-1.09	2.29E-03	ADT
RBM3	7.194	0.252	7.477	0.239	3.66E-05	-1.22	2.29E-03	ADT
FDPS	5.626	0.139	5.795	0.169	3.65E-05	-1.12	2.29E-03	ADT
LRRC45	5.783	0.091	5.675	0.105	3.64E-05	1.08	2.30E-03	ADT
SPEF1	5.702	0.164	5.518	0.155	3.60E-05	1.14	2.30E-03	ADT
ZNF266	6.37	0.269	6.679	0.278	3.72E-05	-1.24	2.32E-03	ADT
R3HDM4	10.159	0.167	10.375	0.231	3.73E-05	-1.16	2.32E-03	ADT
CYTH2	7.291	0.129	7.461	0.186	3.76E-05	-1.13	2.33E-03	ADT
NKD2	6.034	0.167	5.834	0.196	3.81E-05	1.15	2.35E-03	ADT
PHF21B	6.414	0.126	6.276	0.112	3.83E-05	1.10	2.36E-03	ADT
ARFRP1	6.435	0.077	6.341	0.094	3.88E-05	1.07	2.38E-03	ADT
TMEM57	6.519	0.327	6.887	0.317	3.93E-05	-1.29	2.40E-03	ADT
NXT2	4.906	0.218	5.166	0.254	3.97E-05	-1.20	2.42E-03	ADT
CD300A	9.328	0.244	9.598	0.223	3.99E-05	-1.21	2.42E-03	ADT
PITPNM3	5.409	0.115	5.282	0.105	4.07E-05	1.09	2.47E-03	ADT
ZNF692	6.919	0.115	7.073	0.173	4.11E-05	-1.11	2.47E-03	ADT
SCNN1B	5.16	0.115	5.035	0.1	4.13E-05	1.09	2.47E-03	ADT
ZNF750	4.256	0.145	4.086	0.164	4.10E-05	1.13	2.48E-03	ADT
FAM134A	7.575	0.182	7.794	0.218	4.18E-05	-1.16	2.48E-03	ADT
SYNM	5.373	0.12	5.237	0.119	4.15E-05	1.10	2.48E-03	ADT
LILRA1	10.133	0.181	9.912	0.223	4.17E-05	1.17	2.48E-03	ADT
ZNF189	6.619	0.285	6.965	0.35	4.22E-05	-1.27	2.50E-03	ADT
TMUB2	7.298	0.152	7.488	0.201	4.26E-05	-1.14	2.50E-03	ADT
ELMOD3	5.727	0.103	5.873	0.173	4.26E-05	-1.11	2.51E-03	ADT
KDM6B	9.6	0.18	9.401	0.168	4.31E-05	1.15	2.52E-03	ADT
LRRC16B	5.542	0.107	5.427	0.09	4.41E-05	1.08	2.55E-03	ADT
DNASE1L2	6.03	0.115	5.899	0.119	4.39E-05	1.10	2.55E-03	ADT
HN1	8.998	0.252	9.278	0.243	4.41E-05	-1.21	2.56E-03	ADT
SPANXD	4.38	0.518	3.782	0.562	4.38E-05	1.51	2.56E-03	ADT
PRX	6.361	0.125	6.225	0.107	4.47E-05	1.10	2.58E-03	ADT
PDE12	4.764	0.281	5.103	0.344	4.54E-05	-1.26	2.60E-03	ADT
TXNL4B	6.488	0.285	6.79	0.219	4.54E-05	-1.23	2.60E-03	ADT

SASH3	8.287	0.152	8.467	0.177	4.58E-05	-1.13	2.61E-03	ADT
GRIN2C	5.694	0.127	5.556	0.113	4.68E-05	1.10	2.66E-03	ADT
SIX5	5.91	0.118	5.766	0.151	4.71E-05	1.10	2.67E-03	ADT
ELOF1	7.163	0.227	7.418	0.228	4.76E-05	-1.19	2.69E-03	ADT
FADS6	5.527	0.181	5.322	0.187	4.78E-05	1.15	2.69E-03	ADT
TNK1	5.645	0.125	5.509	0.116	4.83E-05	1.10	2.70E-03	ADT
C8orf40	7.767	0.196	7.525	0.255	4.83E-05	1.18	2.71E-03	ADT
OR1D2	5.005	0.244	4.728	0.259	4.86E-05	1.21	2.71E-03	ADT
DHDDS	6.725	0.167	6.958	0.276	4.96E-05	-1.18	2.76E-03	ADT
GPANK1	6.66	0.154	6.853	0.21	4.98E-05	-1.14	2.76E-03	ADT
PROSC	7.291	0.19	7.497	0.173	5.07E-05	-1.15	2.80E-03	ADT
MOCS3	4.847	0.217	5.11	0.273	5.17E-05	-1.20	2.84E-03	ADT
THEG	5.418	0.113	5.283	0.138	5.21E-05	1.10	2.84E-03	ADT
IST1	9.011	0.157	9.176	0.119	5.17E-05	-1.12	2.85E-03	ADT
FAM96A	6.721	0.295	7.054	0.31	5.20E-05	-1.26	2.85E-03	ADT
TAF7	6.199	0.224	6.474	0.293	5.30E-05	-1.21	2.88E-03	ADT
SFT2D2	8.022	0.32	8.356	0.242	5.33E-05	-1.26	2.89E-03	ADT
SOX12	5.934	0.1	5.814	0.124	5.37E-05	1.09	2.89E-03	ADT
CISD2	4.467	0.251	4.744	0.242	5.38E-05	-1.21	2.89E-03	ADT
PLEKHG4	5.417	0.105	5.304	0.095	5.35E-05	1.08	2.89E-03	ADT
LMOD3	3.073	0.147	2.901	0.172	5.42E-05	1.13	2.90E-03	ADT
ZNF193	5.062	0.159	5.286	0.27	5.49E-05	-1.17	2.90E-03	ADT
ACLY	7.606	0.196	7.809	0.142	5.44E-05	-1.15	2.90E-03	ADT
KLHDC8B	5.95	0.269	6.29	0.375	5.48E-05	-1.27	2.91E-03	ADT
NKIRAS2	6.886	0.108	7.045	0.194	5.48E-05	-1.12	2.92E-03	ADT
SIRT7	7.136	0.117	7.271	0.131	5.57E-05	-1.10	2.94E-03	ADT
ZNF673	6.139	0.176	6.33	0.165	5.61E-05	-1.14	2.95E-03	ADT
FUCA2	4.764	0.24	5.032	0.247	5.68E-05	-1.20	2.96E-03	ADT
KCNH4	5.509	0.094	5.394	0.122	5.67E-05	1.08	2.96E-03	ADT
ITGAV	4.91	0.24	5.18	0.255	5.72E-05	-1.21	2.97E-03	ADT
ACBD3	7.72	0.189	7.924	0.172	5.70E-05	-1.15	2.97E-03	ADT
TRIM52	6.546	0.234	6.804	0.231	5.65E-05	-1.20	2.97E-03	ADT
PLEKHA3	7.091	0.127	7.228	0.117	5.76E-05	-1.10	2.98E-03	ADT
ZNF436	4.809	0.109	4.935	0.124	5.79E-05	-1.09	2.98E-03	ADT
TIA1	7.046	0.518	7.586	0.404	5.84E-05	-1.45	2.99E-03	ADT
FAM189A1	5.651	0.122	5.514	0.129	5.83E-05	1.10	3.00E-03	ADT
PPP2R4	5.547	0.187	5.752	0.183	5.88E-05	-1.15	3.00E-03	ADT
RBMXL2	6.496	0.135	6.342	0.151	6.00E-05	1.11	3.06E-03	ADT
RNF4	7.647	0.185	7.839	0.145	6.06E-05	-1.14	3.07E-03	ADT
GPR144	6.396	0.101	6.282	0.11	6.05E-05	1.08	3.07E-03	ADT
ARMCX4	4.348	0.102	4.235	0.104	6.25E-05	1.08	3.14E-03	ADT
ZNF616	4.192	0.265	4.486	0.274	6.28E-05	-1.23	3.14E-03	ADT
UNC5B	5.975	0.099	5.859	0.117	6.24E-05	1.08	3.14E-03	ADT
GNAI2	10.641	0.112	10.772	0.136	6.24E-05	-1.10	3.15E-03	ADT
SHC2	5.695	0.103	5.576	0.119	6.33E-05	1.09	3.16E-03	ADT
SMTN	6.407	0.121	6.279	0.107	6.36E-05	1.09	3.17E-03	ADT
RBMS1	8.854	0.197	8.647	0.164	6.40E-05	1.15	3.18E-03	ADT

BRSK1	6.013	0.091	5.904	0.115	6.42E-05	1.08	3.18E-03	ADT
PCIF1	8.054	0.193	8.253	0.149	6.52E-05	-1.15	3.22E-03	ADT
DUSP15	5.757	0.118	5.622	0.134	6.61E-05	1.10	3.25E-03	ADT
ELMOD2	4.768	0.287	5.068	0.238	6.59E-05	-1.23	3.25E-03	ADT
BLVRA	5.384	0.194	5.625	0.269	6.63E-05	-1.18	3.25E-03	ADT
RIC8A	7.769	0.194	7.997	0.239	6.65E-05	-1.17	3.25E-03	ADT
MSL1	9.616	0.144	9.437	0.2	6.80E-05	1.13	3.31E-03	ADT
RUFY1	7.339	0.209	7.557	0.174	6.82E-05	-1.16	3.31E-03	ADT
KCNC3	6.309	0.116	6.186	0.104	6.84E-05	1.09	3.32E-03	ADT
TMOD2	6.91	0.369	6.5	0.389	6.90E-05	1.33	3.33E-03	ADT
LGALS3	9.816	0.13	9.658	0.172	6.98E-05	1.12	3.35E-03	ADT
DKK3	5.891	0.079	5.791	0.115	6.96E-05	1.07	3.35E-03	ADT
CCDC64B	5.969	0.138	5.805	0.175	7.03E-05	1.12	3.37E-03	ADT
RIBC2	4.003	0.157	3.832	0.158	7.07E-05	1.13	3.38E-03	ADT
FOSL1	6.24	0.146	6.076	0.16	7.24E-05	1.12	3.45E-03	ADT
GLTP	6.8	0.18	7.004	0.205	7.31E-05	-1.15	3.45E-03	ADT
ZMAT3	5.205	0.198	5.417	0.186	7.30E-05	-1.16	3.46E-03	ADT
SCAF1	6.993	0.093	6.896	0.08	7.30E-05	1.07	3.47E-03	ADT
RNF44	8.251	0.218	8.017	0.208	7.41E-05	1.18	3.50E-03	ADT
HIST2H4A	7.111	0.264	7.438	0.369	7.46E-05	-1.25	3.50E-03	ADT
ARL8B	8.127	0.255	8.39	0.206	7.44E-05	-1.20	3.50E-03	ADT
MCFD2	5.447	0.127	5.591	0.144	7.52E-05	-1.10	3.52E-03	ADT
LOXL4	5.391	0.121	5.253	0.141	7.61E-05	1.10	3.55E-03	ADT
ATG5	5.528	0.259	5.797	0.216	7.70E-05	-1.20	3.57E-03	ADT
HSH2D	9.107	0.279	9.408	0.274	7.69E-05	-1.23	3.58E-03	ADT
DNAH17	4.47	0.07	4.391	0.078	7.79E-05	1.06	3.60E-03	ADT
RNASEL	7.12	0.301	7.453	0.323	7.78E-05	-1.26	3.60E-03	ADT
MFAP3	6.307	0.314	6.633	0.267	7.87E-05	-1.25	3.62E-03	ADT
SLCO2B1	4.436	0.121	4.308	0.112	7.87E-05	1.09	3.63E-03	ADT
VPS53	7.261	0.256	7.539	0.26	7.95E-05	-1.21	3.64E-03	ADT
EPS8L2	5.997	0.101	5.888	0.099	8.18E-05	1.08	3.74E-03	ADT
CNRIP1	7.666	0.219	7.426	0.232	8.21E-05	1.18	3.74E-03	ADT
FUT2	4.443	0.157	4.276	0.15	8.30E-05	1.12	3.77E-03	ADT
COL27A1	6.313	0.13	6.176	0.122	8.39E-05	1.10	3.80E-03	ADT
NXN	5.27	0.112	5.139	0.142	8.48E-05	1.10	3.81E-03	ADT
UQCRC2	8.071	0.279	8.351	0.2	8.48E-05	-1.21	3.81E-03	ADT
MLL4	7.705	0.161	7.538	0.141	8.44E-05	1.12	3.82E-03	ADT
PAPLN	6.532	0.088	6.436	0.091	8.56E-05	1.07	3.82E-03	ADT
ADAMTS17	5.594	0.093	5.494	0.094	8.54E-05	1.07	3.82E-03	ADT
PPP1R13L	6.202	0.123	6.076	0.102	8.47E-05	1.09	3.82E-03	ADT
USP21	7.034	0.145	7.219	0.219	8.61E-05	-1.14	3.83E-03	ADT
CCDC151	5.231	0.135	5.089	0.124	8.66E-05	1.10	3.85E-03	ADT
BEAN1	5.719	0.128	5.579	0.135	8.70E-05	1.10	3.85E-03	ADT
LIX1L	7.911	0.162	8.092	0.185	8.74E-05	-1.13	3.86E-03	ADT
BFAR	5.758	0.231	6.003	0.223	8.87E-05	-1.19	3.87E-03	ADT
ZNF701	7.034	0.232	7.28	0.22	8.85E-05	-1.19	3.87E-03	ADT
ORAI3	7.397	0.149	7.569	0.182	8.83E-05	-1.13	3.88E-03	ADT

SDHAF2	7.403	0.198	7.606	0.163	8.80E-05	-1.15	3.88E-03	ADT
PDIA2	5.739	0.141	5.589	0.137	8.82E-05	1.11	3.88E-03	ADT
ZNF143	6.349	0.261	6.613	0.205	9.00E-05	-1.20	3.92E-03	ADT
CCR10	5.007	0.254	4.717	0.304	9.07E-05	1.22	3.92E-03	ADT
PPP1R8	6.77	0.135	6.921	0.155	9.04E-05	-1.11	3.93E-03	ADT
FAM181B	6.038	0.161	5.862	0.174	9.06E-05	1.13	3.93E-03	ADT
FBF1	6.137	0.083	6.047	0.089	9.21E-05	1.06	3.97E-03	ADT
ANKRD6	4.878	0.21	5.131	0.285	9.29E-05	-1.19	4.00E-03	ADT
REG1A	3.977	0.16	4.151	0.17	9.31E-05	-1.13	4.00E-03	ADT
NXPH4	6.235	0.15	6.084	0.117	9.38E-05	1.11	4.02E-03	ADT
TOR1A	7.767	0.288	8.067	0.266	9.62E-05	-1.23	4.11E-03	ADT
POLR2A	8.95	0.124	8.819	0.119	9.65E-05	1.10	4.11E-03	ADT
HIST1H2AC	9.327	0.286	9.654	0.35	9.68E-05	-1.25	4.12E-03	ADT
PLXNB3	6.185	0.119	6.063	0.1	9.72E-05	1.09	4.12E-03	ADT
NAPG	6.039	0.233	6.29	0.242	9.82E-05	-1.19	4.12E-03	ADT
ZNF778	5.089	0.13	5.227	0.127	9.75E-05	-1.10	4.12E-03	ADT
C2orf56	6.03	0.21	6.249	0.196	9.81E-05	-1.16	4.13E-03	ADT
PSME3	6.447	0.334	6.82	0.387	9.80E-05	-1.30	4.14E-03	ADT
BRSK2	6.064	0.103	5.952	0.11	9.89E-05	1.08	4.14E-03	ADT
ARIH2	6.522	0.431	6.945	0.29	9.92E-05	-1.34	4.15E-03	ADT
UBE2R2	9.303	0.169	9.109	0.207	1.00E-04	1.14	4.17E-03	ADT
KCNK15	5.708	0.127	5.569	0.139	1.01E-04	1.10	4.21E-03	ADT
CCDC115	5.677	0.164	5.849	0.155	1.03E-04	-1.13	4.25E-03	ADT
UBC	11.582	0.104	11.684	0.076	1.03E-04	-1.07	4.26E-03	ADT
CAMSAP3	5.841	0.107	5.725	0.113	1.04E-04	1.08	4.27E-03	ADT
RHBDD2	8.996	0.177	9.198	0.217	1.04E-04	-1.15	4.28E-03	ADT
NOS2	4.73	0.109	4.617	0.101	1.05E-04	1.08	4.33E-03	ADT
NMRK2	5.921	0.167	5.75	0.149	1.06E-04	1.13	4.35E-03	ADT
EN1	6.08	0.151	5.912	0.174	1.07E-04	1.12	4.36E-03	ADT
TADA3	7.164	0.123	7.317	0.182	1.08E-04	-1.11	4.39E-03	ADT
OR5B3	3.572	0.165	3.763	0.21	1.08E-04	-1.14	4.40E-03	ADT
MTSS1L	6.025	0.112	5.906	0.114	1.09E-04	1.09	4.42E-03	ADT
DYNLRB1	8.16	0.196	8.38	0.235	1.10E-04	-1.16	4.46E-03	ADT
SF1	9.603	0.136	9.466	0.112	1.11E-04	1.10	4.49E-03	ADT
ADNP2	5.18	0.239	5.42	0.197	1.11E-04	-1.18	4.50E-03	ADT
LRRC4C	3.857	0.271	3.575	0.26	1.13E-04	1.22	4.56E-03	ADT
KRT78	5.444	0.152	5.288	0.14	1.14E-04	1.11	4.59E-03	ADT
KCTD17	6.791	0.141	6.655	0.091	1.15E-04	1.10	4.60E-03	ADT
ARHGEF1	8.901	0.12	8.774	0.126	1.15E-04	1.09	4.60E-03	ADT
ZBTB3	5.142	0.188	5.355	0.231	1.17E-04	-1.16	4.61E-03	ADT
TSGA10IP	6.28	0.14	6.131	0.146	1.18E-04	1.11	4.61E-03	ADT
UBTD2	4.467	0.169	4.646	0.174	1.17E-04	-1.13	4.62E-03	ADT
NFAM1	9.262	0.14	9.108	0.157	1.17E-04	1.11	4.62E-03	ADT
GGCX	6.019	0.16	6.206	0.214	1.17E-04	-1.14	4.63E-03	ADT
OTUB1	7.346	0.164	7.506	0.115	1.17E-04	-1.12	4.63E-03	ADT
WIZ	6.49	0.115	6.371	0.108	1.16E-04	1.09	4.63E-03	ADT
JMJD7	6.804	0.167	7.02	0.267	1.16E-04	-1.16	4.64E-03	ADT

GBX2	7.954	0.175	7.761	0.201	1.17E-04	1.14	4.64E-03	ADT
DSTYK	5.942	0.129	6.084	0.147	1.19E-04	-1.10	4.65E-03	ADT
SEC14L4	4.759	0.096	4.649	0.122	1.21E-04	1.08	4.72E-03	ADT
COPS2	6.156	0.325	6.474	0.238	1.21E-04	-1.25	4.72E-03	ADT
RASD1	5.814	0.18	5.623	0.189	1.22E-04	1.14	4.74E-03	ADT
XRCC6BP1	6.508	0.252	6.765	0.23	1.23E-04	-1.19	4.78E-03	ADT
DTX1	5.995	0.15	5.841	0.139	1.25E-04	1.11	4.82E-03	ADT
LAMA5	6.304	0.098	6.205	0.086	1.27E-04	1.07	4.90E-03	ADT
GLB1	7.181	0.197	7.385	0.193	1.28E-04	-1.15	4.92E-03	ADT
AKAP8L	8.349	0.125	8.501	0.181	1.28E-04	-1.11	4.93E-03	ADT
RAI2	7.768	0.183	7.584	0.161	1.29E-04	1.14	4.94E-03	ADT
FCER1G	9.327	0.394	9.783	0.519	1.29E-04	-1.37	4.95E-03	ADT
UFM1	5.597	0.278	5.88	0.255	1.30E-04	-1.22	4.96E-03	ADT
CTU2	6.511	0.122	6.381	0.133	1.31E-04	1.09	4.98E-03	ADT
CHFR	7.871	0.142	8.01	0.107	1.32E-04	-1.10	5.02E-03	ADT
EXOC3	8.475	0.165	8.663	0.21	1.33E-04	-1.14	5.02E-03	ADT
CCDC159	6.751	0.239	7.018	0.29	1.32E-04	-1.20	5.02E-03	ADT
IGDCC3	5.628	0.104	5.512	0.126	1.33E-04	1.08	5.03E-03	ADT
L1CAM	5.211	0.151	5.068	0.089	1.34E-04	1.10	5.05E-03	ADT
CDK2	5.645	0.243	5.924	0.316	1.34E-04	-1.21	5.05E-03	ADT
OPN1LW	5.366	0.146	5.21	0.159	1.35E-04	1.11	5.05E-03	ADT
PIGC	5.769	0.234	6.02	0.258	1.36E-04	-1.19	5.09E-03	ADT
RBM5	9.671	0.133	9.804	0.113	1.37E-04	-1.10	5.09E-03	ADT
MORF4L2	6.453	0.306	6.755	0.247	1.36E-04	-1.23	5.10E-03	ADT
TMEM238	6.61	0.181	6.399	0.243	1.38E-04	1.16	5.12E-03	ADT
RNF19B	8.832	0.164	9.022	0.218	1.39E-04	-1.14	5.17E-03	ADT
MAP2K2	7.551	0.13	7.698	0.164	1.39E-04	-1.11	5.17E-03	ADT
UBE3C	6.182	0.2	6.397	0.221	1.40E-04	-1.16	5.18E-03	ADT
SIK3	8.63	0.14	8.483	0.146	1.41E-04	1.11	5.20E-03	ADT
SPCS1	6.648	0.137	6.809	0.19	1.43E-04	-1.12	5.27E-03	ADT
INF2	6.258	0.157	6.099	0.146	1.44E-04	1.12	5.29E-03	ADT
FUCA1	5.795	0.274	6.086	0.295	1.44E-04	-1.22	5.29E-03	ADT
MLL2	8.993	0.155	8.833	0.153	1.46E-04	1.12	5.32E-03	ADT
MYH14	5.833	0.11	5.717	0.117	1.46E-04	1.08	5.33E-03	ADT
RBM4	7.603	0.151	7.771	0.183	1.46E-04	-1.12	5.34E-03	ADT
ANKRD24	6.09	0.119	5.964	0.127	1.48E-04	1.09	5.34E-03	ADT
SLC39A4	6.836	0.109	6.708	0.149	1.46E-04	1.09	5.34E-03	ADT
MPDU1	6.167	0.278	6.437	0.217	1.47E-04	-1.21	5.34E-03	ADT
EMR2	10.552	0.256	10.817	0.258	1.47E-04	-1.20	5.34E-03	ADT
SPTLC2	7.245	0.216	7.487	0.271	1.49E-04	-1.18	5.37E-03	ADT
F7	5.799	0.123	5.679	0.098	1.50E-04	1.09	5.41E-03	ADT
RPP25	4.855	0.162	4.698	0.127	1.53E-04	1.11	5.45E-03	ADT
STAM	7.879	0.21	8.083	0.164	1.53E-04	-1.15	5.46E-03	ADT
CYP46A1	5.674	0.116	5.558	0.105	1.52E-04	1.08	5.46E-03	ADT
ATP6V0C	11.56	0.135	11.705	0.155	1.53E-04	-1.11	5.46E-03	ADT
SP2	7.222	0.149	7.075	0.128	1.52E-04	1.11	5.47E-03	ADT
ALDOB	4.617	0.136	4.485	0.106	1.55E-04	1.10	5.51E-03	ADT

PSMA4	7.264	0.44	7.688	0.33	1.55E-04	-1.34	5.51E-03	ADT
NPFFR1	5.631	0.148	5.475	0.16	1.57E-04	1.11	5.52E-03	ADT
SCFD1	5.257	0.395	5.684	0.457	1.56E-04	-1.34	5.53E-03	ADT
FAM25B	6.707	0.215	6.492	0.195	1.57E-04	1.16	5.53E-03	ADT
MIS12	5.061	0.299	5.364	0.282	1.56E-04	-1.23	5.54E-03	ADT
DARS2	5.112	0.227	5.344	0.223	1.58E-04	-1.17	5.54E-03	ADT
PHF21A	11.934	0.197	11.723	0.222	1.59E-04	1.16	5.57E-03	ADT
SDK2	5.239	0.108	5.119	0.134	1.60E-04	1.09	5.58E-03	ADT
ZNF10	2.759	0.156	2.938	0.209	1.61E-04	-1.13	5.63E-03	ADT
PRR25	5.238	0.249	4.976	0.269	1.62E-04	1.20	5.65E-03	ADT
ZNF541	5.054	0.125	4.932	0.104	1.63E-04	1.09	5.69E-03	ADT
C1orf130	11.359	0.204	11.148	0.209	1.64E-04	1.16	5.69E-03	ADT
APOA4	4.999	0.228	4.781	0.168	1.66E-04	1.16	5.73E-03	ADT
ESRRB	4.348	0.164	4.176	0.177	1.66E-04	1.13	5.74E-03	ADT
PRDX6	6.508	0.233	6.748	0.24	1.67E-04	-1.18	5.75E-03	ADT
MYL7	4.384	0.199	4.185	0.183	1.67E-04	1.15	5.77E-03	ADT
NUDT14	5.622	0.17	5.455	0.146	1.68E-04	1.12	5.79E-03	ADT
ZNF25	3.496	0.53	4.049	0.569	1.72E-04	-1.47	5.90E-03	ADT
EIF4ENIF1	6.304	0.192	6.494	0.169	1.74E-04	-1.14	5.94E-03	ADT
SLC22A16	4.035	0.152	4.226	0.244	1.74E-04	-1.14	5.95E-03	ADT
ZFYVE1	6.915	0.132	7.046	0.121	1.75E-04	-1.10	5.96E-03	ADT
RAET1G	6.577	0.2	6.366	0.22	1.76E-04	1.16	5.98E-03	ADT
XAB2	7.231	0.097	7.341	0.129	1.77E-04	-1.08	6.00E-03	ADT
ST7L	4.745	0.228	5.002	0.299	1.79E-04	-1.19	6.03E-03	ADT
MYLK2	4.999	0.148	4.855	0.125	1.79E-04	1.10	6.03E-03	ADT
APTX	6.05	0.244	6.33	0.332	1.80E-04	-1.21	6.04E-03	ADT
USF1	8.655	0.236	8.898	0.245	1.79E-04	-1.18	6.04E-03	ADT
ITPKA	5.868	0.16	5.7	0.174	1.79E-04	1.12	6.04E-03	ADT
KIFC3	6.397	0.123	6.269	0.133	1.79E-04	1.09	6.05E-03	ADT
COL6A1	6.029	0.098	5.927	0.104	1.78E-04	1.07	6.05E-03	ADT
ACTN4	10.289	0.103	10.397	0.112	1.82E-04	-1.08	6.07E-03	ADT
BCAS3	8.425	0.202	8.615	0.141	1.82E-04	-1.14	6.08E-03	ADT
IMPA1	5.922	0.207	6.134	0.214	1.83E-04	-1.16	6.09E-03	ADT
ALPK3	5.654	0.166	5.491	0.147	1.84E-04	1.12	6.09E-03	ADT
PLSCR3	7.855	0.192	7.66	0.191	1.83E-04	1.14	6.10E-03	ADT
EMC6	5.285	0.213	5.51	0.239	1.83E-04	-1.17	6.10E-03	ADT
SIPA1L1	9.912	0.19	9.696	0.254	1.85E-04	1.16	6.12E-03	ADT
TMEM121	6.66	0.096	6.55	0.13	1.86E-04	1.08	6.14E-03	ADT
SLC35A3	4.228	0.343	4.57	0.324	1.86E-04	-1.27	6.15E-03	ADT
BAGE	7.934	0.148	8.104	0.203	1.88E-04	-1.13	6.15E-03	ADT
MPG	6.968	0.125	6.848	0.1	1.87E-04	1.09	6.15E-03	ADT
TMED7	6.913	0.232	7.133	0.177	1.87E-04	-1.16	6.16E-03	ADT
MFSD1	7.883	0.344	8.21	0.26	1.91E-04	-1.25	6.24E-03	ADT
CPAMD8	5.824	0.082	5.731	0.11	1.91E-04	1.07	6.24E-03	ADT
RET	4.788	0.105	4.686	0.089	1.92E-04	1.07	6.27E-03	ADT
FKRP	5.757	0.128	5.622	0.145	1.94E-04	1.10	6.32E-03	ADT
ZNRF4	4.871	0.201	4.677	0.166	1.95E-04	1.14	6.33E-03	ADT

EVI5L	6.264	0.089	6.172	0.095	1.95E-04	1.07	6.33E-03	ADT
SLC9A3R2	6.35	0.112	6.233	0.122	1.98E-04	1.08	6.40E-03	ADT
SSU72	7.63	0.202	7.824	0.168	1.99E-04	-1.14	6.40E-03	ADT
FOXD2	6.414	0.136	6.271	0.155	1.99E-04	1.10	6.42E-03	ADT
SMARCD2	7.538	0.131	7.674	0.144	1.99E-04	-1.10	6.42E-03	ADT
UMODL1	4.732	0.11	4.619	0.117	2.00E-04	1.08	6.44E-03	ADT
ALOX12B	4.703	0.103	4.588	0.137	2.04E-04	1.08	6.48E-03	ADT
POLDIP3	6.658	0.126	6.78	0.108	2.04E-04	-1.09	6.48E-03	ADT
SOLH	6.684	0.107	6.575	0.108	2.03E-04	1.08	6.49E-03	ADT
ARHGAP22	4.986	0.119	4.866	0.121	2.03E-04	1.09	6.49E-03	ADT
NPAS1	5.515	0.122	5.396	0.107	2.03E-04	1.09	6.49E-03	ADT
SLC25A3	7.161	0.201	7.37	0.22	2.02E-04	-1.16	6.49E-03	ADT
ZDHHC13	6.365	0.277	6.662	0.331	2.06E-04	-1.23	6.50E-03	ADT
RBM14	7.471	0.215	7.72	0.302	2.06E-04	-1.19	6.51E-03	ADT
EXOC7	7.399	0.138	7.555	0.186	2.06E-04	-1.11	6.51E-03	ADT
ACBD5	6.366	0.238	6.584	0.144	2.06E-04	-1.16	6.51E-03	ADT
ARNT	8.224	0.195	8.42	0.194	2.05E-04	-1.15	6.52E-03	ADT
TBX1	5.915	0.095	5.806	0.132	2.10E-04	1.08	6.59E-03	ADT
SYAP1	4.579	0.464	5.04	0.448	2.11E-04	-1.38	6.63E-03	ADT
DBT	4.8	0.286	5.071	0.229	2.13E-04	-1.21	6.67E-03	ADT
VPS54	4.509	0.25	4.754	0.231	2.16E-04	-1.19	6.72E-03	ADT
SCPEP1	7.985	0.362	8.391	0.483	2.15E-04	-1.33	6.72E-03	ADT
RP1L1	5.485	0.158	5.335	0.126	2.16E-04	1.11	6.72E-03	ADT
TP53BP1	5.886	0.203	6.081	0.169	2.15E-04	-1.14	6.73E-03	ADT
SOGA2	4.969	0.085	4.88	0.097	2.17E-04	1.06	6.73E-03	ADT
APOO	4.809	0.293	5.084	0.218	2.16E-04	-1.21	6.73E-03	ADT
KCNK13	5.788	0.167	5.604	0.214	2.17E-04	1.14	6.73E-03	ADT
EIF2C2	8.886	0.288	9.173	0.281	2.19E-04	-1.22	6.75E-03	ADT
ZBTB49	6.253	0.156	6.417	0.18	2.19E-04	-1.12	6.76E-03	ADT
WDR18	6.187	0.112	6.066	0.137	2.22E-04	1.09	6.82E-03	ADT
TRAPPC5	7.863	0.192	8.082	0.266	2.21E-04	-1.16	6.82E-03	ADT
KLC2	6.11	0.089	6.02	0.09	2.23E-04	1.06	6.85E-03	ADT
PIGS	7.807	0.166	7.974	0.172	2.26E-04	-1.12	6.92E-03	ADT
CIRBP	8.865	0.196	9.067	0.216	2.29E-04	-1.15	7.02E-03	ADT
AVP	6.762	0.165	6.58	0.216	2.30E-04	1.13	7.02E-03	ADT
USP19	7.496	0.103	7.623	0.164	2.32E-04	-1.09	7.05E-03	ADT
MAP6	4.876	0.15	4.724	0.159	2.32E-04	1.11	7.06E-03	ADT
NOVA2	6.229	0.127	6.104	0.122	2.32E-04	1.09	7.06E-03	ADT
CHADL	6.503	0.115	6.39	0.112	2.35E-04	1.08	7.13E-03	ADT
HDAC1	7.761	0.236	7.986	0.197	2.38E-04	-1.17	7.20E-03	ADT
TFDP3	4.056	0.166	3.89	0.169	2.38E-04	1.12	7.20E-03	ADT
PLIN1	5.805	0.136	5.675	0.116	2.40E-04	1.09	7.21E-03	ADT
RABEP2	6.353	0.098	6.258	0.093	2.39E-04	1.07	7.22E-03	ADT
GLTSCR1	7.111	0.138	6.981	0.109	2.40E-04	1.09	7.22E-03	ADT
MAMDC4	6.402	0.104	6.298	0.106	2.44E-04	1.07	7.23E-03	ADT
COPB1	6.732	0.324	7.021	0.178	2.43E-04	-1.22	7.23E-03	ADT
NOTUM	5.901	0.141	5.762	0.137	2.42E-04	1.10	7.23E-03	ADT

RAPGEF3	5.262	0.103	5.16	0.102	2.43E-04	1.07	7.23E-03	ADT
GRIN3B	6.595	0.111	6.484	0.115	2.42E-04	1.08	7.23E-03	ADT
LRRC43	4.675	0.128	4.547	0.131	2.44E-04	1.09	7.23E-03	ADT
COL20A1	6.446	0.133	6.317	0.122	2.44E-04	1.09	7.24E-03	ADT
ZDHHC22	4.74	0.198	4.54	0.208	2.42E-04	1.15	7.24E-03	ADT
FGF5	4.663	0.155	4.818	0.158	2.41E-04	-1.11	7.24E-03	ADT
ZMAT2	7.844	0.276	7.583	0.23	2.46E-04	1.20	7.27E-03	ADT
ETV2	6.347	0.115	6.219	0.154	2.47E-04	1.09	7.28E-03	ADT
APOE	6.318	0.129	6.188	0.136	2.48E-04	1.09	7.31E-03	ADT
DENND1B	5.695	0.257	5.981	0.345	2.48E-04	-1.22	7.32E-03	ADT
CCDC40	5.033	0.139	4.898	0.131	2.51E-04	1.10	7.36E-03	ADT
EDN3	5.816	0.076	5.726	0.115	2.51E-04	1.06	7.37E-03	ADT
RSBN1L	7.214	0.288	7.483	0.228	2.54E-04	-1.20	7.40E-03	ADT
SGK3	5.992	0.252	6.237	0.238	2.53E-04	-1.19	7.40E-03	ADT
AGBL1	4.087	0.083	3.997	0.105	2.53E-04	1.06	7.40E-03	ADT
CABP2	6.899	0.151	6.757	0.124	2.55E-04	1.10	7.44E-03	ADT
ZFP36L1	9.501	0.145	9.317	0.248	2.56E-04	1.14	7.45E-03	ADT
TBC1D20	7.619	0.154	7.761	0.112	2.58E-04	-1.10	7.48E-03	ADT
MXD3	7.919	0.15	7.753	0.201	2.58E-04	1.12	7.48E-03	ADT
RPL36	7.17	0.287	6.887	0.289	2.60E-04	1.22	7.50E-03	ADT
PGD	10.499	0.231	10.752	0.302	2.60E-04	-1.19	7.51E-03	ADT
STRN4	8.363	0.14	8.179	0.253	2.60E-04	1.14	7.51E-03	ADT
HCCS	5.733	0.258	5.979	0.225	2.61E-04	-1.19	7.51E-03	ADT
BUD31	7.727	0.321	8.07	0.398	2.61E-04	-1.27	7.52E-03	ADT
PLEKHA2	7.34	0.166	7.515	0.201	2.63E-04	-1.13	7.54E-03	ADT
VEGFC	5.229	0.131	5.101	0.126	2.66E-04	1.09	7.60E-03	ADT
ALKBH7	5.025	0.126	4.899	0.131	2.65E-04	1.09	7.61E-03	ADT
RPL27A	7.053	0.3	7.329	0.222	2.67E-04	-1.21	7.62E-03	ADT
MTRNR2L6	12.887	0.158	13.038	0.143	2.68E-04	-1.11	7.63E-03	ADT
APBA1	4.967	0.129	4.847	0.105	2.69E-04	1.09	7.66E-03	ADT
FSTL3	5.615	0.137	5.48	0.138	2.69E-04	1.10	7.66E-03	ADT
IL37	3.861	0.143	3.718	0.151	2.71E-04	1.10	7.66E-03	ADT
TMEM9B	7.278	0.248	7.521	0.243	2.71E-04	-1.18	7.67E-03	ADT
PIGV	6.699	0.185	6.873	0.154	2.70E-04	-1.13	7.67E-03	ADT
LOC100506422	3.868	0.482	3.407	0.435	2.73E-04	1.38	7.70E-03	ADT
TMEM196	3.834	0.281	4.103	0.255	2.74E-04	-1.20	7.70E-03	ADT
HES7	4.915	0.151	4.763	0.161	2.73E-04	1.11	7.71E-03	ADT
NPEPL1	8.586	0.119	8.466	0.129	2.73E-04	1.09	7.71E-03	ADT
PITPNB	6.909	0.253	7.14	0.179	2.76E-04	-1.17	7.73E-03	ADT
IKZF5	6.682	0.241	6.914	0.225	2.78E-04	-1.17	7.74E-03	ADT
PVRL3	3.68	0.107	3.797	0.142	2.76E-04	-1.08	7.74E-03	ADT
ANGEL2	5.258	0.253	5.503	0.239	2.77E-04	-1.19	7.75E-03	ADT
ARF1	9.813	0.115	9.931	0.132	2.76E-04	-1.09	7.75E-03	ADT
RAB3IL1	5.549	0.151	5.398	0.158	2.78E-04	1.11	7.75E-03	ADT
ATE1	6.444	0.333	6.75	0.251	2.79E-04	-1.24	7.77E-03	ADT
ADSSL1	5.607	0.096	5.514	0.094	2.81E-04	1.07	7.79E-03	ADT
DNAJB4	3.117	0.243	3.367	0.277	2.81E-04	-1.19	7.80E-03	ADT

NACA	6.852	0.267	6.595	0.251	2.83E-04	1.19	7.82E-03	ADT
PGBD2	4.115	0.2	4.324	0.239	2.84E-04	-1.16	7.83E-03	ADT
FAM100B	9.315	0.167	9.117	0.257	2.83E-04	1.15	7.83E-03	ADT
ANAPC13	6.97	0.302	7.258	0.272	2.85E-04	-1.22	7.85E-03	ADT
ATL3	5.645	0.256	5.888	0.227	2.87E-04	-1.18	7.89E-03	ADT
COPS6	8.073	0.207	8.298	0.272	2.88E-04	-1.17	7.89E-03	ADT
RAP2B	6.502	0.151	6.647	0.141	2.88E-04	-1.11	7.90E-03	ADT
DDX23	7.958	0.18	8.124	0.14	2.90E-04	-1.12	7.90E-03	ADT
TRAF6	8.208	0.207	8.393	0.134	2.89E-04	-1.14	7.92E-03	ADT
CDH15	5.662	0.136	5.525	0.148	2.90E-04	1.10	7.92E-03	ADT
POLD1	6.158	0.082	6.078	0.084	2.91E-04	1.06	7.92E-03	ADT
AVL9	7.201	0.261	7.437	0.182	2.92E-04	-1.18	7.95E-03	ADT
PITHD1	5.532	0.237	5.764	0.236	2.93E-04	-1.17	7.95E-03	ADT
LRRC25	9.828	0.185	10.033	0.254	2.96E-04	-1.15	8.03E-03	ADT
GLT8D1	5.951	0.218	6.155	0.187	2.98E-04	-1.15	8.08E-03	ADT
EXOC4	6.504	0.171	6.685	0.213	2.99E-04	-1.13	8.09E-03	ADT
MYOCD	3.858	0.128	3.74	0.103	3.00E-04	1.09	8.09E-03	ADT
NHSL2	9.388	0.165	9.222	0.182	3.00E-04	1.12	8.10E-03	ADT
CCNJL	9.211	0.212	8.972	0.301	3.03E-04	1.18	8.14E-03	ADT
LYNX1	5.555	0.11	5.452	0.091	3.05E-04	1.07	8.18E-03	ADT
GGN	6.488	0.138	6.352	0.145	3.07E-04	1.10	8.23E-03	ADT
CARD9	6.247	0.088	6.129	0.166	3.07E-04	1.09	8.23E-03	ADT
NANOGNB	3.186	0.158	3.374	0.248	3.08E-04	-1.14	8.24E-03	ADT
NSMCE2	4.983	0.279	5.278	0.347	3.09E-04	-1.23	8.26E-03	ADT
CCDC90B	3.129	0.701	3.865	0.861	3.11E-04	-1.67	8.28E-03	ADT
BRK1	5.906	0.235	6.124	0.193	3.12E-04	-1.16	8.31E-03	ADT
COG4	6.521	0.269	6.772	0.229	3.13E-04	-1.19	8.33E-03	ADT
ORC3	6.021	0.427	6.424	0.38	3.14E-04	-1.32	8.35E-03	ADT
LHX6	6.196	0.124	6.079	0.112	3.15E-04	1.08	8.35E-03	ADT
COMMD2	5.553	0.208	5.746	0.172	3.16E-04	-1.14	8.36E-03	ADT
ASCC1	4.97	0.283	5.236	0.248	3.16E-04	-1.20	8.37E-03	ADT
G3BP1	7.139	0.356	7.486	0.36	3.18E-04	-1.27	8.38E-03	ADT
SNX2	8.161	0.465	8.576	0.308	3.18E-04	-1.33	8.39E-03	ADT
STT3A	6.96	0.308	7.265	0.326	3.22E-04	-1.24	8.45E-03	ADT
HIC1	6.662	0.159	6.506	0.166	3.21E-04	1.11	8.46E-03	ADT
SERPINA5	4.219	0.146	4.081	0.131	3.22E-04	1.10	8.46E-03	ADT
SPRR1A	5.565	0.218	5.347	0.237	3.24E-04	1.16	8.48E-03	ADT
FAM170B	6.039	0.152	5.892	0.152	3.24E-04	1.11	8.48E-03	ADT
APOA1	5.684	0.129	5.551	0.153	3.25E-04	1.10	8.49E-03	ADT
CSNK1G2	9.449	0.149	9.601	0.171	3.28E-04	-1.11	8.54E-03	ADT
DYNC1LI2	6.391	0.251	6.611	0.151	3.30E-04	-1.16	8.59E-03	ADT
PDPK1	9.306	0.138	9.466	0.21	3.31E-04	-1.12	8.59E-03	ADT
TBL2	5.96	0.136	6.115	0.2	3.31E-04	-1.11	8.60E-03	ADT
OTUD6A	4.963	0.373	4.616	0.322	3.31E-04	1.27	8.60E-03	ADT
HS3ST6	6.625	0.2	6.43	0.203	3.34E-04	1.14	8.64E-03	ADT
SCN4A	4.618	0.101	4.524	0.088	3.37E-04	1.07	8.71E-03	ADT
COX6B2	5.44	0.142	5.299	0.152	3.39E-04	1.10	8.71E-03	ADT

COL5A1	5.592	0.123	5.475	0.112	3.39E-04	1.08	8.71E-03	ADT
SLC9A6	5.761	0.237	5.975	0.176	3.37E-04	-1.16	8.71E-03	ADT
ZFR2	5.999	0.132	5.87	0.136	3.38E-04	1.09	8.71E-03	ADT
ZNF575	5.972	0.124	5.849	0.134	3.40E-04	1.09	8.72E-03	ADT
S1PR2	5.44	0.135	5.31	0.133	3.41E-04	1.09	8.75E-03	ADT
FICD	5.572	0.226	5.791	0.227	3.43E-04	-1.16	8.78E-03	ADT
PRB4	5.421	0.15	5.279	0.139	3.44E-04	1.10	8.78E-03	ADT
OR5M10	2.798	0.355	3.16	0.415	3.46E-04	-1.29	8.83E-03	ADT
TLX3	4.989	0.251	4.744	0.261	3.47E-04	1.19	8.83E-03	ADT
HMOX2	7.161	0.225	7.367	0.186	3.50E-04	-1.15	8.91E-03	ADT
SLC17A5	5.59	0.211	5.779	0.155	3.51E-04	-1.14	8.91E-03	ADT
GCLM	3.896	0.158	4.133	0.356	3.52E-04	-1.18	8.92E-03	ADT
ROBO4	5.603	0.111	5.497	0.106	3.53E-04	1.08	8.93E-03	ADT
VPS18	6.836	0.158	7.022	0.249	3.53E-04	-1.14	8.93E-03	ADT
WNK2	6.052	0.115	5.945	0.105	3.54E-04	1.08	8.95E-03	ADT
C19orf12	7.433	0.232	7.213	0.219	3.56E-04	1.16	8.97E-03	ADT
DDOST	6.927	0.197	7.121	0.212	3.58E-04	-1.14	9.01E-03	ADT
SRP19	7.039	0.243	7.263	0.206	3.59E-04	-1.17	9.02E-03	ADT
CACNA1F	5.104	0.099	5.014	0.08	3.61E-04	1.06	9.07E-03	ADT
ACTR8	5.722	0.292	5.993	0.259	3.62E-04	-1.21	9.08E-03	ADT
UBE2L3	7.896	0.174	8.068	0.188	3.63E-04	-1.13	9.09E-03	ADT
RNF6	5.59	0.227	5.797	0.183	3.64E-04	-1.15	9.11E-03	ADT
CCDC86	5.753	0.116	5.633	0.143	3.67E-04	1.09	9.13E-03	ADT
FGR	10.265	0.161	10.45	0.243	3.66E-04	-1.14	9.13E-03	ADT
SDF2	8.506	0.449	8.93	0.422	3.66E-04	-1.34	9.13E-03	ADT
FDX1L	6.359	0.16	6.212	0.134	3.68E-04	1.11	9.14E-03	ADT
GLIS2	6.509	0.183	6.338	0.162	3.69E-04	1.13	9.15E-03	ADT
GALR3	7.46	0.132	7.324	0.159	3.70E-04	1.10	9.15E-03	ADT
LAMC3	6.259	0.139	6.133	0.11	3.69E-04	1.09	9.16E-03	ADT
GPBP1L1	8.02	0.159	8.195	0.223	3.70E-04	-1.13	9.16E-03	ADT
RBM7	6.453	0.394	6.801	0.268	3.73E-04	-1.27	9.20E-03	ADT
ATP2B3	5.36	0.13	5.242	0.105	3.74E-04	1.09	9.21E-03	ADT
DESI1	7.41	0.203	7.613	0.23	3.75E-04	-1.15	9.22E-03	ADT
B3GNT6	6.704	0.121	6.583	0.135	3.74E-04	1.09	9.22E-03	ADT
CARNS1	6.511	0.135	6.384	0.128	3.78E-04	1.09	9.27E-03	ADT
COX8A	8.255	0.143	8.394	0.149	3.78E-04	-1.10	9.27E-03	ADT
ZNF238	7.62	0.198	7.437	0.176	3.80E-04	1.14	9.29E-03	ADT
CCDC153	6.509	0.168	6.339	0.197	3.80E-04	1.13	9.29E-03	ADT
SRMS	5.612	0.168	5.455	0.151	3.81E-04	1.11	9.30E-03	ADT
AK8	4.652	0.117	4.538	0.125	3.82E-04	1.08	9.31E-03	ADT
MUL1	6.132	0.141	6.281	0.185	3.85E-04	-1.11	9.34E-03	ADT
ZCRB1	5.076	0.278	5.335	0.253	3.85E-04	-1.20	9.35E-03	ADT
ALOX15B	5.553	0.134	5.435	0.093	3.85E-04	1.09	9.36E-03	ADT
MORF4L1	7.786	0.296	8.045	0.191	3.85E-04	-1.20	9.36E-03	ADT
NCF2	11.332	0.196	11.51	0.159	3.87E-04	-1.13	9.37E-03	ADT
SETBP1	5.211	0.159	5.047	0.194	3.88E-04	1.12	9.39E-03	ADT
NDUFA11	5.965	0.119	5.835	0.164	3.92E-04	1.09	9.46E-03	ADT

PARL	6.616	0.289	6.887	0.275	3.95E-04	-1.21	9.51E-03	ADT
KRTAP1-5	5.881	0.175	5.716	0.169	3.96E-04	1.12	9.52E-03	ADT
SELM	6.518	0.218	6.308	0.222	3.95E-04	1.16	9.52E-03	ADT
MAMSTR	5.674	0.101	5.583	0.083	3.97E-04	1.07	9.54E-03	ADT
HMGN4	7.145	0.197	7.362	0.279	3.99E-04	-1.16	9.57E-03	ADT
SPHK2	6.332	0.096	6.242	0.092	4.01E-04	1.06	9.58E-03	ADT
ITGAD	6.361	0.169	6.199	0.17	4.01E-04	1.12	9.58E-03	ADT
BCL9	6.435	0.163	6.259	0.224	4.00E-04	1.13	9.58E-03	ADT
SELK	6.534	0.309	6.818	0.27	4.05E-04	-1.22	9.67E-03	ADT
FAM83G	5.286	0.159	5.135	0.157	4.09E-04	1.11	9.72E-03	ADT
CBR3	5.396	0.17	5.22	0.214	4.10E-04	1.13	9.72E-03	ADT
MLL3	9.686	0.202	9.512	0.125	4.10E-04	1.13	9.73E-03	ADT
MRPS5	5.893	0.237	6.119	0.237	4.09E-04	-1.17	9.73E-03	ADT
FTSJ1	5.858	0.193	6.065	0.26	4.09E-04	-1.15	9.73E-03	ADT
MTMR14	8.609	0.14	8.759	0.187	4.13E-04	-1.11	9.77E-03	ADT
NMNAT3	4.864	0.128	4.736	0.147	4.14E-04	1.09	9.78E-03	ADT
SYNGR4	5.649	0.143	5.508	0.159	4.14E-04	1.10	9.79E-03	ADT
NSUN2	6.187	0.192	6.393	0.259	4.16E-04	-1.15	9.80E-03	ADT
FGF4	5.859	0.112	5.743	0.141	4.19E-04	1.08	9.87E-03	ADT
NPHP3	5.487	0.272	5.731	0.218	4.21E-04	-1.18	9.90E-03	ADT
KCTD18	6.082	0.248	6.303	0.191	4.22E-04	-1.17	9.92E-03	ADT
FAM19A5	6.27	0.122	6.152	0.128	4.26E-04	1.09	9.99E-03	ADT
COL6A2	6.754	0.129	6.637	0.107	4.29E-04	1.08	1.00E-02	ADT
FAM167A	5.103	0.16	4.959	0.129	4.31E-04	1.10	1.01E-02	ADT
KRT35	4.895	0.122	4.774	0.14	4.31E-04	1.09	1.01E-02	ADT
DCAF5	6.879	0.134	7.002	0.123	4.34E-04	-1.09	1.01E-02	ADT
IFNA2	3.626	0.208	3.405	0.277	4.33E-04	1.17	1.01E-02	ADT
RPUSD3	6.283	0.161	6.446	0.193	4.31E-04	-1.12	1.01E-02	ADT
EMX2	4.969	0.122	4.848	0.138	4.35E-04	1.09	1.01E-02	ADT
PCK1	4.323	0.152	4.172	0.172	4.39E-04	1.11	1.02E-02	ADT
PHACTR3	5.074	0.137	4.948	0.126	4.38E-04	1.09	1.02E-02	ADT
TAF12	6.957	0.182	7.135	0.199	4.41E-04	-1.13	1.02E-02	ADT
PTPN9	5.293	0.263	5.56	0.32	4.44E-04	-1.20	1.02E-02	ADT
UNC119	7.539	0.131	7.674	0.165	4.43E-04	-1.10	1.02E-02	ADT
CLEC7A	8.74	0.353	9.06	0.299	4.43E-04	-1.25	1.02E-02	ADT
FOXC2	7.195	0.117	7.077	0.141	4.43E-04	1.09	1.02E-02	ADT
PROSAPIP1	5.586	0.166	5.428	0.17	4.42E-04	1.12	1.02E-02	ADT
B3GNT3	5.714	0.142	5.575	0.157	4.48E-04	1.10	1.03E-02	ADT
BHLHE23	6.264	0.171	6.107	0.154	4.51E-04	1.11	1.03E-02	ADT
PGBD4	6	0.352	6.321	0.31	4.52E-04	-1.25	1.03E-02	ADT
EDNRB	3.234	0.114	3.128	0.108	4.52E-04	1.08	1.03E-02	ADT
ASH2L	7.93	0.244	8.158	0.235	4.53E-04	-1.17	1.04E-02	ADT
UROC1	5.423	0.15	5.282	0.147	4.57E-04	1.10	1.04E-02	ADT
CXorf49	4.948	0.182	4.79	0.124	4.60E-04	1.12	1.05E-02	ADT
LRRC1	3.348	0.151	3.491	0.153	4.61E-04	-1.10	1.05E-02	ADT
PSAP	10.189	0.112	10.303	0.138	4.62E-04	-1.08	1.05E-02	ADT
GRIP1	3.766	0.103	3.651	0.153	4.62E-04	1.08	1.05E-02	ADT

COMP	5.44	0.115	5.328	0.128	4.64E-04	1.08	1.05E-02	ADT
NUP85	6.233	0.225	6.468	0.293	4.65E-04	-1.18	1.05E-02	ADT
TRAPPC8	6.863	0.26	7.087	0.176	4.67E-04	-1.17	1.05E-02	ADT
MON2	7.257	0.259	7.479	0.167	4.67E-04	-1.17	1.05E-02	ADT
DNMT3B	4.895	0.128	4.778	0.113	4.67E-04	1.08	1.06E-02	ADT
SETD2	9.66	0.115	9.537	0.158	4.69E-04	1.09	1.06E-02	ADT
EXOC3L1	6.096	0.129	5.973	0.132	4.71E-04	1.09	1.06E-02	ADT
TMEM14C	6.353	0.416	6.78	0.525	4.73E-04	-1.34	1.06E-02	ADT
MBD6	9.294	0.211	9.104	0.18	4.74E-04	1.14	1.06E-02	ADT
TMEM88B	7.222	0.172	7.065	0.155	4.80E-04	1.11	1.08E-02	ADT
GTF3C2	6.681	0.176	6.865	0.233	4.82E-04	-1.14	1.08E-02	ADT
PIAS4	6.772	0.094	6.682	0.102	4.84E-04	1.06	1.08E-02	ADT
GRB2	9.38	0.156	9.525	0.149	4.84E-04	-1.11	1.08E-02	ADT
GPR173	5.213	0.186	5.04	0.181	4.86E-04	1.13	1.08E-02	ADT
TLCD1	4.373	0.111	4.258	0.144	4.88E-04	1.08	1.09E-02	ADT
KRTAP10-6	6.455	0.267	6.211	0.244	4.90E-04	1.18	1.09E-02	ADT
PPIB	9.594	0.178	9.753	0.148	4.94E-04	-1.12	1.10E-02	ADT
HAUS3	3.952	0.321	4.245	0.294	4.97E-04	-1.23	1.10E-02	ADT
LOC100144595	5.006	0.183	5.174	0.175	4.97E-04	-1.12	1.10E-02	ADT
KRT14	5.567	0.154	5.43	0.128	4.95E-04	1.10	1.10E-02	ADT
ARPC4-TTLL3	7.678	0.183	7.843	0.16	4.99E-04	-1.12	1.10E-02	ADT
CASKIN1	6.282	0.102	6.188	0.097	4.97E-04	1.07	1.10E-02	ADT
FAM100A	8.617	0.192	8.446	0.158	4.98E-04	1.13	1.10E-02	ADT
MYH7B	5.741	0.131	5.624	0.111	5.01E-04	1.08	1.10E-02	ADT
FAM47B	4.554	0.156	4.384	0.225	5.04E-04	1.13	1.11E-02	ADT
HMGCL	5.819	0.136	5.958	0.173	5.05E-04	-1.10	1.11E-02	ADT
PHYHIP	5.067	0.142	4.925	0.171	5.11E-04	1.10	1.12E-02	ADT
ST6GALNAC1	4.652	0.131	4.528	0.137	5.13E-04	1.09	1.12E-02	ADT
PLAA	6.14	0.171	6.295	0.152	5.19E-04	-1.11	1.14E-02	ADT
MLKL	8.38	0.36	8.7	0.302	5.22E-04	-1.25	1.14E-02	ADT
KLHL36	6.949	0.199	7.149	0.245	5.22E-04	-1.15	1.14E-02	ADT
CHTF8	10.751	0.221	10.518	0.302	5.24E-04	1.18	1.14E-02	ADT
ZSCAN20	4.501	0.168	4.35	0.148	5.24E-04	1.11	1.14E-02	ADT
TMEM199	8.001	0.243	8.222	0.223	5.28E-04	-1.17	1.15E-02	ADT
GAS2L1	5.768	0.132	5.649	0.118	5.28E-04	1.09	1.15E-02	ADT
MED17	5.724	0.319	6	0.232	5.29E-04	-1.21	1.15E-02	ADT
SYCE3	4.782	0.141	4.643	0.166	5.31E-04	1.10	1.15E-02	ADT
MTX1	7.611	0.162	7.8	0.265	5.36E-04	-1.14	1.16E-02	ADT
GOLGA6A	4.497	0.081	4.416	0.1	5.38E-04	1.06	1.16E-02	ADT
GABARAPL1	8.446	0.268	8.203	0.244	5.40E-04	1.18	1.16E-02	ADT
ANKRD37	6.784	0.255	7.048	0.338	5.43E-04	-1.20	1.17E-02	ADT
ME3	5.3	0.101	5.205	0.104	5.40E-04	1.07	1.17E-02	ADT
IGSF22	4.794	0.141	4.67	0.115	5.40E-04	1.09	1.17E-02	ADT
MAGED2	5.245	0.193	5.441	0.245	5.43E-04	-1.15	1.17E-02	ADT
CEND1	6.666	0.192	6.495	0.162	5.42E-04	1.13	1.17E-02	ADT
ITPRIPL1	4.89	0.165	5.04	0.155	5.46E-04	-1.11	1.17E-02	ADT
TFPT	6.441	0.108	6.317	0.173	5.47E-04	1.09	1.17E-02	ADT

PDSS2	3.798	0.168	3.971	0.22	5.47E-04	-1.13	1.17E-02	ADT
GTPBP3	5.688	0.132	5.556	0.163	5.49E-04	1.10	1.17E-02	ADT
NPTN	8.659	0.174	8.81	0.129	5.51E-04	-1.11	1.17E-02	ADT
KCNT1	5.478	0.089	5.386	0.117	5.51E-04	1.07	1.17E-02	ADT
SEC14L5	5.776	0.11	5.67	0.122	5.52E-04	1.08	1.17E-02	ADT
ZDHHC2	6.01	0.214	6.232	0.286	5.56E-04	-1.17	1.18E-02	ADT
NOTCH3	6.113	0.114	6.012	0.095	5.55E-04	1.07	1.18E-02	ADT
KRT6A	5.884	0.139	5.76	0.122	5.58E-04	1.09	1.18E-02	ADT
PIP5KL1	6.018	0.144	5.883	0.15	5.60E-04	1.10	1.19E-02	ADT
FAM73A	4.795	0.233	5.026	0.279	5.61E-04	-1.17	1.19E-02	ADT
DNAAF3	5.626	0.117	5.52	0.11	5.68E-04	1.08	1.20E-02	ADT
EXOC3L4	5.804	0.133	5.68	0.14	5.68E-04	1.09	1.20E-02	ADT
SSR3	5.012	0.338	5.317	0.312	5.71E-04	-1.24	1.20E-02	ADT
SLC26A4	3.201	0.124	3.307	0.091	5.70E-04	-1.08	1.20E-02	ADT
TTC40	5.125	0.091	5.041	0.089	5.73E-04	1.06	1.20E-02	ADT
EME1	4.66	0.112	4.543	0.151	5.75E-04	1.08	1.21E-02	ADT
SYCE1L	5.712	0.216	5.527	0.156	5.73E-04	1.14	1.21E-02	ADT
FAM108B1	5.534	0.218	5.731	0.2	5.78E-04	-1.15	1.21E-02	ADT
DPYSL4	5.716	0.111	5.615	0.108	5.83E-04	1.07	1.22E-02	ADT
NDUFS2	6.706	0.178	6.864	0.155	5.85E-04	-1.12	1.22E-02	ADT
WDR90	6.583	0.099	6.493	0.093	5.84E-04	1.06	1.22E-02	ADT
ENTPD7	5.633	0.208	5.837	0.245	5.87E-04	-1.15	1.22E-02	ADT
RASAL1	5.651	0.141	5.531	0.101	5.88E-04	1.09	1.22E-02	ADT
RILPL2	9.267	0.225	9.464	0.184	5.89E-04	-1.15	1.23E-02	ADT
TACC2	4.312	0.122	4.201	0.118	5.90E-04	1.08	1.23E-02	ADT
TPRN	6.841	0.097	6.747	0.115	5.95E-04	1.07	1.23E-02	ADT
CCDC8	5.627	0.105	5.516	0.147	5.96E-04	1.08	1.24E-02	ADT
FAM20C	7.168	0.156	7.027	0.146	5.98E-04	1.10	1.24E-02	ADT
PRPF4	6.588	0.221	6.792	0.222	6.03E-04	-1.15	1.25E-02	ADT
MYO18B	5.193	0.105	5.097	0.105	6.04E-04	1.07	1.25E-02	ADT
OR5C1	5.446	0.282	5.179	0.308	6.04E-04	1.20	1.25E-02	ADT
MTMR1	7.799	0.18	7.984	0.237	6.08E-04	-1.14	1.25E-02	ADT
SCRIB	6.345	0.097	6.258	0.09	6.08E-04	1.06	1.25E-02	ADT
RAC1	9.084	0.19	9.258	0.188	6.11E-04	-1.13	1.25E-02	ADT
TSKS	5.355	0.155	5.224	0.112	6.10E-04	1.10	1.25E-02	ADT
PLEC	7.481	0.181	7.309	0.198	6.11E-04	1.13	1.25E-02	ADT
TLX1	5.619	0.212	5.434	0.173	6.13E-04	1.14	1.25E-02	ADT
CD276	5.755	0.09	5.663	0.117	6.14E-04	1.07	1.26E-02	ADT
DAB2IP	5.561	0.114	5.459	0.107	6.12E-04	1.07	1.26E-02	ADT
ZDHHC7	7.962	0.126	7.837	0.152	6.17E-04	1.09	1.26E-02	ADT
TIPARP	6.775	0.164	6.936	0.196	6.26E-04	-1.12	1.26E-02	ADT
ZNF512B	5.635	0.131	5.517	0.123	6.25E-04	1.09	1.26E-02	ADT
ANKRD13B	5.941	0.14	5.815	0.13	6.19E-04	1.09	1.26E-02	ADT
THAP2	3.212	0.109	3.332	0.167	6.25E-04	-1.09	1.26E-02	ADT
MAP3K11	8.737	0.138	8.611	0.137	6.27E-04	1.09	1.26E-02	ADT
HMX1	6.655	0.128	6.537	0.128	6.24E-04	1.09	1.26E-02	ADT
AEBP1	5.857	0.132	5.736	0.13	6.25E-04	1.09	1.26E-02	ADT

NKX2-3	5.605	0.145	5.472	0.144	6.22E-04	1.10	1.26E-02	ADT
TRPC4AP	9.388	0.134	9.502	0.096	6.22E-04	-1.08	1.27E-02	ADT
GDAP1L1	5.323	0.127	5.201	0.146	6.21E-04	1.09	1.27E-02	ADT
PITX1	5.948	0.18	5.783	0.178	6.24E-04	1.12	1.27E-02	ADT
DUX4L4	6.944	0.147	6.814	0.127	6.24E-04	1.09	1.27E-02	ADT
TPSB2	6.425	0.299	6.129	0.367	6.34E-04	1.23	1.28E-02	ADT
FAHD1	3.456	0.199	3.643	0.214	6.37E-04	-1.14	1.28E-02	ADT
FBXO42	7.085	0.259	7.308	0.205	6.41E-04	-1.17	1.29E-02	ADT
OR51B6	2.533	0.194	2.725	0.239	6.43E-04	-1.14	1.29E-02	ADT
PEX26	6.5	0.118	6.619	0.153	6.46E-04	-1.09	1.29E-02	ADT
RIMBP3	5.162	0.097	5.065	0.122	6.46E-04	1.07	1.29E-02	ADT
SALL3	5.556	0.125	5.442	0.124	6.53E-04	1.08	1.31E-02	ADT
ERP29	6.579	0.39	6.924	0.348	6.56E-04	-1.27	1.31E-02	ADT
ZCCHC8	5.935	0.237	6.146	0.215	6.55E-04	-1.16	1.31E-02	ADT
STARD3	8.123	0.108	8.231	0.136	6.57E-04	-1.08	1.31E-02	ADT
FAM188B	5.943	0.288	5.66	0.351	6.58E-04	1.22	1.31E-02	ADT
SAR1A	8.212	0.212	8.388	0.134	6.60E-04	-1.13	1.31E-02	ADT
TSNARE1	5.629	0.121	5.513	0.139	6.59E-04	1.08	1.31E-02	ADT
C19orf76	5.02	0.254	4.802	0.201	6.65E-04	1.16	1.32E-02	ADT
DDN	5.798	0.144	5.672	0.121	6.66E-04	1.09	1.32E-02	ADT
FAM69C	5.06	0.182	4.9	0.159	6.65E-04	1.12	1.32E-02	ADT
NCOA2	9.922	0.173	9.77	0.153	6.71E-04	1.11	1.33E-02	ADT
NAGPA	5.83	0.238	5.621	0.209	6.76E-04	1.16	1.33E-02	ADT
RBM6	6.446	0.173	6.632	0.257	6.75E-04	-1.14	1.33E-02	ADT
MARCH2	6.701	0.129	6.847	0.207	6.77E-04	-1.11	1.33E-02	ADT
C8orf44-SGK3	8.125	0.441	7.702	0.51	6.76E-04	1.34	1.33E-02	ADT
IGLL1	7.298	0.13	7.183	0.12	6.74E-04	1.08	1.33E-02	ADT
NEUROD2	5.726	0.171	5.563	0.194	6.80E-04	1.12	1.33E-02	ADT
RHOB	7.566	0.134	7.714	0.208	6.81E-04	-1.11	1.33E-02	ADT
CSF3	6.288	0.125	6.174	0.122	6.79E-04	1.08	1.33E-02	ADT
ARID1A	8.848	0.109	8.753	0.093	6.92E-04	1.07	1.35E-02	ADT
ZC3H4	7.412	0.105	7.313	0.117	6.89E-04	1.07	1.35E-02	ADT
TH	6.214	0.124	6.108	0.101	6.93E-04	1.08	1.35E-02	ADT
XPR1	6.124	0.194	6.293	0.164	6.91E-04	-1.12	1.35E-02	ADT
WDR45L	7.401	0.198	7.569	0.145	6.92E-04	-1.12	1.35E-02	ADT
KLK1	5.94	0.145	5.803	0.162	6.91E-04	1.10	1.35E-02	ADT
FOXD4	5.56	0.152	5.412	0.183	6.90E-04	1.11	1.35E-02	ADT
VPS25	6.785	0.23	6.996	0.237	6.96E-04	-1.16	1.35E-02	ADT
UBR7	4.48	0.26	4.699	0.19	6.97E-04	-1.16	1.35E-02	ADT
ADRA2C	6.552	0.176	6.399	0.152	7.00E-04	1.11	1.36E-02	ADT
TSPAN9	5.133	0.139	5.017	0.097	7.02E-04	1.08	1.36E-02	ADT
CNPY3	9.461	0.11	9.584	0.175	7.04E-04	-1.09	1.36E-02	ADT
SAMD10	6.369	0.178	6.204	0.188	7.06E-04	1.12	1.36E-02	ADT
ASS1	5.009	0.118	4.9	0.124	7.05E-04	1.08	1.36E-02	ADT
TMPRSS13	5.076	0.137	4.958	0.113	7.06E-04	1.09	1.36E-02	ADT
ADAMTSL1	4.613	0.105	4.52	0.098	7.10E-04	1.07	1.36E-02	ADT
UEVLD	6.039	0.303	6.296	0.234	7.09E-04	-1.19	1.37E-02	ADT

TPPP2	5.058	0.159	4.916	0.153	7.12E-04	1.10	1.37E-02	ADT
TAF8	7.851	0.171	8.006	0.171	7.18E-04	-1.11	1.38E-02	ADT
CDON	3.598	0.091	3.511	0.107	7.19E-04	1.06	1.38E-02	ADT
SHOX	5.33	0.13	5.209	0.141	7.24E-04	1.09	1.39E-02	ADT
SWSAP1	5.034	0.198	5.226	0.24	7.31E-04	-1.14	1.40E-02	ADT
TJP3	6.034	0.082	5.953	0.103	7.36E-04	1.06	1.41E-02	ADT
ERGIC3	7.256	0.254	7.502	0.304	7.41E-04	-1.19	1.41E-02	ADT
DHX16	6.899	0.144	7.036	0.167	7.43E-04	-1.10	1.42E-02	ADT
RIN3	8.732	0.171	8.574	0.183	7.44E-04	1.12	1.42E-02	ADT
ZNF180	5.717	0.203	5.894	0.178	7.50E-04	-1.13	1.43E-02	ADT
BCAT2	5.254	0.155	5.112	0.162	7.53E-04	1.10	1.43E-02	ADT
R3HDML	4.836	0.129	4.703	0.18	7.55E-04	1.10	1.43E-02	ADT
TMEM71	9.833	0.199	10.008	0.184	7.58E-04	-1.13	1.43E-02	ADT
SPAST	7.406	0.309	7.661	0.209	7.57E-04	-1.19	1.44E-02	ADT
SCAMP1	5.962	0.279	6.212	0.272	7.62E-04	-1.19	1.44E-02	ADT
CREG1	6.781	0.276	7.012	0.198	7.64E-04	-1.17	1.44E-02	ADT
BTBD1	6.407	0.24	6.615	0.21	7.64E-04	-1.16	1.44E-02	ADT
PPFIA3	5.382	0.106	5.28	0.128	7.58E-04	1.07	1.44E-02	ADT
FADS3	6.307	0.149	6.173	0.149	7.60E-04	1.10	1.44E-02	ADT
UBTD1	7.464	0.196	7.298	0.15	7.63E-04	1.12	1.44E-02	ADT
BAX	8.052	0.207	8.236	0.196	7.62E-04	-1.14	1.44E-02	ADT
FBXL15	5.128	0.119	5.024	0.108	7.61E-04	1.07	1.44E-02	ADT
MEGF8	5.959	0.081	5.885	0.085	7.68E-04	1.05	1.44E-02	ADT
ALG6	4.853	0.318	5.124	0.261	7.69E-04	-1.21	1.44E-02	ADT
RNF216	7.42	0.173	7.589	0.215	7.71E-04	-1.12	1.44E-02	ADT
EMC4	8.042	0.222	8.248	0.244	7.73E-04	-1.15	1.45E-02	ADT
FTHL17	5.316	0.207	5.126	0.217	7.74E-04	1.14	1.45E-02	ADT
INIP	6.601	0.267	6.832	0.229	7.76E-04	-1.17	1.45E-02	ADT
GNL3L	6.802	0.179	6.958	0.158	7.79E-04	-1.11	1.45E-02	ADT
DHRS7C	4.611	0.123	4.486	0.165	7.81E-04	1.09	1.45E-02	ADT
SIX6	6.355	0.236	6.152	0.203	7.82E-04	1.15	1.45E-02	ADT
SH2D3A	6.263	0.15	6.125	0.158	7.86E-04	1.10	1.46E-02	ADT
HCN2	6.741	0.121	6.634	0.114	7.87E-04	1.08	1.46E-02	ADT
ZNF735	2.644	0.392	2.268	0.467	7.89E-04	1.30	1.46E-02	ADT
MYT1	4.74	0.127	4.625	0.132	7.91E-04	1.08	1.47E-02	ADT
ZMYM6	6.367	0.285	6.621	0.278	7.93E-04	-1.19	1.47E-02	ADT
VASH1	5.624	0.121	5.514	0.126	7.94E-04	1.08	1.47E-02	ADT
LYAR	4.533	0.228	4.747	0.258	7.97E-04	-1.16	1.47E-02	ADT
FANCD2	5.187	0.253	5.442	0.338	7.99E-04	-1.19	1.47E-02	ADT
RBM12B	5.266	0.193	5.444	0.209	8.01E-04	-1.13	1.47E-02	ADT
BCAR1	6.704	0.119	6.596	0.123	8.01E-04	1.08	1.47E-02	ADT
MAP1A	4.488	0.096	4.383	0.152	8.00E-04	1.08	1.47E-02	ADT
ECSIT	5.858	0.139	5.739	0.117	8.06E-04	1.09	1.47E-02	ADT
LPIN3	5.395	0.098	5.304	0.109	8.06E-04	1.07	1.48E-02	ADT
SOX30	4.256	0.154	4.125	0.126	8.06E-04	1.10	1.48E-02	ADT
SNRNP40	5.418	0.268	5.669	0.304	8.09E-04	-1.19	1.48E-02	ADT
ATPIF1	5.793	0.181	5.964	0.209	8.05E-04	-1.13	1.48E-02	ADT

HECTD2	3.028	0.088	3.11	0.097	8.05E-04	-1.06	1.48E-02	ADT
OR1L6	1.805	0.186	2.021	0.32	8.16E-04	-1.16	1.48E-02	ADT
CREB3	6.405	0.259	6.619	0.184	8.16E-04	-1.16	1.49E-02	ADT
B3GNTL1	6.586	0.175	6.773	0.264	8.15E-04	-1.14	1.49E-02	ADT
TRIP4	6.212	0.205	6.391	0.186	8.19E-04	-1.13	1.49E-02	ADT
DRD2	6.115	0.139	5.993	0.131	8.17E-04	1.09	1.49E-02	ADT
NSUN4	6.175	0.503	5.731	0.481	8.18E-04	1.36	1.49E-02	ADT
PLDN	5.606	0.255	5.822	0.207	8.20E-04	-1.16	1.49E-02	ADT
HTRA1	5.217	0.088	5.129	0.116	8.15E-04	1.06	1.49E-02	ADT
SLC39A7	6.855	0.175	7.034	0.244	8.24E-04	-1.13	1.49E-02	ADT
CCDC48	7.366	0.13	7.249	0.131	8.29E-04	1.08	1.49E-02	ADT
NAV2	4.397	0.101	4.309	0.092	8.31E-04	1.06	1.50E-02	ADT
PPP1R1A	5.932	0.13	5.808	0.156	8.29E-04	1.09	1.50E-02	ADT
CDX1	5.954	0.155	5.82	0.137	8.27E-04	1.10	1.50E-02	ADT
SECTM1	8.982	0.212	8.765	0.295	8.28E-04	1.16	1.50E-02	ADT
GDF6	4.802	0.16	4.649	0.191	8.32E-04	1.11	1.50E-02	ADT
NUP37	4.007	0.226	4.218	0.254	8.35E-04	-1.16	1.50E-02	ADT
MMP12	2.517	0.162	2.663	0.165	8.37E-04	-1.11	1.50E-02	ADT
PRPF40B	5.409	0.12	5.302	0.118	8.36E-04	1.08	1.50E-02	ADT
PSMB6	8.009	0.317	8.282	0.28	8.39E-04	-1.21	1.50E-02	ADT
HDAC6	6.422	0.202	6.603	0.205	8.48E-04	-1.13	1.52E-02	ADT
MGST2	5.755	0.288	6.008	0.272	8.50E-04	-1.19	1.52E-02	ADT
LEAP2	4.832	0.201	5.002	0.162	8.54E-04	-1.13	1.53E-02	ADT
GTF2B	7.257	0.203	7.433	0.184	8.56E-04	-1.13	1.53E-02	ADT
TMEM126B	5.452	0.267	5.7	0.299	8.61E-04	-1.19	1.54E-02	ADT
TLN2	4.395	0.107	4.306	0.078	8.66E-04	1.06	1.54E-02	ADT
KRT4	5.321	0.126	5.206	0.133	8.65E-04	1.08	1.54E-02	ADT
F2	4.92	0.117	4.811	0.133	8.71E-04	1.08	1.55E-02	ADT
AP5M1	5.581	0.277	5.816	0.232	8.71E-04	-1.18	1.55E-02	ADT
UCK1	7.625	0.162	7.767	0.153	8.73E-04	-1.10	1.55E-02	ADT
PFKM	4.838	0.222	5.083	0.358	8.76E-04	-1.19	1.55E-02	ADT
DMRT1	5.105	0.133	4.991	0.116	8.79E-04	1.08	1.56E-02	ADT
PGF	5.83	0.194	5.666	0.158	8.80E-04	1.12	1.56E-02	ADT
LRP5	5.604	0.105	5.511	0.103	8.82E-04	1.07	1.56E-02	ADT
ALYREF	7.501	0.267	7.763	0.347	8.91E-04	-1.20	1.57E-02	ADT
KLHDC3	7.224	0.179	7.436	0.323	8.92E-04	-1.16	1.57E-02	ADT
SYNGR1	5.527	0.146	5.389	0.173	8.90E-04	1.10	1.57E-02	ADT
CILP2	5.911	0.13	5.801	0.112	8.94E-04	1.08	1.57E-02	ADT
TST	7.855	0.274	8.129	0.369	8.98E-04	-1.21	1.57E-02	ADT
LRRC32	5.665	0.11	5.544	0.177	9.00E-04	1.09	1.58E-02	ADT
NKAIN4	5.135	0.178	4.976	0.183	8.98E-04	1.12	1.58E-02	ADT
ATP5SL	5.456	0.194	5.646	0.251	9.01E-04	-1.14	1.58E-02	ADT
SMURF1	6.854	0.187	7.011	0.153	8.98E-04	-1.11	1.58E-02	ADT
MUC5B	6.131	0.183	5.967	0.187	8.97E-04	1.12	1.58E-02	ADT
TSPYL1	7.029	0.126	7.148	0.15	9.05E-04	-1.09	1.58E-02	ADT
MUT	4.717	0.126	4.84	0.162	9.09E-04	-1.09	1.59E-02	ADT
OR3A3	5.523	0.221	5.272	0.375	9.15E-04	1.19	1.59E-02	ADT

NPB	6.756	0.15	6.628	0.128	9.14E-04	1.09	1.59E-02	ADT
EIF1AD	6.708	0.161	6.847	0.149	9.17E-04	-1.10	1.60E-02	ADT
DOT1L	6.404	0.182	6.249	0.156	9.18E-04	1.11	1.60E-02	ADT
GNAI3	8.31	0.3	8.558	0.228	9.21E-04	-1.19	1.60E-02	ADT
DUSP1	11.423	0.21	11.625	0.259	9.22E-04	-1.15	1.60E-02	ADT
POU3F1	6.69	0.195	6.52	0.184	9.19E-04	1.13	1.60E-02	ADT
UNCX	8.215	0.142	8.093	0.128	9.20E-04	1.09	1.60E-02	ADT
KDELR2	6.711	0.254	6.923	0.202	9.24E-04	-1.16	1.60E-02	ADT
DCTN2	7.245	0.174	7.414	0.222	9.26E-04	-1.12	1.60E-02	ADT
ECHDC2	5.471	0.129	5.632	0.253	9.30E-04	-1.12	1.61E-02	ADT
GMPPA	6.244	0.141	6.385	0.192	9.31E-04	-1.10	1.61E-02	ADT
U2AF2	7.626	0.16	7.767	0.156	9.37E-04	-1.10	1.61E-02	ADT
BLOC1S1	7.332	0.173	7.521	0.274	9.35E-04	-1.14	1.61E-02	ADT
EHD2	5.988	0.123	5.886	0.098	9.36E-04	1.07	1.61E-02	ADT
MARCO	5.712	0.147	5.583	0.144	9.35E-04	1.09	1.61E-02	ADT
P4HB	8.176	0.207	8.368	0.235	9.41E-04	-1.14	1.61E-02	ADT
THOP1	6.028	0.108	5.933	0.109	9.40E-04	1.07	1.61E-02	ADT
HAPLN2	5.941	0.117	5.841	0.104	9.39E-04	1.07	1.61E-02	ADT
KRT71	5.1	0.135	4.975	0.154	9.45E-04	1.09	1.62E-02	ADT
GEMIN4	5.281	0.212	5.099	0.193	9.44E-04	1.13	1.62E-02	ADT
PPP2CB	5.067	0.303	5.35	0.354	9.51E-04	-1.22	1.62E-02	ADT
DHTKD1	7.906	0.179	8.054	0.137	9.50E-04	-1.11	1.62E-02	ADT
MIEN1	8.748	0.152	8.946	0.317	9.53E-04	-1.15	1.62E-02	ADT
AAMP	5.663	0.212	5.848	0.203	9.53E-04	-1.14	1.62E-02	ADT
EIF3CL	4.659	0.424	5.161	0.772	9.56E-04	-1.42	1.63E-02	ADT
SLC25A46	6.481	0.314	6.739	0.238	9.61E-04	-1.20	1.63E-02	ADT
GATA6	5.684	0.14	5.563	0.131	9.61E-04	1.09	1.63E-02	ADT
GPN1	4.708	0.349	5.016	0.351	9.68E-04	-1.24	1.64E-02	ADT
GAB4	5.916	0.166	5.779	0.127	9.67E-04	1.10	1.64E-02	ADT
LCE1A	8.299	0.249	8.097	0.174	9.68E-04	1.15	1.64E-02	ADT
C2orf68	8.597	0.113	8.701	0.128	9.67E-04	-1.07	1.64E-02	ADT
GCGR	6.468	0.115	6.37	0.101	9.71E-04	1.07	1.64E-02	ADT
SEC22B	8.07	0.411	8.407	0.309	9.76E-04	-1.26	1.65E-02	ADT
RNF138	6.534	0.45	6.899	0.318	9.79E-04	-1.29	1.65E-02	ADT
NRXN2	5.609	0.114	5.51	0.112	9.78E-04	1.07	1.65E-02	ADT
FUNDC1	5.976	0.262	6.221	0.309	9.81E-04	-1.19	1.65E-02	ADT
C10orf91	5.012	0.217	4.824	0.205	9.82E-04	1.14	1.65E-02	ADT
CHST8	5.664	0.1	5.569	0.12	9.85E-04	1.07	1.66E-02	ADT
TCEB2	7.247	0.225	7.025	0.3	9.87E-04	1.17	1.66E-02	ADT
DUX4	7.04	0.137	6.916	0.147	9.88E-04	1.09	1.66E-02	ADT
KRTAP10-2	6.77	0.177	6.624	0.139	9.91E-04	1.11	1.66E-02	ADT
ZNF621	5.459	0.16	5.622	0.228	9.90E-04	-1.12	1.66E-02	ADT
MC1R	5.795	0.162	5.658	0.145	9.92E-04	1.10	1.66E-02	ADT
TCF3	6.678	0.151	6.55	0.134	9.90E-04	1.09	1.66E-02	ADT
SCXA	6.621	0.283	6.371	0.288	9.95E-04	1.19	1.66E-02	ADT
ACAN	4.882	0.12	4.784	0.091	1.00E-03	1.07	1.67E-02	ADT
FIBP	6.524	0.19	6.693	0.195	1.01E-03	-1.12	1.68E-02	ADT

ATG4A	4.661	0.265	4.9	0.287	1.02E-03	-1.18	1.68E-02	ADT
CWC25	7.555	0.215	7.748	0.228	1.01E-03	-1.14	1.68E-02	ADT
TSSC1	5.721	0.158	5.864	0.172	1.01E-03	-1.10	1.69E-02	ADT
TBX2	5.685	0.114	5.588	0.104	1.01E-03	1.07	1.69E-02	ADT
GPN3	4.708	0.226	4.92	0.271	1.01E-03	-1.16	1.69E-02	ADT
HARS	7.105	0.213	7.28	0.17	1.02E-03	-1.13	1.69E-02	ADT
FAM47A	4.926	0.163	4.79	0.133	1.03E-03	1.10	1.70E-02	ADT
UBB	8.823	0.235	8.616	0.238	1.03E-03	1.15	1.70E-02	ADT
ANXA13	3.233	0.134	3.111	0.148	1.03E-03	1.09	1.70E-02	ADT
SOX3	6.823	0.147	6.694	0.146	1.03E-03	1.09	1.70E-02	ADT
IL28A	5.284	0.157	5.142	0.172	1.03E-03	1.10	1.70E-02	ADT
ICK	5.633	0.215	5.836	0.262	1.03E-03	-1.15	1.70E-02	ADT
LZTS2	6.406	0.12	6.306	0.099	1.03E-03	1.07	1.70E-02	ADT
GPR32	4.566	0.196	4.748	0.231	1.04E-03	-1.13	1.71E-02	ADT
CROCC	6.414	0.093	6.333	0.091	1.04E-03	1.06	1.71E-02	ADT
LOXL2	5.649	0.083	5.576	0.084	1.04E-03	1.05	1.71E-02	ADT
ACO2	6.46	0.258	6.687	0.265	1.05E-03	-1.17	1.72E-02	ADT
ZP1	4.882	0.141	4.765	0.116	1.05E-03	1.08	1.72E-02	ADT
OPN4	6.082	0.123	5.977	0.111	1.05E-03	1.08	1.72E-02	ADT
ZNF500	5.982	0.114	5.876	0.136	1.06E-03	1.08	1.73E-02	ADT
WDR46	5.622	0.203	5.791	0.169	1.06E-03	-1.12	1.73E-02	ADT
SMARCA5	6.347	0.249	6.551	0.195	1.07E-03	-1.15	1.74E-02	ADT
PRKAB1	8.109	0.159	8.249	0.165	1.07E-03	-1.10	1.74E-02	ADT
MRC2	5.773	0.124	5.671	0.099	1.07E-03	1.07	1.74E-02	ADT
CHST12	6.6	0.236	6.811	0.255	1.07E-03	-1.16	1.75E-02	ADT
ARMC3	5.427	0.393	5.044	0.521	1.08E-03	1.30	1.75E-02	ADT
GHSR	4.141	0.222	4.326	0.189	1.08E-03	-1.14	1.75E-02	ADT
PDE4C	5.455	0.091	5.37	0.108	1.08E-03	1.06	1.75E-02	ADT
ZNF580	6.62	0.143	6.492	0.152	1.08E-03	1.09	1.75E-02	ADT
NUP54	4.71	0.305	4.968	0.272	1.08E-03	-1.20	1.75E-02	ADT
NLRP1	9.812	0.221	9.599	0.285	1.09E-03	1.16	1.76E-02	ADT
SYN3	5.53	0.105	5.429	0.133	1.09E-03	1.07	1.76E-02	ADT
QRFP	7.348	0.221	7.159	0.208	1.10E-03	1.14	1.77E-02	ADT
BRPF3	6.888	0.132	6.756	0.185	1.10E-03	1.10	1.77E-02	ADT
NKX2-1	4.871	0.129	4.763	0.111	1.10E-03	1.08	1.78E-02	ADT
TXNDC12	7.783	0.221	7.969	0.198	1.10E-03	-1.14	1.78E-02	ADT
MFSD11	6.329	0.199	6.501	0.196	1.11E-03	-1.13	1.78E-02	ADT
IGSF9B	5.162	0.151	5.02	0.187	1.11E-03	1.10	1.78E-02	ADT
CFDP1	5.38	0.227	5.57	0.196	1.11E-03	-1.14	1.78E-02	ADT
MFSD2A	5.314	0.119	5.438	0.18	1.11E-03	-1.09	1.78E-02	ADT
OR4K2	2.795	0.166	2.978	0.276	1.12E-03	-1.14	1.79E-02	ADT
BOK	6.584	0.131	6.474	0.112	1.12E-03	1.08	1.79E-02	ADT
WDR82	7.446	0.285	7.675	0.203	1.12E-03	-1.17	1.79E-02	ADT
SLC25A13	5.964	0.233	6.173	0.256	1.13E-03	-1.16	1.81E-02	ADT
CTR9	6.689	0.211	6.873	0.217	1.13E-03	-1.14	1.81E-02	ADT
PPP1R37	6.239	0.106	6.145	0.111	1.13E-03	1.07	1.81E-02	ADT
PAK3	3.832	0.123	3.724	0.124	1.13E-03	1.08	1.81E-02	ADT

BAK1	6.723	0.215	6.948	0.33	1.14E-03	-1.17	1.81E-02	ADT
RAB26	6.16	0.127	6.05	0.128	1.14E-03	1.08	1.81E-02	ADT
CHCHD3	5.911	0.284	6.169	0.323	1.14E-03	-1.20	1.81E-02	ADT
KCTD19	4.342	0.115	4.244	0.109	1.14E-03	1.07	1.82E-02	ADT
PCYT1A	8.212	0.268	8.423	0.178	1.15E-03	-1.16	1.82E-02	ADT
UQCRH	4.342	0.286	4.592	0.296	1.15E-03	-1.19	1.83E-02	ADT
CD40	4.746	0.193	4.574	0.208	1.15E-03	1.13	1.83E-02	ADT
DUSP11	8.945	0.209	9.123	0.197	1.16E-03	-1.13	1.83E-02	ADT
ETV3	8.468	0.235	8.655	0.165	1.16E-03	-1.14	1.83E-02	ADT
AMELY	4.095	0.174	3.948	0.16	1.17E-03	1.11	1.85E-02	ADT
C20orf43	8.307	0.165	8.449	0.16	1.17E-03	-1.10	1.86E-02	ADT
R3HDM2	7.993	0.153	7.867	0.128	1.18E-03	1.09	1.86E-02	ADT
ITPKC	5.977	0.212	6.175	0.259	1.19E-03	-1.15	1.87E-02	ADT
HIST1H2BO	5.667	0.237	5.895	0.311	1.19E-03	-1.17	1.88E-02	ADT
PPL	6.124	0.224	5.924	0.245	1.20E-03	1.15	1.88E-02	ADT
ING1	7.03	0.158	7.164	0.149	1.20E-03	-1.10	1.89E-02	ADT
BAG4	5.362	0.271	5.587	0.234	1.21E-03	-1.17	1.90E-02	ADT
NOL12	7.592	0.129	7.709	0.15	1.21E-03	-1.08	1.90E-02	ADT
QRICH1	7.728	0.278	7.959	0.241	1.21E-03	-1.17	1.91E-02	ADT
H2AFV	6.992	0.195	7.156	0.177	1.22E-03	-1.12	1.91E-02	ADT
NTN1	6.545	0.12	6.435	0.141	1.22E-03	1.08	1.91E-02	ADT
RIOK2	3.785	0.318	4.079	0.386	1.22E-03	-1.23	1.91E-02	ADT
VPS37B	7.222	0.12	7.112	0.14	1.23E-03	1.08	1.92E-02	ADT
HNRNPR	6.106	0.352	6.411	0.358	1.23E-03	-1.24	1.93E-02	ADT
CALCOCO1	8.879	0.261	9.127	0.335	1.24E-03	-1.19	1.93E-02	ADT
TRMT2B	6.167	0.299	6.428	0.309	1.24E-03	-1.20	1.94E-02	ADT
ZYG11B	6.548	0.253	6.754	0.205	1.25E-03	-1.15	1.94E-02	ADT
SRSF3	7.487	0.296	7.734	0.269	1.25E-03	-1.19	1.94E-02	ADT
KCNMB3	4.627	0.121	4.73	0.116	1.25E-03	-1.07	1.95E-02	ADT
AHNAK2	5.302	0.09	5.228	0.08	1.25E-03	1.05	1.95E-02	ADT
TMX1	5.866	0.328	6.134	0.27	1.26E-03	-1.20	1.95E-02	ADT
HAPLN4	5.375	0.158	5.237	0.168	1.26E-03	1.10	1.95E-02	ADT
E4F1	6.301	0.088	6.207	0.14	1.26E-03	1.07	1.95E-02	ADT
TRAK2	6.382	0.316	6.635	0.236	1.26E-03	-1.19	1.96E-02	ADT
FOXRED2	5.025	0.128	4.911	0.142	1.26E-03	1.08	1.96E-02	ADT
RASL10A	6.114	0.148	5.983	0.162	1.26E-03	1.10	1.96E-02	ADT
SLCO4A1	5.725	0.127	5.62	0.11	1.27E-03	1.08	1.96E-02	ADT
HPS5	5.104	0.278	5.338	0.261	1.27E-03	-1.18	1.96E-02	ADT
CCRL2	5.086	0.325	5.487	0.653	1.28E-03	-1.32	1.97E-02	ADT
SCARF2	6.749	0.124	6.639	0.137	1.28E-03	1.08	1.97E-02	ADT
MYL9	7.025	0.267	6.799	0.255	1.28E-03	1.17	1.97E-02	ADT
PRMT3	4.517	0.257	4.733	0.24	1.28E-03	-1.16	1.97E-02	ADT
PLA2G4D	5.414	0.147	5.296	0.113	1.28E-03	1.09	1.97E-02	ADT
DNAI2	5.084	0.106	4.988	0.123	1.28E-03	1.07	1.97E-02	ADT
ZBTB32	5.596	0.122	5.494	0.112	1.29E-03	1.07	1.98E-02	ADT
U2AF1L4	6.794	0.261	7.028	0.295	1.30E-03	-1.18	1.98E-02	ADT
ANAPC4	5.502	0.351	5.792	0.31	1.30E-03	-1.22	1.99E-02	ADT

GPR150	6.837	0.141	6.715	0.146	1.30E-03	1.09	1.99E-02	ADT
SNF8	6.794	0.209	6.967	0.186	1.30E-03	-1.13	1.99E-02	ADT
TBC1D16	5.505	0.123	5.396	0.134	1.31E-03	1.08	1.99E-02	ADT
CCDC74A	5.104	0.148	4.984	0.121	1.31E-03	1.09	2.00E-02	ADT
DPF1	5.685	0.168	5.553	0.113	1.31E-03	1.10	2.00E-02	ADT
DUSP6	8.695	0.351	9.004	0.384	1.31E-03	-1.24	2.00E-02	ADT
CLK1	8.134	0.268	8.358	0.249	1.31E-03	-1.17	2.00E-02	ADT
CCDC85C	6.737	0.098	6.657	0.082	1.32E-03	1.06	2.00E-02	ADT
SMAD6	2.816	0.327	2.524	0.371	1.32E-03	1.22	2.00E-02	ADT
MRPS17	3.893	0.511	4.341	0.549	1.32E-03	-1.36	2.00E-02	ADT
RAB1B	8.521	0.124	8.643	0.175	1.32E-03	-1.09	2.01E-02	ADT
STK32C	6.115	0.116	6.015	0.121	1.33E-03	1.07	2.02E-02	ADT
MAPK11	5.821	0.167	5.682	0.155	1.33E-03	1.10	2.02E-02	ADT
GAS1	5.635	0.169	5.48	0.203	1.33E-03	1.11	2.02E-02	ADT
MGAT2	4.736	0.18	4.89	0.181	1.34E-03	-1.11	2.02E-02	ADT
RIBC1	4.607	0.161	4.468	0.168	1.34E-03	1.10	2.02E-02	ADT
MFF	7.216	0.197	7.379	0.177	1.34E-03	-1.12	2.02E-02	ADT
DSC2	8.174	0.53	7.685	0.651	1.34E-03	1.40	2.02E-02	ADT
BSX	6.332	0.152	6.202	0.153	1.34E-03	1.09	2.02E-02	ADT
VDAC3	7.263	0.312	7.509	0.224	1.35E-03	-1.19	2.03E-02	ADT
BCKDK	7.179	0.181	7.348	0.228	1.35E-03	-1.12	2.03E-02	ADT
LOC149373	4.06	0.325	3.773	0.359	1.35E-03	1.22	2.03E-02	ADT
RYR1	5.074	0.085	5.003	0.079	1.36E-03	1.05	2.03E-02	ADT
MACROD1	5.879	0.15	5.749	0.156	1.35E-03	1.09	2.03E-02	ADT
HSBP1	6.171	0.303	6.418	0.254	1.36E-03	-1.19	2.03E-02	ADT
SLC46A2	5.633	0.096	5.545	0.114	1.36E-03	1.06	2.03E-02	ADT
MMP25	8.889	0.248	8.685	0.216	1.36E-03	1.15	2.03E-02	ADT
UTS2	2.979	0.569	3.777	1.369	1.36E-03	-1.74	2.03E-02	ADT
ZNF140	4.88	0.323	5.138	0.252	1.36E-03	-1.20	2.03E-02	ADT
SLC36A4	5.709	0.271	5.939	0.266	1.37E-03	-1.17	2.04E-02	ADT
DOC2B	5.515	0.118	5.396	0.175	1.37E-03	1.09	2.04E-02	ADT
GMEB1	6.92	0.26	7.134	0.23	1.37E-03	-1.16	2.04E-02	ADT
FAM40A	6.828	0.181	6.977	0.161	1.38E-03	-1.11	2.05E-02	ADT
PHF23	6.776	0.152	6.914	0.181	1.38E-03	-1.10	2.05E-02	ADT
ASCL1	4.246	0.211	4.077	0.164	1.38E-03	1.12	2.05E-02	ADT
BEND4	4.907	0.133	4.8	0.108	1.39E-03	1.08	2.07E-02	ADT
NANOS1	6.081	0.089	5.994	0.124	1.39E-03	1.06	2.07E-02	ADT
METTL12	5.108	0.3	5.362	0.297	1.40E-03	-1.19	2.07E-02	ADT
HM13	8.328	0.297	8.595	0.346	1.40E-03	-1.20	2.07E-02	ADT
MNX1	6.359	0.125	6.247	0.143	1.41E-03	1.08	2.09E-02	ADT
SFTPD	6.236	0.176	6.092	0.152	1.42E-03	1.10	2.09E-02	ADT
SCARB1	6.07	0.187	5.905	0.209	1.42E-03	1.12	2.09E-02	ADT
SRD5A1	4.896	0.226	5.128	0.347	1.42E-03	-1.17	2.10E-02	ADT
IGFBP4	5.539	0.162	5.408	0.137	1.42E-03	1.10	2.10E-02	ADT
ANO1	4.372	0.082	4.3	0.088	1.43E-03	1.05	2.10E-02	ADT
DMP1	3.868	0.153	3.741	0.143	1.44E-03	1.09	2.11E-02	ADT
ZNF18	6.632	0.231	6.816	0.183	1.44E-03	-1.14	2.12E-02	ADT

MOB2	5.366	0.12	5.257	0.142	1.44E-03	1.08	2.12E-02	ADT
USP33	6.634	0.296	6.865	0.213	1.44E-03	-1.17	2.12E-02	ADT
PACSIN2	9.71	0.179	9.881	0.24	1.45E-03	-1.13	2.12E-02	ADT
BTF3L4	6.989	0.236	7.181	0.204	1.45E-03	-1.14	2.12E-02	ADT
LCMT1	6.121	0.198	6.282	0.17	1.45E-03	-1.12	2.12E-02	ADT
PDHB	5.486	0.226	5.685	0.253	1.45E-03	-1.15	2.12E-02	ADT
NXNL1	7.663	0.167	7.514	0.193	1.45E-03	1.11	2.13E-02	ADT
LARP7	6.706	0.403	7.026	0.313	1.46E-03	-1.25	2.14E-02	ADT
ZNF674	4.55	0.376	4.869	0.375	1.47E-03	-1.25	2.14E-02	ADT
MFN1	6.853	0.317	7.094	0.185	1.47E-03	-1.18	2.14E-02	ADT
C10orf116	5.743	0.148	5.604	0.192	1.47E-03	1.10	2.14E-02	ADT
AIG1	5.974	0.379	6.296	0.384	1.46E-03	-1.25	2.14E-02	ADT
GTSE1	4.706	0.111	4.609	0.121	1.46E-03	1.07	2.14E-02	ADT
FLRT1	5.153	0.16	5.001	0.214	1.47E-03	1.11	2.14E-02	ADT
PPP2R5D	6.261	0.132	6.393	0.195	1.48E-03	-1.10	2.15E-02	ADT
C20orf96	3.843	0.143	3.712	0.177	1.48E-03	1.10	2.15E-02	ADT
POU2F2	6.883	0.174	6.738	0.166	1.48E-03	1.11	2.15E-02	ADT
SIKE1	5.879	0.315	6.133	0.267	1.49E-03	-1.19	2.15E-02	ADT
CCDC154	5.938	0.149	5.819	0.118	1.49E-03	1.09	2.15E-02	ADT
KRTAP10-8	6.885	0.141	6.767	0.136	1.49E-03	1.09	2.15E-02	ADT
HINFP	6.517	0.184	6.678	0.206	1.50E-03	-1.12	2.16E-02	ADT
FXYD4	4.645	0.15	4.503	0.198	1.50E-03	1.10	2.16E-02	ADT
SALL2	4.387	0.091	4.297	0.131	1.51E-03	1.06	2.17E-02	ADT
PARK7	7.233	0.245	7.433	0.218	1.51E-03	-1.15	2.18E-02	ADT
PLEKHA4	6.299	0.125	6.2	0.102	1.51E-03	1.07	2.18E-02	ADT
NR5A1	5.279	0.175	5.136	0.155	1.52E-03	1.10	2.18E-02	ADT
SPRY3	5.377	0.248	5.173	0.229	1.51E-03	1.15	2.18E-02	ADT
PPP2R1B	5.78	0.337	6.048	0.272	1.52E-03	-1.20	2.18E-02	ADT
PDZRN4	4.792	0.118	4.695	0.106	1.51E-03	1.07	2.18E-02	ADT
CT47A6	7.175	0.335	6.872	0.404	1.52E-03	1.23	2.18E-02	ADT
PMF1-BGLAP	8.092	0.269	8.31	0.236	1.53E-03	-1.16	2.19E-02	ADT
ROR2	5.063	0.129	4.94	0.174	1.53E-03	1.09	2.19E-02	ADT
CACNA1A	5.217	0.089	5.138	0.102	1.53E-03	1.06	2.19E-02	ADT
FAM220A	5.949	0.206	6.129	0.228	1.53E-03	-1.13	2.19E-02	ADT
LOC100130880	3.493	0.282	3.737	0.305	1.53E-03	-1.18	2.19E-02	ADT
CHAT	5.581	0.122	5.483	0.102	1.54E-03	1.07	2.19E-02	ADT
SCGB1C1	6.206	0.179	6.061	0.158	1.53E-03	1.11	2.19E-02	ADT
MRPL47	4.932	0.228	5.132	0.254	1.54E-03	-1.15	2.19E-02	ADT
NLRP6	8.187	0.359	7.9	0.296	1.54E-03	1.22	2.19E-02	ADT
IP6K1	7.278	0.2	7.441	0.179	1.54E-03	-1.12	2.20E-02	ADT
RNF115	5.514	0.312	5.793	0.365	1.55E-03	-1.21	2.20E-02	ADT
ENHO	6.042	0.165	5.911	0.137	1.55E-03	1.10	2.20E-02	ADT
YJEFN3	7.344	0.121	7.246	0.11	1.55E-03	1.07	2.20E-02	ADT
FAM166A	9.178	0.242	8.958	0.299	1.55E-03	1.16	2.21E-02	ADT
PAX5	6.493	0.323	6.166	0.494	1.56E-03	1.25	2.21E-02	ADT
RAE1	6.372	0.182	6.523	0.172	1.56E-03	-1.11	2.22E-02	ADT
KIAA0319L	7.621	0.223	7.814	0.24	1.57E-03	-1.14	2.22E-02	ADT

RS1	4.054	0.186	3.893	0.203	1.58E-03	1.12	2.22E-02	ADT
FAM200A	3.701	0.245	3.914	0.269	1.58E-03	-1.16	2.22E-02	ADT
THNSL2	5.53	0.162	5.707	0.282	1.58E-03	-1.13	2.22E-02	ADT
USP2	5.009	0.114	4.914	0.111	1.57E-03	1.07	2.22E-02	ADT
MCM9	6.788	0.255	6.997	0.234	1.58E-03	-1.16	2.22E-02	ADT
MTDH	6.374	0.195	6.544	0.217	1.57E-03	-1.13	2.22E-02	ADT
MED20	5.681	0.165	5.83	0.201	1.57E-03	-1.11	2.22E-02	ADT
TMEM132E	5.691	0.132	5.574	0.15	1.57E-03	1.08	2.22E-02	ADT
LTBP3	6.86	0.185	6.705	0.184	1.58E-03	1.11	2.22E-02	ADT
GNG10	10.291	0.183	10.089	0.322	1.59E-03	1.15	2.23E-02	ADT
PIGG	7.116	0.126	7.221	0.122	1.59E-03	-1.08	2.23E-02	ADT
ODZ1	5.587	0.822	4.854	0.969	1.59E-03	1.66	2.23E-02	ADT
PACS2	5.77	0.108	5.678	0.115	1.60E-03	1.07	2.25E-02	ADT
LRRC6	7.666	0.534	7.118	0.837	1.61E-03	1.46	2.25E-02	ADT
GPATCH3	5.849	0.134	5.967	0.153	1.61E-03	-1.09	2.26E-02	ADT
DAZAP2	9.685	0.167	9.813	0.114	1.62E-03	-1.09	2.26E-02	ADT
PRDM7	4.177	0.147	4.052	0.154	1.62E-03	1.09	2.26E-02	ADT
PPP1R15B	8.497	0.236	8.709	0.283	1.62E-03	-1.16	2.26E-02	ADT
PSG3	3.542	0.219	3.743	0.275	1.63E-03	-1.15	2.26E-02	ADT
TSPAN11	5.67	0.135	5.559	0.125	1.62E-03	1.08	2.26E-02	ADT
SEPT5	6.564	0.192	6.389	0.239	1.62E-03	1.13	2.26E-02	ADT
APPL1	5.927	0.66	6.457	0.568	1.63E-03	-1.44	2.27E-02	ADT
SLITRK5	5.381	0.175	5.241	0.147	1.63E-03	1.10	2.27E-02	ADT
E2F4	7.645	0.148	7.774	0.164	1.64E-03	-1.09	2.27E-02	ADT
NUDT18	6.558	0.182	6.701	0.141	1.64E-03	-1.10	2.28E-02	ADT
KRTAP10-1	6.651	0.193	6.493	0.179	1.65E-03	1.12	2.28E-02	ADT
NUP107	4.407	0.324	4.678	0.322	1.65E-03	-1.21	2.28E-02	ADT
CUX2	5.359	0.088	5.285	0.09	1.65E-03	1.05	2.28E-02	ADT
ADRA2A	5.557	0.131	5.455	0.098	1.65E-03	1.07	2.28E-02	ADT
SMUG1	5.628	0.205	5.789	0.161	1.66E-03	-1.12	2.29E-02	ADT
ADAM17	7.68	0.394	7.984	0.283	1.66E-03	-1.23	2.29E-02	ADT
UTF1	6.433	0.131	6.313	0.169	1.67E-03	1.09	2.30E-02	ADT
NPHP4	6.165	0.078	6.096	0.091	1.66E-03	1.05	2.30E-02	ADT
GOLGA2	6.713	0.154	6.831	0.107	1.67E-03	-1.09	2.30E-02	ADT
CALM3	8.544	0.106	8.651	0.163	1.67E-03	-1.08	2.30E-02	ADT
LANCL2	5.001	0.135	5.123	0.165	1.67E-03	-1.09	2.30E-02	ADT
MGAT1	6.462	0.123	6.562	0.113	1.67E-03	-1.07	2.30E-02	ADT
SLC2A4RG	6.397	0.1	6.295	0.155	1.68E-03	1.07	2.31E-02	ADT
SLC17A7	5.362	0.114	5.269	0.102	1.69E-03	1.07	2.32E-02	ADT
CRB3	6.69	0.131	6.582	0.127	1.69E-03	1.08	2.32E-02	ADT
ZFAND2A	8.562	0.321	8.824	0.3	1.69E-03	-1.20	2.32E-02	ADT
IRF3	7.22	0.11	7.316	0.124	1.71E-03	-1.07	2.34E-02	ADT
POLR2G	6.384	0.196	6.542	0.176	1.73E-03	-1.12	2.35E-02	ADT
KRT77	4.923	0.152	4.802	0.128	1.73E-03	1.09	2.36E-02	ADT
TADA2B	8.382	0.146	8.515	0.187	1.73E-03	-1.10	2.36E-02	ADT
MIF	6.36	0.126	6.237	0.183	1.73E-03	1.09	2.36E-02	ADT
EXT1	6.415	0.383	6.047	0.539	1.73E-03	1.29	2.36E-02	ADT

FAM208A	5.421	0.339	5.691	0.29	1.73E-03	-1.21	2.36E-02	ADT
ZUFSP	3.072	0.191	3.227	0.177	1.73E-03	-1.11	2.36E-02	ADT
MAPK8IP3	7.486	0.152	7.35	0.186	1.72E-03	1.10	2.36E-02	ADT
PTK6	6.306	0.129	6.196	0.139	1.72E-03	1.08	2.36E-02	ADT
SSR1	7.661	0.309	7.897	0.216	1.74E-03	-1.18	2.36E-02	ADT
ZNF787	7.424	0.119	7.327	0.108	1.74E-03	1.07	2.36E-02	ADT
ZBTB7C	5.863	0.14	5.745	0.147	1.75E-03	1.09	2.36E-02	ADT
CASP8AP2	5.845	0.432	6.201	0.42	1.74E-03	-1.28	2.37E-02	ADT
UHRF1	5.134	0.13	5.034	0.098	1.75E-03	1.07	2.37E-02	ADT
SLC35E4	5.366	0.135	5.232	0.202	1.75E-03	1.10	2.37E-02	ADT
MRPL36	4.97	0.184	4.803	0.229	1.74E-03	1.12	2.37E-02	ADT
GIPC3	5.661	0.153	5.538	0.138	1.76E-03	1.09	2.37E-02	ADT
ADRA1D	5.099	0.198	4.933	0.204	1.76E-03	1.12	2.37E-02	ADT
ZNF587	7.673	0.259	7.882	0.232	1.76E-03	-1.16	2.38E-02	ADT
FBXO22	6.256	0.199	6.437	0.251	1.77E-03	-1.13	2.38E-02	ADT
PSTPIP1	8.362	0.162	8.496	0.161	1.77E-03	-1.10	2.39E-02	ADT
LARP4B	7.346	0.156	7.47	0.136	1.78E-03	-1.09	2.39E-02	ADT
RAB3GAP1	6.105	0.269	6.316	0.22	1.78E-03	-1.16	2.39E-02	ADT
SLC35E2B	7.87	0.135	7.997	0.184	1.78E-03	-1.09	2.39E-02	ADT
EDEM2	7.532	0.135	7.649	0.151	1.78E-03	-1.08	2.40E-02	ADT
SPOP	6.988	0.161	7.115	0.132	1.79E-03	-1.09	2.40E-02	ADT
ODZ4	4.567	0.089	4.491	0.095	1.79E-03	1.05	2.40E-02	ADT
TEX13B	5.169	0.175	5.019	0.194	1.80E-03	1.11	2.42E-02	ADT
POU5F1	6.122	0.187	5.96	0.21	1.81E-03	1.12	2.42E-02	ADT
PRICKLE4	4.947	0.164	4.804	0.189	1.81E-03	1.10	2.42E-02	ADT
CCDC114	5.51	0.102	5.42	0.124	1.81E-03	1.06	2.42E-02	ADT
CYGB	5.756	0.185	5.603	0.182	1.81E-03	1.11	2.42E-02	ADT
PRPH	5.427	0.135	5.316	0.131	1.82E-03	1.08	2.43E-02	ADT
ARMC10	5.51	0.332	5.77	0.27	1.82E-03	-1.20	2.43E-02	ADT
USP4	10.169	0.243	10.371	0.246	1.83E-03	-1.15	2.44E-02	ADT
ODF3L2	6.741	0.17	6.603	0.162	1.84E-03	1.10	2.45E-02	ADT
PHLDA2	6.96	0.155	6.826	0.174	1.85E-03	1.10	2.45E-02	ADT
SELV	5.695	0.165	5.558	0.164	1.85E-03	1.10	2.45E-02	ADT
FOXJ2	7.5	0.143	7.382	0.14	1.84E-03	1.09	2.46E-02	ADT
NDUFB3	6.672	0.505	6.218	0.628	1.85E-03	1.37	2.46E-02	ADT
ONECUT3	6.777	0.161	6.65	0.137	1.85E-03	1.09	2.46E-02	ADT
FBLN7	5.992	0.124	5.889	0.125	1.86E-03	1.07	2.46E-02	ADT
WNT3	5.405	0.182	5.254	0.183	1.85E-03	1.11	2.46E-02	ADT
SPINK6	3.387	0.172	3.548	0.233	1.86E-03	-1.12	2.46E-02	ADT
FAM20B	5.583	0.153	5.721	0.191	1.86E-03	-1.10	2.46E-02	ADT
PCMT1	8.591	0.485	8.96	0.349	1.86E-03	-1.29	2.46E-02	ADT
TCF24	5.535	0.102	5.449	0.109	1.86E-03	1.06	2.46E-02	ADT
NPDC1	6.447	0.167	6.315	0.144	1.88E-03	1.10	2.49E-02	ADT
TRIM72	5.782	0.153	5.654	0.157	1.89E-03	1.09	2.49E-02	ADT
CCDC42B	5.034	0.171	4.877	0.224	1.89E-03	1.11	2.49E-02	ADT
RNPS1	6.392	0.147	6.527	0.196	1.89E-03	-1.10	2.49E-02	ADT
OR7A5	2.658	0.14	2.782	0.168	1.90E-03	-1.09	2.49E-02	ADT

ADAMTS13	6.342	0.106	6.256	0.1	1.90E-03	1.06	2.50E-02	ADT
TYW5	3.963	0.198	4.147	0.265	1.90E-03	-1.14	2.50E-02	ADT
SLC22A17	6.491	0.086	6.413	0.11	1.90E-03	1.06	2.50E-02	ADT
CDKL3	2.715	0.111	2.817	0.147	1.91E-03	-1.07	2.50E-02	ADT
CCDC107	5.85	0.107	5.763	0.103	1.91E-03	1.06	2.50E-02	ADT
NEDD8	7.244	0.211	7.418	0.212	1.92E-03	-1.13	2.52E-02	ADT
INTS10	5.662	0.262	5.874	0.248	1.93E-03	-1.16	2.52E-02	ADT
RAB6A	8.143	0.164	8.275	0.152	1.93E-03	-1.10	2.52E-02	ADT
NFYC	8.273	0.121	8.369	0.108	1.93E-03	-1.07	2.53E-02	ADT
SCUBE1	5.805	0.118	5.711	0.104	1.94E-03	1.07	2.54E-02	ADT
ZNF524	6.919	0.178	6.774	0.174	1.95E-03	1.11	2.54E-02	ADT
TMEM72	5.639	0.195	5.492	0.135	1.95E-03	1.11	2.54E-02	ADT
SDS	4.802	0.144	4.681	0.157	1.97E-03	1.09	2.56E-02	ADT
TMEM14B	6.716	0.352	7.003	0.344	1.97E-03	-1.22	2.56E-02	ADT
ARSH	4.452	0.169	4.321	0.14	1.97E-03	1.10	2.57E-02	ADT
LCN1	4.805	0.174	4.661	0.18	1.98E-03	1.10	2.57E-02	ADT
NKPD1	5.804	0.135	5.699	0.112	1.99E-03	1.08	2.59E-02	ADT
CCDC33	5.074	0.126	4.976	0.105	1.99E-03	1.07	2.59E-02	ADT
JPH3	5.012	0.164	4.887	0.127	2.00E-03	1.09	2.59E-02	ADT
PITPNM2	5.291	0.152	5.164	0.16	2.00E-03	1.09	2.59E-02	ADT
PTPRJ	10.052	0.244	9.816	0.359	2.00E-03	1.18	2.59E-02	ADT
PCP2	6.414	0.149	6.292	0.148	2.00E-03	1.09	2.59E-02	ADT
CLIP3	5.854	0.126	5.757	0.098	2.01E-03	1.07	2.60E-02	ADT
CPLX3	4.798	0.183	4.646	0.19	2.02E-03	1.11	2.61E-02	ADT
ZNF451	5.683	0.215	5.868	0.249	2.03E-03	-1.14	2.62E-02	ADT
GTF2IRD2	6.898	0.188	7.069	0.249	2.03E-03	-1.13	2.63E-02	ADT
MVD	6.365	0.124	6.267	0.113	2.04E-03	1.07	2.63E-02	ADT
PSMD11	6.374	0.22	6.553	0.214	2.05E-03	-1.13	2.63E-02	ADT
OGDHL	4.878	0.121	4.778	0.123	2.04E-03	1.07	2.63E-02	ADT
FOXL2	6.323	0.166	6.178	0.201	2.04E-03	1.11	2.63E-02	ADT
GTPBP10	5.667	0.275	5.888	0.262	2.04E-03	-1.17	2.63E-02	ADT
MOB3A	11.24	0.119	11.344	0.146	2.04E-03	-1.07	2.63E-02	ADT
HOXA7	5.32	0.178	5.17	0.194	2.05E-03	1.11	2.63E-02	ADT
ANGPT4	4.912	0.148	4.784	0.171	2.05E-03	1.09	2.63E-02	ADT
UPRT	4.34	0.392	4.648	0.346	2.06E-03	-1.24	2.64E-02	ADT
FOXI2	6.228	0.147	6.114	0.121	2.06E-03	1.08	2.64E-02	ADT
ZNF446	6.684	0.169	6.557	0.119	2.06E-03	1.09	2.64E-02	ADT
UROD	5.41	0.196	5.581	0.236	2.06E-03	-1.13	2.64E-02	ADT
CCDC157	6.023	0.123	5.921	0.124	2.06E-03	1.07	2.64E-02	ADT
FDXR	5.994	0.103	5.907	0.115	2.06E-03	1.06	2.64E-02	ADT
LARP6	4.793	0.143	4.681	0.124	2.07E-03	1.08	2.64E-02	ADT
RECQL	4.432	0.312	4.673	0.251	2.07E-03	-1.18	2.64E-02	ADT
TICAM1	6.913	0.128	6.814	0.102	2.08E-03	1.07	2.66E-02	ADT
CYS1	6.009	0.201	5.849	0.185	2.09E-03	1.12	2.66E-02	ADT
FTCD	6.06	0.127	5.956	0.127	2.10E-03	1.07	2.67E-02	ADT
UBAC2	6.248	0.207	6.051	0.297	2.10E-03	1.15	2.67E-02	ADT
DDX20	5.442	0.329	5.686	0.218	2.11E-03	-1.18	2.68E-02	ADT

ATP5S	4.787	0.236	4.998	0.299	2.12E-03	-1.16	2.69E-02	ADT
KRT33B	5.458	0.186	5.311	0.167	2.12E-03	1.11	2.69E-02	ADT
ERP27	7.813	0.26	7.578	0.336	2.12E-03	1.18	2.69E-02	ADT
SP6	5.794	0.157	5.661	0.178	2.12E-03	1.10	2.69E-02	ADT
FGF19	5.017	0.179	4.868	0.19	2.13E-03	1.11	2.69E-02	ADT
KHNYN	6.855	0.108	6.948	0.124	2.13E-03	-1.07	2.70E-02	ADT
NCK1	5.467	0.203	5.625	0.174	2.14E-03	-1.12	2.70E-02	ADT
ZCCHC12	3.924	0.154	3.795	0.166	2.14E-03	1.09	2.70E-02	ADT
LRRC69	2.84	0.159	2.986	0.214	2.14E-03	-1.11	2.71E-02	ADT
LMNA	5.578	0.179	5.437	0.163	2.14E-03	1.10	2.71E-02	ADT
SEC62	6.545	0.302	6.771	0.216	2.14E-03	-1.17	2.71E-02	ADT
TMEM208	7.379	0.28	7.622	0.334	2.16E-03	-1.18	2.72E-02	ADT
KPNA6	6.925	0.18	7.074	0.192	2.15E-03	-1.11	2.72E-02	ADT
PCSK4	5.825	0.137	5.718	0.122	2.16E-03	1.08	2.72E-02	ADT
RASSF10	6.08	0.186	5.935	0.16	2.18E-03	1.11	2.74E-02	ADT
TMED9	6.434	0.159	6.572	0.19	2.18E-03	-1.10	2.74E-02	ADT
SERPINF2	5.704	0.139	5.593	0.133	2.18E-03	1.08	2.74E-02	ADT
SBNO2	8.899	0.177	8.741	0.226	2.19E-03	1.12	2.75E-02	ADT
CDH22	6.38	0.126	6.278	0.124	2.19E-03	1.07	2.75E-02	ADT
ZFR	4.185	0.243	4.374	0.21	2.20E-03	-1.14	2.76E-02	ADT
EXOC5	5.58	0.359	5.85	0.266	2.20E-03	-1.21	2.76E-02	ADT
S100A10	8.77	0.851	9.426	0.707	2.20E-03	-1.58	2.76E-02	ADT
GREM1	5.07	0.118	4.968	0.141	2.20E-03	1.07	2.76E-02	ADT
WNT2B	5.235	0.139	5.105	0.195	2.21E-03	1.09	2.76E-02	ADT
NME6	5.822	0.265	6.031	0.24	2.21E-03	-1.16	2.76E-02	ADT
TM9SF4	7.296	0.176	7.467	0.265	2.21E-03	-1.13	2.76E-02	ADT
RNF40	7.305	0.112	7.405	0.144	2.24E-03	-1.07	2.79E-02	ADT
ARHGAP23	5.435	0.1	5.355	0.098	2.24E-03	1.06	2.79E-02	ADT
FAM76A	5.119	0.229	5.296	0.195	2.25E-03	-1.13	2.80E-02	ADT
IP6K2	6.539	0.204	6.692	0.151	2.25E-03	-1.11	2.80E-02	ADT
MBD1	6.802	0.124	6.905	0.133	2.26E-03	-1.07	2.81E-02	ADT
AIPL1	5.198	0.142	5.089	0.115	2.27E-03	1.08	2.82E-02	ADT
TRMT61A	6.374	0.15	6.247	0.17	2.27E-03	1.09	2.82E-02	ADT
GOLGA5	7.668	0.296	7.888	0.21	2.27E-03	-1.16	2.82E-02	ADT
RAB40A	4.954	0.155	4.838	0.111	2.28E-03	1.08	2.83E-02	ADT
RNF139	7.156	0.223	7.333	0.208	2.28E-03	-1.13	2.83E-02	ADT
COL13A1	5.442	0.143	5.332	0.122	2.30E-03	1.08	2.84E-02	ADT
STX12	7.298	0.303	7.522	0.207	2.30E-03	-1.17	2.84E-02	ADT
PRMT10	4.972	0.272	5.216	0.356	2.30E-03	-1.18	2.85E-02	ADT
ATG13	8.402	0.134	8.505	0.113	2.31E-03	-1.07	2.85E-02	ADT
PRODH2	5.188	0.129	5.087	0.116	2.31E-03	1.07	2.85E-02	ADT
ASTE1	5.04	0.27	5.248	0.232	2.32E-03	-1.16	2.86E-02	ADT
GALR1	4.424	0.15	4.297	0.173	2.32E-03	1.09	2.86E-02	ADT
UHMK1	5.356	0.182	5.5	0.172	2.32E-03	-1.10	2.86E-02	ADT
MOB3B	5.444	0.153	5.312	0.182	2.32E-03	1.10	2.86E-02	ADT
DGUOK	7.588	0.269	7.806	0.274	2.33E-03	-1.16	2.86E-02	ADT
C11orf95	6.674	0.136	6.57	0.112	2.34E-03	1.07	2.88E-02	ADT

VAT1	6.453	0.144	6.331	0.165	2.34E-03	1.09	2.88E-02	ADT
CABLES2	6.504	0.135	6.394	0.139	2.35E-03	1.08	2.88E-02	ADT
LOR	7.114	0.163	6.983	0.163	2.35E-03	1.10	2.89E-02	ADT
RND2	4.818	0.124	4.709	0.156	2.37E-03	1.08	2.91E-02	ADT
FAM177A1	4.932	0.196	5.084	0.172	2.37E-03	-1.11	2.91E-02	ADT
FOLR1	5.005	0.147	4.886	0.151	2.38E-03	1.09	2.91E-02	ADT
SHBG	5.811	0.129	5.711	0.115	2.38E-03	1.07	2.91E-02	ADT
AFAP1L2	4.653	0.132	4.551	0.119	2.38E-03	1.07	2.91E-02	ADT
CAPN5	5.556	0.105	5.462	0.138	2.39E-03	1.07	2.93E-02	ADT
KRTCAP2	7.485	0.233	7.674	0.237	2.40E-03	-1.14	2.93E-02	ADT
APLN	4.854	0.112	4.754	0.148	2.41E-03	1.07	2.94E-02	ADT
MNT	7.274	0.169	7.145	0.139	2.41E-03	1.09	2.94E-02	ADT
RIMS4	5.19	0.138	5.083	0.125	2.42E-03	1.08	2.95E-02	ADT
RRS1	5.418	0.28	5.19	0.292	2.43E-03	1.17	2.96E-02	ADT
LIMD2	9.372	0.123	9.267	0.146	2.44E-03	1.08	2.97E-02	ADT
ZSCAN21	4.86	0.146	4.99	0.189	2.44E-03	-1.09	2.97E-02	ADT
CDK9	8.154	0.154	8.275	0.144	2.44E-03	-1.09	2.97E-02	ADT
C16orf82	5.182	0.16	5.062	0.125	2.45E-03	1.09	2.97E-02	ADT
AMH	7.075	0.13	6.973	0.121	2.44E-03	1.07	2.97E-02	ADT
SEMA4B	7.808	0.127	7.684	0.198	2.45E-03	1.09	2.98E-02	ADT
NEB	4.874	0.266	4.667	0.239	2.46E-03	1.15	2.98E-02	ADT
LRCH3	7.619	0.249	7.823	0.263	2.47E-03	-1.15	2.99E-02	ADT
EIF2S3	8.321	0.237	8.502	0.202	2.48E-03	-1.13	2.99E-02	ADT
PARG	6.449	0.263	6.645	0.194	2.48E-03	-1.15	2.99E-02	ADT
UMOD	5.199	0.133	5.094	0.126	2.47E-03	1.08	2.99E-02	ADT
SCRT1	4.899	0.171	4.756	0.193	2.47E-03	1.10	3.00E-02	ADT
ATP4A	5.201	0.132	5.098	0.12	2.49E-03	1.07	3.00E-02	ADT
NAA30	4.723	0.199	4.886	0.215	2.49E-03	-1.12	3.01E-02	ADT
SRP9	8.092	0.305	8.316	0.211	2.51E-03	-1.17	3.02E-02	ADT
EVPL	6.008	0.101	5.92	0.124	2.52E-03	1.06	3.04E-02	ADT
LRP3	6.225	0.113	6.13	0.133	2.53E-03	1.07	3.05E-02	ADT
PIK3CB	7.427	0.384	7.73	0.365	2.53E-03	-1.23	3.05E-02	ADT
CSTF3	6.59	0.55	6.993	0.378	2.54E-03	-1.32	3.05E-02	ADT
MARCH4	5.416	0.115	5.322	0.125	2.54E-03	1.07	3.05E-02	ADT
BACE1	4.866	0.092	4.783	0.122	2.54E-03	1.06	3.05E-02	ADT
KIAA0664	5.715	0.13	5.611	0.13	2.55E-03	1.07	3.06E-02	ADT
MYL5	5.383	0.126	5.269	0.17	2.55E-03	1.08	3.06E-02	ADT
KIAA1383	4.353	0.102	4.273	0.096	2.55E-03	1.06	3.06E-02	ADT
THRA	6.41	0.168	6.255	0.236	2.56E-03	1.11	3.06E-02	ADT
ZDHHC16	5.754	0.267	5.979	0.31	2.57E-03	-1.17	3.08E-02	ADT
FAM63B	4.252	0.254	4.462	0.28	2.57E-03	-1.16	3.08E-02	ADT
NUDT8	6.569	0.158	6.453	0.114	2.58E-03	1.08	3.09E-02	ADT
ST5	4.38	0.113	4.294	0.1	2.58E-03	1.06	3.09E-02	ADT
KNDC1	5.854	0.138	5.754	0.093	2.60E-03	1.07	3.10E-02	ADT
LNX2	5.841	0.338	6.103	0.308	2.60E-03	-1.20	3.10E-02	ADT
CCM2	8.11	0.251	8.307	0.239	2.60E-03	-1.15	3.10E-02	ADT
WDR62	5.669	0.098	5.596	0.076	2.60E-03	1.05	3.10E-02	ADT

RGAG1	3.876	0.125	3.77	0.145	2.60E-03	1.08	3.10E-02	ADT
ATP5C1	6.915	0.301	7.134	0.206	2.60E-03	-1.16	3.10E-02	ADT
CCNI	8.843	0.143	8.973	0.198	2.60E-03	-1.09	3.10E-02	ADT
PRTN3	6.862	0.149	6.744	0.148	2.61E-03	1.09	3.10E-02	ADT
ECH1	7.839	0.404	8.169	0.435	2.62E-03	-1.26	3.11E-02	ADT
TLE6	5.125	0.141	5.017	0.125	2.63E-03	1.08	3.11E-02	ADT
C6orf170	3.285	0.189	3.441	0.21	2.63E-03	-1.11	3.12E-02	ADT
RNF25	5.727	0.145	5.86	0.205	2.63E-03	-1.10	3.12E-02	ADT
GRIK4	5.24	0.11	5.152	0.111	2.63E-03	1.06	3.12E-02	ADT
OR8B3	2.242	0.31	2.531	0.45	2.64E-03	-1.22	3.12E-02	ADT
ZNF28	5.087	0.317	5.329	0.278	2.64E-03	-1.18	3.13E-02	ADT
MYH6	5.082	0.11	4.992	0.119	2.64E-03	1.06	3.13E-02	ADT
MUC6	5.889	0.114	5.8	0.106	2.65E-03	1.06	3.13E-02	ADT
DBNDD2	6	0.128	5.898	0.13	2.67E-03	1.07	3.14E-02	ADT
ZNF845	5.418	0.411	5.717	0.287	2.66E-03	-1.23	3.14E-02	ADT
PENK	5.223	0.169	5.086	0.178	2.68E-03	1.10	3.15E-02	ADT
PAFAH2	5.854	0.267	6.097	0.369	2.68E-03	-1.18	3.15E-02	ADT
ITIH2	3.657	0.105	3.577	0.094	2.68E-03	1.06	3.15E-02	ADT
PMF1	7.165	0.195	7.339	0.261	2.68E-03	-1.13	3.16E-02	ADT
ANKLE1	6.344	0.091	6.256	0.142	2.69E-03	1.06	3.16E-02	ADT
ZNF423	4.464	0.113	4.377	0.101	2.70E-03	1.06	3.17E-02	ADT
FOXA1	4.91	0.152	4.797	0.121	2.71E-03	1.08	3.18E-02	ADT
CRCP	5.872	0.22	6.044	0.208	2.72E-03	-1.13	3.19E-02	ADT
GNAZ	6.16	0.257	5.963	0.225	2.72E-03	1.15	3.19E-02	ADT
GPR135	6.964	0.13	6.861	0.13	2.74E-03	1.07	3.21E-02	ADT
COL9A2	6.667	0.176	6.531	0.162	2.75E-03	1.10	3.22E-02	ADT
CALML5	4.784	0.168	4.635	0.221	2.75E-03	1.11	3.22E-02	ADT
FKBP7	3.794	0.136	3.922	0.203	2.76E-03	-1.09	3.23E-02	ADT
GPN2	6.594	0.122	6.693	0.132	2.77E-03	-1.07	3.24E-02	ADT
ANXA8L2	7.312	0.183	7.175	0.151	2.79E-03	1.10	3.24E-02	ADT
TRMT12	4.937	0.463	5.308	0.479	2.78E-03	-1.29	3.24E-02	ADT
MRPL10	6.072	0.248	6.27	0.254	2.78E-03	-1.15	3.24E-02	ADT
PSMD5	5.848	0.297	6.071	0.244	2.78E-03	-1.17	3.24E-02	ADT
IQCC	5.677	0.165	5.545	0.171	2.78E-03	1.10	3.24E-02	ADT
COL1A1	5.977	0.107	5.891	0.113	2.78E-03	1.06	3.24E-02	ADT
CHTF18	6.619	0.081	6.549	0.101	2.78E-03	1.05	3.24E-02	ADT
SNAPC1	5.224	0.328	5.529	0.478	2.80E-03	-1.24	3.25E-02	ADT
GMNC	2.828	0.178	2.964	0.158	2.80E-03	-1.10	3.25E-02	ADT
VMO1	4.764	0.246	4.581	0.198	2.81E-03	1.14	3.26E-02	ADT
TGS1	5.606	0.251	5.794	0.208	2.81E-03	-1.14	3.26E-02	ADT
PEX12	5.798	0.348	6.072	0.345	2.81E-03	-1.21	3.26E-02	ADT
LRSAM1	5.85	0.203	6.014	0.216	2.81E-03	-1.12	3.26E-02	ADT
TEF	6.071	0.146	5.957	0.139	2.82E-03	1.08	3.26E-02	ADT
ANAPC5	6.974	0.26	7.186	0.285	2.83E-03	-1.16	3.28E-02	ADT
TPMT	3.899	0.284	4.129	0.306	2.84E-03	-1.17	3.29E-02	ADT
LOC388849	6.045	0.158	5.924	0.143	2.84E-03	1.09	3.29E-02	ADT
RPL7L1	5.822	0.318	6.072	0.314	2.85E-03	-1.19	3.29E-02	ADT

WNT10B	5.907	0.124	5.812	0.113	2.85E-03	1.07	3.29E-02	ADT
UBXN2A	3.391	0.311	3.641	0.329	2.86E-03	-1.19	3.29E-02	ADT
KCTD6	5.074	0.22	5.27	0.298	2.86E-03	-1.15	3.30E-02	ADT
MKRN1	8.826	0.168	8.946	0.112	2.87E-03	-1.09	3.30E-02	ADT
LHX4	5.024	0.17	4.899	0.13	2.87E-03	1.09	3.30E-02	ADT
HRH1	4.012	0.178	4.142	0.132	2.88E-03	-1.09	3.31E-02	ADT
MIR205HG	3.229	0.207	3.39	0.198	2.88E-03	-1.12	3.32E-02	ADT
FAM82B	5.022	0.316	5.272	0.319	2.89E-03	-1.19	3.32E-02	ADT
TTC14	5.481	0.319	5.725	0.286	2.89E-03	-1.18	3.32E-02	ADT
TUSC1	5.507	0.166	5.384	0.131	2.89E-03	1.09	3.32E-02	ADT
ADPGK	8.168	0.17	8.299	0.156	2.90E-03	-1.10	3.33E-02	ADT
PUS1	5.954	0.156	5.834	0.146	2.91E-03	1.09	3.34E-02	ADT
TPM2	5.596	0.162	5.468	0.165	2.91E-03	1.09	3.34E-02	ADT
MFAP4	4.746	0.138	4.64	0.128	2.93E-03	1.08	3.35E-02	ADT
ZIC2	6.581	0.102	6.506	0.077	2.94E-03	1.05	3.36E-02	ADT
ARMCX2	4.436	0.112	4.348	0.111	2.94E-03	1.06	3.36E-02	ADT
ZMYM5	6.048	0.278	6.259	0.247	2.94E-03	-1.16	3.36E-02	ADT
SOX21	5.372	0.136	5.257	0.167	2.95E-03	1.08	3.36E-02	ADT
TNC	4.182	0.097	4.107	0.092	2.95E-03	1.05	3.37E-02	ADT
RNF113B	4.858	0.203	4.69	0.235	2.95E-03	1.12	3.37E-02	ADT
CCDC87	4.935	0.155	4.811	0.164	2.96E-03	1.09	3.37E-02	ADT
TMEM183A	8.032	0.191	8.171	0.143	2.97E-03	-1.10	3.37E-02	ADT
CELSR1	5.353	0.097	5.273	0.113	2.96E-03	1.06	3.37E-02	ADT
DNTTIP1	9.094	0.171	9.229	0.173	2.98E-03	-1.10	3.38E-02	ADT
POLRMT	6.085	0.087	6.012	0.102	2.98E-03	1.05	3.38E-02	ADT
PTPN7	6.936	0.228	7.124	0.26	2.99E-03	-1.14	3.39E-02	ADT
BIN2	9.654	0.219	9.811	0.15	2.98E-03	-1.11	3.39E-02	ADT
GAB3	8.281	0.146	8.427	0.245	2.99E-03	-1.11	3.39E-02	ADT
FUS	8.32	0.246	8.502	0.2	2.99E-03	-1.13	3.39E-02	ADT
C9orf72	9.12	0.14	9.233	0.148	3.00E-03	-1.08	3.39E-02	ADT
FBRSL1	7.175	0.112	7.077	0.147	3.00E-03	1.07	3.40E-02	ADT
HRAS	6.26	0.119	6.161	0.14	3.00E-03	1.07	3.40E-02	ADT
FBXW11	4.865	0.362	5.14	0.324	3.01E-03	-1.21	3.40E-02	ADT
NTM	4.503	0.115	4.416	0.103	3.01E-03	1.06	3.40E-02	ADT
IL18BP	6.905	0.226	7.098	0.284	3.03E-03	-1.14	3.42E-02	ADT
MGAT5B	6.134	0.15	6.027	0.105	3.04E-03	1.08	3.43E-02	ADT
RPS3	7.786	0.326	7.531	0.324	3.06E-03	1.19	3.44E-02	ADT
UFSP2	3.9	0.25	4.096	0.25	3.05E-03	-1.15	3.44E-02	ADT
MDP1	5.375	0.269	5.57	0.202	3.06E-03	-1.14	3.44E-02	ADT
RGS11	5.233	0.149	5.125	0.11	3.05E-03	1.08	3.44E-02	ADT
DDX59	5.263	0.24	5.441	0.198	3.06E-03	-1.13	3.44E-02	ADT
ZNF732	3.536	0.137	3.652	0.169	3.06E-03	-1.08	3.44E-02	ADT
FAM186B	5.22	0.238	5.04	0.214	3.07E-03	1.13	3.44E-02	ADT
KDM4A	6.55	0.176	6.681	0.144	3.06E-03	-1.10	3.44E-02	ADT
OGDH	7.92	0.184	8.072	0.213	3.07E-03	-1.11	3.44E-02	ADT
CDC26	6.151	0.309	6.384	0.276	3.06E-03	-1.18	3.44E-02	ADT
SAR1B	5.715	0.343	5.99	0.371	3.09E-03	-1.21	3.46E-02	ADT

OR10V1	3.91	0.406	3.616	0.301	3.09E-03	1.23	3.46E-02	ADT
MAD2L1BP	7.504	0.327	7.756	0.317	3.10E-03	-1.19	3.47E-02	ADT
DNAJC18	4.887	0.145	5	0.146	3.10E-03	-1.08	3.47E-02	ADT
DUSP16	6.368	0.272	6.583	0.283	3.13E-03	-1.16	3.49E-02	ADT
ZNF358	6.385	0.134	6.283	0.126	3.13E-03	1.07	3.49E-02	ADT
NRBP2	5.518	0.159	5.386	0.187	3.12E-03	1.10	3.49E-02	ADT
MBD3L1	2.624	0.176	2.464	0.254	3.12E-03	1.12	3.49E-02	ADT
KLK14	6.054	0.137	5.945	0.147	3.13E-03	1.08	3.49E-02	ADT
HUNK	4.28	0.138	4.174	0.133	3.14E-03	1.08	3.50E-02	ADT
NRM	6.439	0.229	6.627	0.262	3.15E-03	-1.14	3.50E-02	ADT
GPKOW	6.649	0.13	6.759	0.157	3.15E-03	-1.08	3.50E-02	ADT
ASGR2	5.667	0.143	5.56	0.125	3.15E-03	1.08	3.50E-02	ADT
GAGE10	4.526	0.217	4.349	0.248	3.15E-03	1.13	3.51E-02	ADT
MYO7A	5.232	0.104	5.154	0.093	3.16E-03	1.06	3.51E-02	ADT
EMC10	6.846	0.179	6.978	0.147	3.17E-03	-1.10	3.51E-02	ADT
LDHD	6.042	0.141	5.934	0.131	3.17E-03	1.08	3.51E-02	ADT
MAPT	5.099	0.113	5.015	0.095	3.18E-03	1.06	3.52E-02	ADT
ZNF410	5.779	0.186	5.923	0.182	3.19E-03	-1.10	3.53E-02	ADT
GABARAP	12.331	0.1	12.257	0.087	3.19E-03	1.05	3.54E-02	ADT
GALC	6.775	0.349	7.034	0.295	3.20E-03	-1.20	3.54E-02	ADT
LILRB5	5.89	0.113	5.787	0.164	3.21E-03	1.07	3.55E-02	ADT
EGFL7	5.965	0.241	5.77	0.267	3.21E-03	1.14	3.55E-02	ADT
INTS12	4.4	0.2	4.561	0.22	3.23E-03	-1.12	3.57E-02	ADT
PAK6	5.287	0.102	5.203	0.119	3.24E-03	1.06	3.57E-02	ADT
SALL4	4.626	0.137	4.517	0.145	3.24E-03	1.08	3.57E-02	ADT
SKIL	6.091	0.277	6.317	0.318	3.25E-03	-1.17	3.58E-02	ADT
ARHGEF17	5.23	0.108	5.139	0.132	3.25E-03	1.07	3.58E-02	ADT
YAF2	6.049	0.255	6.236	0.21	3.25E-03	-1.14	3.58E-02	ADT
ARFGAP2	6.553	0.135	6.674	0.19	3.26E-03	-1.09	3.59E-02	ADT
MKKS	3.646	0.423	3.974	0.418	3.27E-03	-1.26	3.59E-02	ADT
RBM33	8.333	0.102	8.246	0.129	3.27E-03	1.06	3.59E-02	ADT
SAMD11	6.524	0.134	6.427	0.105	3.28E-03	1.07	3.60E-02	ADT
ZNF784	7.168	0.107	7.082	0.12	3.28E-03	1.06	3.60E-02	ADT
CCDC121	5.565	0.15	5.695	0.199	3.29E-03	-1.09	3.60E-02	ADT
KRTAP16-1	5.685	0.179	5.551	0.157	3.28E-03	1.10	3.60E-02	ADT
DEFB4A	2.538	0.419	2.866	0.431	3.29E-03	-1.26	3.60E-02	ADT
LRRC3B	6.44	0.221	6.273	0.203	3.29E-03	1.12	3.60E-02	ADT
ANKRD33	5.285	0.135	5.184	0.122	3.30E-03	1.07	3.61E-02	ADT
SOX8	6.296	0.173	6.169	0.14	3.30E-03	1.09	3.61E-02	ADT
GAS2L2	6.12	0.137	6.013	0.139	3.30E-03	1.08	3.61E-02	ADT
C4orf21	6.459	0.382	6.747	0.348	3.31E-03	-1.22	3.61E-02	ADT
GLB1L2	5.233	0.106	5.149	0.112	3.32E-03	1.06	3.62E-02	ADT
DDX53	3.31	0.314	3.078	0.263	3.31E-03	1.17	3.62E-02	ADT
ARL2BP	6.335	0.351	6.589	0.271	3.33E-03	-1.19	3.62E-02	ADT
RRAGC	8.443	0.293	8.653	0.216	3.33E-03	-1.16	3.62E-02	ADT
TTC28	5.059	0.111	4.972	0.113	3.33E-03	1.06	3.63E-02	ADT
RGS9BP	4.23	0.221	4.053	0.241	3.33E-03	1.13	3.63E-02	ADT

RAD9A	7.027	0.149	7.152	0.185	3.35E-03	-1.09	3.65E-02	ADT
ZNF497	6.557	0.128	6.463	0.104	3.36E-03	1.07	3.66E-02	ADT
MED21	6.772	0.297	6.992	0.252	3.37E-03	-1.16	3.66E-02	ADT
FAIM	3.977	0.172	3.846	0.165	3.37E-03	1.10	3.66E-02	ADT
TGFBR2	9.577	0.168	9.458	0.115	3.38E-03	1.09	3.66E-02	ADT
NKD1	5.512	0.117	5.422	0.114	3.38E-03	1.06	3.66E-02	ADT
TMC6	9.806	0.213	9.961	0.171	3.39E-03	-1.11	3.67E-02	ADT
PSMC1	7.156	0.208	7.305	0.152	3.40E-03	-1.11	3.68E-02	ADT
ANO8	5.848	0.113	5.763	0.102	3.40E-03	1.06	3.68E-02	ADT
BHLHA9	7.151	0.243	6.977	0.179	3.40E-03	1.13	3.68E-02	ADT
TRIM21	8.565	0.388	8.855	0.347	3.41E-03	-1.22	3.68E-02	ADT
NEDD4	3.661	0.277	3.89	0.334	3.41E-03	-1.17	3.68E-02	ADT
TNFSF13	7.266	0.258	7.461	0.237	3.41E-03	-1.14	3.68E-02	ADT
CLASP2	4.662	0.624	5.138	0.597	3.43E-03	-1.39	3.70E-02	ADT
PISD	9.241	0.249	9.435	0.258	3.43E-03	-1.14	3.70E-02	ADT
CELF4	5.107	0.105	5.026	0.106	3.44E-03	1.06	3.70E-02	ADT
SPANXN2	4.148	0.349	3.869	0.386	3.44E-03	1.21	3.70E-02	ADT
PPP1R1B	6.103	0.127	6.006	0.123	3.44E-03	1.07	3.71E-02	ADT
STIP1	6.477	0.306	6.696	0.23	3.45E-03	-1.16	3.71E-02	ADT
EPM2A	3.884	0.166	3.751	0.185	3.46E-03	1.10	3.72E-02	ADT
CTGF	5.356	0.125	5.257	0.132	3.47E-03	1.07	3.73E-02	ADT
VASN	5.95	0.165	5.819	0.178	3.49E-03	1.10	3.74E-02	ADT
TOMM20	4.772	0.154	4.896	0.174	3.50E-03	-1.09	3.75E-02	ADT
TMEM161A	5.884	0.115	5.793	0.123	3.51E-03	1.07	3.77E-02	ADT
NDUFS1	6.418	0.289	6.623	0.206	3.52E-03	-1.15	3.77E-02	ADT
KIRREL2	5.326	0.108	5.245	0.101	3.52E-03	1.06	3.77E-02	ADT
YAP1	4.499	0.119	4.412	0.102	3.53E-03	1.06	3.78E-02	ADT
H2AFY2	4.992	0.122	4.899	0.119	3.54E-03	1.07	3.78E-02	ADT
PYCARD	9.451	0.161	9.31	0.219	3.54E-03	1.10	3.78E-02	ADT
FEM1A	6.905	0.071	6.84	0.106	3.54E-03	1.05	3.78E-02	ADT
ASMT	5.632	0.184	5.494	0.169	3.54E-03	1.10	3.78E-02	ADT
PLXNA3	5.845	0.165	5.71	0.197	3.54E-03	1.10	3.78E-02	ADT
DCAF15	7.187	0.121	7.28	0.123	3.55E-03	-1.07	3.78E-02	ADT
TNFRSF10B	6.632	0.31	6.876	0.329	3.55E-03	-1.18	3.79E-02	ADT
EBP	6.052	0.252	6.254	0.282	3.56E-03	-1.15	3.79E-02	ADT
EPHX4	4.169	0.147	4.273	0.106	3.56E-03	-1.07	3.79E-02	ADT
RASGRF1	4.413	0.1	4.333	0.109	3.56E-03	1.06	3.79E-02	ADT
GLIPR2	10.601	0.184	10.738	0.165	3.58E-03	-1.10	3.80E-02	ADT
TREX2	6.062	0.216	5.879	0.278	3.58E-03	1.14	3.80E-02	ADT
UPK3BL	8.811	0.371	9.087	0.332	3.59E-03	-1.21	3.81E-02	ADT
BRP44	6.739	0.245	6.929	0.249	3.58E-03	-1.14	3.81E-02	ADT
FAM3D	5.361	0.143	5.257	0.117	3.59E-03	1.07	3.81E-02	ADT
A2LD1	5.495	0.145	5.385	0.14	3.60E-03	1.08	3.81E-02	ADT
TUBB4B	8.88	0.202	9.054	0.267	3.60E-03	-1.13	3.81E-02	ADT
ASGR1	6.049	0.138	5.942	0.141	3.60E-03	1.08	3.81E-02	ADT
ZFAT	6.451	0.14	6.343	0.143	3.61E-03	1.08	3.82E-02	ADT
POLDIP2	7.951	0.13	8.053	0.138	3.62E-03	-1.07	3.82E-02	ADT

ERBB3	4.024	0.111	3.944	0.088	3.62E-03	1.06	3.83E-02	ADT
STYX	6.724	0.361	6.985	0.289	3.63E-03	-1.20	3.83E-02	ADT
GP9	6.927	0.221	6.765	0.185	3.63E-03	1.12	3.83E-02	ADT
PRSS27	6.883	0.138	6.779	0.128	3.63E-03	1.07	3.84E-02	ADT
USP29	3.173	0.276	2.973	0.228	3.64E-03	1.15	3.84E-02	ADT
AQP5	5.473	0.153	5.353	0.161	3.65E-03	1.09	3.85E-02	ADT
TMEM38B	4.15	0.213	4.322	0.245	3.65E-03	-1.13	3.85E-02	ADT
PPP2R2D	8.407	0.275	8.601	0.2	3.66E-03	-1.14	3.85E-02	ADT
SMTNL2	6.316	0.133	6.216	0.125	3.66E-03	1.07	3.85E-02	ADT
FAM176B	4.157	0.342	4.489	0.567	3.68E-03	-1.26	3.86E-02	ADT
TMEM50B	5.12	0.314	5.368	0.343	3.68E-03	-1.19	3.86E-02	ADT
MBLAC1	5.981	0.179	5.848	0.161	3.68E-03	1.10	3.86E-02	ADT
TSTA3	7.019	0.17	7.174	0.253	3.68E-03	-1.11	3.86E-02	ADT
TMEM68	4.083	0.329	4.324	0.281	3.67E-03	-1.18	3.86E-02	ADT
SCUBE2	4.775	0.104	4.697	0.096	3.68E-03	1.06	3.86E-02	ADT
FBXO34	6.02	0.194	6.166	0.182	3.69E-03	-1.11	3.87E-02	ADT
HIAT1	7.997	0.294	8.203	0.204	3.70E-03	-1.15	3.87E-02	ADT
AMOTL1	4.901	0.118	4.813	0.107	3.70E-03	1.06	3.87E-02	ADT
ERCC3	6.197	0.207	6.368	0.252	3.71E-03	-1.13	3.87E-02	ADT
POT1	4.176	0.307	4.399	0.253	3.71E-03	-1.17	3.88E-02	ADT
CTSZ	7.667	0.171	7.801	0.183	3.71E-03	-1.10	3.88E-02	ADT
SYCE1	4.296	0.137	4.184	0.162	3.71E-03	1.08	3.88E-02	ADT
KANSL2	5.991	0.332	6.241	0.314	3.71E-03	-1.19	3.88E-02	ADT
CYP20A1	5.333	0.191	5.486	0.216	3.73E-03	-1.11	3.88E-02	ADT
KLHDC10	5.996	0.192	6.143	0.191	3.73E-03	-1.11	3.88E-02	ADT
PTF1A	6.34	0.09	6.266	0.107	3.73E-03	1.05	3.88E-02	ADT
PLXNA2	6.578	0.328	6.312	0.382	3.73E-03	1.20	3.88E-02	ADT
SLC35D1	4.813	0.317	5.094	0.449	3.73E-03	-1.22	3.88E-02	ADT
ACSBG1	4.479	0.127	4.383	0.125	3.74E-03	1.07	3.88E-02	ADT
SH3PXD2A	4.909	0.117	4.823	0.103	3.74E-03	1.06	3.89E-02	ADT
RIPK4	5.028	0.201	4.878	0.185	3.75E-03	1.11	3.89E-02	ADT
IRS1	4.581	0.149	4.465	0.158	3.76E-03	1.08	3.90E-02	ADT
TNFRSF6B	5.302	0.173	5.165	0.19	3.78E-03	1.10	3.92E-02	ADT
TNRC18	8.109	0.109	8.027	0.106	3.78E-03	1.06	3.92E-02	ADT
TRMU	5.686	0.166	5.811	0.156	3.78E-03	-1.09	3.92E-02	ADT
DLG4	5.237	0.109	5.147	0.135	3.81E-03	1.06	3.94E-02	ADT
HOXB3	5.821	0.143	5.708	0.16	3.81E-03	1.08	3.94E-02	ADT
MYF5	4.513	0.198	4.373	0.146	3.81E-03	1.10	3.94E-02	ADT
SARS2	5.456	0.146	5.349	0.127	3.82E-03	1.08	3.94E-02	ADT
MAPK12	5.691	0.161	5.574	0.135	3.83E-03	1.08	3.96E-02	ADT
ASNSD1	5.604	0.381	5.303	0.423	3.84E-03	1.23	3.96E-02	ADT
MEA1	7.193	0.247	7.375	0.22	3.84E-03	-1.13	3.96E-02	ADT
GCG	3.119	0.185	3.262	0.192	3.84E-03	-1.10	3.96E-02	ADT
DUS2L	7.495	0.322	7.743	0.333	3.85E-03	-1.19	3.97E-02	ADT
MICB	6.804	0.298	7.043	0.342	3.86E-03	-1.18	3.97E-02	ADT
MMRN2	4.401	0.135	4.296	0.144	3.85E-03	1.08	3.97E-02	ADT
CKAP4	7.64	0.235	7.445	0.292	3.86E-03	1.14	3.97E-02	ADT

SP1	8.979	0.145	8.864	0.164	3.86E-03	1.08	3.97E-02	ADT
A1BG	6.411	0.125	6.316	0.125	3.86E-03	1.07	3.97E-02	ADT
BTN3A1	8.249	0.316	8.499	0.352	3.88E-03	-1.19	3.99E-02	ADT
COX5A	6.806	0.275	7.007	0.242	3.89E-03	-1.15	3.99E-02	ADT
C1orf131	6.64	0.301	6.409	0.31	3.90E-03	1.17	4.00E-02	ADT
YRDC	6.087	0.152	6.204	0.155	3.90E-03	-1.08	4.00E-02	ADT
JDP2	7.319	0.237	7.506	0.261	3.90E-03	-1.14	4.00E-02	ADT
GPR133	4.94	0.083	4.874	0.097	3.91E-03	1.05	4.00E-02	ADT
ZBTB48	6.708	0.18	6.852	0.208	3.91E-03	-1.10	4.00E-02	ADT
GYLTL1B	6.256	0.113	6.166	0.129	3.91E-03	1.06	4.00E-02	ADT
RBM23	9.731	0.154	9.852	0.169	3.93E-03	-1.09	4.01E-02	ADT
RAB5B	8.584	0.191	8.436	0.203	3.93E-03	1.11	4.01E-02	ADT
PPP1R14A	5.629	0.165	5.478	0.25	3.93E-03	1.11	4.02E-02	ADT
PNLIP	2.823	0.111	2.913	0.134	3.97E-03	-1.06	4.04E-02	ADT
BTNL8	7.568	0.367	7.83	0.292	3.96E-03	-1.20	4.04E-02	ADT
PCNA	4.117	0.251	4.306	0.242	3.96E-03	-1.14	4.04E-02	ADT
SYP	5.505	0.201	5.354	0.196	4.00E-03	1.11	4.07E-02	ADT
LILRA6	9.602	0.207	9.413	0.314	4.00E-03	1.14	4.07E-02	ADT
GTF2IRD1	5.186	0.15	5.077	0.129	4.00E-03	1.08	4.07E-02	ADT
SPPL2A	8.495	0.287	8.717	0.306	4.01E-03	-1.17	4.07E-02	ADT
SASS6	4.905	0.417	5.195	0.299	4.00E-03	-1.22	4.07E-02	ADT
NUDT3	7.197	0.197	7.375	0.293	4.01E-03	-1.13	4.07E-02	ADT
MIER2	5.482	0.137	5.38	0.125	4.01E-03	1.07	4.07E-02	ADT
PNPLA5	5.331	0.178	5.21	0.112	4.03E-03	1.09	4.09E-02	ADT
PEG3	3.82	0.132	3.724	0.116	4.04E-03	1.07	4.09E-02	ADT
SUV420H1	7.132	0.227	7.295	0.189	4.05E-03	-1.12	4.10E-02	ADT
HSPE1-MOB4	6.156	0.47	6.478	0.304	4.05E-03	-1.25	4.10E-02	ADT
DNAH3	3.621	0.097	3.549	0.089	4.06E-03	1.05	4.11E-02	ADT
PIK3R5	9.408	0.139	9.29	0.186	4.06E-03	1.09	4.11E-02	ADT
ENDOG	7.052	0.135	6.946	0.149	4.07E-03	1.08	4.11E-02	ADT
CTNS	5.835	0.302	6.098	0.423	4.08E-03	-1.20	4.12E-02	ADT
COL15A1	4.638	0.106	4.56	0.097	4.07E-03	1.06	4.12E-02	ADT
CPSF3L	7.011	0.101	7.096	0.134	4.08E-03	-1.06	4.12E-02	ADT
MKX	5.088	0.159	4.973	0.137	4.11E-03	1.08	4.14E-02	ADT
RAB33B	8.046	0.195	8.206	0.239	4.12E-03	-1.12	4.16E-02	ADT
COQ2	7.177	0.281	7.405	0.339	4.13E-03	-1.17	4.16E-02	ADT
HAP1	4.966	0.106	4.886	0.105	4.12E-03	1.06	4.16E-02	ADT
LMO2	6.134	0.152	6.259	0.189	4.13E-03	-1.09	4.16E-02	ADT
FAM149B1	5.018	0.227	5.182	0.192	4.14E-03	-1.12	4.16E-02	ADT
TMEM235	6.096	0.158	5.978	0.151	4.14E-03	1.09	4.16E-02	ADT
PHF15	6.523	0.173	6.681	0.265	4.15E-03	-1.12	4.17E-02	ADT
TET3	6.799	0.65	6.321	0.594	4.17E-03	1.39	4.19E-02	ADT
CELA1	4.791	0.147	4.672	0.178	4.17E-03	1.09	4.19E-02	ADT
CX3CR1	6.902	0.306	7.173	0.442	4.18E-03	-1.21	4.19E-02	ADT
LTBP2	6.175	0.112	6.09	0.116	4.19E-03	1.06	4.20E-02	ADT
PHF5A	6.14	0.23	6.307	0.202	4.21E-03	-1.12	4.21E-02	ADT
RTCA	6.866	0.295	7.07	0.204	4.22E-03	-1.15	4.22E-02	ADT

PXDC1	4.558	0.247	4.748	0.261	4.22E-03	-1.14	4.22E-02	ADT
PKN1	7.446	0.122	7.565	0.211	4.21E-03	-1.09	4.22E-02	ADT
DRAP1	8.774	0.566	8.367	0.482	4.21E-03	1.33	4.22E-02	ADT
INPP5K	7.67	0.167	7.804	0.194	4.22E-03	-1.10	4.22E-02	ADT
SERPINF1	4.691	0.125	4.588	0.157	4.22E-03	1.07	4.22E-02	ADT
RBP1	3.692	0.181	3.558	0.172	4.23E-03	1.10	4.22E-02	ADT
LRPAP1	7.61	0.15	7.726	0.16	4.23E-03	-1.08	4.22E-02	ADT
MYO15A	5.662	0.111	5.585	0.079	4.25E-03	1.05	4.23E-02	ADT
ZNF490	5.97	0.251	6.152	0.222	4.25E-03	-1.13	4.23E-02	ADT
PPIL2	6.88	0.188	7.027	0.209	4.25E-03	-1.11	4.23E-02	ADT
MMP3	3.588	0.131	3.707	0.197	4.25E-03	-1.09	4.23E-02	ADT
GLI1	5.626	0.097	5.55	0.11	4.25E-03	1.05	4.23E-02	ADT
STRC	5.169	0.098	5.099	0.078	4.25E-03	1.05	4.23E-02	ADT
FCRL5	5.09	0.33	4.857	0.264	4.27E-03	1.18	4.24E-02	ADT
DEFA6	3.74	0.223	3.917	0.255	4.28E-03	-1.13	4.25E-02	ADT
AIRE	6.783	0.167	6.668	0.117	4.28E-03	1.08	4.25E-02	ADT
PTCHD3	3.283	0.25	3.106	0.2	4.28E-03	1.13	4.25E-02	ADT
MAP3K9	5.601	0.105	5.512	0.141	4.29E-03	1.06	4.25E-02	ADT
FAM183A	4.266	0.223	4.103	0.201	4.32E-03	1.12	4.27E-02	ADT
PIN1	6.929	0.218	7.098	0.238	4.32E-03	-1.12	4.27E-02	ADT
SNX22	5.613	0.104	5.52	0.154	4.32E-03	1.07	4.27E-02	ADT
GPR6	6.503	0.235	6.336	0.193	4.32E-03	1.12	4.28E-02	ADT
DHFR	4.43	0.221	4.6	0.238	4.33E-03	-1.13	4.28E-02	ADT
STRN3	5.805	0.213	5.953	0.155	4.36E-03	-1.11	4.30E-02	ADT
GOLGA7B	4.997	0.143	4.892	0.131	4.36E-03	1.08	4.30E-02	ADT
SH3GL1	7.734	0.095	7.666	0.077	4.38E-03	1.05	4.31E-02	ADT
USP6	6.566	0.123	6.476	0.115	4.37E-03	1.06	4.31E-02	ADT
KREMEN2	7.307	0.146	7.201	0.128	4.38E-03	1.08	4.31E-02	ADT
OR13J1	7.421	0.257	7.219	0.29	4.37E-03	1.15	4.31E-02	ADT
SYT5	5.987	0.148	5.882	0.121	4.37E-03	1.08	4.31E-02	ADT
MCF2L	5.379	0.084	5.313	0.093	4.39E-03	1.05	4.31E-02	ADT
CASKIN2	5.664	0.124	5.574	0.114	4.39E-03	1.06	4.31E-02	ADT
SYT4	4.324	0.16	4.208	0.139	4.39E-03	1.08	4.31E-02	ADT
TMEM200C	5.481	0.2	5.327	0.214	4.38E-03	1.11	4.31E-02	ADT
PHB	6.84	0.22	7.011	0.24	4.40E-03	-1.13	4.32E-02	ADT
COL4A6	4.724	0.108	4.643	0.11	4.40E-03	1.06	4.32E-02	ADT
IL22	3.186	0.156	3.039	0.255	4.41E-03	1.11	4.32E-02	ADT
CYB561D1	6.423	0.133	6.517	0.112	4.40E-03	-1.07	4.32E-02	ADT
SNRNP200	7.927	0.18	8.062	0.179	4.41E-03	-1.10	4.32E-02	ADT
SIRPG	8.065	0.331	7.806	0.373	4.42E-03	1.20	4.32E-02	ADT
IGSF6	10.339	0.231	10.172	0.204	4.42E-03	1.12	4.33E-02	ADT
GIF	3.963	0.17	3.838	0.162	4.43E-03	1.09	4.33E-02	ADT
LMX1B	6.167	0.148	6.059	0.136	4.43E-03	1.08	4.33E-02	ADT
DPEP1	5.874	0.129	5.782	0.107	4.44E-03	1.07	4.34E-02	ADT
ANKRD13A	10.573	0.149	10.462	0.145	4.44E-03	1.08	4.34E-02	ADT
PFDN1	5.878	0.334	6.129	0.339	4.46E-03	-1.19	4.35E-02	ADT
METTL6	4.992	0.428	5.286	0.305	4.47E-03	-1.23	4.35E-02	ADT

SSTR5	6.236	0.128	6.139	0.131	4.47E-03	1.07	4.35E-02	ADT
PRMT2	6.459	0.195	6.616	0.236	4.49E-03	-1.11	4.37E-02	ADT
AAGAB	5.001	0.191	5.142	0.18	4.48E-03	-1.10	4.37E-02	ADT
KIF18B	5.752	0.144	5.645	0.142	4.48E-03	1.08	4.37E-02	ADT
AKR1C3	3.289	0.158	3.449	0.289	4.50E-03	-1.12	4.38E-02	ADT
MTCH1	8.837	0.133	8.945	0.165	4.50E-03	-1.08	4.38E-02	ADT
CARD10	6.202	0.106	6.121	0.114	4.51E-03	1.06	4.38E-02	ADT
ABTB1	10.732	0.202	10.579	0.209	4.51E-03	1.11	4.38E-02	ADT
PLIN4	6.742	0.258	6.553	0.245	4.53E-03	1.14	4.38E-02	ADT
GAS6	5.524	0.131	5.433	0.1	4.51E-03	1.07	4.38E-02	ADT
KCNQ2	5.701	0.138	5.605	0.106	4.52E-03	1.07	4.38E-02	ADT
IGIP	2.816	0.217	2.984	0.241	4.53E-03	-1.12	4.38E-02	ADT
GMPPB	6.006	0.117	6.105	0.156	4.54E-03	-1.07	4.38E-02	ADT
KCNG4	5.805	0.178	5.673	0.174	4.53E-03	1.10	4.38E-02	ADT
UFD1L	7.27	0.246	7.438	0.166	4.53E-03	-1.12	4.38E-02	ADT
PEMT	5.922	0.185	5.772	0.229	4.52E-03	1.11	4.38E-02	ADT
TRO	4.287	0.128	4.196	0.109	4.53E-03	1.07	4.38E-02	ADT
TNFRSF1A	10.48	0.163	10.354	0.179	4.55E-03	1.09	4.38E-02	ADT
RGL1	4.895	0.113	5.005	0.195	4.54E-03	-1.08	4.39E-02	ADT
MGRN1	8.461	0.269	8.274	0.21	4.54E-03	1.14	4.39E-02	ADT
YIPF5	6.02	0.203	6.186	0.255	4.56E-03	-1.12	4.39E-02	ADT
VPS4B	7.516	0.227	7.675	0.18	4.56E-03	-1.12	4.39E-02	ADT
FAM125A	6.266	0.135	6.375	0.165	4.57E-03	-1.08	4.40E-02	ADT
SNTA1	6.201	0.13	6.107	0.119	4.57E-03	1.07	4.40E-02	ADT
ALDH3A1	5.488	0.119	5.401	0.112	4.58E-03	1.06	4.40E-02	ADT
HYDIN	3.937	0.096	3.868	0.084	4.58E-03	1.05	4.41E-02	ADT
PRCD	6.169	0.148	6.05	0.179	4.60E-03	1.09	4.42E-02	ADT
LPPR3	6.282	0.135	6.185	0.117	4.61E-03	1.07	4.42E-02	ADT
GAST	6.829	0.231	6.657	0.232	4.61E-03	1.13	4.42E-02	ADT
OR7A10	2.424	0.162	2.54	0.141	4.62E-03	-1.08	4.43E-02	ADT
ITGA7	5.231	0.098	5.153	0.118	4.63E-03	1.06	4.43E-02	ADT
FAM18B2	5.82	0.274	6.021	0.258	4.62E-03	-1.15	4.43E-02	ADT
RHD	4.923	0.244	5.116	0.285	4.63E-03	-1.14	4.43E-02	ADT
YIPF4	5.898	0.267	6.079	0.174	4.63E-03	-1.13	4.43E-02	ADT
SPTY2D1	4.941	0.217	5.092	0.167	4.65E-03	-1.11	4.45E-02	ADT
HSD17B7	5.226	0.324	5.454	0.263	4.66E-03	-1.17	4.45E-02	ADT
RDH12	4.877	0.134	4.776	0.138	4.66E-03	1.07	4.45E-02	ADT
GPR25	7.052	0.141	6.942	0.16	4.66E-03	1.08	4.46E-02	ADT
RPL3L	5.949	0.161	5.835	0.131	4.67E-03	1.08	4.46E-02	ADT
KRT8	6.756	0.273	6.954	0.25	4.70E-03	-1.15	4.47E-02	ADT
RALA	5.066	0.256	5.252	0.233	4.69E-03	-1.14	4.47E-02	ADT
SPSB2	6.804	0.152	6.695	0.133	4.70E-03	1.08	4.47E-02	ADT
PIGH	5.511	0.252	5.685	0.185	4.70E-03	-1.13	4.48E-02	ADT
SLC25A30	4.428	0.265	4.627	0.271	4.69E-03	-1.15	4.48E-02	ADT
EXD3	6.458	0.106	6.381	0.098	4.71E-03	1.05	4.48E-02	ADT
CAB39	8.595	0.294	8.794	0.199	4.72E-03	-1.15	4.49E-02	ADT
DNAJC8	6.424	0.298	6.641	0.275	4.73E-03	-1.16	4.49E-02	ADT

KCNK7	6.635	0.17	6.506	0.181	4.72E-03	1.09	4.49E-02	ADT
HSPA12B	4.308	0.61	3.846	0.647	4.73E-03	1.38	4.49E-02	ADT
USP1	5.559	0.255	5.74	0.22	4.74E-03	-1.13	4.50E-02	ADT
PRPF6	6.783	0.154	6.899	0.159	4.76E-03	-1.08	4.51E-02	ADT
RHPN1	6.283	0.128	6.19	0.118	4.77E-03	1.07	4.52E-02	ADT
RAB43	7.907	0.164	7.782	0.177	4.77E-03	1.09	4.52E-02	ADT
ZMYND12	3.936	0.138	4.035	0.126	4.79E-03	-1.07	4.54E-02	ADT
MLF2	9.676	0.17	9.825	0.249	4.80E-03	-1.11	4.54E-02	ADT
PDHA1	5.35	0.194	5.496	0.199	4.80E-03	-1.11	4.54E-02	ADT
MAPK8IP2	6.269	0.141	6.168	0.125	4.82E-03	1.07	4.56E-02	ADT
CNOT10	6.054	0.311	6.268	0.237	4.82E-03	-1.16	4.56E-02	ADT
MYH7	4.841	0.128	4.75	0.113	4.83E-03	1.07	4.56E-02	ADT
AGXT2L2	7.441	0.143	7.563	0.2	4.87E-03	-1.09	4.59E-02	ADT
CABYR	4.008	0.138	3.907	0.132	4.88E-03	1.07	4.60E-02	ADT
KLF9	5.933	0.207	5.78	0.206	4.88E-03	1.11	4.60E-02	ADT
SH3RF3	7.069	0.313	6.831	0.34	4.89E-03	1.18	4.60E-02	ADT
RGS13	3.731	0.197	3.871	0.17	4.88E-03	-1.10	4.60E-02	ADT
SETD1A	6.651	0.119	6.563	0.12	4.89E-03	1.06	4.60E-02	ADT
CHRM3	6.095	0.121	6.004	0.125	4.88E-03	1.07	4.60E-02	ADT
FAM185A	7.645	0.19	7.795	0.226	4.90E-03	-1.11	4.61E-02	ADT
TCTE1	5.17	0.191	5.027	0.197	4.90E-03	1.10	4.61E-02	ADT
DHDH	5.686	0.162	5.564	0.173	4.91E-03	1.09	4.61E-02	ADT
ERCC6	6.406	0.22	6.582	0.273	4.91E-03	-1.13	4.61E-02	ADT
MAPKAPK3	7.861	0.162	7.982	0.164	4.91E-03	-1.09	4.61E-02	ADT
ADAMTS14	5.551	0.114	5.469	0.103	4.92E-03	1.06	4.61E-02	ADT
GBA	8.123	0.329	8.38	0.38	4.93E-03	-1.19	4.62E-02	ADT
DHX40	5.969	0.253	6.15	0.229	4.95E-03	-1.13	4.63E-02	ADT
ROMO1	6.76	0.326	6.506	0.377	4.96E-03	1.19	4.64E-02	ADT
PRPF3	7.225	0.211	7.369	0.156	4.99E-03	-1.10	4.67E-02	ADT
RNF130	9.681	0.237	9.841	0.165	4.99E-03	-1.12	4.67E-02	ADT
XCR1	5.54	0.249	5.729	0.272	5.00E-03	-1.14	4.67E-02	ADT
AUP1	8.622	0.119	8.737	0.208	5.00E-03	-1.08	4.68E-02	ADT
SLC44A2	10.857	0.129	10.766	0.114	5.01E-03	1.07	4.68E-02	ADT
YWHAZ	9.178	0.15	9.278	0.098	5.01E-03	-1.07	4.68E-02	ADT
GABPA	6.136	0.151	6.249	0.158	5.03E-03	-1.08	4.69E-02	ADT
SOX9	6.162	0.153	6.054	0.133	5.03E-03	1.08	4.69E-02	ADT
CD2BP2	7.055	0.122	7.163	0.186	5.04E-03	-1.08	4.70E-02	ADT
SEMA4G	4.921	0.119	4.837	0.099	5.05E-03	1.06	4.70E-02	ADT
PPM1J	5.513	0.112	5.43	0.116	5.05E-03	1.06	4.70E-02	ADT
KRTAP4-2	6.809	0.175	6.686	0.149	5.06E-03	1.09	4.70E-02	ADT
ZNF768	5.122	0.212	5.278	0.211	5.07E-03	-1.11	4.71E-02	ADT
VTI1A	6.252	0.17	6.366	0.114	5.07E-03	-1.08	4.71E-02	ADT
HDLBP	7.944	0.145	8.053	0.153	5.07E-03	-1.08	4.71E-02	ADT
CEBPA	6.889	0.184	6.748	0.204	5.06E-03	1.10	4.71E-02	ADT
MYO18A	6.703	0.199	6.873	0.279	5.06E-03	-1.13	4.71E-02	ADT
ERO1LB	5.358	0.267	5.559	0.282	5.08E-03	-1.15	4.71E-02	ADT
BTK	7.484	0.37	7.77	0.424	5.12E-03	-1.22	4.74E-02	ADT

KIAA0141	6.7	0.107	6.803	0.186	5.12E-03	-1.07	4.74E-02	ADT
RBMY1D	5.269	0.338	5.032	0.285	5.12E-03	1.18	4.74E-02	ADT
MAP4K4	8.766	0.255	8.569	0.291	5.13E-03	1.15	4.75E-02	ADT
TMEM88	10.239	0.347	9.993	0.305	5.15E-03	1.19	4.76E-02	ADT
RLTPR	6.298	0.159	6.178	0.173	5.16E-03	1.09	4.77E-02	ADT
TUB	5.109	0.135	5.012	0.127	5.17E-03	1.07	4.77E-02	ADT
TLN1	10.122	0.115	10.036	0.119	5.17E-03	1.06	4.78E-02	ADT
BET3L	3.675	0.215	3.529	0.158	5.18E-03	1.11	4.78E-02	ADT
PTPRT	4.934	0.098	4.863	0.093	5.18E-03	1.05	4.78E-02	ADT
ELAVL3	4.996	0.103	4.908	0.147	5.18E-03	1.06	4.78E-02	ADT
SAP25	7.84	0.239	7.67	0.21	5.19E-03	1.13	4.78E-02	ADT
NCAPG2	3.512	0.153	3.632	0.181	5.19E-03	-1.09	4.78E-02	ADT
DDX17	9.739	0.093	9.808	0.095	5.22E-03	-1.05	4.80E-02	ADT
TAPT1	5.848	0.248	6.017	0.186	5.22E-03	-1.12	4.81E-02	ADT
RGMA	6.284	0.135	6.181	0.152	5.23E-03	1.07	4.81E-02	ADT
ZNF564	6.312	0.328	6.529	0.207	5.23E-03	-1.16	4.81E-02	ADT
DCAF10	6.776	0.27	6.961	0.211	5.24E-03	-1.14	4.81E-02	ADT
EED	6.283	0.242	6.459	0.233	5.26E-03	-1.13	4.83E-02	ADT
MEMO1	5.998	0.224	6.149	0.162	5.27E-03	-1.11	4.83E-02	ADT
NT5M	5.663	0.122	5.564	0.159	5.26E-03	1.07	4.83E-02	ADT
PSMB4	8.249	0.202	8.393	0.181	5.26E-03	-1.10	4.83E-02	ADT
OSBP2	5.062	0.189	4.932	0.153	5.27E-03	1.09	4.83E-02	ADT
DCXR	7.839	0.215	8.018	0.291	5.30E-03	-1.13	4.85E-02	ADT
EGLN1	8.629	0.193	8.475	0.238	5.30E-03	1.11	4.85E-02	ADT
AWAT1	4.586	0.208	4.446	0.151	5.31E-03	1.10	4.86E-02	ADT
ZNF664-FAM101	5.597	0.153	5.49	0.131	5.33E-03	1.08	4.87E-02	ADT
MRPS10	4.9	0.358	5.138	0.24	5.33E-03	-1.18	4.88E-02	ADT
CCDC140	4.863	0.207	4.721	0.166	5.35E-03	1.10	4.88E-02	ADT
FAM22A	5.787	0.092	5.72	0.095	5.34E-03	1.05	4.88E-02	ADT
MMP19	4.489	0.148	4.386	0.122	5.35E-03	1.07	4.89E-02	ADT
F3	3.962	0.127	3.868	0.131	5.37E-03	1.07	4.90E-02	ADT
WDR37	7.362	0.181	7.492	0.173	5.37E-03	-1.09	4.90E-02	ADT
TRPM5	6.058	0.13	5.967	0.111	5.38E-03	1.07	4.90E-02	ADT
FAM101A	3.831	0.61	3.391	0.581	5.38E-03	1.36	4.90E-02	ADT
ZMYM1	3.849	0.244	4.03	0.255	5.38E-03	-1.13	4.90E-02	ADT
SRPR	8.444	0.172	8.565	0.151	5.39E-03	-1.09	4.90E-02	ADT
PHLDA1	4.279	0.094	4.204	0.118	5.39E-03	1.05	4.90E-02	ADT
OTUD7A	6.012	0.102	5.937	0.102	5.40E-03	1.05	4.91E-02	ADT
CRLF3	8.329	0.258	8.508	0.218	5.40E-03	-1.13	4.91E-02	ADT
TMSB4X	9.097	0.323	8.875	0.262	5.42E-03	1.17	4.92E-02	ADT
ST8SIA3	3.356	0.157	3.245	0.143	5.43E-03	1.08	4.92E-02	ADT
SPINK5	2.588	0.639	2.129	0.602	5.44E-03	1.37	4.93E-02	ADT
KCNA2	3.889	0.128	3.799	0.114	5.44E-03	1.06	4.93E-02	ADT
DERA	4.854	0.178	4.997	0.228	5.46E-03	-1.10	4.94E-02	ADT
TMEM55B	7.785	0.195	7.941	0.247	5.45E-03	-1.11	4.94E-02	ADT
JPH4	6.458	0.142	6.359	0.121	5.46E-03	1.07	4.94E-02	ADT
SDE2	6.947	0.268	7.133	0.226	5.47E-03	-1.14	4.94E-02	ADT

RNF168	6.229	0.222	6.394	0.233	5.46E-03	-1.12	4.94E-02	ADT
ERBB2IP	10.683	0.278	10.484	0.263	5.46E-03	1.15	4.94E-02	ADT
TNS4	4.858	0.117	4.767	0.139	5.48E-03	1.07	4.94E-02	ADT
PNMT	5.125	0.254	4.956	0.179	5.47E-03	1.12	4.94E-02	ADT
LYSMD1	4.88	0.217	5.04	0.223	5.48E-03	-1.12	4.94E-02	ADT
LEPRE1	5.631	0.173	5.769	0.218	5.46E-03	-1.10	4.94E-02	ADT
ZBTB7B	7.321	0.135	7.423	0.149	5.49E-03	-1.07	4.94E-02	ADT
SLC28A1	4.439	0.123	4.352	0.11	5.48E-03	1.06	4.94E-02	ADT
HDGFRP2	7.178	0.098	7.1	0.121	5.50E-03	1.06	4.96E-02	ADT
ING3	7.523	0.247	7.706	0.261	5.51E-03	-1.14	4.96E-02	ADT
GK5	4.123	0.288	4.352	0.36	5.52E-03	-1.17	4.97E-02	ADT
SETD6	6.176	0.121	6.087	0.124	5.53E-03	1.06	4.97E-02	ADT
FOXB2	6.752	0.134	6.657	0.125	5.53E-03	1.07	4.97E-02	ADT
ZFYVE26	6.372	0.297	6.598	0.331	5.54E-03	-1.17	4.97E-02	ADT
PRELP	4.991	0.118	4.897	0.146	5.55E-03	1.07	4.98E-02	ADT
RBBP5	6.414	0.206	6.553	0.155	5.55E-03	-1.10	4.98E-02	ADT
TENC1	5.301	0.113	5.223	0.092	5.54E-03	1.06	4.98E-02	ADT
SLC36A1	8.566	0.501	8.921	0.456	5.56E-03	-1.28	4.99E-02	ADT
HVCN1	8.854	0.199	9.037	0.327	5.57E-03	-1.14	4.99E-02	ADT
SRRM4	5.276	0.109	5.194	0.118	5.57E-03	1.06	4.99E-02	ADT
IZUMO4	6.7	0.103	6.617	0.133	5.58E-03	1.06	4.99E-02	ADT
PABPN1	7.944	0.149	8.056	0.164	5.60E-03	-1.08	5.00E-02	ADT
HIC1	6.771	0.019	6.506	0.166	1.58E-12	1.20	2.80E-08	ID
PRDM8	7.443	0.03	7.129	0.19	2.64E-11	1.24	2.34E-07	ID
FOXO1	7.599	0.028	7.385	0.162	1.99E-08	1.16	1.17E-04	ID
P2RY11	5.784	0.035	5.531	0.176	2.96E-08	1.19	1.31E-04	ID
SOLH	6.71	0.018	6.575	0.108	3.95E-08	1.10	1.40E-04	ID
SLC2A14	9.298	0.036	9.564	0.227	8.65E-08	-1.20	2.55E-04	ID
ARMC10	5.476	0.038	5.77	0.27	1.27E-07	-1.23	3.21E-04	ID
TOR3A	5.276	0.029	5.485	0.19	1.93E-07	-1.16	3.80E-04	ID
KLF16	7.918	0.038	7.666	0.181	1.93E-07	1.19	4.27E-04	ID
SRD5A2	4.449	0.028	4.251	0.181	2.74E-07	1.15	4.85E-04	ID
FKBP1A	9.12	0.038	8.88	0.184	3.29E-07	1.18	5.29E-04	ID
UNC5CL	5.572	0.018	5.438	0.138	7.18E-07	1.10	9.77E-04	ID
GNA14	3.962	0.026	3.792	0.167	7.85E-07	1.13	9.91E-04	ID
ACOT9	7.394	0.103	8.033	0.413	7.14E-07	-1.56	1.05E-03	ID
CHRNA9	3.703	0.034	3.925	0.226	1.48E-06	-1.17	1.75E-03	ID
COX6B2	5.508	0.036	5.299	0.152	1.63E-06	1.16	1.80E-03	ID
GDI2	8.709	0.057	9.066	0.191	1.87E-06	-1.28	1.83E-03	ID
LEUTX	4.445	0.043	4.69	0.205	1.78E-06	-1.19	1.85E-03	ID
AZU1	6.529	0.032	6.347	0.162	2.06E-06	1.13	1.92E-03	ID
MMP12	2.52	0.016	2.663	0.165	2.50E-06	-1.10	2.21E-03	ID
FOXA1	4.939	0.026	4.797	0.121	2.70E-06	1.10	2.27E-03	ID
TOR1A	7.696	0.069	8.067	0.266	4.73E-06	-1.29	3.80E-03	ID
FOXK1	7.176	0.025	7.034	0.151	5.66E-06	1.10	4.35E-03	ID
SOCS4	4.736	0.048	4.988	0.241	6.63E-06	-1.19	4.88E-03	ID
REG1A	3.882	0.05	4.151	0.17	7.10E-06	-1.20	5.02E-03	ID

PHOX2A	5.385	0.013	5.264	0.151	7.44E-06	1.09	5.06E-03	ID
DBR1	4.095	0.055	4.373	0.244	7.90E-06	-1.21	5.17E-03	ID
LRRC6	7.779	0.062	7.118	0.837	8.37E-06	1.58	5.29E-03	ID
DCUN1D5	4.143	0.05	4.396	0.228	9.16E-06	-1.19	5.40E-03	ID
IMMP1L	3.614	0.04	3.828	0.229	9.12E-06	-1.16	5.56E-03	ID
SNAI2	3.389	0.054	3.096	0.176	9.96E-06	1.23	5.68E-03	ID
JMJD7	6.725	0.06	7.02	0.267	1.13E-05	-1.23	6.07E-03	ID
OSR1	5.49	0.047	5.244	0.152	1.21E-05	1.19	6.09E-03	ID
WFDC6	3.485	0.066	3.823	0.228	1.11E-05	-1.26	6.14E-03	ID
UFSP2	3.845	0.05	4.096	0.25	1.20E-05	-1.19	6.26E-03	ID
COL20A1	6.439	0.025	6.317	0.122	1.35E-05	1.09	6.63E-03	ID
PSKH2	4.537	0.047	4.305	0.229	1.45E-05	1.17	6.91E-03	ID
LRRC37B	6.623	0.041	6.823	0.2	1.58E-05	-1.15	7.34E-03	ID
RAD51B	4.588	0.059	4.868	0.257	1.70E-05	-1.21	7.51E-03	ID
PPM1G	7.629	0.037	7.435	0.221	1.68E-05	1.14	7.59E-03	ID
TRIP11	4.831	0.062	5.127	0.234	1.77E-05	-1.23	7.64E-03	ID
FHL3	8.7	0.055	8.386	0.381	2.00E-05	1.24	8.21E-03	ID
ENC1	5.413	0.094	4.89	0.26	1.96E-05	1.44	8.26E-03	ID
MTAP	3.976	0.044	4.207	0.272	2.35E-05	-1.17	9.25E-03	ID
NOD2	7.157	0.13	7.766	0.61	2.31E-05	-1.53	9.26E-03	ID
TOMM20	4.769	0.011	4.896	0.174	2.56E-05	-1.09	9.85E-03	ID
ENOPH1	4.661	0.06	4.934	0.255	2.79E-05	-1.21	1.05E-02	ID
MRPL49	6.665	0.097	7.121	0.329	3.00E-05	-1.37	1.06E-02	ID
C8orf44-SGK3	8.424	0.153	7.702	0.51	2.99E-05	1.65	1.08E-02	ID
SNRPD3	6.331	0.093	6.797	0.278	2.94E-05	-1.38	1.08E-02	ID
NDUFB3	6.957	0.165	6.218	0.628	3.61E-05	1.67	1.25E-02	ID
GP9	7.008	0.053	6.765	0.185	3.79E-05	1.18	1.26E-02	ID
G3BP1	7.221	0.037	7.486	0.36	3.74E-05	-1.20	1.27E-02	ID
EIF2S3	8.146	0.072	8.502	0.202	4.44E-05	-1.28	1.40E-02	ID
PCSK6	5.319	0.05	5.1	0.185	4.39E-05	1.16	1.41E-02	ID
PHF20L1	8.784	0.062	8.511	0.273	4.36E-05	1.21	1.43E-02	ID
FUCA2	4.837	0.038	5.032	0.247	4.69E-05	-1.14	1.45E-02	ID
FAM171B	4.318	0.032	4.178	0.127	4.78E-05	1.10	1.46E-02	ID
PSMB11	4.202	0.057	3.957	0.203	5.31E-05	1.19	1.51E-02	ID
RAPGEF3	5.334	0.036	5.16	0.102	5.26E-05	1.13	1.52E-02	ID
SIRT4	3.909	0.03	4.067	0.204	5.18E-05	-1.12	1.53E-02	ID
GZMB	5.288	0.086	5.786	0.661	5.16E-05	-1.41	1.55E-02	ID
TMTC1	4.423	0.052	4.856	0.615	5.81E-05	-1.35	1.63E-02	ID
SCXA	6.628	0.058	6.371	0.288	5.98E-05	1.19	1.65E-02	ID
PCMT1	8.37	0.124	8.96	0.349	6.16E-05	-1.51	1.68E-02	ID
TRAM2	6.574	0.043	6.343	0.306	6.71E-05	1.17	1.80E-02	ID
ZFP90	4.178	0.094	4.572	0.369	7.12E-05	-1.31	1.88E-02	ID
M6PR	6.723	0.054	7.039	0.436	7.70E-05	-1.24	1.92E-02	ID
C1orf212	6.049	0.065	5.777	0.227	7.68E-05	1.21	1.94E-02	ID
FAM204A	5.784	0.06	6.062	0.345	7.59E-05	-1.21	1.94E-02	ID
TGIF1	6.025	0.038	5.864	0.174	7.56E-05	1.12	1.97E-02	ID
CAMTA1	5.095	0.025	4.992	0.114	8.57E-05	1.07	2.11E-02	ID

EFNB1	5.446	0.04	5.282	0.138	8.72E-05	1.12	2.11E-02	ID
TLK1	6.763	0.049	6.964	0.205	9.43E-05	-1.15	2.25E-02	ID
FGF4	5.843	0.017	5.743	0.141	9.67E-05	1.07	2.28E-02	ID
CCDC23	6.43	0.209	5.548	0.638	1.06E-04	1.84	2.46E-02	ID
UBE2G2	5.643	0.047	5.85	0.259	1.10E-04	-1.15	2.52E-02	ID
SIRT3	7.354	0.024	7.247	0.138	1.15E-04	1.08	2.56E-02	ID
WBP1L	7.399	0.039	7.234	0.198	1.14E-04	1.12	2.59E-02	ID
FCER1G	9.393	0.083	9.783	0.519	1.21E-04	-1.31	2.68E-02	ID
TREML4	5.311	0.261	6.437	1.421	1.30E-04	-2.18	2.84E-02	ID
LMO3	4.07	0.032	3.945	0.118	1.37E-04	1.09	2.96E-02	ID
CT47A1	4.819	0.03	4.7	0.128	1.39E-04	1.09	2.97E-02	ID
STYX	6.7	0.073	6.985	0.289	1.44E-04	-1.22	3.04E-02	ID
ZNF420	3.308	0.051	3.523	0.274	1.53E-04	-1.16	3.17E-02	ID
MYOG	6.396	0.031	6.532	0.178	1.65E-04	-1.10	3.24E-02	ID
EIF2B2	4.93	0.085	5.257	0.304	1.67E-04	-1.25	3.24E-02	ID
ANP32B	6.388	0.054	6.612	0.284	1.61E-04	-1.17	3.24E-02	ID
NAPG	6.044	0.064	6.29	0.242	1.58E-04	-1.19	3.24E-02	ID
ACTB	12.555	0.017	12.473	0.114	1.65E-04	1.06	3.27E-02	ID
F12	6.213	0.016	6.129	0.121	1.61E-04	1.06	3.27E-02	ID
PPIB	9.572	0.046	9.753	0.148	1.71E-04	-1.13	3.29E-02	ID
CCDC30	3.89	0.046	3.678	0.294	1.74E-04	1.16	3.31E-02	ID
KIAA0754	4.532	0.016	4.683	0.236	1.76E-04	-1.11	3.32E-02	ID
GTF3A	5.249	0.049	5.489	0.347	2.00E-04	-1.18	3.61E-02	ID
OR10Z1	3.491	0.081	3.185	0.28	1.99E-04	1.24	3.62E-02	ID
ZNF587	7.592	0.074	7.882	0.232	1.95E-04	-1.22	3.64E-02	ID
KIR3DL1	4.971	0.089	5.359	0.529	2.04E-04	-1.31	3.64E-02	ID
ESRRB	4.345	0.045	4.176	0.177	1.98E-04	1.12	3.64E-02	ID
NACC2	7.583	0.076	7.263	0.195	2.09E-04	1.25	3.66E-02	ID
BORA	3.518	0.054	3.734	0.277	2.12E-04	-1.16	3.67E-02	ID
BTBD8	3.949	0.132	4.445	0.505	2.09E-04	-1.41	3.69E-02	ID
SAFB	7.654	0.035	7.794	0.099	2.23E-04	-1.10	3.82E-02	ID
OST4	9.087	0.059	8.86	0.273	2.27E-04	1.17	3.85E-02	ID
NKAIN2	4.122	0.026	4.008	0.16	2.40E-04	1.08	4.04E-02	ID
NEK9	6.357	0.068	6.604	0.258	2.62E-04	-1.19	4.36E-02	ID
LY75	7.726	0.151	7.173	0.515	2.69E-04	1.47	4.45E-02	ID
N4BP2L1	4.372	0.054	4.574	0.246	2.83E-04	-1.15	4.63E-02	ID
EDIL3	3.805	0.045	3.64	0.135	2.93E-04	1.12	4.75E-02	ID
CPO	3.21	0.058	3.438	0.153	2.97E-04	-1.17	4.78E-02	ID
CADM1	3.715	0.043	3.868	0.157	3.09E-04	-1.11	4.91E-02	ID
IL10	3.865	0.055	4.062	0.199	3.14E-04	-1.15	4.96E-02	ID
FAM123A	5.591	0.045	5.422	0.132	3.32E-04	1.12	5.14E-02	ID
ARHGAP17	6.896	0.085	7.199	0.309	3.30E-04	-1.23	5.16E-02	ID
LGALS9	10.791	0.094	10.457	0.391	3.81E-04	1.26	5.85E-02	ID
STOM	6.535	0.033	6.772	0.391	3.95E-04	-1.18	5.97E-02	ID
ASNSD1	5.992	0.175	5.303	0.423	3.93E-04	1.61	5.99E-02	ID
ITGB1	7.448	0.043	7.79	0.57	4.11E-04	-1.27	6.15E-02	ID
ZNF804B	3.939	0.042	3.794	0.17	4.32E-04	1.11	6.41E-02	ID

RPS3	7.774	0.066	7.531	0.324	4.35E-04	1.18	6.41E-02	ID
SLC9A6	5.721	0.068	5.975	0.176	4.50E-04	-1.19	6.58E-02	ID
STARD4	3.194	0.077	3.475	0.372	4.55E-04	-1.22	6.60E-02	ID
PFN2	4.951	0.056	4.754	0.248	4.96E-04	1.15	7.12E-02	ID
ARTN	6.151	0.011	6.061	0.153	5.05E-04	1.06	7.14E-02	ID
MED17	5.627	0.098	6	0.232	5.04E-04	-1.30	7.18E-02	ID
ZNF180	5.562	0.083	5.894	0.178	5.19E-04	-1.26	7.28E-02	ID
PHF21A	11.938	0.064	11.723	0.222	5.26E-04	1.16	7.32E-02	ID
CPNE8	3.509	0.049	3.688	0.243	5.36E-04	-1.13	7.40E-02	ID
EXPH5	3.761	0.006	3.831	0.122	5.80E-04	-1.05	7.95E-02	ID
DAOA	2.794	0.017	2.865	0.111	6.22E-04	-1.05	8.46E-02	ID
CCDC115	5.614	0.065	5.849	0.155	6.43E-04	-1.18	8.62E-02	ID
NCF2	11.404	0.027	11.51	0.159	6.41E-04	-1.08	8.65E-02	ID
NDUFC1	4.29	0.044	4.436	0.179	6.75E-04	-1.11	8.97E-02	ID
IDS	10.361	0.066	10.137	0.181	7.03E-04	1.17	9.28E-02	ID
ZNF43	4.284	0.052	4.478	0.287	7.10E-04	-1.14	9.29E-02	ID
RBMXL2	6.516	0.052	6.342	0.151	7.54E-04	1.13	9.53E-02	ID
CFC1	6.334	0.013	6.256	0.135	7.33E-04	1.06	9.53E-02	ID
ST8SIA4	5.5	0.121	5.956	0.69	7.44E-04	-1.37	9.53E-02	ID
LGSN	2.74	0.112	3.119	0.303	7.51E-04	-1.30	9.55E-02	ID
SUV420H1	7.098	0.061	7.295	0.189	7.43E-04	-1.15	9.59E-02	ID
RHPN1	6.304	0.035	6.19	0.118	7.78E-04	1.08	9.62E-02	ID
PER2	6.051	0.031	5.943	0.151	7.75E-04	1.08	9.65E-02	ID
SATL1	3.152	0.051	2.986	0.148	8.09E-04	1.12	9.67E-02	ID
XBP1	6.287	0.028	6.463	0.308	8.01E-04	-1.13	9.70E-02	ID
NDUFS8	5.697	0.063	5.49	0.181	7.91E-04	1.15	9.71E-02	ID
CHCHD7	3.682	0.096	4.01	0.244	7.75E-04	-1.26	9.71E-02	ID
ZFP36L1	9.544	0.071	9.317	0.248	8.09E-04	1.17	9.73E-02	ID
DDX6	8.14	0.041	8.274	0.173	7.99E-04	-1.10	9.74E-02	ID

Supplementary table 2. Differentially expressed miRNAs from ADU, ADT, and ID

miRNA	Mean (test sample)	SD (test sample)	Mean (HC)	SD (HC)	*p* value	Fold change	FDR	Phenotype
miR-34a	3.005	0.755	2.273	0.543	6.47E-06	1.66	5.48E-03	ADU
miR-936	1.34	0.248	1.628	0.375	1.44E-04	-1.22	2.44E-02	ADU
miR-127-3p	3.014	1.315	1.995	0.718	9.03E-05	2.03	2.55E-02	ADU
miR-409-3p	3.766	1.086	2.871	0.842	1.28E-04	1.86	2.70E-02	ADU
miR-933	3.147	0.644	2.57	0.658	1.93E-04	1.49	2.73E-02	ADU
miR-379	2.106	0.725	1.566	0.453	2.27E-04	1.45	2.75E-02	ADU
miR-494	4.193	0.972	3.396	0.854	2.60E-04	1.74	2.75E-02	ADU
miR-551a	3.438	0.825	2.713	0.689	7.40E-05	1.65	3.13E-02	ADU
miR-138-1-star	2.599	0.32	3.213	0.399	1.59E-09	-1.53	1.35E-06	ADT
miR-198	1.518	0.352	3.016	1.345	2.29E-08	-2.82	9.68E-06	ADT
miR-944	2.193	0.329	2.688	0.302	5.11E-08	-1.41	1.44E-05	ADT
miR-1234	3.201	0.359	3.956	0.699	2.22E-07	-1.69	4.70E-05	ADT
miR-16	13.558	0.142	13.361	0.131	3.01E-07	1.15	5.09E-05	ADT
miR-551b-star	4.343	0.999	5.704	0.977	6.80E-07	-2.57	9.60E-05	ADT
let-7i-star	3.889	0.658	2.963	0.755	1.19E-06	1.90	1.44E-04	ADT
miR-130a	5.812	0.833	4.68	0.972	2.85E-06	2.19	3.01E-04	ADT
miR-337-3p	2.76	0.385	3.375	0.612	3.40E-06	-1.53	3.20E-04	ADT
miR-551a	3.794	0.928	2.713	0.689	3.79E-06	2.12	3.21E-04	ADT
miR-34a	3.321	0.95	2.273	0.543	5.32E-06	2.07	4.10E-04	ADT
miR-193a-3p	2.839	0.746	2.016	0.559	9.84E-06	1.77	6.94E-04	ADT
miR-214	3.271	0.391	3.796	0.521	1.15E-05	-1.44	7.50E-04	ADT
miR-1285	4.183	0.579	3.412	0.778	1.45E-05	1.71	8.77E-04	ADT
miR-30c-1-star	5.168	0.456	4.506	0.73	2.03E-05	1.58	1.15E-03	ADT
miR-933	3.315	0.664	2.57	0.658	2.56E-05	1.68	1.28E-03	ADT
miR-92a-2-star	1.936	0.341	2.344	0.394	2.56E-05	-1.33	1.35E-03	ADT
miR-595	1.995	0.464	2.573	0.602	3.25E-05	-1.49	1.53E-03	ADT
miR-181c-star	5.615	0.543	4.942	0.75	6.00E-05	1.59	2.54E-03	ADT
miR-432	3.305	1.225	4.967	1.955	5.73E-05	-3.16	2.55E-03	ADT
miR-135b-star	2.662	0.368	3.041	0.347	7.43E-05	-1.30	3.00E-03	ADT
miR-15a	10.07	0.302	9.528	0.723	8.45E-05	1.46	3.25E-03	ADT
miR-181d	4.748	0.421	4.196	0.671	9.49E-05	1.47	3.35E-03	ADT
miR-17-star	6.892	0.342	6.293	0.802	9.33E-05	1.51	3.44E-03	ADT
miR-664-star	5.339	0.402	4.705	0.858	1.37E-04	1.55	4.65E-03	ADT
miR-503	6.197	0.314	5.603	0.843	1.60E-04	1.51	5.20E-03	ADT
miR-1262	1.755	0.414	2.207	0.522	1.80E-04	-1.37	5.45E-03	ADT
miR-921	2.016	0.367	2.465	0.562	1.80E-04	-1.37	5.64E-03	ADT
miR-331-3p	5.667	0.331	5.089	0.835	2.20E-04	1.49	6.01E-03	ADT
miR-425	11.653	0.156	11.463	0.245	2.14E-04	1.14	6.04E-03	ADT
miR-642	1.451	0.344	1.81	0.404	2.07E-04	-1.28	6.05E-03	ADT
miR-422a	4.351	0.576	3.69	0.836	2.55E-04	1.58	6.55E-03	ADT
miR-1288	3.308	0.422	3.732	0.474	2.55E-04	-1.34	6.75E-03	ADT
miR-494	4.288	0.977	3.396	0.854	2.77E-04	1.86	6.89E-03	ADT
miR-129-3p	2.32	0.435	2.742	0.463	3.02E-04	-1.34	7.31E-03	ADT

miR-379	2.408	1.047	1.566	0.453	3.22E-04	1.79	7.36E-03	ADT
miR-367-star	1.255	0.246	1.476	0.211	3.14E-04	-1.17	7.40E-03	ADT
miR-127-3p	3.106	1.372	1.995	0.718	3.58E-04	2.16	7.40E-03	ADT
miR-146b-5p	6.133	0.714	5.32	1.059	3.38E-04	1.76	7.53E-03	ADT
miR-635	1.718	0.344	2.044	0.355	3.56E-04	-1.25	7.55E-03	ADT
miR-936	1.323	0.29	1.628	0.375	3.50E-04	-1.24	7.60E-03	ADT
miR-574-3p	8.967	0.548	9.436	0.422	3.94E-04	-1.38	7.76E-03	ADT
miR-140-5p	7.381	0.479	6.687	1.017	3.94E-04	1.62	7.94E-03	ADT
miR-15a-star	2.883	0.654	2.331	0.487	4.30E-04	1.47	8.28E-03	ADT
miR-802	2.011	0.425	2.402	0.443	5.23E-04	-1.31	9.42E-03	ADT
miR-877	4.041	0.662	4.679	0.768	5.19E-04	-1.56	9.55E-03	ADT
miR-18a-star	4.234	0.509	3.71	0.671	5.08E-04	1.44	9.57E-03	ADT
miR-301a	3.46	1.055	2.601	0.76	5.86E-04	1.81	1.03E-02	ADT
miR-221-star	2.697	0.674	2.132	0.559	6.23E-04	1.48	1.08E-02	ADT
miR-1825	2.954	0.602	3.467	0.539	6.64E-04	-1.43	1.12E-02	ADT
miR-181a-2-star	5.197	0.441	4.689	0.73	6.82E-04	1.42	1.13E-02	ADT
miR-539	1.739	0.356	2.074	0.427	8.13E-04	-1.26	1.28E-02	ADT
miR-423-5p	9.247	0.593	9.74	0.511	7.88E-04	-1.41	1.28E-02	ADT
miR-27a	12.135	0.283	11.876	0.321	8.11E-04	1.20	1.30E-02	ADT
miR-188-3p	2.092	0.302	2.394	0.411	8.74E-04	-1.23	1.35E-02	ADT
miR-608	1.249	0.237	1.502	0.363	9.18E-04	-1.19	1.36E-02	ADT
miR-19a	5.045	0.518	4.453	0.881	9.17E-04	1.51	1.39E-02	ADT
miR-370	2.529	0.947	1.835	0.492	1.04E-03	1.62	1.52E-02	ADT
miR-662	2.298	0.445	2.762	0.671	1.07E-03	-1.38	1.53E-02	ADT
miR-152	3.624	1.005	2.847	0.812	1.37E-03	1.71	1.94E-02	ADT
miR-888-star	1.172	0.259	1.454	0.437	1.42E-03	-1.22	1.97E-02	ADT
miR-199b-5p	2.028	0.694	1.532	0.411	1.58E-03	1.41	2.09E-02	ADT
miR-380-star	1.509	0.311	1.754	0.275	1.53E-03	-1.19	2.09E-02	ADT
miR-196a-star	1.249	0.253	1.475	0.309	1.56E-03	-1.17	2.10E-02	ADT
miR-623	2.091	0.502	2.475	0.431	1.79E-03	-1.30	2.33E-02	ADT
miR-552	1.473	0.299	1.726	0.336	1.83E-03	-1.19	2.35E-02	ADT
miR-320a	11.398	0.417	11.724	0.394	1.97E-03	-1.25	2.49E-02	ADT
miR-331-5p	3.326	0.592	2.782	0.812	2.13E-03	1.46	2.66E-02	ADT
miR-589	2.443	0.569	2.037	0.402	2.17E-03	1.33	2.66E-02	ADT
miR-193b-star	1.909	0.433	2.298	0.579	2.38E-03	-1.31	2.88E-02	ADT
miR-505	4.198	0.765	3.562	0.899	2.61E-03	1.55	3.12E-02	ADT
miR-454	3.452	0.54	3.003	0.65	2.86E-03	1.37	3.37E-02	ADT
let-7b	13.068	0.19	13.222	0.217	2.92E-03	-1.11	3.39E-02	ADT
miR-938	2.768	0.533	3.15	0.443	3.03E-03	-1.30	3.42E-02	ADT
miR-142-5p	4.485	0.782	3.847	0.913	3.02E-03	1.56	3.46E-02	ADT
miR-1267	1.952	0.497	2.301	0.399	3.36E-03	-1.27	3.74E-02	ADT
miR-145	11.305	0.243	11.065	0.408	3.46E-03	1.18	3.76E-02	ADT
miR-766	6.845	0.444	7.211	0.547	3.42E-03	-1.29	3.76E-02	ADT
miR-889	1.322	0.26	1.515	0.251	3.53E-03	-1.14	3.78E-02	ADT
let-7b-star	1.85	0.257	2.126	0.495	3.90E-03	-1.21	4.13E-02	ADT
miR-143	11.009	0.245	10.776	0.405	4.29E-03	1.18	4.44E-02	ADT
miR-361-3p	2.504	0.581	2.126	0.377	4.25E-03	1.30	4.44E-02	ADT

miR-134	2.773	1.02	2.134	0.59	4.89E-03	1.56	4.99E-02	ADT
miR-198	1.528	0.427	3.016	1.345	6.45E-08	-2.80	5.47E-05	ID
miR-130a	5.877	0.548	4.68	0.972	3.13E-07	2.29	1.33E-04	ID
miR-138-1-star	2.643	0.384	3.213	0.399	1.72E-05	-1.48	4.85E-03	ID
miR-1262	1.62	0.376	2.207	0.522	2.31E-05	-1.50	4.88E-03	ID
miR-17-star	6.906	0.221	6.293	0.802	4.75E-05	1.53	8.04E-03	ID
miR-16	13.543	0.135	13.361	0.131	5.88E-05	1.13	8.30E-03	ID
miR-92b-star	4.053	0.498	3.308	0.824	1.17E-04	1.68	1.10E-02	ID
miR-423-5p	9.089	0.504	9.74	0.511	1.07E-04	-1.57	1.13E-02	ID
miR-574-3p	8.882	0.428	9.436	0.422	9.86E-05	-1.47	1.19E-02	ID
miR-551b-star	4.526	0.971	5.704	0.977	2.27E-04	-2.26	1.92E-02	ID
miR-432	3.037	1.537	4.967	1.955	2.93E-04	-3.81	2.26E-02	ID
miR-193b-star	1.807	0.368	2.298	0.579	3.70E-04	-1.41	2.61E-02	ID
miR-15a	10.007	0.262	9.528	0.723	5.69E-04	1.39	3.71E-02	ID
miR-613	1.331	0.21	1.576	0.27	7.01E-04	-1.19	4.24E-02	ID

Supplementary table 3. Functional annotation of differentially expressed genes (showing p-values from pathway enrichment analysis).

Canonical Pathway	ADU	ADT	ID
Integrin Signaling		9.67E-04	
Wnt/β-catenin Signaling		1.26E-03	
GP6 Signaling Pathway		1.62E-03	
Signaling by Rho Family GTPases		1.68E-03	
Amyotrophic Lateral Sclerosis Signaling		1.74E-03	
Actin Cytoskeleton Signaling		1.80E-03	
Role of Oct4 in Mammalian Embryonic Stem Cell Pluripotency		1.91E-03	
Paxillin Signaling		2.17E-03	
ILK Signaling		2.51E-03	
Renal Cell Carcinoma Signaling		6.04E-03	
Ephrin Receptor Signaling		6.49E-03	
Tight Junction Signaling		6.81E-03	
nNOS Signaling in Neurons		7.34E-03	
HIF1α Signaling		7.96E-03	
Hepatic Fibrosis / Hepatic Stellate Cell Activation		8.21E-03	
TGF-β Signaling		9.35E-03	
AMPK Signaling	1.36E-03		
Role of IL-17F in Allergic Inflammatory Airway Diseases	1.94E-03		
Heme Degradation	2.31E-03		
ERK/MAPK Signaling	2.31E-03		
Molybdenum Cofactor Biosynthesis	2.31E-03		
p38 MAPK Signaling	2.79E-03		
Calcium Signaling	3.34E-03		
ATM Signaling	3.44E-03		
Hypoxia Signaling in the Cardiovascular System	3.80E-03		
Activation of IRF by Cytosolic Pattern Recognition Receptors	8.33E-03		
Phagosome Maturation	9.70E-03		
Leukocyte Extravasation Signaling			3.02E-03
Autoimmune Thyroid Disease Signaling			3.84E-03
VEGF Signaling			5.11E-03

Supplementary table 4. Trancripiton factors with enriched (p-values) target genes in differentially expressed genes from 3 phenotypes.

Regulators	ADU	ADT	ID
HNF4A	6.10E-04	4.24E-06	
XBP1	2.85E-02	3.00E-04	
SCX		1.78E-03	8.73E-03
PAX1		2.12E-03	
SOX7		2.85E-03	
GLI3		3.45E-03	
NFYB		3.66E-03	
SRY		5.71E-03	
JMY		8.11E-03	
PURA		8.20E-03	
ELF5			4.22E-04
ELF1			6.21E-04
PITX2			1.55E-03
IRF4			3.24E-03
BACH2			3.26E-03
YAP1			4.90E-03
SIX2			6.75E-03
HOXA4			6.75E-03
TBPL2			6.75E-03
TBX18			6.75E-03
SIRT1			7.10E-03
NFE2L2			7.27E-03
NKX2-1			7.58E-03
FOXC1			8.73E-03

Supplementary table 5. Genes and their fold changes across three phenotypes from clusters/sub-cluste

Gene Symbol	Fold change ADU vs HC	Fold change ADT vs HC	Fold change ID vs HC	Cluster/sub-cluster
ADGRE1	-1.800	-1.414	-1.257	DDd
UTS2	-1.426	-1.739	-1.066	DDd
NTAN1	-1.365	-1.338	-1.358	DDd
ANXA1	-1.345	-1.150	-1.182	DDd
GSDMB	-1.344	-1.235	-1.324	DDd
DOK2	-1.336	-1.259	-1.087	DDd
TMEM14C	-1.328	-1.344	-1.172	DDd
CD300LF	-1.326	-1.261	-1.216	DDd
SCPEP1	-1.322	-1.325	-1.240	DDd
RNF5	-1.316	-1.380	-1.199	DDd
TRAPPC4	-1.316	-1.330	-1.245	DDd
ERP29	-1.302	-1.270	-1.272	DDd
ZSCAN29	-1.302	-1.204	-1.156	DDd
PSPH	-1.297	-1.367	-1.165	DDd
CTNS	-1.285	-1.200	-1.120	DDd
CD300LB	-1.285	-1.315	-1.241	DDd
CCDC189	-1.282	-1.293	-1.235	DDd
S100Z	-1.280	-1.114	-1.170	DDd
CRYZL1	-1.279	-1.117	-1.150	DDd
MLKL	-1.268	-1.248	-1.153	DDd
TRMT112	-1.262	-1.319	-1.222	DDd
TMEM14B	-1.258	-1.220	-1.134	DDd
ATP8B4	-1.251	-1.183	-1.243	DDd
RBX1	-1.248	-1.146	-1.174	DDd
XCL2	-1.247	-1.115	-1.132	DDd
SOCS4	-1.235	-1.309	-1.191	DDd
GOLGA5	-1.229	-1.165	-1.207	DDd
TTC9C	-1.229	-1.126	-1.153	DDd
FUCA1	-1.228	-1.223	-1.169	DDd
INPP5B	-1.227	-1.185	-1.137	DDd
ARL2BP	-1.227	-1.193	-1.201	DDd
AIG1	-1.220	-1.250	-1.083	DDd
PCBD2	-1.220	-1.174	-1.146	DDd
PIN1	-1.217	-1.124	-1.124	DDd
PPP2R1B	-1.216	-1.204	-1.082	DDd
DYNLRB1	-1.211	-1.165	-1.116	DDd
HAX1	-1.209	-1.295	-1.174	DDd
NUP85	-1.208	-1.177	-1.206	DDd
HSH2D	-1.205	-1.232	-1.106	DDd
LNX2	-1.204	-1.199	-1.106	DDd
MKKS	-1.203	-1.255	-1.131	DDd
RMDN1	-1.202	-1.189	-1.187	DDd
SSNA1	-1.201	-1.188	-1.127	DDd

HCCS	-1.199	-1.186	-1.187	DDd
SASS6	-1.199	-1.223	-1.090	DDd
SLC35D1	-1.198	-1.215	-1.135	DDd
EBP	-1.197	-1.150	-1.118	DDd
CDK2	-1.197	-1.213	-1.125	DDd
ADAT1	-1.197	-1.158	-1.146	DDd
CASP8AP2	-1.195	-1.280	-1.062	DDd
ZNF266	-1.193	-1.239	-1.153	DDd
EMC4	-1.193	-1.153	-1.146	DDd
VPS53	-1.191	-1.213	-1.165	DDd
TEFM	-1.191	-1.142	-1.110	DDd
RPL7L1	-1.191	-1.189	-1.141	DDd
PFKM	-1.189	-1.185	-1.131	DDd
SERTAD1	-1.188	-1.120	-1.004	DDd
CBFB	-1.187	-1.105	-1.094	DDd
ZNF140	-1.184	-1.196	-1.119	DDd
FAM63B	-1.184	-1.157	-1.102	DDd
ZNF234	-1.183	-1.131	-1.149	DDd
RNF115	-1.183	-1.213	-1.084	DDd
CYB5B	-1.183	-1.078	-1.052	DDd
CLEC7A	-1.182	-1.248	-1.140	DDd
CHRFAM7A	-1.181	-1.123	-1.078	DDd
CDC37	-1.180	-1.087	-1.103	DDd
PTER	-1.179	-1.100	-1.087	DDd
PAFAH2	-1.178	-1.183	-1.083	DDd
PSMD5	-1.178	-1.167	-1.168	DDd
TTC30B	-1.178	-1.130	-1.004	DDd
HIST1H2AC	-1.175	-1.254	-1.106	DDd
JMJD7-PLA2G4B	-1.175	-1.103	-1.105	DDd
IL18BP	-1.175	-1.143	-1.148	DDd
DNAJB4	-1.174	-1.189	-1.005	DDd
GALC	-1.173	-1.197	-1.113	DDd
CUTC	-1.173	-1.107	-1.059	DDd
DUS2	-1.170	-1.188	-1.162	DDd
KRTCAP2	-1.170	-1.140	-1.084	DDd
SLC36A1	-1.170	-1.279	-1.152	DDd
BLVRA	-1.170	-1.182	-1.136	DDd
SH3BGRL	-1.169	-1.069	-1.105	DDd
PIK3CB	-1.166	-1.234	-1.165	DDd
UCK1	-1.166	-1.103	-1.066	DDd
CIRBP	-1.165	-1.150	-1.128	DDd
MFAP3	-1.165	-1.254	-1.127	DDd
LACTB2	-1.165	-1.142	-1.058	DDd
DPEP2	-1.164	-1.117	-1.008	DDd
MIGA1	-1.163	-1.174	-1.103	DDd
CD300C	-1.162	-1.249	-1.136	DDd
MIEN1	-1.162	-1.147	-1.064	DDd

ANAPC4	-1.161	-1.223	-1.153	DDd
DHRS1	-1.161	-1.104	-1.010	DDd
ZHX1	-1.160	-1.071	-1.087	DDd
ACTR8	-1.160	-1.207	-1.087	DDd
TNFAIP8L2-SCN	-1.159	-1.187	-1.025	DDd
SCFD1	-1.158	-1.344	-1.151	DDd
PCIF1	-1.158	-1.148	-1.050	DDd
COX5A	-1.157	-1.149	-1.053	DDd
OS9	-1.157	-1.267	-1.120	DDd
EED	-1.157	-1.130	-1.091	DDd
KIZ	-1.157	-1.051	-1.088	DDd
ZDHHC16	-1.157	-1.169	-1.084	DDd
TRIM52	-1.157	-1.196	-1.095	DDd
GTPBP10	-1.157	-1.166	-1.067	DDd
ANGEL2	-1.155	-1.185	-1.088	DDd
FANCD2	-1.154	-1.193	-1.113	DDd
VDAC3	-1.154	-1.186	-1.139	DDd
RTCA	-1.154	-1.152	-1.142	DDd
DNAJC14	-1.154	-1.164	-1.113	DDd
ANAPC5	-1.153	-1.158	-1.097	DDd
METTL6	-1.153	-1.226	-1.075	DDd
ZMYM6	-1.152	-1.193	-1.139	DDd
ITGAV	-1.151	-1.206	-1.134	DDd
ARHGAP25	-1.151	-1.184	-1.083	DDd
MGST2	-1.150	-1.192	-1.076	DDd
ALKBH6	-1.150	-1.069	-1.011	DDd
AAMP	-1.149	-1.137	-1.064	DDd
DIP2A	-1.149	-1.070	-1.053	DDd
ASTE1	-1.149	-1.155	-1.065	DDd
AGGF1	-1.149	-1.094	-1.037	DDd
RHBDD2	-1.149	-1.150	-1.056	DDd
BTF3L4	-1.149	-1.142	-1.097	DDd
HM13	-1.148	-1.203	-1.055	DDd
TRIM21	-1.147	-1.223	-1.072	DDd
TVP23C	-1.146	-1.149	-1.141	DDd
DBT	-1.146	-1.207	-1.129	DDd
POLR2G	-1.145	-1.116	-1.117	DDd
KCTD6	-1.145	-1.146	-1.143	DDd
GOLGA1	-1.144	-1.067	-1.120	DDd
ADAM17	-1.144	-1.235	-1.113	DDd
ZNF615	-1.144	-1.072	-1.116	DDd
MAGED2	-1.143	-1.146	-1.138	DDd
ERO1B	-1.143	-1.149	-1.040	DDd
VPS33B	-1.142	-1.093	-1.091	DDd
GLB1	-1.142	-1.152	-1.061	DDd
ASH2L	-1.141	-1.171	-1.136	DDd
SLC25A46	-1.141	-1.196	-1.092	DDd

DESI2	-1.140	-1.215	-1.037	DDd
FICD	-1.138	-1.164	-1.105	DDd
PRMT9	-1.138	-1.184	-1.046	DDd
PPM1D	-1.138	-1.167	-1.123	DDd
ADRM1	-1.138	-1.170	-1.111	DDd
CLN3	-1.137	-1.183	-1.088	DDd
NEDD8	-1.137	-1.128	-1.109	DDd
CDK2AP2	-1.137	-1.063	-1.052	DDd
MTMR9	-1.137	-1.098	-1.093	DDd
ACBD5	-1.137	-1.163	-1.123	DDd
ORAI3	-1.134	-1.127	-1.006	DDd
IMPA1	-1.134	-1.158	-1.132	DDd
FBXO8	-1.133	-1.203	-1.118	DDd
RBM4	-1.132	-1.123	-1.119	DDd
CTR9	-1.132	-1.136	-1.114	DDd
DENND1B	-1.132	-1.219	-1.113	DDd
ADGRE2	-1.131	-1.202	-1.087	DDd
NDUFAF7	-1.131	-1.164	-1.129	DDd
MDP1	-1.131	-1.145	-1.008	DDd
IGIP	-1.131	-1.123	-1.075	DDd
RUFY1	-1.131	-1.163	-1.066	DDd
MCM9	-1.131	-1.156	-1.106	DDd
TCEANC2	-1.130	-1.213	-1.107	DDd
GTF2E1	-1.129	-1.224	-1.045	DDd
PEX12	-1.128	-1.209	-1.098	DDd
COX8A	-1.127	-1.101	-1.067	DDd
RAE1	-1.127	-1.110	-1.123	DDd
U2AF1L4	-1.127	-1.176	-1.083	DDd
MED7	-1.125	-1.186	-1.113	DDd
SPCS1	-1.125	-1.118	-1.091	DDd
SPRYD3	-1.125	-1.076	-1.014	DDd
CHCHD4	-1.125	-1.079	-1.023	DDd
ERCC6	-1.124	-1.130	-1.081	DDd
COPS6	-1.124	-1.169	-1.084	DDd
MTF2	-1.124	-1.228	-1.058	DDd
PTPA	-1.123	-1.153	-1.121	DDd
PSME3	-1.123	-1.295	-1.058	DDd
VRK3	-1.123	-1.176	-1.121	DDd
ELOF1	-1.123	-1.193	-1.022	DDd
SSU72	-1.123	-1.144	-1.027	DDd
TSTA3	-1.122	-1.113	-1.106	DDd
HDAC6	-1.122	-1.134	-1.120	DDd
LRSAM1	-1.122	-1.120	-1.080	DDd
RBM6	-1.121	-1.138	-1.109	DDd
STAMBP	-1.121	-1.160	-1.109	DDd
GLT8D1	-1.121	-1.152	-1.094	DDd
CISD2	-1.120	-1.212	-1.080	DDd

SCAMP1	-1.120	-1.189	-1.041	DDd
THNSL2	-1.119	-1.131	-1.067	DDd
F11R	-1.118	-1.213	-1.058	DDd
DCXR	-1.118	-1.132	-1.093	DDd
CRLF3	-1.117	-1.132	-1.064	DDd
KAT8	-1.117	-1.080	-1.082	DDd
BAK1	-1.117	-1.169	-1.071	DDd
SFI1	-1.117	-1.068	-1.003	DDd
DGUOK	-1.116	-1.163	-1.061	DDd
LCAT	-1.116	-1.078	-1.078	DDd
ELMOD2	-1.116	-1.231	-1.014	DDd
TBC1D10A	-1.115	-1.073	-1.015	DDd
TPMT	-1.113	-1.173	-1.053	DDd
WDR55	-1.113	-1.223	-1.107	DDd
FBXO34	-1.113	-1.106	-1.077	DDd
TBC1D20	-1.113	-1.103	-1.094	DDd
TMED6	-1.113	-1.101	-1.075	DDd
CFDP1	-1.113	-1.141	-1.091	DDd
ZNF621	-1.113	-1.120	-1.092	DDd
OR1L6	-1.113	-1.162	-1.053	DDd
TBCC	-1.112	-1.202	-1.069	DDd
FAM220A	-1.112	-1.133	-1.044	DDd
NIT1	-1.111	-1.174	-1.017	DDd
WDR37	-1.111	-1.094	-1.081	DDd
TMED4	-1.111	-1.175	-1.067	DDd
HMGCL	-1.110	-1.101	-1.031	DDd
DNAJC8	-1.110	-1.162	-1.100	DDd
LAMTOR1	-1.110	-1.152	-1.094	DDd
UBE2Q1	-1.110	-1.186	-1.086	DDd
DCAF5	-1.110	-1.089	-1.109	DDd
CHMP6	-1.110	-1.015	-1.032	DDd
GPANK1	-1.109	-1.143	-1.100	DDd
STARD3	-1.109	-1.078	-1.010	DDd
PROSC	-1.109	-1.153	-1.101	DDd
ACAD10	-1.109	-1.063	-1.106	DDd
RSBN1L	-1.108	-1.205	-1.091	DDd
NDUFC1	-1.107	-1.167	-1.106	DDd
RACGAP1	-1.107	-1.080	-1.100	DDd
NOL12	-1.106	-1.084	-1.056	DDd
PREB	-1.106	-1.123	-1.027	DDd
RAB6A	-1.105	-1.096	-1.050	DDd
BCAS3	-1.105	-1.141	-1.056	DDd
KLHDC10	-1.104	-1.107	-1.039	DDd
HMOX2	-1.103	-1.153	-1.056	DDd
PIP5K1A	-1.103	-1.180	-1.057	DDd
DUSP11	-1.103	-1.131	-1.081	DDd
FBXO42	-1.101	-1.167	-1.081	DDd

PLEKHJ1	-1.101	-1.054	-1.059	DDd
GGCX	-1.100	-1.138	-1.048	DDd
KDELR2	-1.100	-1.158	-1.063	DDd
GATAD1	-1.100	-1.147	-1.017	DDd
ORAI1	-1.100	-1.061	-1.068	DDd
LIX1L	-1.100	-1.134	-1.056	DDd
DAGLB	-1.100	-1.166	-1.072	DDd
RALGPS1	-1.098	-1.045	-1.094	DDd
SLC17A5	-1.098	-1.140	-1.035	DDd
GTF3C2	-1.097	-1.136	-1.038	DDd
ZNF768	-1.097	-1.114	-1.056	DDd
RALA	-1.097	-1.138	-1.080	DDd
CNOT10	-1.097	-1.160	-1.035	DDd
ATP5S	-1.097	-1.157	-1.057	DDd
BLOC1S1	-1.096	-1.140	-1.002	DDd
RHD	-1.096	-1.143	-1.020	DDd
APEX2	-1.096	-1.041	-1.022	DDd
NFS1	-1.095	-1.050	-1.084	DDd
ATP5D	-1.094	-1.035	-1.050	DDd
TRADD	-1.094	-1.117	-1.022	DDd
CCNL2	-1.094	-1.127	-1.037	DDd
MVB12A	-1.094	-1.078	-1.056	DDd
SCNM1	-1.093	-1.223	-1.010	DDd
DDOST	-1.092	-1.144	-1.050	DDd
PARK7	-1.092	-1.149	-1.078	DDd
SRPRA	-1.091	-1.087	-1.063	DDd
COPS7A	-1.091	-1.094	-1.008	DDd
COQ2	-1.091	-1.171	-1.087	DDd
DDX59	-1.090	-1.131	-1.032	DDd
KLHDC8B	-1.090	-1.266	-1.076	DDd
CXXC1	-1.089	-1.084	-1.046	DDd
KLHL36	-1.089	-1.149	-1.042	DDd
CERS2	-1.088	-1.147	-1.063	DDd
INTS10	-1.088	-1.158	-1.060	DDd
RPUSD3	-1.088	-1.120	-1.076	DDd
ZSCAN32	-1.087	-1.073	-1.018	DDd
TGS1	-1.087	-1.139	-1.053	DDd
KLHDC3	-1.087	-1.158	-1.045	DDd
IRF3	-1.087	-1.069	-1.064	DDd
U2AF2	-1.087	-1.103	-1.070	DDd
CCM2	-1.085	-1.146	-1.013	DDd
NDUFS1	-1.084	-1.153	-1.070	DDd
SLC25A3	-1.084	-1.156	-1.070	DDd
MED20	-1.084	-1.109	-1.033	DDd
SLC25A13	-1.084	-1.156	-1.023	DDd
PEX19	-1.083	-1.149	-1.082	DDd
COMMD2	-1.083	-1.143	-1.042	DDd

ARL6IP4	-1.083	-1.046	-1.080	DDd
OR5B3	-1.083	-1.142	-1.011	DDd
MPC2	-1.082	-1.141	-1.045	DDd
RAD9A	-1.081	-1.091	-1.058	DDd
TCTA	-1.081	-1.171	-1.058	DDd
SPOP	-1.081	-1.092	-1.003	DDd
USP21	-1.081	-1.137	-1.052	DDd
SMARCD2	-1.081	-1.099	-1.078	DDd
ZNF692	-1.080	-1.113	-1.025	DDd
ZNF10	-1.079	-1.132	-1.048	DDd
TNFSF13	-1.079	-1.145	-1.018	DDd
ZFPL1	-1.078	-1.047	-1.011	DDd
PMF1	-1.078	-1.128	-1.073	DDd
SASH3	-1.077	-1.133	-1.062	DDd
ZNF436	-1.077	-1.091	-1.070	DDd
DSTYK	-1.075	-1.103	-1.071	DDd
TTC14	-1.075	-1.184	-1.053	DDd
ARNT	-1.074	-1.146	-1.061	DDd
ANAPC13	-1.074	-1.221	-1.058	DDd
UNC119	-1.073	-1.098	-1.039	DDd
ELMOD3	-1.071	-1.106	-1.008	DDd
CREG1	-1.070	-1.174	-1.046	DDd
AKR1C3	-1.070	-1.117	-1.023	DDd
MUT	-1.069	-1.089	-1.023	DDd
PTPN7	-1.069	-1.139	-1.036	DDd
MTX1	-1.068	-1.140	-1.014	DDd
PRCC	-1.068	-1.160	-1.004	DDd
MICB	-1.067	-1.180	-1.061	DDd
MBD1	-1.067	-1.074	-1.034	DDd
CCDC121	-1.067	-1.094	-1.059	DDd
BAX	-1.066	-1.136	-1.027	DDd
PPP2R5D	-1.065	-1.096	-1.042	DDd
RNF4	-1.064	-1.142	-1.060	DDd
OR7A5	-1.064	-1.090	-1.058	DDd
TSSC1	-1.062	-1.104	-1.055	DDd
RBM5	-1.062	-1.097	-1.055	DDd
TP53BP1	-1.061	-1.145	-1.018	DDd
XPR1	-1.061	-1.124	-1.052	DDd
PSAP	-1.061	-1.082	-1.045	DDd
TSPYL1	-1.061	-1.086	-1.050	DDd
KCNMB3	-1.061	-1.074	-1.054	DDd
DHTKD1	-1.061	-1.108	-1.050	DDd
MUL1	-1.059	-1.109	-1.049	DDd
STRIP1	-1.059	-1.109	-1.053	DDd
OR7A10	-1.059	-1.084	-1.021	DDd
B3GNTL1	-1.058	-1.138	-1.052	DDd
TADA3	-1.058	-1.112	-1.035	DDd

DAZAP2	-1.057	-1.093	-1.048	DDd
CYP20A1	-1.057	-1.112	-1.048	DDd
PDHA1	-1.056	-1.106	-1.020	DDd
DHX16	-1.056	-1.100	-1.026	DDd
GMEB1	-1.056	-1.160	-1.024	DDd
IKBKB	-1.055	-1.089	-1.045	DDd
SLC35E2B	-1.053	-1.092	-1.032	DDd
CALM3	-1.052	-1.077	-1.005	DDd
RAP2B	-1.051	-1.106	-1.022	DDd
LYSMD1	-1.050	-1.117	-1.009	DDd
MLF2	-1.050	-1.109	-1.031	DDd
MAP2K2	-1.049	-1.107	-1.001	DDd
FAM134A	-1.049	-1.164	-1.036	DDd
CNPY3	-1.048	-1.089	-1.001	DDd
IP6K1	-1.047	-1.120	-1.028	DDd
MAPKAPK3	-1.045	-1.087	-1.001	DDd
GPBP1L1	-1.042	-1.129	-1.018	DDd
KDM4A	-1.041	-1.095	-1.006	DDd
ARPC4-TTLL3	-1.040	-1.121	-1.015	DDd
OGDH	-1.040	-1.111	-1.020	DDd
PCYT1A	-1.037	-1.157	-1.032	DDd
NFYC	-1.035	-1.069	-1.033	DDd
MEMO1	-1.034	-1.110	-1.016	DDd
ARF1	-1.032	-1.085	-1.013	DDd
RGL1	-1.031	-1.079	-1.011	DDd
CNPPD1	-1.030	-1.110	-1.023	DDd
NCAPG2	-1.027	-1.087	-1.026	DDd
RAB1B	-1.023	-1.088	-1.002	DDd
RRAGC	-1.022	-1.157	-1.010	DDd
USP4	-1.017	-1.150	-1.014	DDd
HRH1	-1.016	-1.094	-1.013	DDd
TRAPPC5	-1.010	-1.164	-1.007	DDd
CYB561D1	-1.010	-1.067	-1.001	DDd
MIR205HG	-1.009	-1.118	-1.003	DDd
PAX5	1.207	1.254	-1.084	UUD
PRKAR1A	1.173	1.050	-1.104	UUD
NEB	1.166	1.154	-1.028	UUD
GDF3	1.152	1.051	-1.071	UUD
BICDL2	1.099	1.120	-1.020	UUD
WNT3	1.094	1.110	-1.063	UUD
FREM1	1.092	1.053	-1.023	UUD
KDM4E	1.092	1.071	-1.042	UUD
FAM166A	1.090	1.165	-1.007	UUD
CELA1	1.087	1.086	-1.019	UUD
SCARB1	1.086	1.121	-1.021	UUD
FXYD4	1.082	1.103	-1.001	UUD
MVD	1.081	1.070	-1.003	UUD

OR1D2	1.075	1.212	-1.027	UUD
PNMT	1.073	1.124	-1.151	UUD
LZTS3	1.073	1.116	-1.025	UUD
ZNF512B	1.072	1.085	-1.010	UUD
IFNL2	1.072	1.103	-1.003	UUD
ZDHHC22	1.070	1.149	-1.036	UUD
MARCO	1.069	1.094	-1.002	UUD
ZNF358	1.068	1.073	-1.057	UUD
LOC149373	1.067	1.220	-1.214	UUD
SALL2	1.064	1.064	-1.009	UUD
EGFL7	1.061	1.145	-1.007	UUD
EXOC3L1	1.061	1.089	-1.002	UUD
MAPK12	1.060	1.084	-1.055	UUD
PPFIA3	1.060	1.073	-1.003	UUD
F2	1.058	1.078	-1.004	UUD
AEBP1	1.058	1.087	-1.038	UUD
HAPLN2	1.057	1.072	-1.028	UUD
WDR18	1.056	1.087	-1.016	UUD
PYCRL	1.056	1.131	-1.014	UUD
IGLL1	1.055	1.083	-1.019	UUD
CLIP3	1.055	1.070	-1.001	UUD
LZTS2	1.055	1.072	-1.056	UUD
SYN3	1.054	1.073	-1.010	UUD
PLEKHG4	1.054	1.081	-1.006	UUD
RGS11	1.053	1.078	-1.015	UUD
CT47A6	1.053	1.234	-1.033	UUD
PRELP	1.053	1.067	-1.014	UUD
VASN	1.053	1.095	-1.056	UUD
FAM83G	1.053	1.110	-1.055	UUD
SOX9	1.052	1.078	-1.013	UUD
CD40	1.051	1.127	-1.064	UUD
KCTD17	1.050	1.099	-1.028	UUD
COL13A1	1.050	1.079	-1.009	UUD
LMOD3	1.050	1.127	-1.013	UUD
IFNA2	1.049	1.166	-1.083	UUD
TGM2	1.048	1.087	-1.005	UUD
FBXL15	1.048	1.075	-1.040	UUD
LMX1B	1.046	1.078	-1.006	UUD
TMPRSS13	1.046	1.085	-1.025	UUD
IGFBP4	1.045	1.095	-1.003	UUD
TMOD2	1.045	1.329	-1.007	UUD
TJP3	1.045	1.058	-1.010	UUD
LEUTX	1.045	1.053	-1.185	UUD
FAIM	1.044	1.095	-1.061	UUD
SYCE1L	1.044	1.137	-1.113	UUD
ARSH	1.043	1.095	-1.052	UUD
MYOG	1.042	1.029	-1.099	UUD

DPYSL4	1.042	1.073	-1.027	UUD
TRPM5	1.042	1.065	-1.003	UUD
JPH4	1.042	1.071	-1.015	UUD
LARGE2	1.042	1.064	-1.010	UUD
PAK6	1.040	1.060	-1.018	UUD
KRT4	1.040	1.083	-1.011	UUD
GATA5	1.040	1.122	-1.010	UUD
BCAR1	1.039	1.078	-1.005	UUD
CRB3	1.039	1.078	-1.007	UUD
ACSBG1	1.038	1.069	-1.041	UUD
BPIFB1	1.038	1.084	-1.005	UUD
EME1	1.038	1.084	-1.028	UUD
MAP3K9	1.037	1.064	-1.014	UUD
ZCCHC12	1.037	1.094	-1.106	UUD
POU5F1	1.037	1.119	-1.023	UUD
ANXA13	1.036	1.088	-1.040	UUD
CCDC114	1.036	1.064	-1.004	UUD
NKX2-1	1.035	1.078	-1.045	UUD
CABYR	1.035	1.073	-1.003	UUD
KRTAP16-1	1.035	1.097	-1.008	UUD
PRPF40B	1.035	1.077	-1.073	UUD
KCNA2	1.033	1.064	-1.045	UUD
TLCD1	1.033	1.083	-1.005	UUD
GLB1L2	1.032	1.060	-1.028	UUD
KIF18B	1.032	1.077	-1.058	UUD
NKX2-3	1.032	1.097	-1.021	UUD
BSX	1.032	1.094	-1.017	UUD
THOP1	1.032	1.068	-1.052	UUD
NXNL1	1.032	1.109	-1.015	UUD
DMP1	1.030	1.092	-1.028	UUD
UMOD	1.030	1.075	-1.015	UUD
ALDH3A1	1.029	1.062	-1.008	UUD
MOB3B	1.028	1.096	-1.027	UUD
ARMCX2	1.027	1.063	-1.032	UUD
RIBC2	1.027	1.126	-1.021	UUD
STK32C	1.027	1.072	-1.030	UUD
PPP1R14A	1.026	1.110	-1.017	UUD
RPUSD1	1.026	1.089	-1.061	UUD
SELENOV	1.026	1.100	-1.010	UUD
ROR2	1.025	1.089	-1.045	UUD
WDR62	1.025	1.052	-1.001	UUD
R3HDML	1.025	1.097	-1.068	UUD
ONECUT3	1.023	1.092	-1.013	UUD
PRB4	1.022	1.103	-1.017	UUD
DHDH	1.022	1.088	-1.041	UUD
TNS2	1.017	1.056	-1.005	UUD
SMTNL2	1.017	1.072	-1.030	UUD

GDF6	1.017	1.112	-1.008	UUD
TRO	1.017	1.065	-1.009	UUD
TPM2	1.016	1.093	-1.105	UUD
HRAS	1.011	1.071	-1.018	UUD
YAP1	1.004	1.062	-1.022	UUD
HDGFRP2	1.004	1.056	-1.010	UUD
SMIM12	-1.045	1.009	1.207	DUU
TRAM2	-1.041	1.008	1.174	DUU
CCDC30	-1.032	1.007	1.158	DUU
SIRT3	-1.026	1.027	1.077	DUU
KLF16	-1.014	1.026	1.191	DUU
ERP27	-1.012	1.177	1.059	DUU
CAMTA1	-1.004	1.001	1.074	DUU
FLRT1	-1.003	1.111	1.098	DUU
USP6	-1.001	1.064	1.043	DUU
GABARAP	-1.001	1.053	1.038	DUU
UNC5CL	-1.001	1.027	1.097	DUU
TMTC1	1.100	-1.037	-1.350	UDD
NOD2	1.061	-1.053	-1.525	UDD
GBA	1.030	-1.195	-1.130	UDD
HIST1H2BO	1.030	-1.171	-1.009	UDD
MKRN1	1.025	-1.087	-1.027	UDD
SYAP1	1.022	-1.376	-1.429	UDD
SPTY2D1	1.020	-1.110	-1.096	UDD
FKBP7	1.018	-1.093	-1.035	UDD
SKIL	1.017	-1.170	-1.164	UDD
BTNL8	1.013	-1.199	-1.006	UDD
CADM1	1.009	-1.003	-1.112	UDD
JDP2	1.008	-1.138	-1.103	UDD
TOR3A	1.007	-1.056	-1.156	UDD
TUBB4B	1.005	-1.128	-1.137	UDD
KIR3DL1	1.003	-1.102	-1.309	UDD
PNLIP	1.003	-1.064	-1.117	UDD
USP19	1.001	-1.092	-1.004	UDD
CD9	-1.483	-1.324	1.006	DDU
ZNF25	-1.275	-1.467	1.092	DDU
CORO1A	-1.195	-1.092	1.041	DDU
NME6	-1.185	-1.156	1.034	DDU
TMEM81	-1.162	-1.056	1.038	DDU
CALCOCO1	-1.149	-1.188	1.002	DDU
TMEM38B	-1.142	-1.127	1.001	DDU
BTN3A1	-1.132	-1.189	1.071	DDU
CD300A	-1.122	-1.206	1.006	DDU
CENPQ	-1.121	-1.092	1.024	DDU
AMMECR1L	-1.120	-1.085	1.024	DDU
TXNDC12	-1.119	-1.138	1.016	DDU
RNF34	-1.119	-1.091	1.012	DDU

UPK3BL	-1.119	-1.211	1.013	DDU
PGBD2	-1.116	-1.156	1.055	DDU
CLK2	-1.103	-1.150	1.003	DDU
FAM185A	-1.103	-1.110	1.042	DDU
ZBTB43	-1.101	-1.068	1.002	DDU
GMPPA	-1.087	-1.103	1.013	DDU
ZBTB48	-1.086	-1.105	1.001	DDU
MRPL10	-1.083	-1.147	1.012	DDU
DHFR2	-1.080	-1.055	1.019	DDU
ZFYVE1	-1.076	-1.095	1.003	DDU
IP6K2	-1.075	-1.112	1.019	DDU
KRT8	-1.073	-1.147	1.030	DDU
ACOX3	-1.069	-1.061	1.150	DDU
SWSAP1	-1.068	-1.142	1.028	DDU
ZBTB49	-1.068	-1.120	1.015	DDU
RETSAT	-1.064	-1.142	1.010	DDU
PHF23	-1.064	-1.100	1.001	DDU
ATP5SL	-1.063	-1.141	1.028	DDU
CHST12	-1.062	-1.157	1.127	DDU
BRK1	-1.055	-1.163	1.118	DDU
DHDDS	-1.055	-1.175	1.018	DDU
MTCH1	-1.054	-1.078	1.019	DDU
RAC1	-1.052	-1.128	1.016	DDU
PISD	-1.051	-1.144	1.040	DDU
BAGE	-1.050	-1.125	1.081	DDU
ZMYND12	-1.049	-1.071	1.014	DDU
ATPIF1	-1.048	-1.126	1.098	DDU
RHOB	-1.047	-1.108	1.035	DDU
KPNA6	-1.045	-1.109	1.003	DDU
NECTIN3	-1.044	-1.084	1.031	DDU
WBP1L	-1.037	-1.049	1.121	DDU
TBC1D32	-1.035	-1.114	1.032	DDU
FHL3	-1.035	-1.006	1.243	DDU
CD2BP2	-1.035	-1.078	1.009	DDU
PSG3	-1.032	-1.149	1.012	DDU
GNB1	-1.030	-1.114	1.036	DDU
CDKL3	-1.030	-1.073	1.011	DDU
MGAT1	-1.029	-1.072	1.054	DDU
FAM20B	-1.028	-1.100	1.050	DDU
TMEM196	-1.027	-1.205	1.073	DDU
GPN2	-1.025	-1.071	1.026	DDU
OR2T29	-1.025	-1.120	1.024	DDU
CPSF3L	-1.025	-1.061	1.001	DDU
DCAF15	-1.022	-1.067	1.066	DDU
XCR1	-1.015	-1.140	1.018	DDU
CTSZ	-1.011	-1.097	1.048	DDU
OR4K2	-1.010	-1.135	1.063	DDU

KIAA0141	-1.007	-1.074	1.012	DDU
GPR32	-1.004	-1.134	1.011	DDU
MTMR14	-1.003	-1.110	1.008	DDU
CXCL8	1.471	1.186	1.154	UUu
PLXNB2	1.372	1.236	1.162	UUu
ROMO1	1.371	1.193	1.125	UUu
DRAP1	1.339	1.326	1.072	UUu
RPS11	1.302	1.234	1.113	UUu
ENTPD3	1.296	1.155	1.053	UUu
HCST	1.275	1.200	1.137	UUu
NSUN4	1.235	1.360	1.004	UUu
HSPA12B	1.220	1.377	1.104	UUu
DYSF	1.217	1.147	1.169	UUu
CD55	1.205	1.129	1.085	UUu
QPCT	1.203	1.155	1.087	UUu
TANC2	1.197	1.127	1.064	UUu
MAP4K4	1.195	1.146	1.182	UUu
MYL9	1.177	1.170	1.077	UUu
EXT1	1.170	1.291	1.170	UUu
RASSF3	1.170	1.207	1.131	UUu
ARHGAP24	1.165	1.121	1.117	UUu
ARL8A	1.159	1.088	1.106	UUu
INHBB	1.157	1.088	1.030	UUu
TPSB2	1.157	1.228	1.038	UUu
SUMO1	1.156	1.110	1.050	UUu
RAB43	1.156	1.091	1.117	UUu
SIPA1L1	1.153	1.162	1.132	UUu
FCRL5	1.151	1.175	1.135	UUu
TP53I11	1.143	1.112	1.081	UUu
OR3A3	1.139	1.190	1.118	UUu
LRP10	1.136	1.084	1.103	UUu
PARVB	1.134	1.122	1.015	UUu
HEBP2	1.132	1.067	1.129	UUu
TSPAN10	1.132	1.109	1.090	UUu
RIPK4	1.128	1.110	1.009	UUu
CARM1	1.125	1.097	1.080	UUu
STRN4	1.124	1.136	1.119	UUu
VPS37B	1.122	1.079	1.038	UUu
FNDC8	1.120	1.058	1.055	UUu
TRAPPC3L	1.119	1.106	1.051	UUu
FAM25BP	1.119	1.161	1.042	UUu
GPRC5B	1.118	1.083	1.045	UUu
ASGR2	1.113	1.077	1.025	UUu
B4GALT1	1.111	1.069	1.053	UUu
ASNA1	1.110	1.056	1.017	UUu
RBMS1	1.110	1.154	1.053	UUu
RRS1	1.110	1.171	1.046	UUu

GIPC3	1.108	1.089	1.030	UUu
GOLGA7	1.107	1.042	1.056	UUu
TMEM150B	1.107	1.054	1.021	UUu
GEMIN4	1.106	1.134	1.090	UUu
PLIN4	1.106	1.140	1.083	UUu
ADRA1D	1.106	1.122	1.102	UUu
SMAD6	1.105	1.224	1.001	UUu
NFIC	1.103	1.105	1.088	UUu
TONSL	1.102	1.134	1.087	UUu
FOXI1	1.101	1.028	1.033	UUu
GPR173	1.101	1.127	1.035	UUu
GNAZ	1.100	1.146	1.064	UUu
SETBP1	1.100	1.120	1.020	UUu
RPP25	1.095	1.115	1.073	UUu
PENK	1.094	1.100	1.078	UUu
MC1R	1.094	1.100	1.076	UUu
NAGPA	1.094	1.156	1.056	UUu
TCTE1	1.094	1.104	1.059	UUu
RGS9BP	1.093	1.131	1.002	UUu
KLF9	1.092	1.112	1.009	UUu
LCE1A	1.092	1.150	1.083	UUu
NO	1.091	1.131	1.026	UUu
APOA1	1.089	1.097	1.078	UUu
MAP6	1.089	1.111	1.041	UUu
TMSB4X	1.089	1.166	1.022	UUu
SNPH	1.089	1.138	1.075	UUu
SP6	1.089	1.097	1.066	UUu
MYLK2	1.088	1.105	1.010	UUu
LENG9	1.088	1.158	1.037	UUu
C16orf82	1.088	1.087	1.085	UUu
DPF1	1.087	1.096	1.060	UUu
DLG4	1.086	1.064	1.006	UUu
FADS3	1.085	1.097	1.073	UUu
PHC2	1.084	1.024	1.061	UUu
CEBPA	1.084	1.103	1.055	UUu
DHRS7C	1.083	1.091	1.017	UUu
SDS	1.082	1.087	1.037	UUu
ZNF423	1.082	1.062	1.056	UUu
ALKBH7	1.082	1.091	1.070	UUu
RNF113B	1.082	1.123	1.039	UUu
TSPAN11	1.082	1.080	1.064	UUu
NO	1.081	1.100	1.057	UUu
SHROOM2	1.081	1.113	1.074	UUu
DTX1	1.081	1.113	1.047	UUu
RUNDC3A	1.081	1.098	1.020	UUu
RAB26	1.081	1.079	1.047	UUu
DACT1	1.081	1.101	1.079	UUu

DMRT3	1.081	1.125	1.013	UUu
AWAT1	1.081	1.102	1.071	UUu
UNC5B	1.081	1.084	1.037	UUu
CCDC87	1.081	1.090	1.077	UUu
FAM167A	1.081	1.105	1.053	UUu
PRRT3	1.081	1.064	1.059	UUu
TBX10	1.081	1.142	1.024	UUu
BEND4	1.081	1.077	1.067	UUu
SPINK5	1.080	1.375	1.025	UUu
ADGRD2	1.080	1.082	1.023	UUu
POU2F2	1.079	1.106	1.076	UUu
NEFH	1.078	1.124	1.027	UUu
CORO2B	1.078	1.127	1.019	UUu
KRT78	1.078	1.114	1.040	UUu
CCDC188	1.078	1.087	1.001	UUu
ASGR1	1.078	1.077	1.066	UUu
SRF	1.078	1.027	1.073	UUu
B3GNT3	1.078	1.101	1.062	UUu
INF2	1.078	1.117	1.022	UUu
SLC9A5	1.077	1.030	1.020	UUu
TLN1	1.077	1.061	1.057	UUu
NKD2	1.077	1.149	1.018	UUu
FDX1L	1.077	1.107	1.013	UUu
PPM1J	1.077	1.059	1.063	UUu
DNASE1L2	1.076	1.095	1.056	UUu
GUCY2D	1.076	1.100	1.042	UUu
SCGB1C1	1.076	1.106	1.042	UUu
QRFP	1.076	1.140	1.039	UUu
OR10V1	1.076	1.226	1.055	UUu
FGF4	1.075	1.084	1.072	UUu
VEGFC	1.075	1.093	1.017	UUu
NO	1.075	1.138	1.059	UUu
EMX2	1.074	1.087	1.061	UUu
LRP3	1.074	1.068	1.025	UUu
ANO8	1.074	1.061	1.073	UUu
PLEKHN1	1.074	1.048	1.046	UUu
DOT1L	1.074	1.113	1.033	UUu
NXPH4	1.073	1.110	1.032	UUu
COL5A1	1.073	1.084	1.015	UUu
OBSL1	1.073	1.058	1.060	UUu
OSBP2	1.073	1.094	1.050	UUu
FAM20C	1.073	1.103	1.017	UUu
IGSF9B	1.072	1.103	1.065	UUu
CEND1	1.072	1.126	1.045	UUu
FBLN7	1.072	1.074	1.058	UUu
TSGA10IP	1.072	1.109	1.022	UUu
TFDP3	1.071	1.122	1.016	UUu

SDK2	1.070	1.087	1.043	UUu
OGDHL	1.070	1.072	1.034	UUu
MMP17	1.070	1.149	1.011	UUu
ME3	1.070	1.068	1.064	UUu
ARHGAP22	1.070	1.087	1.015	UUu
ZNF575	1.070	1.089	1.051	UUu
SOX4	1.069	1.039	1.032	UUu
RHBDL1	1.069	1.032	1.052	UUu
NKAIN4	1.069	1.117	1.039	UUu
NKPD1	1.069	1.075	1.057	UUu
C20orf144	1.068	1.127	1.056	UUu
PLXNA1	1.067	1.044	1.044	UUu
PRTN3	1.067	1.085	1.019	UUu
PEMT	1.067	1.110	1.025	UUu
FOXI2	1.067	1.082	1.034	UUu
AK8	1.067	1.082	1.041	UUu
EPM2A	1.066	1.097	1.026	UUu
MAGED4B	1.066	1.086	1.029	UUu
CCDC8	1.066	1.080	1.050	UUu
DERL3	1.066	1.031	1.010	UUu
HMX1	1.065	1.085	1.061	UUu
ITPKA	1.065	1.123	1.020	UUu
LMNA	1.065	1.103	1.020	UUu
RGMA	1.065	1.074	1.033	UUu
RAP2C	1.065	1.054	1.001	UUu
KIF7	1.065	1.125	1.044	UUu
HAPLN4	1.065	1.100	1.030	UUu
PRRX2	1.064	1.155	1.057	UUu
SIRPG	1.064	1.197	1.063	UUu
ST6GALNAC1	1.064	1.090	1.025	UUu
TCEB3C	1.064	1.104	1.061	UUu
SOX3	1.064	1.094	1.053	UUu
NRXN2	1.064	1.071	1.058	UUu
ZNF541	1.064	1.088	1.052	UUu
ADSSL1	1.064	1.067	1.048	UUu
CFAP73	1.064	1.115	1.037	UUu
GFAP	1.063	1.106	1.042	UUu
L1CAM	1.063	1.104	1.052	UUu
SLC22A17	1.063	1.056	1.062	UUu
RND2	1.062	1.078	1.019	UUu
LAMC3	1.062	1.091	1.017	UUu
CDC42BPG	1.062	1.101	1.042	UUu
PLEKHA4	1.062	1.071	1.048	UUu
SCUBE1	1.062	1.067	1.012	UUu
TMEM200C	1.062	1.113	1.041	UUu
COL1A1	1.061	1.061	1.014	UUu
COMP	1.061	1.081	1.037	UUu

NEUROD2	1.061	1.120	1.048	UUu
CDX1	1.061	1.097	1.040	UUu
RAB11FIP3	1.061	1.112	1.022	UUu
GAS2L1	1.061	1.086	1.040	UUu
PITPNM3	1.061	1.092	1.027	UUu
RAB40A	1.061	1.084	1.035	UUu
ODF3L2	1.061	1.100	1.040	UUu
CPAMD8	1.060	1.067	1.035	UUu
RAX	1.060	1.131	1.006	UUu
KCNK15	1.060	1.101	1.029	UUu
NUDT14	1.060	1.123	1.036	UUu
SARS2	1.059	1.077	1.047	UUu
KLC3	1.058	1.119	1.050	UUu
PLA2G4D	1.058	1.085	1.054	UUu
HCN2	1.058	1.077	1.039	UUu
SALL3	1.058	1.082	1.057	UUu
DBNDD2	1.058	1.073	1.015	UUu
WNK2	1.058	1.077	1.033	UUu
FKRP	1.058	1.098	1.048	UUu
GPR150	1.058	1.088	1.041	UUu
KRT77	1.058	1.087	1.038	UUu
CACNA1A	1.057	1.056	1.015	UUu
KRT35	1.057	1.087	1.002	UUu
LTBP3	1.057	1.113	1.041	UUu
PCK1	1.057	1.110	1.053	UUu
SERPINF2	1.057	1.080	1.007	UUu
RAB3IL1	1.057	1.110	1.029	UUu
SMTN	1.057	1.093	1.013	UUu
SOX12	1.057	1.087	1.050	UUu
SYNM	1.057	1.099	1.042	UUu
MAMDC4	1.057	1.075	1.042	UUu
ADGRD1	1.057	1.047	1.019	UUu
FTCD	1.056	1.075	1.037	UUu
RTEL1	1.056	1.083	1.032	UUu
CAMSAP3	1.056	1.084	1.030	UUu
EMID1	1.056	1.103	1.055	UUu
ACAN	1.056	1.070	1.015	UUu
MUC6	1.056	1.064	1.035	UUu
RPL3L	1.056	1.082	1.011	UUu
MRC2	1.056	1.073	1.014	UUu
TCF15	1.055	1.139	1.045	UUu
KCTD19	1.055	1.070	1.039	UUu
RBFA	1.054	1.102	1.046	UUu
MGAT5B	1.054	1.077	1.047	UUu
GRIN2C	1.053	1.100	1.049	UUu
CYP46A1	1.053	1.084	1.020	UUu
TEX13B	1.053	1.110	1.018	UUu

SLC17A7	1.053	1.067	1.037	UUu
CARMIL3	1.053	1.083	1.012	UUu
CDH15	1.053	1.100	1.022	UUu
TCF3	1.053	1.093	1.040	UUu
KCNK7	1.053	1.094	1.006	UUu
TACC2	1.053	1.080	1.017	UUu
KCNH4	1.053	1.083	1.020	UUu
MYO15A	1.053	1.055	1.034	UUu
MMRN2	1.053	1.075	1.020	UUu
COL6A2	1.052	1.084	1.033	UUu
ANKRD24	1.052	1.091	1.038	UUu
YJEFN3	1.052	1.070	1.039	UUu
PPP1R1A	1.051	1.090	1.046	UUu
AIPL1	1.051	1.078	1.027	UUu
MYH14	1.051	1.084	1.021	UUu
PLPPR3	1.051	1.070	1.022	UUu
CCDC151	1.050	1.103	1.023	UUu
GRIK4	1.050	1.063	1.017	UUu
ADAMTS13	1.050	1.061	1.020	UUu
SLC2A4RG	1.050	1.073	1.037	UUu
AHNAK2	1.050	1.053	1.030	UUu
FAM183A	1.050	1.120	1.026	UUu
FOXB2	1.050	1.068	1.013	UUu
LAMA5	1.049	1.071	1.003	UUu
IGF2BP1	1.049	1.101	1.032	UUu
ROBO4	1.049	1.076	1.017	UUu
PHACTR3	1.049	1.091	1.043	UUu
CILP2	1.049	1.079	1.025	UUu
ZBTB7C	1.049	1.085	1.032	UUu
GTSE1	1.048	1.070	1.041	UUu
MYO7A	1.048	1.056	1.045	UUu
NOTCH3	1.048	1.073	1.046	UUu
ZBTB32	1.048	1.073	1.042	UUu
COL27A1	1.048	1.100	1.027	UUu
SCARF2	1.048	1.079	1.044	UUu
NOVA2	1.047	1.091	1.036	UUu
PEG3	1.047	1.069	1.040	UUu
LPIN3	1.047	1.065	1.018	UUu
TLN2	1.047	1.064	1.042	UUu
ZNF497	1.047	1.067	1.043	UUu
CEP170B	1.047	1.090	1.006	UUu
COL4A6	1.046	1.058	1.019	UUu
COL6A1	1.046	1.073	1.022	UUu
SARDH	1.046	1.083	1.040	UUu
KCNC3	1.046	1.089	1.010	UUu
CARNS1	1.046	1.092	1.012	UUu
MOB2	1.046	1.078	1.045	UUu

RP1L1	1.046	1.110	1.023	UUu
PDE4C	1.045	1.061	1.001	UUu
ZDHHC7	1.045	1.091	1.039	UUu
SRCIN1	1.045	1.091	1.040	UUu
KCNG4	1.045	1.096	1.032	UUu
LRRC45	1.045	1.078	1.042	UUu
EDN3	1.045	1.064	1.003	UUu
PAK3	1.045	1.078	1.020	UUu
FSTL3	1.045	1.098	1.042	UUu
ARHGAP23	1.045	1.057	1.005	UUu
RIMBP3	1.045	1.070	1.030	UUu
ADAMTS17	1.045	1.072	1.004	UUu
MNX1	1.044	1.081	1.035	UUu
NTM	1.044	1.062	1.040	UUu
ZNF580	1.044	1.093	1.032	UUu
H2AFY2	1.044	1.067	1.009	UUu
NPAS4	1.044	1.082	1.010	UUu
RASSF10	1.044	1.106	1.039	UUu
PHLDA1	1.043	1.053	1.006	UUu
VASH1	1.043	1.079	1.035	UUu
FSD1	1.043	1.095	1.040	UUu
ARHGEF17	1.042	1.065	1.020	UUu
DDN	1.042	1.091	1.039	UUu
CCDC33	1.042	1.070	1.009	UUu
PLIN1	1.042	1.094	1.040	UUu
FAM19A5	1.042	1.085	1.038	UUu
CHADL	1.042	1.081	1.006	UUu
CXorf49	1.042	1.116	1.037	UUu
AIRE	1.041	1.083	1.018	UUu
CACNA1F	1.041	1.064	1.032	UUu
CCDC107	1.041	1.062	1.038	UUu
MYH6	1.040	1.064	1.028	UUu
ANGPTL4	1.040	1.110	1.034	UUu
KLC2	1.040	1.064	1.007	UUu
OPN4	1.040	1.075	1.033	UUu
FAM181B	1.040	1.130	1.003	UUu
EYA2	1.040	1.083	1.025	UUu
KRT6A	1.040	1.090	1.006	UUu
CELSR1	1.040	1.057	1.032	UUu
ADAMTSL1	1.040	1.067	1.015	UUu
MEGF8	1.039	1.053	1.006	UUu
CFAP46	1.039	1.060	1.024	UUu
IGSF22	1.039	1.090	1.009	UUu
SPTBN4	1.038	1.087	1.035	UUu
SRRM4	1.038	1.058	1.022	UUu
MPG	1.037	1.087	1.036	UUu
ADIRF	1.037	1.101	1.032	UUu

CUX2	1.037	1.053	1.019	UUu
HUNK	1.037	1.076	1.020	UUu
RGAG1	1.037	1.076	1.006	UUu
MYO18B	1.037	1.069	1.015	UUu
GAS2L2	1.037	1.077	1.023	UUu
F7	1.037	1.087	1.027	UUu
ITGAD	1.037	1.119	1.033	UUu
DNAH17	1.037	1.056	1.021	UUu
CYGB	1.037	1.112	1.030	UUu
RYR1	1.036	1.050	1.005	UUu
GGACT	1.036	1.079	1.017	UUu
CHAT	1.035	1.070	1.027	UUu
FOLR1	1.035	1.086	1.034	UUu
RBBP8NL	1.035	1.106	1.023	UUu
RASGRF1	1.035	1.057	1.008	UUu
KNDC1	1.035	1.072	1.012	UUu
UTF1	1.034	1.087	1.022	UUu
CELF4	1.034	1.058	1.018	UUu
GPR135	1.034	1.074	1.013	UUu
ANKRD13A	1.034	1.080	1.032	UUu
AMOTL1	1.034	1.063	1.017	UUu
SHBG	1.033	1.072	1.023	UUu
LYNX1	1.033	1.074	1.032	UUu
PCP2	1.033	1.088	1.019	UUu
DNMT3B	1.032	1.084	1.001	UUu
PTK6	1.032	1.079	1.018	UUu
WNT10B	1.032	1.068	1.022	UUu
BRSK2	1.032	1.081	1.015	UUu
STRC	1.032	1.050	1.016	UUu
LDHD	1.032	1.078	1.011	UUu
MFAP4	1.032	1.076	1.011	UUu
BRSK1	1.032	1.078	1.017	UUu
MYH7	1.031	1.065	1.029	UUu
EXD3	1.031	1.055	1.015	UUu
IGDCC3	1.030	1.084	1.020	UUu
LARP6	1.030	1.081	1.003	UUu
SCUBE2	1.030	1.056	1.008	UUu
TPPP2	1.030	1.103	1.010	UUu
SETD1A	1.029	1.063	1.025	UUu
PPP1R1B	1.029	1.070	1.022	UUu
ARMCX4	1.028	1.081	1.010	UUu
SCN4A	1.027	1.067	1.017	UUu
KCNT1	1.027	1.066	1.025	UUu
ERBB3	1.027	1.057	1.006	UUu
NPEPL1	1.027	1.087	1.021	UUu
CTGF	1.025	1.071	1.023	UUu
POLRMT	1.025	1.052	1.017	UUu

TUSC1	1.025	1.089	1.003	UUu
POLD1	1.025	1.057	1.018	UUu
COL15A1	1.024	1.056	1.010	UUu
RABEP2	1.024	1.068	1.007	UUu
FAM170B	1.024	1.107	1.015	UUu
TENM4	1.023	1.054	1.011	UUu
LCN1	1.022	1.105	1.013	UUu
CSF3	1.021	1.082	1.020	UUu
GOLGA6A	1.013	1.058	1.008	UUu
TUB	-1.006	1.070	-1.039	DUD
SIRT4	-1.006	1.017	-1.116	DUD
SNX22	-1.009	1.067	-1.000	DUD
GCG	1.003	-1.104	1.009	UDU
HDLBP	1.001	-1.078	1.030	UDU
SNAI2	1.025	-1.008	1.225	UDU
XPNPEP2	1.078	-1.001	1.005	UDU
ENC1	1.071	-1.039	1.437	UDU
CCNI	1.055	-1.094	1.037	UDU
ZBTB7B	1.008	-1.073	1.030	UDU
EVA1B	1.011	-1.259	1.200	UDU
NUDT18	1.004	-1.104	1.069	UDU
SVBP	1.050	-1.006	1.843	UDU

Supplementary table 6. Functional annotation of genes from individual clusters/sub-clusters (showing p-values from pathway enrichment analysis).

Canonical Pathway	Sub-cluster DDd	Cluster DDU	Cluster DUU	Cluster UUD	Sub-cluster UUu
ATM Sigling	1.98E-04				
Mitotic Roles of Polo-Like Kise	7.05E-04				
PI3K/AKT Sigling	1.01E-03				
Heme Degradation	1.55E-03				
Mitochondrial Dysfunction	1.98E-03				
Role of CHK Proteins in Cell Cycle Checkpoint Control	2.31E-03				
2-ketoglutarate Dehydrogese Complex	2.55E-03				
Cell Cycle Regulation by BTG Family Proteins	2.96E-03				
Lactose Degradation III	3.78E-03				
Protein Ubiquitition Pathway	4.23E-03				
NRF2-mediated Oxidative Stress Response	4.44E-03				
Acetyl-CoA Biosynthesis I (Pyruvate Dehydrogese Complex)	5.24E-03				
Superpathway of Inositol Phosphate Compounds	5.28E-03				
3-phosphoinositide Biosynthesis	5.58E-03				
D-myo-inositol (1,4,5,6)-Tetrakisphosphate Biosynthesis	9.30E-03				
Telomerase Sigling	9.59E-03				
Cyclins and Cell Cycle Regulation	9.80E-03				
Dolichol and Dolichyl Phosphate Biosynthesis		5.61E-03			
Semaphorin Sigling in Neurons		9.76E-03			
Actin Nucleation by ARP-WASP Complex		1.08E-02			
Sirtuin Sigling Pathway			7.75E-03		
Sertoli Cell-Sertoli Cell Junction Sigling				2.64E-04	
GNRH Sigling				7.63E-04	
CDK5 Sigling				1.50E-03	
Germ Cell-Sertoli Cell Junction Sigling				1.73E-03	
Paxillin Sigling				2.44E-03	
Renin-Angiotensin Sigling				3.22E-03	
ERK/MAPK Sigling				3.23E-03	
PCP pathway				3.47E-03	
Activation of IRF by Cytosolic Pattern Recognition Receptors				3.80E-03	
Agrin Interactions at Neuromuscular Junction				4.91E-03	
STAT3 Pathway				5.97E-03	
BMP sigling pathway				6.43E-03	
CXCR4 Sigling				9.27E-03	
Hepatic Fibrosis / Hepatic Stellate Cell Activation					4.51E-06
GP6 Sigling Pathway					1.07E-04
Actin Cytoskeleton Sigling					1.95E-03
ILK Sigling					7.10E-03

Supplementary table 7. miRNAs and transcription factors (TF) with enriched (p-values) target genes in individual clusters/sub-clusters.

Regulators	DDd	DUU	UDD	UUD	UUu	Regulator type
miR-744					5.05E-04	miRNA
miR-744-5p					5.05E-04	miRNA
miR-1716					5.05E-04	miRNA
miR-212-5p					1.38E-03	miRNA
miR-7308-3p					1.38E-03	miRNA
miR-4319					2.49E-03	miRNA
miR-670					2.49E-03	miRNA
miR-351					3.05E-03	miRNA
miR-325-3p					3.98E-03	miRNA
miR-216			3.03E-03			miRNA
miR-329-3p			4.26E-03			miRNA
miR-144			7.85E-03			miRNA
miR-9-5p			8.10E-03			miRNA
miR-9a-5p			8.10E-03			miRNA
miR-144-3p			9.43E-03			miRNA
miR-381		8.00E-03				miRNA
miR-300		8.00E-03				miRNA
miR-133ab		9.28E-03				miRNA
miR-133bc-3p		9.28E-03				miRNA
miR-133b		9.28E-03				miRNA
GGGAGGRR_MAZ_Q6					5.06E-06	TF
NRSF_01					7.87E-05	TF
EGR2_01					1.72E-04	TF
CAGGTG_E12_Q6					3.87E-04	TF
E12_Q6					6.32E-04	TF
SF1_Q6					1.10E-03	TF
EGR_Q6					2.39E-03	TF
CP2_01					3.93E-03	TF
MYOGNF1_01					4.41E-03	TF
RREB1_01					4.79E-03	TF
ZF5_01					5.16E-03	TF
SREBP1_Q6					5.51E-03	TF
CACBINDINGPROTEIN_Q6					5.69E-03	TF
SP1_Q6_01					5.69E-03	TF
CAGCTG_AP4_Q5					7.44E-03	TF
ZIC3_01					7.99E-03	TF
AREB6_03					8.24E-03	TF
BACH1_01					9.26E-03	TF
POU3F2_02					9.53E-03	TF
SREBP_Q3					9.81E-03	TF
OCT1_01			1.99E-03			TF
OCT1_B			2.10E-03			TF
SP1_01		9.86E-03				TF

LFA1_Q6				4.41E-03		TF
TTF1_Q6				5.06E-03		TF
MAF_Q6				5.68E-03		TF
SCGGAAGY_ELK1_02	5.10E-11					TF
SREBP1_01	1.23E-05					TF
MGGAAGTG_GABP_B	4.10E-05					TF
TCANNTGAY_SREBP1_01	5.12E-04					TF
RCGCANGCGY_NRF1_Q6	8.22E-04					TF
ARNT_02	1.72E-03					TF
GTGACGY_E4F1_Q6	2.57E-03					TF
ELK1_02	4.02E-03					TF
GCCATNTTG_YY1_Q6	4.06E-03					TF
USF_01	5.18E-03					TF
NFY_Q6_01	6.21E-03					TF
NRF2_01	8.27E-03					TF
ELF1_Q6	9.86E-03					TF

Supplementary table 8. Summary of gene, miRNA, TF nodes, and edges in indvidual networks.

Cluster/sub-cluster	# gene node	# TF node	# edge (TF-gene)	# miRNA node	# edge (miRNA-gene)	# edge (gene-gene)
UUu	220	19	394	9	233	29
UDD	10	2	6	6	27	2
DUU	6	1	2	5	17	1
UUD	12	3	17	0	0	1
DDd	135	11	260	0	0	20

Supplementary table 9. Paired nodes and edge type for indvidual networks from clusters/sub-clusters.

Node1	edge (PT: protein-TF; PP: protein-protein; PM: protein-miRNA)	Node2	cluster/sub-cluster
RAB1B	PT	NRF1_Q6	DDd
RNF5	PT	ELF1_Q6	DDd
RNF115	PT	ARNT_02	DDd
DNAJB4	PT	E4F1_Q6	DDd
PSME3	PT	ARNT_02	DDd
PSME3	PT	E4F1_Q6	DDd
ANAPC13	PT	ARNT_02	DDd
TCTA	PT	NFY_Q6_01	DDd
PIP5K1A	PT	SREBP1_01	DDd
ATP5D	PT	NRF1_Q6	DDd
EED	PT	NRF1_Q6	DDd
BAX	PT	GABP_B	DDd
STARD3	PT	SREBP1_01	DDd
EMC4	PT	ELK1_02	DDd
GPANK1	PT	ELK1_02	DDd
FAM63B	PT	SREBP1_01	DDd
HM13	PT	ELF1_Q6	DDd
RNF4	PT	GABP_B	DDd
NDUFS1	PT	USF_01	DDd
BAX	PT	USF_01	DDd
CUTC	PT	ELK1_02	DDd
SOCS4	PT	GABP_B	DDd
WDR55	PT	ELK1_02	DDd
TRMT112	PT	ELK1_02	DDd
RAE1	PT	GABP_B	DDd
VRK3	PT	ELK1_02	DDd
RAB1B	PT	GABP_B	DDd
KLHDC3	PT	ELK1_02	DDd
TTC9C	PT	NFY_Q6_01	DDd
RBM5	PT	YY1_Q6	DDd
NOL12	PT	ELK1_02	DDd
PIP5K1A	PT	SREBP1_01	DDd
CNPPD1	PT	NRF1_Q6	DDd
U2AF2	PT	GABP_B	DDd
TRAPPC4	PT	ELK1_02	DDd
IL18BP	PT	GABP_B	DDd
TADA3	PT	GABP_B	DDd
NDUFS1	PT	YY1_Q6	DDd
MAGED2	PT	GABP_B	DDd

CNOT10	PT	GABP_B	DDd
XPR1	PT	ELF1_Q6	DDd
U2AF1L4	PT	GABP_B	DDd
GOLGA1	PT	NRF1_Q6	DDd
IKBKB	PT	GABP_B	DDd
U2AF2	PT	YY1_Q6	DDd
FAM134A	PT	ELF1_Q6	DDd
RRAGC	PT	SREBP1_01	DDd
RNF5	PT	NFY_Q6_01	DDd
MAP2K2	PT	NRF1_Q6	DDd
COX8A	PT	ELK1_02	DDd
FANCD2	PT	ELK1_02	DDd
RTCA	PT	NRF1_Q6	DDd
FAM134A	PT	SREBP1_01	DDd
TP53BP1	PT	SREBP1_01	DDd
RACGAP1	PT	YY1_Q6	DDd
U2AF2	PT	SREBP1_01	DDd
KLHDC3	PT	ELK1_02	DDd
PREB	PT	NRF1_Q6	DDd
CDK2AP2	PT	E4F1_Q6	DDd
METTL6	PT	ELK1_02	DDd
SERTAD1	PT	ELK1_02	DDd
LAMTOR1	PT	SREBP1_01	DDd
WDR55	PT	GABP_B	DDd
TADA3	PT	ELK1_02	DDd
NEDD8	PT	GABP_B	DDd
CNPPD1	PT	SREBP1_01	DDd
RACGAP1	PT	NFY_Q6_01	DDd
U2AF2	PT	SREBP1_01	DDd
MAGED2	PT	ELK1_02	DDd
ZSCAN29	PT	NRF1_Q6	DDd
FAM134A	PT	NRF2_01	DDd
CUTC	PT	GABP_B	DDd
BLOC1S1	PT	ARNT_02	DDd
ZSCAN29	PT	ELK1_02	DDd
STARD3	PT	ELK1_02	DDd
BCAS3	PT	GABP_B	DDd
SCFD1	PT	ELK1_02	DDd
RRAGC	PT	SREBP1_01	DDd
GTF3C2	PT	ELK1_02	DDd
TCEANC2	PT	ELK1_02	DDd
PSME3	PT	ELK1_02	DDd
BLOC1S1	PT	SREBP1_01	DDd
FAM134A	PT	E4F1_Q6	DDd
ZNF436	PT	NFY_Q6_01	DDd
DHTKD1	PT	NFY_Q6_01	DDd
CDK2	PT	SREBP1_01	DDd

MLF2	PT	GABP_B	DDd
PROSC	PT	E4F1_Q6	DDd
SLC36A1	PT	USF_01	DDd
HMOX2	PT	E4F1_Q6	DDd
PCYT1A	PT	ELK1_02	DDd
NDUFS1	PT	ELK1_02	DDd
ERCC6	PT	NRF1_Q6	DDd
TSSC1	PT	GABP_B	DDd
DAZAP2	PT	SREBP1_01	DDd
DAZAP2	PT	SREBP1_01	DDd
OS9	PT	NFY_Q6_01	DDd
TRMT112	PT	NRF2_01	DDd
MLF2	PT	E4F1_Q6	DDd
COX8A	PT	YY1_Q6	DDd
CTR9	PT	GABP_B	DDd
COX8A	PT	ELK1_02	DDd
ANAPC5	PT	NFY_Q6_01	DDd
RAD9A	PT	USF_01	DDd
DDOST	PT	ELK1_02	DDd
IKBKB	PT	ELF1_Q6	DDd
PCIF1	PT	E4F1_Q6	DDd
SLC25A3	PT	E4F1_Q6	DDd
VPS53	PT	GABP_B	DDd
PROSC	PT	YY1_Q6	DDd
TTC14	PT	ELK1_02	DDd
COPS7A	PT	ELK1_02	DDd
CLN3	PT	SREBP1_01	DDd
FUCA1	PT	GABP_B	DDd
ORAI3	PT	E4F1_Q6	DDd
CHCHD4	PT	NRF1_Q6	DDd
USP21	PT	ELK1_02	DDd
RALA	PT	ELF1_Q6	DDd
VPS53	PT	NRF2_01	DDd
TBCC	PT	ELK1_02	DDd
ERCC6	PT	ELK1_02	DDd
ANAPC4	PT	ELK1_02	DDd
MIEN1	PT	E4F1_Q6	DDd
CLN3	PT	USF_01	DDd
CUTC	PT	NRF2_01	DDd
ACBD5	PT	ELK1_02	DDd
NIT1	PT	USF_01	DDd
MUT	PT	ELK1_02	DDd
KRTCAP2	PT	ELK1_02	DDd
U2AF1L4	PT	SREBP1_01	DDd
SLC36A1	PT	SREBP1_01	DDd
PCYT1A	PT	ELK1_02	DDd
ZNF768	PT	GABP_B	DDd

NFS1	PT	ELK1_02	DDd
RAD9A	PT	SREBP1_01	DDd
VPS53	PT	ELK1_02	DDd
KRTCAP2	PT	ELK1_02	DDd
ANAPC4	PT	YY1_Q6	DDd
CTR9	PT	ELK1_02	DDd
BLOC1S1	PT	SREBP1_01	DDd
TEFM	PT	ELK1_02	DDd
CTNS	PT	SREBP1_01	DDd
U2AF1L4	PT	E4F1_Q6	DDd
U2AF1L4	PT	NRF1_Q6	DDd
HIST1H2AC	PT	NFY_Q6_01	DDd
FBXO8	PT	GABP_B	DDd
CLN3	PT	ELK1_02	DDd
ANAPC13	PT	SREBP1_01	DDd
RAB1B	PT	NRF2_01	DDd
CBFB	PT	GABP_B	DDd
OGDH	PT	E4F1_Q6	DDd
GTF3C2	PT	NRF1_Q6	DDd
TBCC	PT	ELK1_02	DDd
DNAJC14	PT	ELK1_02	DDd
TBCC	PT	NRF2_01	DDd
DDOST	PT	ELK1_02	DDd
XCL2	PT	GABP_B	DDd
RUFY1	PT	YY1_Q6	DDd
RUFY1	PT	ELF1_Q6	DDd
MAGED2	PT	NRF1_Q6	DDd
HCCS	PT	ELK1_02	DDd
U2AF2	PT	ARNT_02	DDd
U2AF2	PT	NRF1_Q6	DDd
CNPY3	PT	ELK1_02	DDd
TP53BP1	PT	ELK1_02	DDd
TCTA	PT	ELK1_02	DDd
TRADD	PT	ELK1_02	DDd
FAM134A	PT	ARNT_02	DDd
BAX	PT	SREBP1_01	DDd
SLC25A3	PT	NRF1_Q6	DDd
CIRBP	PT	NRF1_Q6	DDd
KCNMB3	PT	ELF1_Q6	DDd
CNPPD1	PT	ARNT_02	DDd
DGUOK	PT	GABP_B	DDd
MTMR9	PT	ELK1_02	DDd
PAFAH2	PT	GABP_B	DDd
RNF115	PT	USF_01	DDd
CNPPD1	PT	NRF2_01	DDd
IKBKB	PT	E4F1_Q6	DDd
SLC36A1	PT	SREBP1_01	DDd

KDELR2	PT	E4F1_Q6	DDd
SOCS4	PT	NRF1_Q6	DDd
GPANK1	PT	NRF1_Q6	DDd
RNF115	PT	SREBP1_01	DDd
CNPPD1	PT	USF_01	DDd
AAMP	PT	E4F1_Q6	DDd
NIT1	PT	E4F1_Q6	DDd
HM13	PT	GABP_B	DDd
RAD9A	PT	SREBP1_01	DDd
RNF5	PT	YY1_Q6	DDd
RALA	PT	YY1_Q6	DDd
CNPPD1	PT	SREBP1_01	DDd
TMED4	PT	NRF1_Q6	DDd
TCTA	PT	NRF1_Q6	DDd
SLC36A1	PT	ARNT_02	DDd
FAM63B	PT	ELK1_02	DDd
BAX	PT	SREBP1_01	DDd
F11R	PT	ELK1_02	DDd
ZMYM6	PT	NRF1_Q6	DDd
U2AF2	PT	USF_01	DDd
SPOP	PT	NRF1_Q6	DDd
CLN3	PT	SREBP1_01	DDd
FAM134A	PT	USF_01	DDd
PPM1D	PT	NRF1_Q6	DDd
U2AF2	PT	ELK1_02	DDd
ZNF692	PT	NRF1_Q6	DDd
ZSCAN29	PT	ELK1_02	DDd
TRMT112	PT	GABP_B	DDd
TNFSF13	PT	ELF1_Q6	DDd
SRPRA	PT	E4F1_Q6	DDd
MTF2	PT	YY1_Q6	DDd
FBXO8	PT	ELK1_02	DDd
NOL12	PT	GABP_B	DDd
NDUFS1	PT	ELK1_02	DDd
GTPBP10	PT	NRF1_Q6	DDd
STAMBP	PT	E4F1_Q6	DDd
CNOT10	PT	ELK1_02	DDd
GPANK1	PT	NRF2_01	DDd
CNPPD1	PT	ELF1_Q6	DDd
TBCC	PT	GABP_B	DDd
FAM134A	PT	NRF1_Q6	DDd
SLC25A13	PT	NFY_Q6_01	DDd
SLC35D1	PT	NRF1_Q6	DDd
GTF3C2	PT	ELK1_02	DDd
TP53BP1	PT	NFY_Q6_01	DDd
RRAGC	PT	ARNT_02	DDd
DDOST	PT	NRF2_01	DDd

RAD9A	PT	ARNT_02	DDd
BAX	PT	ARNT_02	DDd
HM13	PT	E4F1_Q6	DDd
FAM134A	PT	SREBP1_01	DDd
TP53BP1	PT	YY1_Q6	DDd
U2AF1L4	PT	NRF2_01	DDd
CXXC1	PT	ELK1_02	DDd
GOLGA1	PT	ELK1_02	DDd
NFYC	PT	YY1_Q6	DDd
MIEN1	PT	ELK1_02	DDd
DDOST	PT	GABP_B	DDd
CLN3	PT	ARNT_02	DDd
NEDD8	PT	ELK1_02	DDd
TSTA3	PT	ELK1_02	DDd
FBXO8	PT	E4F1_Q6	DDd
ACTR8	PT	YY1_Q6	DDd
CBFB	PT	NRF1_Q6	DDd
ATP8B4	PT	ELK1_02	DDd
SASH3	PT	USF_01	DDd
TSSC1	PT	ELF1_Q6	DDd
RNF5	PT	E4F1_Q6	DDd
DGUOK	PT	ELK1_02	DDd
PCIF1	PT	YY1_Q6	DDd
RNF115	PT	SREBP1_01	DDd
SSU72	PT	ELK1_02	DDd
TSTA3	PT	ELK1_02	DDd
DAZAP2	PT	NRF2_01	DDd
SLC36A1	PT	ELK1_02	DDd
CNPPD1	PT	E4F1_Q6	DDd
CDC37	PT	ELK1_02	DDd
PCIF1	PT	NRF1_Q6	DDd
SRPRA	PT	ELK1_02	DDd
FANCD2	PT	ELK1_02	DDd
MTMR9	PP	MTMR9	DDd
TRADD	PP	TRADD	DDd
GOLGA1	PP	GOLGA1	DDd
RACGAP1	PP	RACGAP1	DDd
CDK2	PP	CDC37	DDd
DGUOK	PP	DGUOK	DDd
CUTC	PP	CUTC	DDd
EED	PP	EED	DDd
PSME3	PP	PSME3	DDd
MUT	PP	MUT	DDd
BLOC1S1	PP	BLOC1S1	DDd
MAP2K2	PP	MAP2K2	DDd
F11R	PP	F11R	DDd
IKBKB	PP	IKBKB	DDd

NEDD8	PP	NEDD8	DDd
CDK2	PP	CDK2	DDd
CBFB	PP	CBFB	DDd
DAZAP2	PP	DAZAP2	DDd
RRAGC	PP	RRAGC	DDd
IKBKB	PP	CDC37	DDd
CCDC30	PM	miR-133b	DUU
TRAM2	PM	miR-133b	DUU
USP6	PM	miR-133b	DUU
CAMTA1	PM	miR-300	DUU
CCDC30	PM	miR-300	DUU
KLF16	PM	miR-300	DUU
USP6	PM	miR-300	DUU
KLF16	PT	SP1_01	DUU
GABARAP	PT	SP1_01	DUU
TRAM2	PP	TRAM2	DUU
CADM1	PP	CADM1	UDD
SYAP1	PM	miR-144	UDD
JDP2	PM	miR-144	UDD
SKIL	PM	miR-144	UDD
FKBP7	PM	miR-144	UDD
CADM1	PM	miR-144	UDD
BTNL8	PM	miR-216	UDD
TMTC1	PM	miR-216	UDD
SKIL	PM	miR-216	UDD
CADM1	PM	miR-216	UDD
CADM1	PM	miR-329-3p	UDD
JDP2	PM	miR-329-3p	UDD
TOR3A	PM	miR-329-3p	UDD
SYAP1	PM	miR-9-5p	UDD
FKBP7	PM	miR-9-5p	UDD
GBA	PM	miR-9-5p	UDD
TMTC1	PM	miR-9-5p	UDD
JDP2	PM	miR-9-5p	UDD
SPTY2D1	PT	OCT1_01	UDD
CADM1	PT	OCT1_01	UDD
SKIL	PT	OCT1_01	UDD
CADM1	PT	OCT1_B	UDD
SPTY2D1	PT	OCT1_B	UDD
SKIL	PT	OCT1_B	UDD
SKIL	PP	SKIL	UDD
TPM2	PP	TPM2	UUD
NKX2-3	PT	LFA1_Q6	UUD
WNT3	PT	MAF_Q6	UUD
FXYD4	PT	MAF_Q6	UUD
NKX2-1	PT	MAF_Q6	UUD
LMOD3	PT	LFA1_Q6	UUD

LZTS2	PT	TTF1_Q6	UUD
FBXL15	PT	LFA1_Q6	UUD
LZTS2	PT	MAF_Q6	UUD
JPH4	PT	LFA1_Q6	UUD
EXOC3L1	PT	MAF_Q6	UUD
NKX2-1	PT	LFA1_Q6	UUD
STK32C	PT	TTF1_Q6	UUD
LZTS2	PT	LFA1_Q6	UUD
WNT3	PT	TTF1_Q6	UUD
TPM2	PT	TTF1_Q6	UUD
NKX2-1	PT	TTF1_Q6	UUD
MYOG	PT	LFA1_Q6	UUD
ZDHHC7	PM	miR-351	UUu
TLN1	PM	miR-325-3p	UUu
KCNH4	PM	miR-351	UUu
NEUROD2	PM	miR-325-3p	UUu
ANGPTL4	PM	miR-325-3p	UUu
EMID1	PM	miR-351	UUu
FBLN7	PM	miR-212-5p	UUu
FAM167A	PM	miR-670	UUu
FBLN7	PM	miR-7308-3p	UUu
MEGF8	PM	miR-4319	UUu
B4GALT1	PM	miR-4319	UUu
FADS3	PM	miR-744	UUu
INF2	PM	miR-325-3p	UUu
CORO2B	PM	miR-670	UUu
SRRM4	PM	miR-212-5p	UUu
MEGF8	PM	miR-670	UUu
RND2	PM	miR-351	UUu
SRCIN1	PM	miR-670	UUu
CCDC151	PM	miR-325-3p	UUu
LRP3	PM	miR-1716	UUu
RAB43	PM	miR-7308-3p	UUu
TANC2	PM	miR-7308-3p	UUu
SLC17A7	PM	miR-325-3p	UUu
SARS2	PM	miR-325-3p	UUu
GAS2L1	PM	miR-670	UUu
RASGRF1	PM	miR-325-3p	UUu
SLC22A17	PM	miR-325-3p	UUu
SPINK5	PM	miR-325-3p	UUu
RASSF10	PM	miR-351	UUu
NPAS4	PM	miR-1716	UUu
ENTPD3	PM	miR-670	UUu
GAS2L1	PM	miR-4319	UUu
KCNC3	PM	miR-4319	UUu
OSBP2	PM	miR-7308-3p	UUu
AIPL1	PM	miR-325-3p	UUu

UTF1	PM	miR-325-3p	UUu
MYO7A	PM	miR-670	UUu
CDC42BPG	PM	miR-4319	UUu
RASSF3	PM	miR-4319	UUu
PHC2	PM	miR-4319	UUu
TANC2	PM	miR-212-5p	UUu
RND2	PM	miR-4319	UUu
FAM167A	PM	miR-4319	UUu
BRSK2	PM	miR-351	UUu
IGF2BP1	PM	miR-670	UUu
KLC3	PM	miR-351	UUu
RAX	PM	miR-7308-3p	UUu
RASSF3	PM	miR-351	UUu
ZDHHC7	PM	miR-325-3p	UUu
NPAS4	PM	miR-744-5p	UUu
LRP3	PM	miR-744	UUu
SOX12	PM	miR-4319	UUu
LRP3	PM	miR-325-3p	UUu
ERBB3	PM	miR-351	UUu
B3GNT3	PM	miR-4319	UUu
FADS3	PM	miR-744-5p	UUu
BEND4	PM	miR-7308-3p	UUu
PLIN4	PM	miR-325-3p	UUu
PLPPR3	PM	miR-325-3p	UUu
NKPD1	PM	miR-325-3p	UUu
SLC17A7	PM	miR-351	UUu
RASGRF1	PM	miR-351	UUu
NKPD1	PM	miR-670	UUu
CDC42BPG	PM	miR-351	UUu
FADS3	PM	miR-7308-3p	UUu
PHC2	PM	miR-351	UUu
PRRX2	PM	miR-7308-3p	UUu
SETBP1	PM	miR-212-5p	UUu
IGF2BP1	PM	miR-4319	UUu
RABEP2	PM	miR-351	UUu
NFIC	PM	miR-4319	UUu
SRCIN1	PM	miR-351	UUu
ZNF423	PM	miR-325-3p	UUu
SCARF2	PM	miR-4319	UUu
KCNH4	PM	miR-4319	UUu
BRSK2	PM	miR-670	UUu
FAM20C	PM	miR-325-3p	UUu
RASSF10	PM	miR-670	UUu
B4GALT1	PM	miR-351	UUu
LDHD	PM	miR-7308-3p	UUu
OSBP2	PM	miR-212-5p	UUu
EMID1	PM	miR-325-3p	UUu

FADS3	PM	miR-1716	UUu
IGF2BP1	PM	miR-351	UUu
LDHD	PM	miR-212-5p	UUu
IGF2BP1	PM	miR-7308-3p	UUu
KCNH4	PM	miR-670	UUu
NSUN4	PM	miR-325-3p	UUu
COL5A1	PM	miR-325-3p	UUu
ASNA1	PM	miR-325-3p	UUu
RP1L1	PM	miR-212-5p	UUu
FDX1L	PM	miR-325-3p	UUu
CAMSAP3	PM	miR-351	UUu
AIRE	PM	miR-7308-3p	UUu
TACC2	PM	miR-351	UUu
SRF	PM	miR-670	UUu
B3GNT3	PM	miR-670	UUu
RASGRF1	PM	miR-670	UUu
EMID1	PM	miR-4319	UUu
HSPA12B	PM	miR-325-3p	UUu
EMID1	PM	miR-670	UUu
ANO8	PM	miR-325-3p	UUu
STRN4	PM	miR-325-3p	UUu
VPS37B	PM	miR-351	UUu
CDC42BPG	PM	miR-325-3p	UUu
WNK2	PM	miR-212-5p	UUu
ERBB3	PM	miR-4319	UUu
SOX12	PM	miR-670	UUu
RND2	PM	miR-670	UUu
PLXNA1	PM	miR-670	UUu
WNK2	PM	miR-7308-3p	UUu
BRSK2	PM	miR-4319	UUu
SOX12	PM	miR-1716	UUu
GPR173	PM	miR-670	UUu
PAK3	PM	miR-325-3p	UUu
PLA2G4D	PM	miR-670	UUu
HUNK	PM	miR-7308-3p	UUu
B3GNT3	PM	miR-351	UUu
KRT35	PM	miR-325-3p	UUu
NKPD1	PM	miR-4319	UUu
SPTBN4	PM	miR-1716	UUu
RABEP2	PM	miR-670	UUu
BEND4	PM	miR-212-5p	UUu
GNAZ	PM	miR-212-5p	UUu
CYGB	PM	miR-212-5p	UUu
SRCIN1	PM	miR-4319	UUu
MEGF8	PM	miR-351	UUu
PHC2	PM	miR-670	UUu
KLC3	PM	miR-670	UUu

CORO2B	PM	miR-351	UUu
B4GALT1	PM	miR-670	UUu
CYP46A1	PM	miR-325-3p	UUu
SCARF2	PM	miR-351	UUu
EMX2	PM	miR-325-3p	UUu
RAB43	PM	miR-212-5p	UUu
SCGB1C1	PM	miR-325-3p	UUu
IGF2BP1	PM	miR-212-5p	UUu
MFAP4	PM	miR-325-3p	UUu
SETBP1	PM	miR-7308-3p	UUu
RABEP2	PM	miR-325-3p	UUu
LRP3	PM	miR-744-5p	UUu
SLC17A7	PM	miR-670	UUu
CAMSAP3	PM	miR-4319	UUu
MYO7A	PM	miR-351	UUu
SOX12	PM	miR-744	UUu
SLC17A7	PM	miR-4319	UUu
FAM167A	PM	miR-351	UUu
POU2F2	PM	miR-670	UUu
TACC2	PM	miR-4319	UUu
RIPK4	PM	miR-325-3p	UUu
BRSK2	PM	miR-325-3p	UUu
LTBP3	PM	miR-325-3p	UUu
ARMCX4	PM	miR-325-3p	UUu
FDX1L	PM	miR-744	UUu
RASSF3	PM	miR-7308-3p	UUu
VPS37B	PM	miR-4319	UUu
POU2F2	PM	miR-351	UUu
RASGRF1	PM	miR-4319	UUu
CDC42BPG	PM	miR-670	UUu
SPTBN4	PM	miR-744	UUu
NPAS4	PM	miR-744	UUu
FADS3	PM	miR-212-5p	UUu
SRF	PM	miR-325-3p	UUu
CELSR1	PM	miR-325-3p	UUu
TANC2	PM	miR-670	UUu
PLXNA1	PM	miR-4319	UUu
PEMT	PM	miR-325-3p	UUu
PHACTR3	PM	miR-4319	UUu
RASSF10	PM	miR-4319	UUu
NKPD1	PM	miR-351	UUu
KRT78	PM	miR-325-3p	UUu
GRIK4	PM	miR-325-3p	UUu
MYO7A	PM	miR-4319	UUu
HAPLN4	PM	miR-325-3p	UUu
NUDT14	PM	miR-325-3p	UUu
SOX12	PM	miR-744-5p	UUu

PEG3	PM	miR-325-3p	UUu
AK8	PM	miR-325-3p	UUu
TACC2	PM	miR-670	UUu
SCARF2	PM	miR-670	UUu
AIRE	PM	miR-212-5p	UUu
NFIC	PM	miR-670	UUu
KLC3	PM	miR-4319	UUu
BEND4	PM	miR-325-3p	UUu
CAMSAP3	PM	miR-670	UUu
CORO2B	PM	miR-4319	UUu
PRRX2	PM	miR-212-5p	UUu
GAS2L1	PM	miR-351	UUu
C20orf144	PM	miR-325-3p	UUu
GPR173	PM	miR-351	UUu
KCNC3	PM	miR-670	UUu
SRF	PM	miR-351	UUu
SRF	PM	miR-4319	UUu
RBMS1	PM	miR-325-3p	UUu
SOX3	PM	miR-325-3p	UUu
ARHGEF17	PM	miR-325-3p	UUu
ENTPD3	PM	miR-351	UUu
PLA2G4D	PM	miR-4319	UUu
AHNAK2	PM	miR-325-3p	UUu
SPTBN4	PM	miR-744-5p	UUu
RASSF3	PM	miR-212-5p	UUu
PHACTR3	PM	miR-670	UUu
GNAZ	PM	miR-7308-3p	UUu
HCN2	PM	miR-325-3p	UUu
HEBP2	PM	miR-325-3p	UUu
PRTN3	PM	miR-325-3p	UUu
SRRM4	PM	miR-7308-3p	UUu
HUNK	PM	miR-212-5p	UUu
FDX1L	PM	miR-744-5p	UUu
RAX	PM	miR-212-5p	UUu
FDX1L	PM	miR-1716	UUu
ENTPD3	PM	miR-4319	UUu
DNASE1L2	PM	miR-325-3p	UUu
RP1L1	PM	miR-7308-3p	UUu
NFIC	PM	miR-325-3p	UUu
HUNK	PM	miR-325-3p	UUu
RABEP2	PM	miR-4319	UUu
CACNA1F	PM	miR-325-3p	UUu
CYGB	PM	miR-7308-3p	UUu
RPP25	PM	miR-325-3p	UUu
PLXNA1	PM	miR-351	UUu
PLA2G4D	PM	miR-351	UUu
ZDHHC7	PM	miR-670	UUu

TANC2	PM	miR-4319	UUu
ZDHHC7	PM	miR-4319	UUu
KCNK15	PM	miR-325-3p	UUu
VPS37B	PM	miR-670	UUu
ERBB3	PM	miR-670	UUu
RASSF3	PM	miR-670	UUu
GPR173	PM	miR-4319	UUu
POU2F2	PM	miR-4319	UUu
ZNF580	PM	miR-325-3p	UUu
DDN	PM	miR-325-3p	UUu
CDC42BPG	PP	CDC42BPG	UUu
SCARF2	PP	SCARF2	UUu
B4GALT1	PP	B4GALT1	UUu
DNMT3B	PP	SUMO1	UUu
POU2F2	PP	POU2F2	UUu
SCUBE1	PP	SCUBE2	UUu
LMNA	PP	LMNA	UUu
RYR1	PP	RYR1	UUu
SCUBE2	PP	SCUBE2	UUu
PLXNA1	PP	PLXNA1	UUu
AIRE	PP	AIRE	UUu
ARHGEF17	PP	ARHGEF17	UUu
PAK3	PP	PAK3	UUu
EXT1	PP	EXT1	UUu
SRF	PP	SRF	UUu
ERBB3	PP	ERBB3	UUu
ROBO4	PP	ROBO4	UUu
SUMO1	PP	DNMT3B	UUu
PHC2	PP	PHC2	UUu
GNAZ	PP	EYA2	UUu
EMID1	PP	EMID1	UUu
SCUBE2	PP	SCUBE1	UUu
GNAZ	PP	GNAZ	UUu
PPM1J	PP	SUMO1	UUu
SCUBE1	PP	SCUBE1	UUu
SERPINF2	PP	SERPINF2	UUu
SUMO1	PP	PPM1J	UUu
DTX1	PP	DTX1	UUu
ASNA1	PP	ASNA1	UUu
ARMCX4	PT	MAZ_Q6	UUu
HEBP2	PT	CACBINDINGPROTEIN_Q6	UUu
EMX2	PT	RREB1_01	UUu
RYR1	PT	MAZ_Q6	UUu
CORO2B	PT	AP4_Q5	UUu
PENK	PT	MAZ_Q6	UUu
SMTN	PT	E12_Q6	UUu

RBMS1	PT	SF1_Q6	UUu
ROBO4	PT	AP4_Q5	UUu
SRCIN1	PT	CP2_01	UUu
SCUBE2	PT	AREB6_03	UUu
IGF2BP1	PT	ZF5_01	UUu
DRAP1	PT	SF1_Q6	UUu
ZNF575	PT	MAZ_Q6	UUu
SOX12	PT	E12_Q6	UUu
OSBP2	PT	EGR_Q6	UUu
MRC2	PT	EGR2_01	UUu
ERBB3	PT	ZIC3_01	UUu
NKPD1	PT	CP2_01	UUu
SRCIN1	PT	SREBP1_Q6	UUu
GPR173	PT	MAZ_Q6	UUu
WNT10B	PT	E12_Q6	UUu
IGF2BP1	PT	E12_Q6	UUu
IGF2BP1	PT	EGR_Q6	UUu
PLIN1	PT	MAZ_Q6	UUu
CORO2B	PT	E12_Q6	UUu
COL27A1	PT	MAZ_Q6	UUu
DNASE1L2	PT	EGR2_01	UUu
CACNA1A	PT	SREBP_Q3	UUu
MYO18B	PT	AP4_Q5	UUu
PLEKHA4	PT	AP4_Q5	UUu
WNK2	PT	E12_Q6	UUu
RBMS1	PT	MAZ_Q6	UUu
CSF3	PT	BACH1_01	UUu
SOX12	PT	SREBP1_Q6	UUu
GUCY2D	PT	MAZ_Q6	UUu
ERBB3	PT	E12_Q6	UUu
FSTL3	PT	ZIC3_01	UUu
FOXI1	PT	E12_Q6	UUu
NPAS4	PT	CACBINDINGPROTEIN_Q6	UUu
GPR173	PT	E12_Q6	UUu
BRSK2	PT	ZF5_01	UUu
BRSK2	PT	MAZ_Q6	UUu
ERBB3	PT	AP4_Q5	UUu
CELF4	PT	RREB1_01	UUu
GNAZ	PT	RREB1_01	UUu
SPINK5	PT	AP4_Q5	UUu
DTX1	PT	SREBP_Q3	UUu
RAB43	PT	RREB1_01	UUu
NOVA2	PT	MAZ_Q6	UUu
DRAP1	PT	ZF5_01	UUu
CELF4	PT	EGR2_01	UUu
SNPH	PT	MAZ_Q6	UUu

DTX1	PT	SREBP1_Q6	UUu
ARHGEF17	PT	CACBINDINGPROTEIN_Q6	UUu
VASH1	PT	EGR2_01	UUu
CEND1	PT	SREBP_Q3	UUu
CDH15	PT	E12_Q6	UUu
FAM181B	PT	E12_Q6	UUu
WNT10B	PT	AP4_Q5	UUu
NXPH4	PT	AP4_Q5	UUu
SRCIN1	PT	EGR2_01	UUu
NXPH4	PT	ZF5_01	UUu
LRP10	PT	E12_Q6	UUu
SLC9A5	PT	EGR_Q6	UUu
CACNA1A	PT	ZF5_01	UUu
RAB26	PT	E12_Q6	UUu
KIF7	PT	ZIC3_01	UUu
ARHGAP24	PT	MAZ_Q6	UUu
ZNF423	PT	POU3F2_02	UUu
RAB43	PT	E12_Q6	UUu
RGAG1	PT	BACH1_01	UUu
GPR173	PT	RREB1_01	UUu
OGDHL	PT	POU3F2_02	UUu
SMTN	PT	AREB6_03	UUu
SLC22A17	PT	MAZ_Q6	UUu
FOXI2	PT	MYOGNF1_01	UUu
HMX1	PT	MAZ_Q6	UUu
CACNA1A	PT	SP1_Q6_01	UUu
GFAP	PT	E12_Q6	UUu
CCDC151	PT	E12_Q6	UUu
ZNF575	PT	EGR_Q6	UUu
UNC5B	PT	SF1_Q6	UUu
NXPH4	PT	E12_Q6	UUu
GEMIN4	PT	ZIC3_01	UUu
LAMA5	PT	E12_Q6	UUu
MYLK2	PT	E12_Q6	UUu
SRRM4	PT	EGR2_01	UUu
SOX12	PT	CP2_01	UUu
FGF4	PT	MAZ_Q6	UUu
LENG9	PT	AP4_Q5	UUu
LTBP3	PT	E12_Q6	UUu
TP53I11	PT	EGR2_01	UUu
DNAH17	PT	E12_Q6	UUu
RABEP2	PT	SP1_Q6_01	UUu
SP6	PT	ZIC3_01	UUu
COL1A1	PT	E12_Q6	UUu
SNPH	PT	BACH1_01	UUu
MYLK2	PT	MAZ_Q6	UUu

NOTCH3	PT	MAZ_Q6	UUu
LRRC16B	PT	E12_Q6	UUu
BEND4	PT	MAZ_Q6	UUu
SOX4	PT	AP4_Q5	UUu
GPR173	PT	ZIC3_01	UUu
SRCIN1	PT	AP4_Q5	UUu
ANKRD13A	PT	ZF5_01	UUu
SOX12	PT	MAZ_Q6	UUu
KRT78	PT	CP2_01	UUu
SNPH	PT	AP4_Q5	UUu
PHACTR3	PT	E12_Q6	UUu
FAM181B	PT	MAZ_Q6	UUu
PHC2	PT	ZF5_01	UUu
ADAMTS17	PT	E12_Q6	UUu
STRC	PT	E12_Q6	UUu
PHACTR3	PT	CACBINDINGPROTEIN_Q6	UUu
SOX4	PT	E12_Q6	UUu
KCNH4	PT	NRSF_01	UUu
MOB2	PT	AP4_Q5	UUu
NPAS4	PT	EGR_Q6	UUu
FSTL3	PT	MAZ_Q6	UUu
KRT78	PT	SREBP_Q3	UUu
MYH14	PT	E12_Q6	UUu
RASGRF1	PT	NRSF_01	UUu
NEUROD2	PT	AP4_Q5	UUu
NOVA2	PT	E12_Q6	UUu
AMOTL1	PT	MAZ_Q6	UUu
SLC22A17	PT	AP4_Q5	UUu
RUNDC3A	PT	MAZ_Q6	UUu
LAMA5	PT	MAZ_Q6	UUu
IGSF22	PT	MYOGNF1_01	UUu
FOXI2	PT	MAZ_Q6	UUu
TCF15	PT	MAZ_Q6	UUu
SALL3	PT	POU3F2_02	UUu
NEFH	PT	NRSF_01	UUu
BRSK1	PT	CP2_01	UUu
MYH14	PT	E12_Q6	UUu
NEUROD2	PT	RREB1_01	UUu
MGAT5B	PT	NRSF_01	UUu
CEND1	PT	CP2_01	UUu
TACC2	PT	AREB6_03	UUu
SOX4	PT	EGR2_01	UUu
RGMA	PT	CP2_01	UUu
CACNA1A	PT	EGR_Q6	UUu
SOX12	PT	AP4_Q5	UUu
EMX2	PT	CACBINDINGPROTEIN_Q6	UUu

EMX2	PT	MAZ_Q6	UUu
TP53I11	PT	MAZ_Q6	UUu
PHC2	PT	E12_Q6	UUu
NEFH	PT	E12_Q6	UUu
ACAN	PT	MAZ_Q6	UUu
CELF4	PT	EGR_Q6	UUu
PPM1J	PT	AP4_Q5	UUu
TACC2	PT	E12_Q6	UUu
PPM1J	PT	SP1_Q6_01	UUu
NPAS4	PT	NRSF_01	UUu
BRSK1	PT	E12_Q6	UUu
RGS9BP	PT	MAZ_Q6	UUu
CELF4	PT	MAZ_Q6	UUu
SCUBE1	PT	E12_Q6	UUu
RGAG1	PT	AP4_Q5	UUu
LMNA	PT	ZIC3_01	UUu
COL1A1	PT	CP2_01	UUu
GFAP	PT	SREBP_Q3	UUu
SCUBE2	PT	E12_Q6	UUu
HEBP2	PT	ZIC3_01	UUu
INF2	PT	AP4_Q5	UUu
BRSK1	PT	MYOGNF1_01	UUu
DACT1	PT	AP4_Q5	UUu
CORO2B	PT	E12_Q6	UUu
LAMA5	PT	E12_Q6	UUu
LRRC16B	PT	AP4_Q5	UUu
NEUROD2	PT	SREBP_Q3	UUu
MEGF8	PT	AREB6_03	UUu
SP6	PT	MAZ_Q6	UUu
TACC2	PT	MAZ_Q6	UUu
RIPK4	PT	BACH1_01	UUu
DYSF	PT	BACH1_01	UUu
COL27A1	PT	EGR_Q6	UUu
CDX1	PT	E12_Q6	UUu
RGMA	PT	MAZ_Q6	UUu
CCDC107	PT	POU3F2_02	UUu
MMRN2	PT	AP4_Q5	UUu
CSF3	PT	POU3F2_02	UUu
SOX4	PT	MAZ_Q6	UUu
CELF4	PT	E12_Q6	UUu
CYP46A1	PT	SF1_Q6	UUu
PHACTR3	PT	RREB1_01	UUu
CYP46A1	PT	AP4_Q5	UUu
KCNC3	PT	MAZ_Q6	UUu
ARMCX4	PT	E12_Q6	UUu
PPM1J	PT	E12_Q6	UUu
RAB26	PT	SP1_Q6_01	UUu

CDH15	PT	AREB6_03	UUu
ANKRD13A	PT	SF1_Q6	UUu
SOX3	PT	E12_Q6	UUu
ITPKA	PT	E12_Q6	UUu
EMID1	PT	CACBINDINGPROTEIN_Q6	UUu
IGF2BP1	PT	SREBP1_Q6	UUu
FGF4	PT	E12_Q6	UUu
FOXI1	PT	E12_Q6	UUu
IGF2BP1	PT	RREB1_01	UUu
SPTBN4	PT	MAZ_Q6	UUu
ASGR1	PT	MAZ_Q6	UUu
BRSK1	PT	AREB6_03	UUu
ARHGAP24	PT	AP4_Q5	UUu
GOLGA7	PT	AP4_Q5	UUu
PHC2	PT	MAZ_Q6	UUu
MEGF8	PT	ZIC3_01	UUu
WNK2	PT	E12_Q6	UUu
DNMT3B	PT	AREB6_03	UUu
MYLK2	PT	E12_Q6	UUu
NXPH4	PT	MAZ_Q6	UUu
STRN4	PT	E12_Q6	UUu
DNMT3B	PT	SREBP1_Q6	UUu
CELF4	PT	E12_Q6	UUu
SRF	PT	AP4_Q5	UUu
NRXN2	PT	MAZ_Q6	UUu
FOLR1	PT	ZIC3_01	UUu
KCNK7	PT	SREBP1_Q6	UUu
ACAN	PT	CACBINDINGPROTEIN_Q6	UUu
TONSL	PT	AP4_Q5	UUu
HCN2	PT	MAZ_Q6	UUu
SRCIN1	PT	MAZ_Q6	UUu
SHROOM2	PT	MAZ_Q6	UUu
GFAP	PT	BACH1_01	UUu
MYO7A	PT	AP4_Q5	UUu
ANKRD13A	PT	EGR_Q6	UUu
RND2	PT	SP1_Q6_01	UUu
SUMO1	PT	MAZ_Q6	UUu
RAB26	PT	EGR_Q6	UUu
RBMS1	PT	RREB1_01	UUu
EYA2	PT	AP4_Q5	UUu
FGF4	PT	MYOGNF1_01	UUu
SLC22A17	PT	POU3F2_02	UUu
SRCIN1	PT	E12_Q6	UUu
NPAS4	PT	SP1_Q6_01	UUu
BRSK2	PT	AP4_Q5	UUu
ADAMTSL1	PT	BACH1_01	UUu
MGAT5B	PT	E12_Q6	UUu

NXPH4	PT	SF1_Q6	UUu
ZNF575	PT	EGR2_01	UUu
NOVA2	PT	RREB1_01	UUu
BRSK2	PT	CACBINDINGPROTEIN_Q6	UUu
RAB43	PT	CACBINDINGPROTEIN_Q6	UUu
TCF15	PT	E12_Q6	UUu
MYH7	PT	RREB1_01	UUu
FADS3	PT	SREBP_Q3	UUu
RIPK4	PT	E12_Q6	UUu
ZNF575	PT	E12_Q6	UUu
ERBB3	PT	AREB6_03	UUu
SRRM4	PT	SREBP1_Q6	UUu
LMNA	PT	MAZ_Q6	UUu
ARHGAP24	PT	E12_Q6	UUu
NEUROD2	PT	SREBP1_Q6	UUu
ZDHHC7	PT	SF1_Q6	UUu
FADS3	PT	BACH1_01	UUu
PPP1R1B	PT	EGR2_01	UUu
PHACTR3	PT	E12_Q6	UUu
MYH6	PT	MAZ_Q6	UUu
DPF1	PT	CACBINDINGPROTEIN_Q6	UUu
STRN4	PT	MAZ_Q6	UUu
DTX1	PT	MAZ_Q6	UUu
ERBB3	PT	MAZ_Q6	UUu
MOB2	PT	SF1_Q6	UUu
SERPINF2	PT	E12_Q6	UUu
ARHGEF17	PT	MAZ_Q6	UUu
MEGF8	PT	E12_Q6	UUu
COL1A1	PT	MAZ_Q6	UUu
PPP1R1B	PT	SREBP_Q3	UUu
SRRM4	PT	MAZ_Q6	UUu
IGF2BP1	PT	CACBINDINGPROTEIN_Q6	UUu
MEGF8	PT	AP4_Q5	UUu
GPR173	PT	CP2_01	UUu
LRP10	PT	E12_Q6	UUu
MFAP4	PT	E12_Q6	UUu
KLC3	PT	ZF5_01	UUu
LTBP3	PT	CP2_01	UUu
MEGF8	PT	E12_Q6	UUu
L1CAM	PT	NRSF_01	UUu
SCN4A	PT	AP4_Q5	UUu
WNK2	PT	AP4_Q5	UUu
SRF	PT	MAZ_Q6	UUu
ADRA1D	PT	CACBINDINGPROTEIN_Q6	UUu
ITPKA	PT	MAZ_Q6	UUu
CDX1	PT	EGR_Q6	UUu
KLC3	PT	MAZ_Q6	UUu

RAP2C	PT	MAZ_Q6	UUu
PLEKHN1	PT	CP2_01	UUu
SRCIN1	PT	EGR_Q6	UUu
RBMS1	PT	ZIC3_01	UUu
DNMT3B	PT	SREBP_Q3	UUu
NOTCH3	PT	SP1_Q6_01	UUu
CDX1	PT	MAZ_Q6	UUu
PHACTR3	PT	AREB6_03	UUu
WNT10B	PT	ZIC3_01	UUu
RUNDC3A	PT	E12_Q6	UUu
DRAP1	PT	SP1_Q6_01	UUu
CDH15	PT	AP4_Q5	UUu
DYSF	PT	AP4_Q5	UUu
FSTL3	PT	SF1_Q6	UUu
PCK1	PT	E12_Q6	UUu
PPP1R1B	PT	RREB1_01	UUu
SRF	PT	EGR2_01	UUu
DNAH17	PT	AREB6_03	UUu
FKRP	PT	MAZ_Q6	UUu
SRCIN1	PT	SF1_Q6	UUu
SHROOM2	PT	E12_Q6	UUu
PEG3	PT	SREBP_Q3	UUu
PHACTR3	PT	MAZ_Q6	UUu
NEUROD2	PT	MAZ_Q6	UUu
STRN4	PT	POU3F2_02	UUu
NPAS4	PT	E12_Q6	UUu
ARHGAP24	PT	E12_Q6	UUu
SRRM4	PT	NRSF_01	UUu
COL4A6	PT	MAZ_Q6	UUu
IGF2BP1	PT	EGR2_01	UUu
RYR1	PT	AP4_Q5	UUu
DNMT3B	PT	MAZ_Q6	UUu
CCDC33	PT	E12_Q6	UUu
ITPKA	PT	EGR2_01	UUu
RIPK4	PT	AREB6_03	UUu
RUNDC3A	PT	NRSF_01	UUu
NPAS4	PT	MAZ_Q6	UUu
SCUBE2	PT	ZF5_01	UUu
OGDHL	PT	NRSF_01	UUu
SMTN	PT	SREBP1_Q6	UUu
IGF2BP1	PT	SP1_Q6_01	UUu
MYH6	PT	E12_Q6	UUu
LAMA5	PT	AP4_Q5	UUu
PPM1J	PT	MAZ_Q6	UUu
RAB26	PT	E12_Q6	UUu
FKRP	PT	E12_Q6	UUu
OGDHL	PT	SF1_Q6	UUu

RUNDC3A	PT	BACH1_01	UUu
KCNH4	PT	MAZ_Q6	UUu
L1CAM	PT	AP4_Q5	UUu
TBX10	PT	E12_Q6	UUu
GFAP	PT	E12_Q6	UUu
EMX2	PT	SREBP_Q3	UUu
KCNG4	PT	SF1_Q6	UUu
RGMA	PT	E12_Q6	UUu
MYH14	PT	BACH1_01	UUu
MRC2	PT	MAZ_Q6	UUu
RGMA	PT	AREB6_03	UUu
FNDC8	PT	AP4_Q5	UUu
H2AFY2	PT	EGR2_01	UUu
NEUROD2	PT	MYOGNF1_01	UUu
ROBO4	PT	MAZ_Q6	UUu
ENTPD3	PT	E12_Q6	UUu
PPM1J	PT	AREB6_03	UUu
NEFH	PT	MAZ_Q6	UUu
CSF3	PT	MAZ_Q6	UUu
GFAP	PT	SREBP1_Q6	UUu
CTGF	PT	MAZ_Q6	UUu
GPRC5B	PT	E12_Q6	UUu
RP1L1	PT	E12_Q6	UUu
RASGRF1	PT	ZF5_01	UUu
DNMT3B	PT	E12_Q6	UUu
UNC5B	PT	E12_Q6	UUu
LMNA	PT	POU3F2_02	UUu
HEBP2	PT	CP2_01	UUu
NEUROD2	PT	CACBINDINGPROTEIN_Q6	UUu
RAX	PT	MAZ_Q6	UUu
IGF2BP1	PT	E12_Q6	UUu
EDN3	PT	AP4_Q5	UUu
FADS3	PT	SREBP1_Q6	UUu
NXPH4	PT	SP1_Q6_01	UUu
RBMS1	PT	E12_Q6	UUu
IGF2BP1	PT	MAZ_Q6	UUu
FKRP	PT	POU3F2_02	UUu
PHC2	PT	POU3F2_02	UUu
SP6	PT	E12_Q6	UUu
CSF3	PT	E12_Q6	UUu
CELF4	PT	ZF5_01	UUu
SLC22A17	PT	E12_Q6	UUu
KIF7	PT	AP4_Q5	UUu
RYR1	PT	E12_Q6	UUu
EXT1	PT	E12_Q6	UUu
CPAMD8	PT	NRSF_01	UUu
RAB26	PT	AP4_Q5	UUu

SCUBE2	PT	MAZ_Q6	UUu
SRCIN1	PT	SREBP_Q3	UUu
ITPKA	PT	ZF5_01	UUu
LMNA	PT	BACH1_01	UUu
EMX2	PT	SREBP1_Q6	UUu
NPAS4	PT	CP2_01	UUu
BEND4	PT	AP4_Q5	UUu
NEUROD2	PT	EGR_Q6	UUu
NRXN2	PT	SF1_Q6	UUu
RHBDL1	PT	E12_Q6	UUu
ZNF580	PT	MAZ_Q6	UUu
CDX1	PT	AP4_Q5	UUu
SLC22A17	PT	EGR_Q6	UUu
SP6	PT	POU3F2_02	UUu

Supplementary table 10. Primers used for quantitative real-time RT-PCR validation.

Gene symbol	Primer direction	Sequence (5'~3')
ATP5B	Forward	GATGAGGGACTACCACCAATTC
	Reverse	CTTACTGTGCTCTCACCCAAA
CD300LF	Forward	ACACTTACTGGTGTGGAATTGA
	Reverse	GGGAGCTGCTAGTTTCTTCTT
F11R	Forward	CCACCAGACTCGTTTGCTATAA
	Reverse	GTCACGGACTTGAAGGTGATAC
FKBP1A	Forward	GGGACAGAAACAAGCCCTTTA
	Reverse	AGTGGCACCATAGGCATAATC
HAX1	Forward	TCTTGGAGAGTGATGCAAGAAG
	Reverse	GGTCCATAGGCCATACATCATC
MYNN	Forward	GGAAGGCATTTGCTGTCTCTA
	Reverse	GTTGAGCTCTCCTGAGGAAATAA
NDUFAF1	Forward	CATGAGCTTCCGCTTGATAAGA
	Reverse	TGGATCAGTAAACACGCCAATA
PDIA6	Forward	GAATGGAAGAAAGCAGCAACTG
	Reverse	GGAAATCCCTGAACACCATACT
PSMD2	Forward	CCATGAGACTTGGTTCCATCTT
	Reverse	TGGACTTTGAATCTCCCATCAC
SLC3A2	Forward	AGTCTCTTGCAATCGGCTAAA
	Reverse	GTGTCAACCTGAGTGGAGAAC
SOCS4	Forward	GATGGTGATGGCAGCAGTTA
	Reverse	CTGGGCACTTTCTGGATGTAT

Supplementary table 11. Target sequences of miRNA for qRT-PCR.

Name on array	Assay name	Assay ID	Target sequence
hsa-miR-34a	hsa-miR-34a	426	UGGCAGUGUCUUAGCUGGUUGU
hsa-miR-551a	hsa-miR-551a	1519	GCGACCCACUCUUGGUUUCCA
miR-337-3p	hsa-miR-337-3p	2157	CUCCUAUAUGAUGCCUUUCUUC
miR-198	hsa-miR-198	2273	GGUCCAGAGGGGAGAUAGGUUC
miR-877*	hsa-miR-877*	241029_mat	UCCUCUUCUCCCUCCUCCCAG
miR-16	hsa-miR-16	391	UAGCAGCACGUAAAUAUUGGCG
hsa-miR-127-3p	hsa-miR-127	452	UCGGAUCCGUCUGAGCUUGGCU
hsa-miR-379	mmu-miR-379	1138	UGGUAGACUAUGGAACGUAGG
miR-130a	hsa-miR-130a	454	CAGUGCAAUGUUAAAAGGGCAU
hsa-miR-936	hsa-miR-936	2179	ACAGUAGAGGGAGGAAUCGCAG
	hsa-miR-191	2299	CAACGGAAUCCCAAAAGCAGCUG
